# Supplementary material for: Evolution of the electronic structure in open-shell donor-acceptor organic semiconductors
Source: Nat Commun. 2021 Oct 7;12:5889. doi: 10.1038/s41467-021-26173-3 (PMC8497548; doi:10.1038/s41467-021-26173-3)
Supplement: Supplementary file 1 — Supplementary Information [file 41467_2021_26173_MOESM1_ESM.pdf]

Evolution of the Electronic Structure in Open-Shell Donor-Acceptor Organic Semiconductors

Zhongxin Chen,[1#] *Wenqiang Li,*[1#] *Md Abdus Sabuj,*[2#] *Yuan Li,*[1*] *Weiya Zhu,*[1] *Miao Zeng,*[1] *Chandra S. Sarap,*[2] *Md Masrul Huda,*[2] *Xianfeng Qiao,*[1] *Xiaobin Peng,*[1] *Dongge Ma,*[1] *Yuguang Ma,*[1] *Neeraj Rai,*[2*] *and Fei Huang*[1*]

Affiliations

[1] Institute of Polymer Optoelectronic Materials and Devices, State Key Laboratory of Luminescent Materials and Devices, South China University of Technology, Guangzhou 510640, P. R. China

[2] Dave C. Swalm School of Chemical Engineering and Center for Advanced Vehicular Systems, Mississippi State University, Mississippi State, MS 39762, United States.

E-mail: celiy@scut.edu.cn, neerajrai@che.msstate.edu, msfhuang@scut.edu.cn

Table of contents

1. Molecular structures and synthetic details S3

1.1. Molecular structures S3

1.2. Materials and methods S4

2. Results S15

2.1. HPLC, UV-vis-NIR absorption, PL emission and CV spectra S15

2.2. Theoretical calculations S18

2.3. Electron spin resonance spectra S30

2.4. X-ray crystallography and packing motifs of the single crystals S36

2.5. X-ray crystallographic data for TPAOMe-TDPP S39

2.6. X-ray crystallographic data for Py-TDPP S50

2.7. X-ray crystallographic data for An-TDPP S61

2.8. X-ray crystallographic data for Flu-TDPP S67

2.9. X-ray crystallographic data for TPAOMe-TDPP-C4 .... S85

2.10. X-ray crystallographic data for Flu-TDPP-C8 .... S93

2.11. ^{1}H, ^{13}C NMR and mass spectra S116

2.12 Optimized coordinates S143

Reference S167

1. Molecular structures and synthetic details

1.1. Molecular structures

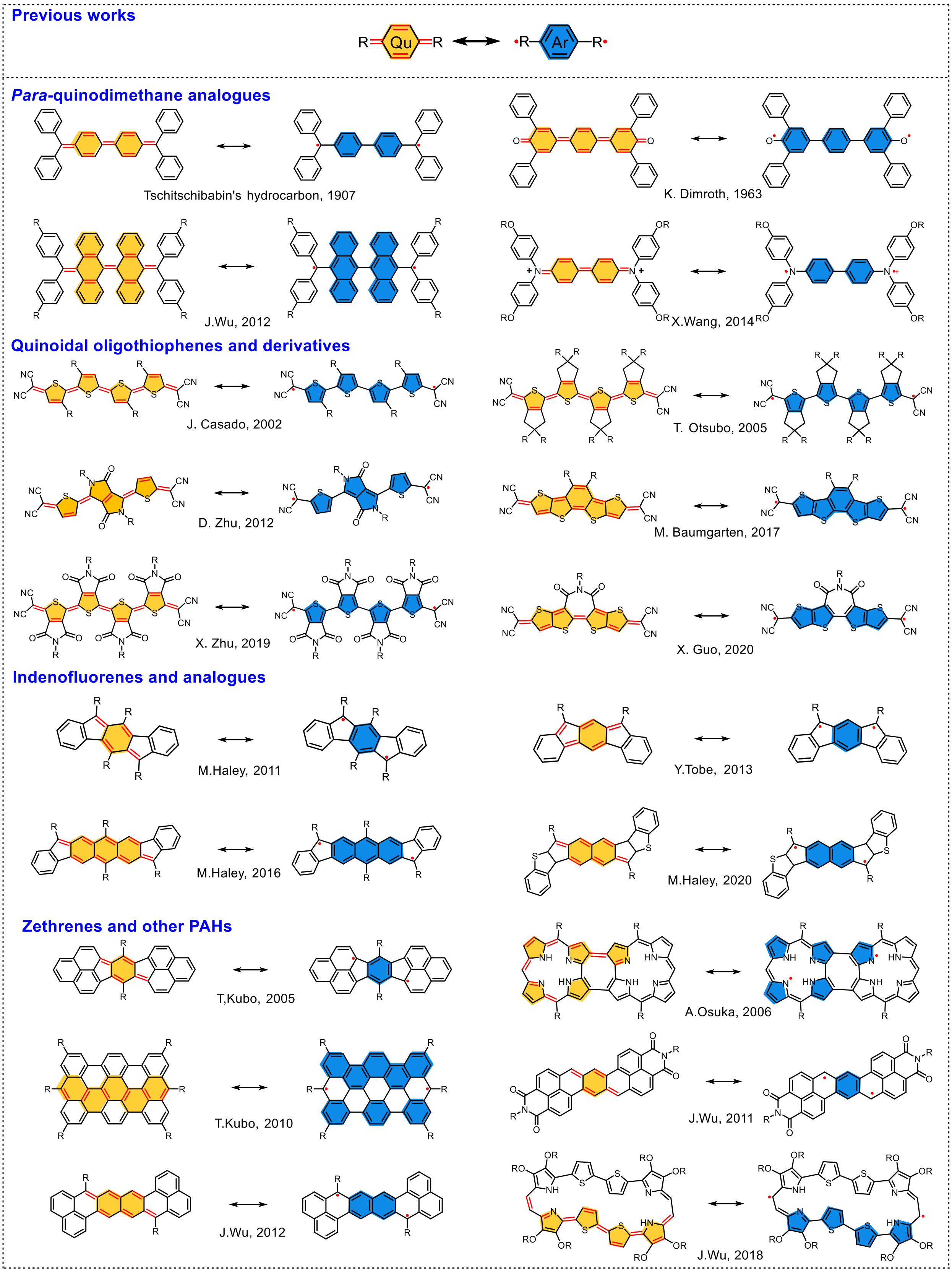

Supplementary Figure 1. The molecular structures of open-shell species in previous work including *para*-quinodimethane analogues,[1-2] quinoidal oligothiophenes and derivatives,[3-8] indenofluorenes based materials,[9-11] and quinoidal polycyclic aromatic hydrocarbons.[12-15]

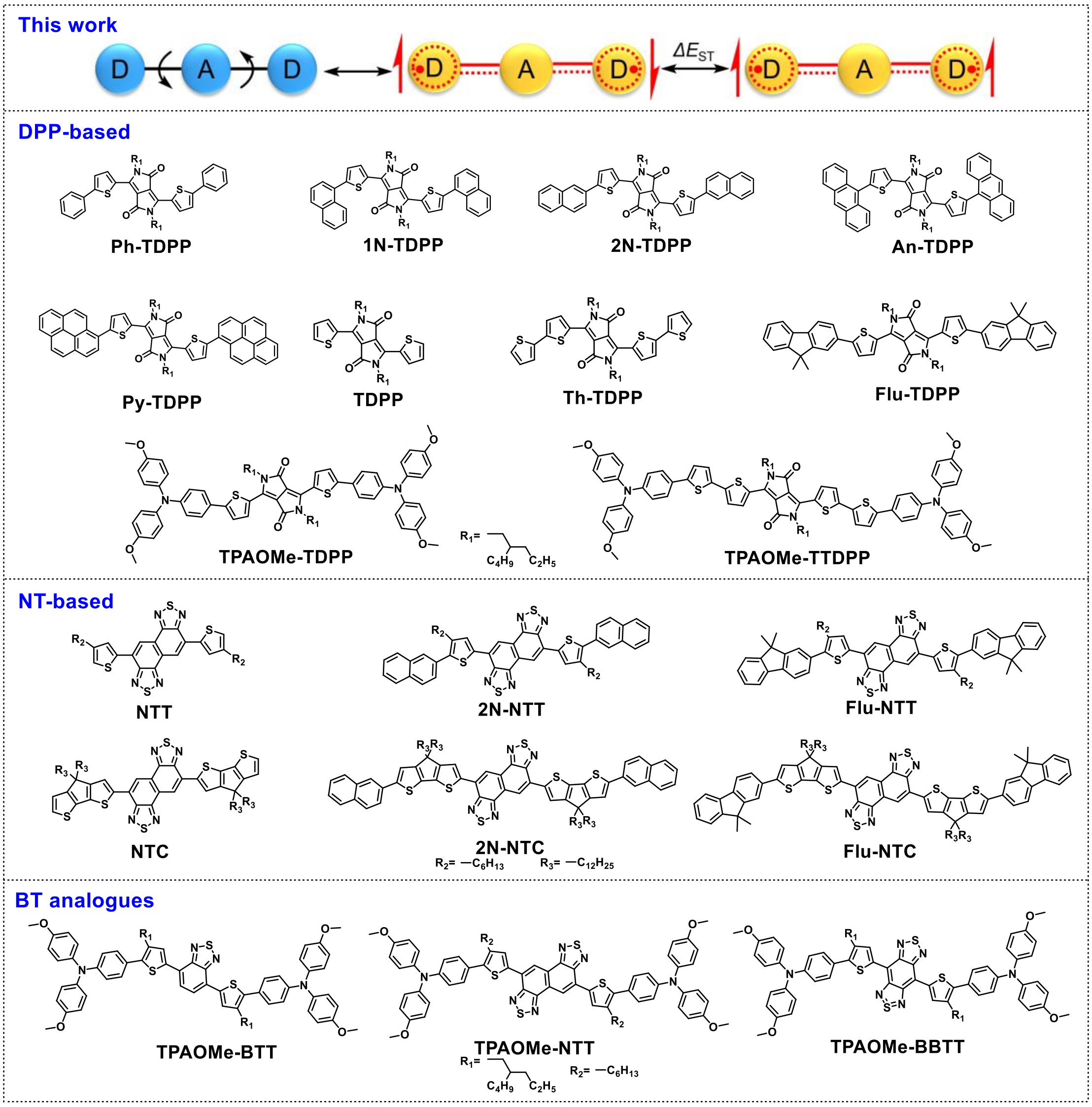

Supplementary Figure 2. The molecular structures considered in this work including DPP-based and NT-based small molecules, BT, and BBT analogues.

1.2. Materials and methods

General remarks. All manipulations of air/and or moisture-sensitive compounds were performed under an inert atmosphere in a nitrogen-filled glovebox, or using standard Schlenk techniques. Reagents, unless otherwise specified, were purchased from Sigma-Aldrich and SunaTech Inc. and used without further purification. Chloroform ($CHCl_3$), dichloromethane, hexanes, and acetonitrile were degassed and dried over 4 Å molecular sieves prior to use. Deuterated solvents (chloroform-*d*, dichloromethane-d_2) were purchased from Aldrich and used as received. Tetrakis(triphenylphosphine)palladium (0) was purchased from TCI Shanghai and used as received. 4*H*-cyclopenta[2,1-*b*:3,4-*b'*]dithiophene (CPDT),[16] 4,4-didodecyl-4*H*-cyclopenta[2,1-*b*:3,4-*b'*]dithiophene (C_{12}-CPDT),[17] tributyl(4,4-didodecyl-4*H*-cyclopenta[2,1-*b*:3,4-*b'*]dithiophen-2-yl)stannane (C_{12}-CPDT-Sn),[16] 5,10-dibromonaphtho[1,2-*c*:5,6-*c'*]bis([1,2,5]thiadiazole) (NT-Br_2), 5,10-bis(4-hexylthiophen-2-yl)napht-ho[1,2-*c*:5,6-*c'*]bis([1,2,5]thiadiazole) (NTT) and the dibromosubstituted precursor 5,10-bis(5-bromo-4-hexylthiophen-2-yl)naphtho[1,2-*c*:5,6-*c'*]bis([1,2,5]thiadiazole) (NTT-Br_2)[18] were prepared according to literature procedures. 2,5-bis(2-ethylhexyl)-3,6-di(thiophen-2-yl)-2,5-dihydropyrrolo[3,4-*c*]pyrrole-1,4-dione (TDPP) was commercially available. 2,5-bis(2-ethylhexyl)-3,6-bis(5-phenylthiophen-2-yl)-2,5-dihydropyrrolo[3,4-*c*]pyrrole-1,4-dione (Ph-TDPP), 2,5-bis(2-ethylhexyl)-3,6-bis(5-(naphthalen-1-yl)thiophen-2-yl)-2,5-dihydropyrr-

olo[3,4-*c*]pyrole-1,4-dione (1N-TDPP), 2,5-bis(2-ethylhexyl)-3,6-bis(5-(naphthalen-2-yl)thiophen-2-yl)-2,5-dihydropyrrolo[3,4-*c*]pyrrole-1,4-dione (2N-TDPP), 2,5-bis(2-ethylhexyl)-3,6-bis(5-(pyren-4-yl)thiophen-2-yl)-2,5-dihydropyrrolo[3,4-*c*]pyrrole-1,4-dione (Py-TDPP), 3,6-bis(5-(4-(bis(4-methoxyphenyl)amino)phenyl)thiophen-2-yl)-2,5-bis(2-ethylhexyl)-2,5-dihydropyrrolo[3,4-*c*]pyrrole-1,4-dione (TPAOMe-TDPP) and 3,6-di([2,2'-bithiophen]-5-yl)-2,5-bis(2-ethylhexyl)-2,5-dihydropyrrolo[3,4-*c*]pyrrole-1,4-dione (Th-TDPP), 4,4'-(5,5'-(benzo[*c*][1,2,5]thiadiazole-4,7-diyl)bis(3-hexylthiophene-5,2-diyl))bis (*N,N*-bis(4-methoxy-phenyl)aniline) (TPAOMe-BTT) were synthesized according to previously published procedures.[19] ^{1}H and ^{13}C NMR spectra were collected on a Bruker Avance III 400 MHz spectrometer and chemical shifts, δ (ppm), were referenced to the residual solvent impurity peak of the given solvent. Solutions tested in ^{13}C NMR are saturated. Data reported as: s = singlet, d = doublet, t = triplet, m = multiplet, br = broad; coupling constant(s), *J* are given in Hz. Flash chromatography was performed on a Teledyne Isco CombiFlash Purification System using Redi*Sep* Rf prepacked columns. The melting point of each material was estimated by differential scanning calorimetry DSC (Netzsch DSC 200F3) at a heating rate of 10 °C·min^{-1} under nitrogen. Element composition (C, H, S, N) was collected on a Elementar Vario EL cube. The composition of O element was calculated by subtracting the composition of C, H, S, N elements.

HPLC analysis was conducted using Cosmosil 5C_{18}-MS-II column with the chromatographic conditions as follow. (1) For NTT, 2N-NTT, Flu-NTT, NTC, 2N-NTC, Flu-NTC, Ph-TDPP, 2N-TDPP, flow rate = 0.3 ml min^{-1}, eluent = CH_2Cl_2/isopropanol =1:1, column temperature = 25 °C. (2) For 1N-TDPP, Py-TDPP, Flu-TDPP, Flu-TDPP-C8, TPAOMe-TDPP, TPAOMe-TDPP-C4, TPAOMe-TTDPP, flow rate = 0.3 ml min^{-1}, eluent = CH_2Cl_2/isopropanol =4:1, column temperature = 25 °C. (3) For An-TDPP, Th-TDPP, TPAOMe-BBTT, flow rate = 0.3 ml min^{-1}, eluent = CH_2Cl_2/isopropanol =19:1, column temperature = 25 °C.

2,5-bis(2-ethylhexyl)-3,6-bis(5-phenylthiophen-2-yl)-2,5-dihydropyrrolo[3,4-*c*]pyrrole-1,4-dione (Ph-TDPP)

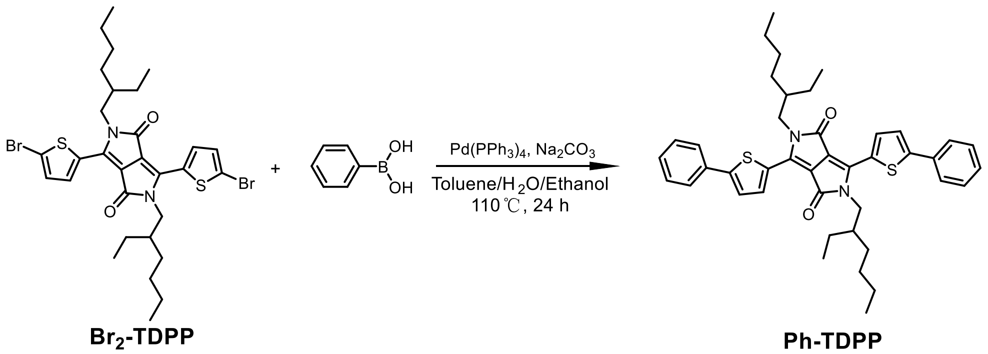

Ph-TDPP was synthesized according to previously published procedures.[19] Melting point (Mp): 213 °C; ^{1}H NMR (400 MHz, chloroform-*d*) δ 8.96 (s, 2H), 7.68 (d, *J* = 6.2 Hz, 4H), 7.50 – 7.29 (m, 8H), 4.14 – 4.02 (m, 4H), 1.94 (d, *J* = 5.9 Hz, 2H), 1.45 – 1.23 (m, 16H), 0.92 (t, *J* = 7.4 Hz, 6H), 0.87 (t, *J* = 7.0 Hz, 6H). UV/Vis: λ_{max} 578 nm; MOLDI-TOF-MS (m/z): Calcd. for $C_{42}H_{48}N_2O_2S_2$: *m/z*: 676.3157. Found: 676.3426; analysis (calcd., found for $C_{42}H_{48}N_2O_2S_2$): C(74.52, 74.82), H(7.15, 7.12), S(9.47, 9.31), N(4.14, 4.20), O(4.73, 4.55).

2,5-bis(2-ethylhexyl)-3,6-bis(5-(naphthalen-1-yl)thiophen-2-yl)-2,5-dihydropyrrolo[3,4-*c*]pyrrole-1,4-dione (1N-TDPP)

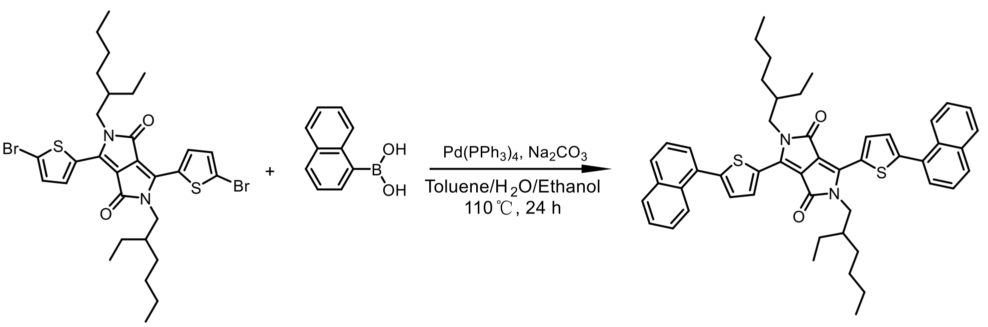

1N-TDPP was synthesized according to previously published procedures.[19] Melting point (Mp): 183 °C; ^{1}H NMR (400 MHz, chloroform-*d*) δ 9.07 (s, 2H), 8.31 – 8.25 (m, 2H), 8.00 – 7.88 (m, 4H), 7.69 – 7.43 (m, 10H), 4.11 (p, *J* = 7.4 Hz, 4H), 2.00 (d, *J* = 5.4 Hz, 2H), 1.49 – 1.24 (m, 16H), 0.93 (t, *J* = 7.4 Hz, 6H), 0.85 (t, *J* = 7.0 Hz, 6H). ^{13}C NMR (101 MHz, $CDCl_3$) δ 161.88, 147.77, 140.17, 136.10, 133.95, 131.41, 131.08, 129.86, 129.45, 128.93, 128.58, 128.37, 126.98, 126.38, 125.38, 125.29, 108.13, 46.08, 39.32, 30.33, 28.51, 23.65, 23.14, 14.08, 10.59. UV/Vis: λ_{max} 600 nm; MOLDI-TOF-MS (m/z): Calcd. for $C_{50}H_{52}N_2O_2S_2$: *m/z*: 776.3470. Found: 776.3426; analysis (calcd., found for $C_{50}H_{52}N_2O_2S_2$): C(77.28, 77.21), H(6.75, 6.31), S(8.25, 8.11), N(3.60, 3.55), O(4.12, 4.82).

2,5-bis(2-ethylhexyl)-3,6-bis(5-(naphthalen-2-yl)thiophen-2-yl)-2,5-dihydropyrrolo[3,4-*c*]pyrrole-1,4-dione (2N-TDPP)

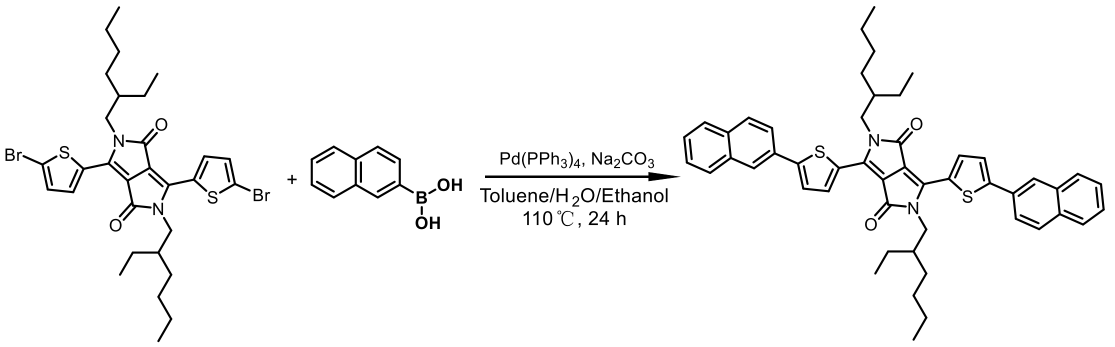

2N-TDPP was synthesized according to previously published procedures.[19] Melting point (Mp): 227 °C; ^{1}H NMR (400 MHz, Chloroform-*d*) δ 9.00 (s, 2H), 8.13 (s, 2H), 7.91 – 7.79 (m, 8H), 7.60 (dd, *J* = 4.1, 2.7 Hz, 2H), 7.53 (d, *J* = 6.6 Hz, 2H), 4.13 (t, *J* = 6.7 Hz, 4H), 1.98 (d, *J* = 7.0 Hz, 2H), 1.45 – 1.30 (m, 16H), 0.95 (d, *J* = 7.3 Hz, 6H), 0.91 (d, *J* = 6.8 Hz, 6H). UV/Vis: λ_{max} 650 nm; MOLDI-TOF-MS (m/z): Calcd for $C_{50}H_{52}N_2O_2S_2$: *m/z*: 776.3470. Found: 776.3467; analysis (calcd., found for $C_{50}H_{52}N_2O_2S_2$): C(77.28, 77.32), H(6.75, 6.33), S(8.25, 8.10), N(3.60, 3.50), O(4.12, 4.75).

3,6-bis(5-(anthracen-9-yl)thiophen-2-yl)-2,5-bis(2-ethylhexyl)-2,5-dihydropyrrolo[3,4-*c*]pyrrole-1,4-dione (An-TDPP)

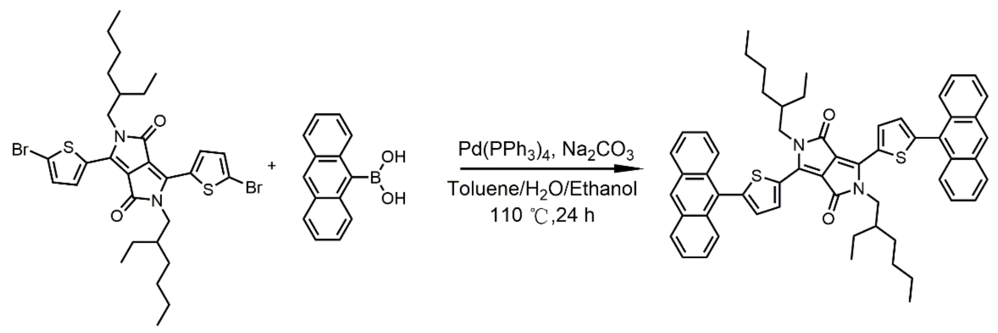

Br$_2$-TDPP **An-TDPP**

3,6-bis(5-bromothiophen-2-yl)-2,5-bis(2-ethylhexyl)-2,5-dihydropyrrolo[3,4-*c*]pyrrole-1,4-dione (Br$_2$-TDPP) (200 mg, 0.29 mmol) was dissolved in a mixture of toluene (20 ml) and ethanol (8 ml). Anthracen-9-ylboronic acid (193.2 mg, 0.87 mmol), Na_2CO_3 (3 mL, 2M), Pd (PPh_3)$_4$ (16.8 mg, 0.0145 mmol) were added to the mixture. The reaction mixture was cooled to room temperature, extracted with 60 ml dichloromethane, and washed with saturated brine water (3×100 ml) three times, dried over anhydrous $MgSO_4$, and volatiles were removed *in vacuo*. The crude product was purified by column chromatography on silica gel using dichloromethane and petroleum ether and recrystallization resulting in a deep-brown solid An-TDPP (171 mg, 67%). Melting point (Mp): 209 °C; ^{1}H NMR (400 MHz, chloroform-*d*) δ 9.23 (s, 2H), 8.59 (s, 2H), 8.07 (d, *J* = 8.2 Hz, 4H), 7.92 (d, *J* = 8.6 Hz, 4H), 7.59 – 7.35 (m, 10H), 4.18 – 4.04 (m, 4H), 2.03 (d, *J* = 5.8 Hz, 2H), 1.46 – 1.21 (m, 16H), 0.97 – 0.86 (m, 6H), 0.85 – 0.72 (m, 6H). ^{13}C NMR (101 MHz, $CDCl_3$) δ 161.93, 145.14, 140.28, 136.14, 131.58, 131.17, 128.84, 128.49, 126.74, 126.45, 126.14, 125.46, 108.18, 46.11, 39.31, 30.22, 28.41, 23.50, 23.06, 14.00, 10.50. UV/Vis: λ_{max} 582 nm; MOLDI-TOF-MS (m/z): calcd. for $C_{58}H_{56}N_2O_2S_2$, 899.3783; found: 899.3773; analysis (calcd., found for $C_{58}H_{56}N_2O_2S_2$): C(79.41, 79.11), H(6.43, 6.41), S(7.31, 7.37), N(3.19, 3.15), O(3.65, 3.97).

2,5-bis(2-ethylhexyl)-3,6-bis(5-(pyren-1-yl)thiophen-2-yl)-2,5-dihydropyrrolo[3,4-*c*]pyrrole-1,4-dione (Py-TDPP)

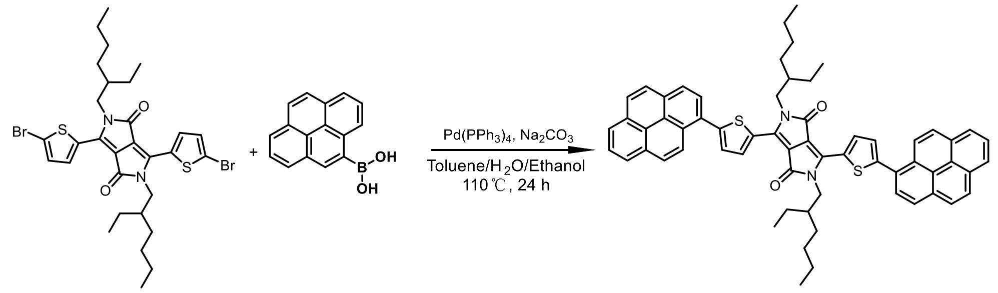

Br$_2$-TDPP **Py-TDPP**

Py-TDPP was synthesized according to previously published procedures.[19] Melting point (Mp): 217 °C; ^{1}H NMR (500 MHz, Chloroform-*d*) δ 9.14 (s, 2H), 8.57 (d, *J* = 9.2 Hz, 2H), 8.35 – 7.79 (m, 16H), 7.60 (d, *J* = 3.6 Hz, 2H), 4.15 (q, *J* = 15.2, 11.7 Hz, 4H), 2.07 (s, 2H), 1.53 – 1.23 (m, 16H), 0.97 (t, *J* = 7.3 Hz, 6H), 0.88 (t, *J* = 7.0 Hz, 6H). UV/Vis: λ_{max} 585 nm; MOLDI-TOF-MS (m/z): Calcd. for $C_{62}H_{56}N_2O_2S_2$: *m/z*: 924.3873. Found: 924.3883; analysis (calcd., found for $C_{62}H_{56}N_2O_2S_2$): C(80.48, 80.15), H(6.10, 6.12), S(6.93, 7.06), N(3.03, 2.95), O(3.46, 3.73).

3,6-di([2,2'-bithiophen]-5-yl)-2,5-bis(2-ethylhexyl)-2,5-dihydropyrrolo[3,4-*c*]pyrrole-1,4-dione (Th-TDPP)

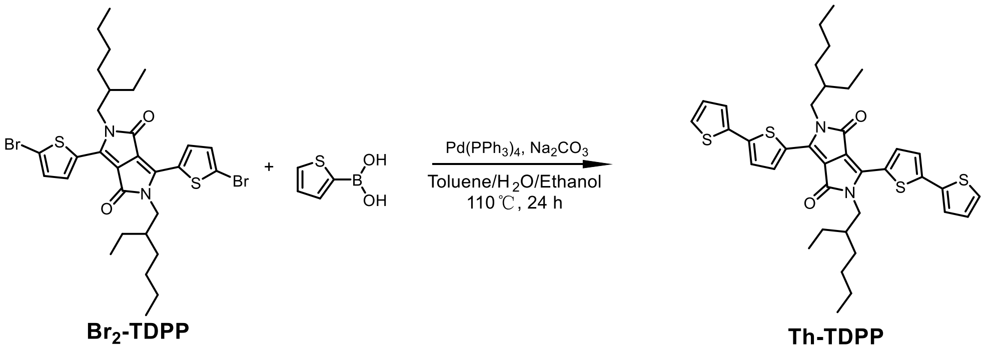

Th-TDPP was synthesized according to previously published procedures.[19] Melting point (Mp): 186 ^oC; ^1H NMR (400 MHz, Chloroform-*d*) δ 8.92 (s, 2H), 7.32 (d, *J* = 4.1 Hz, 6H), 7.11 – 7.05 (m, 2H), 4.05 (p, *J* = 7.2 Hz, 4H), 1.92 (d, *J* = 5.8 Hz, 2H), 1.44 – 1.21 (m, 16H), 0.89 (dt, *J* = 16.8, 7.3 Hz, 12H). UV/Vis: λ_{max} 580 nm; analysis (calcd., found for $C_{38}H_{44}N_2O_2S_4$): C(66.24, 65.70), H(6.44, 6.22), S(18.61, 18.99), N(4.07, 3.95), O(4.64, 5.14).

3,6-bis(5-(9,9-dimethyl-9*H*-fluoren-2-yl)thiophen-2-yl)-2,5-bis(2-ethylhexyl)-2,5-dihydropyrrolo[3,4-*c*]pyrrole-1,4-dione (Flu-TDPP)

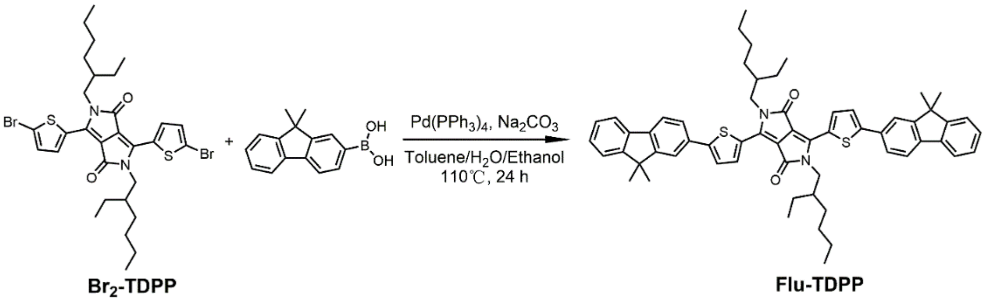

Flu-TDPP was synthesized from Br_2-TDPP (200 mg, 0.29 mmol) and (9,9-dimethyl-9*H*-fluoren-2-yl)boronic acid (174 mg, 0.73 mmol) using an analogous procedure similar to that for An-TDPP resulting in a brown solid (210 mg, 79 %). Melting point (Mp): 216 ^oC; ^1H NMR (400 MHz, chloroform-*d*) δ 9.00 (s, 2H), 7.85 – 7.58 (m, 8H), 7.52 (d, *J* = 4.1 Hz, 2H), 7.68–7.46 (m, 2H), 7.39 – 7.35 (m, 4H), 4.18 – 4.07 (t, *J* = 7.9 Hz, 4H), 1.97 (m, 2H), 1.45 – 1.25 (m, 16H), 0.96 – 0.88 (m, 12H). ^{13}C NMR (101 MHz, $CDCl_3$) δ 153.98, 127.81, 127.18, 122.69, 120.31, 77.35, 47.04, 46.01, 39.33, 30.50, 28.65, 27.16, 23.77, 23.18, 14.14, 10.67. UV/Vis: λ_{max} 616 nm; MOLDI-TOF-MS (m/z): calcd. for $C_{60}H_{64}N_2O_2S_2$, 908.4409; found: 908.4425; analysis (calcd., found for $C_{60}H_{64}N_2O_2S_2$): C(79.25, 79.31), H(7.09, 7.13), S(7.05, 7.07), N(3.08, 3.10), O(3.52, 3.40).

3,6-bis(5-(9,9-dimethyl-9*H*-fluoren-2-yl)thiophen-2-yl)-2,5-dioctyl-2,5-dihydropyrrolo-[3,4-*c*]pyrrole-1,4-dione (DPPT-Flu-C8)

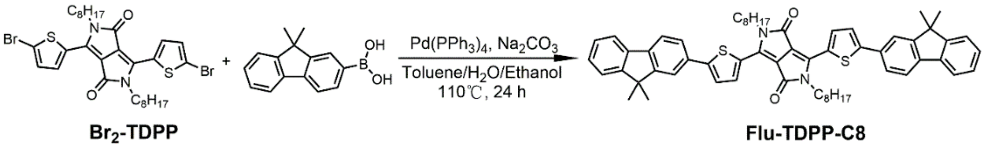

Flu-TDPP-C8 was synthesized from Br_2-TDPP (200 mg, 0.29 mmol) and (9,9-dimethyl-9*H*-fluoren-2-yl)boronic acid (174 mg, 0.73 mmol) in a procedure similar to that for Flu-TDPP resulting in a brown solid (200 mg, 75%). ^{1}H NMR (400 MHz, chloroform-*d*) δ 9.00 (s, 2H), 7.85 – 7.58 (m, 8H), 7.54 (d, *J* = 4.1 Hz, 2H), 7.49 – 7.44 (m, 2H), 7.40 – 7.32 (m, 4H), 4.17 (t, *J* = 7.9 Hz, 4H), 1.83 (p, *J* = 7.6 Hz, 4H), 1.54 – 1.23 (m, 20H), 0.91 – 0.84 (m, 6H). ^{13}C NMR (101 MHz, $CDCl_3$) δ 154.59, 153.98, 140.21, 139.36, 138.36, 136.79, 132.09, 128.56, 127.83, 127.20, 125.49, 124.56, 122.70, 120.63, 120.30, 47.05, 42.34, 31.84, 30.06, 29.72, 29.29, 27.17, 22.67, 14.12. MOLDI-TOF-MS (m/z): calcd. for $C_{60}H_{64}N_2O_2S_2$, 908.4409; found: 908.4469.

3,6-bis(5-(4-(bis(4-methoxyphenyl)amino)phenyl)thiophen-2-yl)-2,5-bis(2-ethylhexyl)-2,5-dihydropyrrolo[3,4-*c*]pyrrole-1,4-dione (TPAOMe-TDPP)

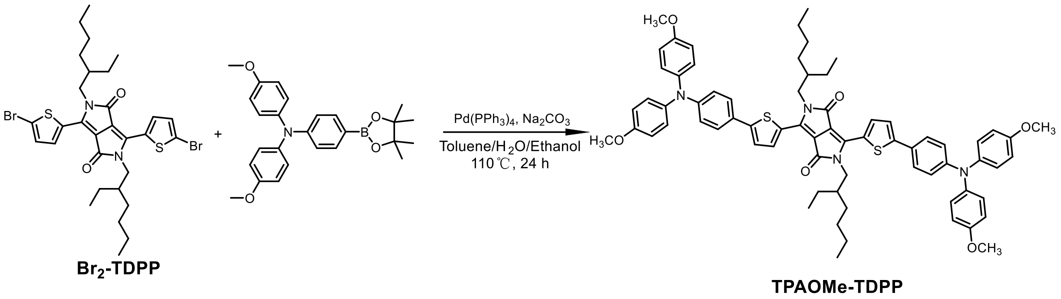

TPAOMe-TDPP was synthesized according to previously published procedures.[19] Melting point (Mp): 207 °C; ^{1}H NMR (400 MHz, Chloroform-*d*) δ 8.97 (s, 2H), 7.44 (s, 6H), 7.08 (s, 8H), 6.86 (d, *J* = 8.5 Hz, 12H), 4.02 (d, *J* = 25.2 Hz, 4H), 3.81 (s, 12H), 1.95 (s, 2H), 1.44 – 1.18 (m, 16H), 0.94 – 0.79 (m, 12H). UV/Vis: λ_{max} 610 nm; MOLDI-TOF-MS (m/z): Calcd for $C_{70}H_{74}N_4O_6S_2$: *m/z*: 1130.5050. Found: 1130.5164; analysis (calcd., found for $C_{70}H_{74}N_4O_6S_2$): C(74.31, 73.95), H(6.59, 6.41), S(5.67, 5.71), N(4.95, 4.82), O(8.48, 9.11).

3,6-bis(5-(4-(bis(4-methoxyphenyl)amino)phenyl)thiophen-2-yl)-2,5-dibutyl-2,5-dihydropyrrolo[3,4-*c*]pyrrole-1,4-dione (TPAOMe-TDPP-C4)

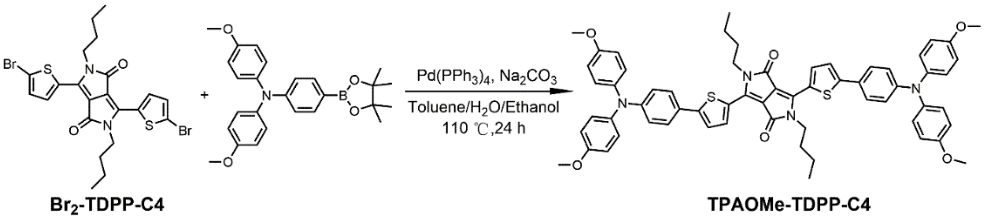

TPAOMe-TDPP-C4 was prepared from 3,6-bis(5-bromothiophen-2-yl)-2,5-dibutyl-2,5-dihydropyrrolo[3,4-*c*]pyrrole-1,4-dione (200 mg, 0.35 mmol) and 4-methoxy-*N*-(4-methoxyphenyl)-*N*-(4-(4,4,5,5-tetramethyl-1,3,2-dioxaborolan-2-yl)phenyl)aniline (378.1 mg, 0.88 mmol) using a procedure similar to that for TPAOMe-TDPP resulting in a dark-brown solid (278.3 mg, 75%). ^{1}H NMR (400 MHz, Chloroform-*d*) δ 9.01 (s, 2H), 7.39 (d, *J* = 56.7 Hz, 6H), 7.09 (d, *J* = 8.4 Hz, 8H), 6.95 – 6.81 (m, 12H), 4.10 (d, *J* = 8.0 Hz, 4H), 3.81 (s, 12H), 1.83 – 1.71 (m, 4H), 1.47 (h, *J* = 7.4 Hz, 4H), 0.98 (t, *J* = 7.4 Hz, 6H). ^{13}C NMR (101 MHz, $CDCl_3$) δ 156.40, 127.26, 114.96, 114.80, 55.52, 42.05, 20.26, 13.83. MOLDI-TOF-MS (m/z): calcd. for $C_{62}H_{58}N_4O_6S_2$, 1018.3798; found: 1018.3806.

3,6-bis(5'-(4-(bis(4-methoxyphenyl)amino)phenyl)-[2,2'-bithiophen]-5-yl)-2,5-bis(2-ethylhexyl)-2,5-dihydropyrrolo[3,4-*c*]pyrrole-1,4-dione (TPAOMe-TTDPP)

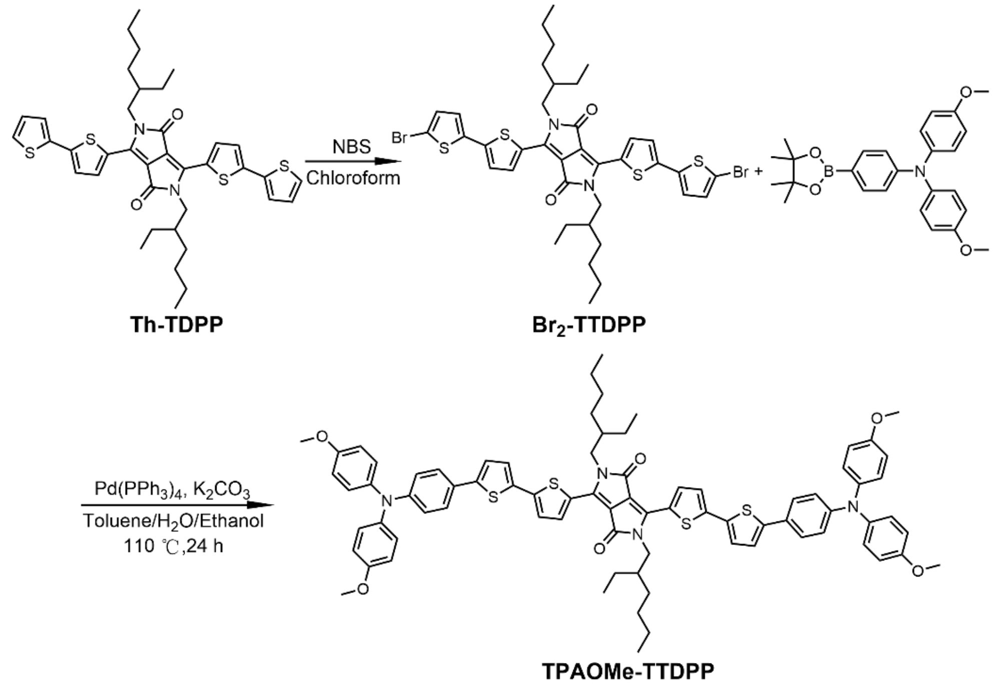

3,6-bis(5'-bromo-[2,2'-bithiophen]-5-yl)-2,5-bis(2-ethylhexyl)pyrrolo[3,4-*c*]pyrrole-1,4(2*H*,5*H*)-dione (Br$_2$-TTDPP): Th-TDPP (150 mg, 0.218 mmol) was dissolved in 30 mL of chloroform in a 50 mL flask under nitrogen. The reaction mixture was stirred and allowed to cool to 0 °C, where it was stirred for an additional 10 min. *N*-bromosuccinimide (NBS) (81 mg, 0.458 mmol) was added over a period of 50 min. After stirring for another 10 min, the reaction was warmed to room temperature and stirred overnight. The reaction mixture was poured into a separatory funnel and washed with saturated brine water (3×100 ml) three times, dried over anhydrous $MgSO_4$, and volatiles were removed *in vacuo*. The residue was purified by column chromatography on silica gel resulting in Br$_2$-TTDPP (138 mg, yield 75%). ^{1}H NMR (400 MHz, Chloroform-*d*) δ 8.88 (t, *J* = 4.8 Hz, 2H), 7.24 (d, *J* = 4.1 Hz, 2H), 7.05 (dd, *J* = 10.9, 3.9 Hz, 4H), 4.10 – 3.93 (m, 4H), 1.90 (s, 2H), 1.47 – 1.16 (m, 16H), 0.99 – 0.74 (m, 12H).

TPAOMe-TTDPP: Br$_2$-TTDPP (130 mg, 0.154 mmol) and 4-methoxy-*N*-(4-methoxyphenyl)-*N*-(4-(4,4,5,5-tetramethyl-1,3,2-dioxaborolan-2-yl)phenyl)aniline (198.6 mg, 0.61 mmol), Pd(PPh$_3$)$_4$ (7.4 mg, 0.0064 mmol) were dissolve in a mixture of toluene (20 mL), ethanol (5 mL) and 3 mL K_2CO_3 (2M) in a 50 mL two-necked round bottomed flask under an atmosphere of nitrogen. The mixture was heated to 110 °C and stirred at this temperature for 24 h under nitrogen. The reaction mixture was cooled to room temperature, extracted with 150 ml dichloromethane and washed with saturated brine water three times (3×200 ml), dried over anhydrous $MgSO_4$, and volatiles were removed *in vacuo*. The residue was purified by silica gel column chromatography and recrystallization resulting in a dark brown solid TPAOMe-TTDPP (115 mg, yield 58%). Melting point (Mp): 240 °C; ^{1}H NMR (400 MHz, Chloroform-*d*) δ 8.87 (s, 2H), 7.37 (s, 6H), 6.93 (t, *J* = 36.3 Hz, 24H), 4.03 (s, 4H), 3.82 (s, 12H), 1.94 (s, 2H), 1.44 – 1.23 (m, 17H), 0.96 – 0.81 (m, 12H). ^{13}C NMR (126 MHz, $CDCl_3$) δ 156.20, 148.79, 140.29, 126.89, 126.35, 120.07, 114.76, 55.50, 45.99, 39.27, 30.38, 28.57, 23.70, 23.14, 14.12, 10.60.

UV/Vis λ_{max} 664 nm; MOLDI-TOF-MS (m/z): calcd. for $C_{78}H_{78}N_4O_6S_4$, 1294.4804; found: 1294.4776; analysis (calcd., found for $C_{78}H_{78}N_4O_6S_4$): C(72.30, 72.45), H(6.07, 6.17), S(9.90, 9.65), N(4.32, 4.26), O(7.41, 7.48).

5,10-bis(4-hexyl-5-(naphthalen-2-yl)thiophen-2-yl)naphtho[1,2-*c*:5,6-*c*']bis([1,2,5]thiadiazole) (2N-NTT)

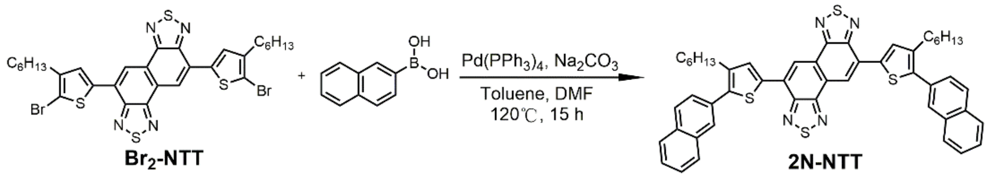

Under a nitrogen atmosphere, Br_2-NTT (300 mg, 0.41 mmol) was dissolved in a mixture of toluene and *N, N*-dimethylformamide. Naphthalen-2-ylboronic acid (351 mg, 2.04 mmol), Na_2CO_3 (1.6 ml, 2M), Pd $(PPh_3)_4$ (23.1 mg, 0.02 mmol) were added to the mixture. The resulting solution was heated to 120 °C for 15 hours. The reaction mixture was cooled to room temperature, extracted with 100 ml dichloromethane, and washed with saturated brine water three times (3×150 ml), dried over anhydrous $MgSO_4$, and volatiles were removed *in vacuo*. The residue was purified by column chromatography on silica gel using dichloromethane and petroleum ether and recrystallization resulting in a red solid 2N-NTT (200 mg, 59%). Melting point (Mp): 186 °C; ^{1}H NMR (400 MHz, Chloroform-*d*) δ 8.92 (s, 2H), 8.16 (s, 2H), 8.00 (d, *J* = 1.7 Hz, 2H), 7.93 – 7.84 (m, 6H), 7.67 (dd, *J* = 8.4, 1.8 Hz, 2H), 7.56 – 7.49 (m, 4H), 2.89 – 2.74 (m, 4H), 1.82 – 1.71 (m, 4H), 1.46 – 1.25 (m, 12H), 0.92 – 0.81 (m, 6H). ^{13}C NMR (126 MHz, $CDCl_3$) δ 153.44, 152.37, 140.49, 140.28, 137.13, 133.39, 132.63, 131.87, 131.14, 128.17, 128.05, 127.72, 127.26, 126.48, 126.28, 124.65, 121.82, 31.67, 31.06, 29.35, 29.14, 22.68, 14.10. UV/Vis λ_{max} 342 nm; MOLDI-TOF-MS (m/z): calcd. for $C_{50}H_{44}N_4S_4$, 828.2449; found: 828.2546; analysis (calcd., found for $C_{50}H_{44}N_4S_4$): C(72.43, 72.67), H(5.35, 5.29), S(15.47, 15.28), N(6.76, 6.67).

5,10-bis(5-(9,9-dimethyl-9*H*-fluoren-2-yl)-4-hexylthiophen-2-yl)naphtho[1,2-*c*:5,6-*c'*]bis([1,2,5]thiadiazole) (Flu-NTT)

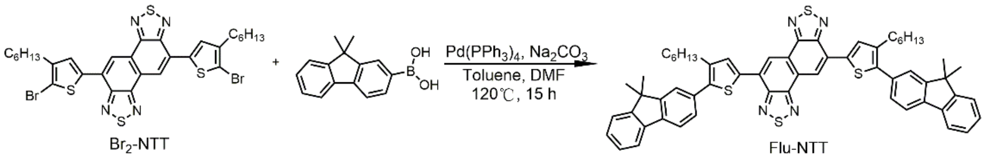

Flu-NTT was prepared from Br_2-NTT (200 mg, 0.27 mmol) and (9,9-dimethyl-9*H*-fluoren-2-yl)boronic acid (324 mg, 1.36 mmol) in a procedure similar to that for 2N-NTT resulting in a purple solid (184 mg, 71%). Melting point (Mp): 217 °C; ^{1}H NMR (400 MHz, Chloroform-*d*) δ 9.00 (s, 2H), 8.20 (s, 2H), 7.81 – 7.75 (m, 4H), 7.64 (d, *J* = 1.6 Hz, 2H), 7.55 (dd, *J* = 7.8, 1.7 Hz, 2H), 7.50 – 7.46 (m, 2H), 7.37 (ddd, *J* = 7.3, 5.1, 1.5 Hz, 4H), 2.83 (dd, *J* = 9.2, 6.7 Hz, 4H), 1.79 (d, *J* = 7.6 Hz, 4H), 1.33 (tt, *J* = 5.9, 2.6 Hz, 8H), 0.92 – 0.85 (m, 6H). ^{13}C NMR (126 MHz, $CDCl_3$) δ 154.05, 153.91, 153.59, 152.52, 141.21, 139.98, 138.85, 138.77, 136.72, 133.30, 131.32, 128.18, 127.49, 127.12, 126.46, 124.76, 123.49, 122.69, 121.96, 120.18, 120.09, 47.01, 31.71, 31.16, 29.39, 29.22, 27.22, 22.70, 14.12. UV/Vis λ_{max} 345 nm; MOLDI-

TOF-MS (m/z): calcd. for $C_{60}H_{56}N_4S_4$, 960.3388; found: 960.3397; analysis (calcd., found for $C_{60}H_{56}N_4S_4$): C(74.96, 75.01), H(5.87, 5.92), S(13.34, 13.12), N(5.83, 5.81).

4,4'-(naphtho[1,2-*c*:5,6-*c'*]bis([1,2,5]thiadiazole)-5,10-diylbis(3-hexylthiophene-5,2-diyl))bis(*N,N*-bis(4-methoxyphenyl)aniline) (TPAOMe-NTT)

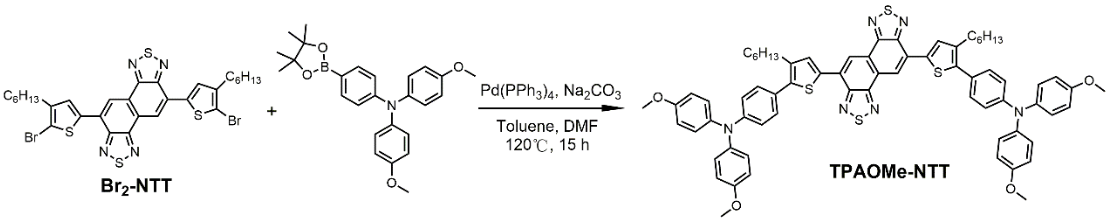

TPAOMe-NTT was prepared from Br_2-NTT (300 mg, 0.41 mmol) and 4-methoxy-*N*-(4-methoxyphenyl)-*N*-(4-(4,4,5,5-tetramethyl-1,3,2-dioxaborolan-2-yl)phenyl)aniline (881mg, 2.04 mmol) in a procedure similar to that for 2N-NTT resulting in a dark-blue solid (150 mg, 31%). Melting point (M_p): 216 °C; ^{1}H NMR (400 MHz, Chloroform-*d*) δ 8.82 (s, 2H), 8.08 (s, 2H), 7.36 – 7.32 (m, 4H), 7.16 – 7.08 (m, 8H), 6.98 (d, *J* = 8.1 Hz, 4H), 6.91 – 6.84 (m, 8H), 3.82 (s, 12H), 2.74 (s, 4H), 1.80 – 1.69 (m, 4H), 1.47 – 1.19 (m, 12H), 0.95 – 0.84 (m, 6H). UV/Vis λ_{max} 360 nm; analysis (calcd., found for $C_{70}H_{66}N_6O_4S_4$): C(71.04, 71.35), H(5.62, 5.55), S(10.83, 10.76), N(7.10, 7.01), O(5.41, 5.34).

5,10-bis(4,4-didodecyl-4*H*-cyclopenta[2,1-*b*:3,4-*b'*]dithiophen-2-yl)naphtho[1,2-*c*:5,6-*c'*]bis([1,2,5]thiadiazole) (NTC)

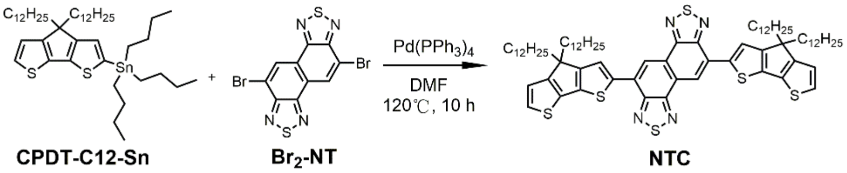

Under a nitrogen atmosphere, Br_2-NT (250 mg, 0.62 mmol), CPDT-C12-Sn (1.5 g, 1.86 mmol) and $Pd(PPh_3)_4$ (190 mg, 0.16 mmol) were dissolved in 60 ml DMF. Then the solution was heated to 120 °C and stirred for 10 hours. The reaction mixture was cooled to room temperature, extracted with 100 ml dichloromethane and washed with saturated brine water three times (3×150 ml), dried over anhydrous $MgSO_4$, and volatiles were removed *in vacuo*. The residue was purified by column chromatography on silica gel using dichloromethane and petroleum ether and recrystallization resulting in a dark-blue solid NTC (310mg, 39%). Melting point (Mp): 128 °C; ^{1}H NMR (400 MHz, Chloroform-*d*) δ 8.97 (s, 2H), 8.19 (s, 2H), 7.28 (d, *J* = 4.8 Hz, 2H), 7.01 (d, *J* = 4.8 Hz, 2H), 1.98 (ddd, *J* = 9.4, 5.7, 2.5 Hz, 8H), 1.17 (s, 80H), 0.83 (t, *J* = 7.0 Hz, 12H). ^{13}C NMR (126 MHz, $CDCl_3$) δ 159.18, 158.98, 153.54, 152.35, 139.90, 138.98, 136.62, 132.15, 128.56, 126.98, 126.20, 124.37, 122.57, 121.77, 120.73, 53.92, 37.96, 31.89, 30.08, 29.64, 29.63, 29.62, 29.42, 29.32, 24.64, 22.66, 14.10. UV/Vis λ_{max} 603 nm; MOLDI-TOF-MS (m/z): calcd. for $C_{76}H_{108}N_4S_6$, 1268.6898; found: 1268.6955; analysis (calcd., found for $C_{76}H_{108}N_4S_6$): C(71.87, 72.15), H(8.57, 8.41), S(15.15, 15.01), N(4.41, 4.67).

5,10-bis(4,4-didodecyl-6-(naphthalen-2-yl)-4*H*-cyclopenta[2,1-*b*:3,4-*b'*]dithiophen-2-yl)naphtho[1,2-*c*:5,6-*c'*]bis([1,2,5]thiadiazole) (2N-NTC)

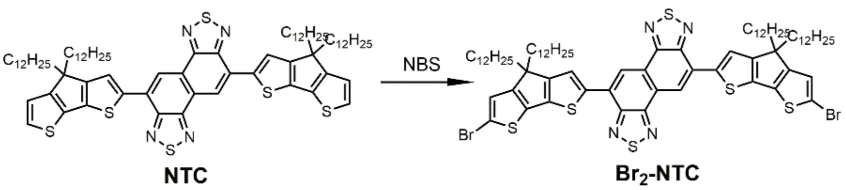

5,10-bis(6-bromo-4,4-didodecyl-4*H*-cyclopenta[2,1-*b*:3,4-*b'*]dithiophen-2-yl)naphtho[1,2-*c*:5,6-*c'*]bis([1,2,5]thiadiazole) (Br_2-NTC): NTC (300mg, 0.24 mmol) was dissolved in 50 ml chloroform and cooled to -30 °C. NBS (107 mg, 0.60 mmol) was added to the solution in several times in dark. The solution was stirred in dark for 2 hours. After finishing the reaction, water was added to the solution and the aqueous layer extracted with dichloromethane. The organic layer was washed with brine, dried over anhydrous $MgSO_4$ and then concentrated under reduced pressure. The reaction mixture was warmed to room temperature and washed with saturated brine water three times (3×150 ml), dried over anhydrous $MgSO_4$, and volatiles were removed *in vacuo*. The residue was purified by column chromatography on silica gel using dichloromethane and petroleum ether resulting in a dark-blue solid Br_2-NTC (330 mg, 98%). ^{1}H NMR (400 MHz, Chloroform-*d*) δ 8.95 (s, 2H), 8.15 (s, 2H), 7.02 (s, 2H), 1.95 (td, J = 9.2, 6.1 Hz, 8H), 1.36 – 1.13 (m, 80H), 0.83 (t, J = 7.0 Hz, 12H).

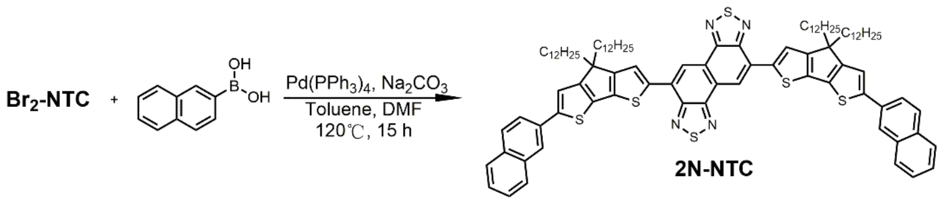

2N-NTC was prepared from Br_2-NTC (150mg, 0.11 mmol) and naphthalen-2-ylboronic acid (90.3 mg, 0.53 mmol) in a procedure similar to that for 2N-NTT resulting in a dark-blue solid (120 mg, 75%). Melting point (Mp): 174 °C;^{1}H NMR (400 MHz, dichloromethane-*d2*) δ 8.83 (d, J = 56.7 Hz, 2H), 8.26 (d, J = 15.2 Hz, 2H), 7.86 – 7.61 (m, 8H), 7.59 – 7.32 (m, 6H), 2.13 (d, J = 9.4 Hz, 8H), 1.38 – 1.11 (m, 80H), 0.82 (t, J = 6.9 Hz, 12H). ^{13}C NMR (126 MHz, $CDCl_3$) δ 37.03, 30.85, 29.12, 28.64, 28.62, 28.60, 28.44, 28.29, 23.71, 21.63, 13.06. UV/Vis λ_{max} 639 nm; MOLDI-TOF-MS (m/z): calcd. for $C_{96}H_{120}N_4S_6$, 1521.7871; found: 1521.7827; analysis (calcd., found for $C_{96}H_{120}N_4S_6$): C(75.74, 76.11), H(7.95, 7.87), S(12.64, 12.26), N(3.68, 3.70).

5,10-bis(6-(9,9-dimethyl-9*H*-fluoren-2-yl)-4,4-didodecyl-4*H*-cyclopenta[2,1-*b*:3,4-*b'*]dithiophen-2-yl)naphtho[1,2-*c*:5,6-*c'*]bis([1,2,5]thiadiazole) (Flu-NTC)

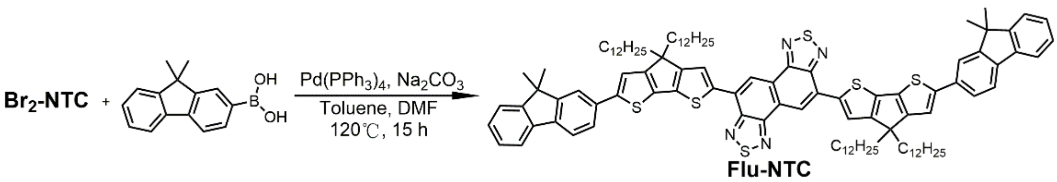

Flu-NTC was prepared from Br_2-NTC (150 mg, 0.11 mmol) and (9,9-dimethyl-9*H*-fluoren-2-yl)boronic acid (125 mg, 0.55 mmol) in a procedure similar to that for 2N-NTT resulting in a dark-green solid (85.9 mg, 59.3%). ^{1}H NMR (400 MHz, Chloroform-*d*) δ 8.93 (s, 2H), 8.20 (s, 2H), 7.73 – 7.66 (m, 6H), 7.63 (dd, J = 7.8, 1.6 Hz, 2H), 7.47 – 7.43 (m, 2H), 7.37 – 7.30 (m, 6H), 2.05 (q, J = 6.5 Hz, 9H), 1.57 (s, 12H), 1.27 – 1.10 (m, 80H), 0.81 (t, J = 6.9 Hz, 12H).

^{13}C NMR (126 MHz, $CDCl_3$) δ 153.80, 152.30, 127.35, 127.09, 124.27, 122.60, 120.02, 46.96, 38.15, 31.87, 30.18, 29.70, 29.66, 29.64, 29.62, 29.48, 29.32, 27.24, 24.71, 22.65, 14.08. UV/Vis λ_{max} 431 nm; MOLDI-TOF-MS (m/z): calcd. for $C_{106}H_{132}N_4S_6$, 1653.8810; found: 1653.8831; analysis (calcd., found for $C_{106}H_{132}N_4S_6$): C(76.95, 77.10), H(8.04, 8.08), S(11.63, 11.29), N(3.39, 3.47).

4,4'-(benzo[1,2-*c*:4,5-*c*]bis([1,2,5]thiadiazole)-4,7-diylbis(3-(2-ethylhexyl)thiophene-5,2-diyl))bis(*N*,*N*-bis(4-methoxyphenyl)ani-line) (TPAOMe-BBTT)

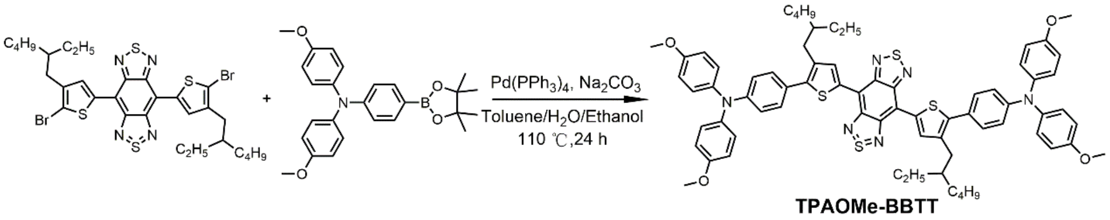

TPAOMe-BBTT was prepared from 4,7-dibromobenzo[1,2-*c*:4,5-*c*]bis([1,2,5]thiadiazole (120 mg, 0.16 mmol) and 4-methoxy-*N*-(4-methoxyphenyl)-*N*-(4-(4,4,5,5-tetramethyl-1,3,2-dioxaborolan-2-yl)phenyl)aniline (209.6 mg, 0.49 mmol). The synthesis procedures are similar to that of An-TDPP resulting in a green dark solid product (59.9 mg, 31.5%). ^1H NMR (400 MHz, Chloroform-*d*) δ 8.83 (s, 2H), 7.40 (dd, *J* = 8.5, 3.4 Hz, 4H), 7.12 (dt, *J* = 9.8, 2.2 Hz, 8H), 6.98 (d, *J* = 8.1 Hz, 4H), 6.86 (dt, *J* = 9.5, 2.2 Hz, 8H), 3.81 (t, *J* = 2.2 Hz, 12H), 2.77 (d, *J* = 7.0 Hz, 4H), 1.40 – 1.17 (m, 26H), 0.85 (q, *J* = 7.3, 6.8 Hz, 12H). MOLDI-TOF-MS (m/z): calcd. for $C_{70}H_{72}N_6O_4S_4$, 1188.4498; found: 1188.4316.

2. Results

2.1. HPLC, UV-vis-NIR absorption, PL emission and CV spectra

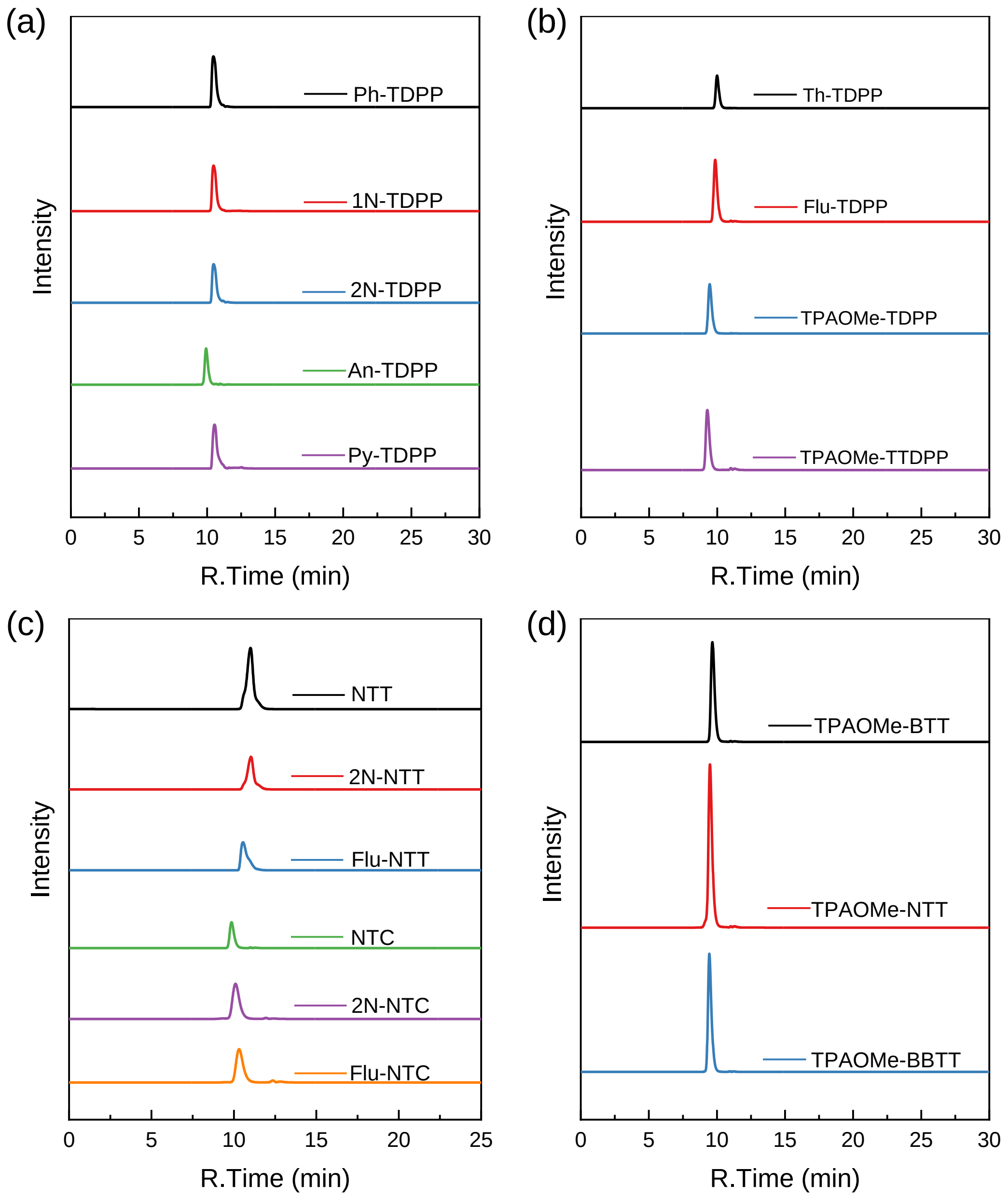

Supplementary Figure 3. HPLC analysis for (a, b) DPP, (c) NT, (d) TPA-based molecules with Cosmosil 5C_{18}-MS-II column.

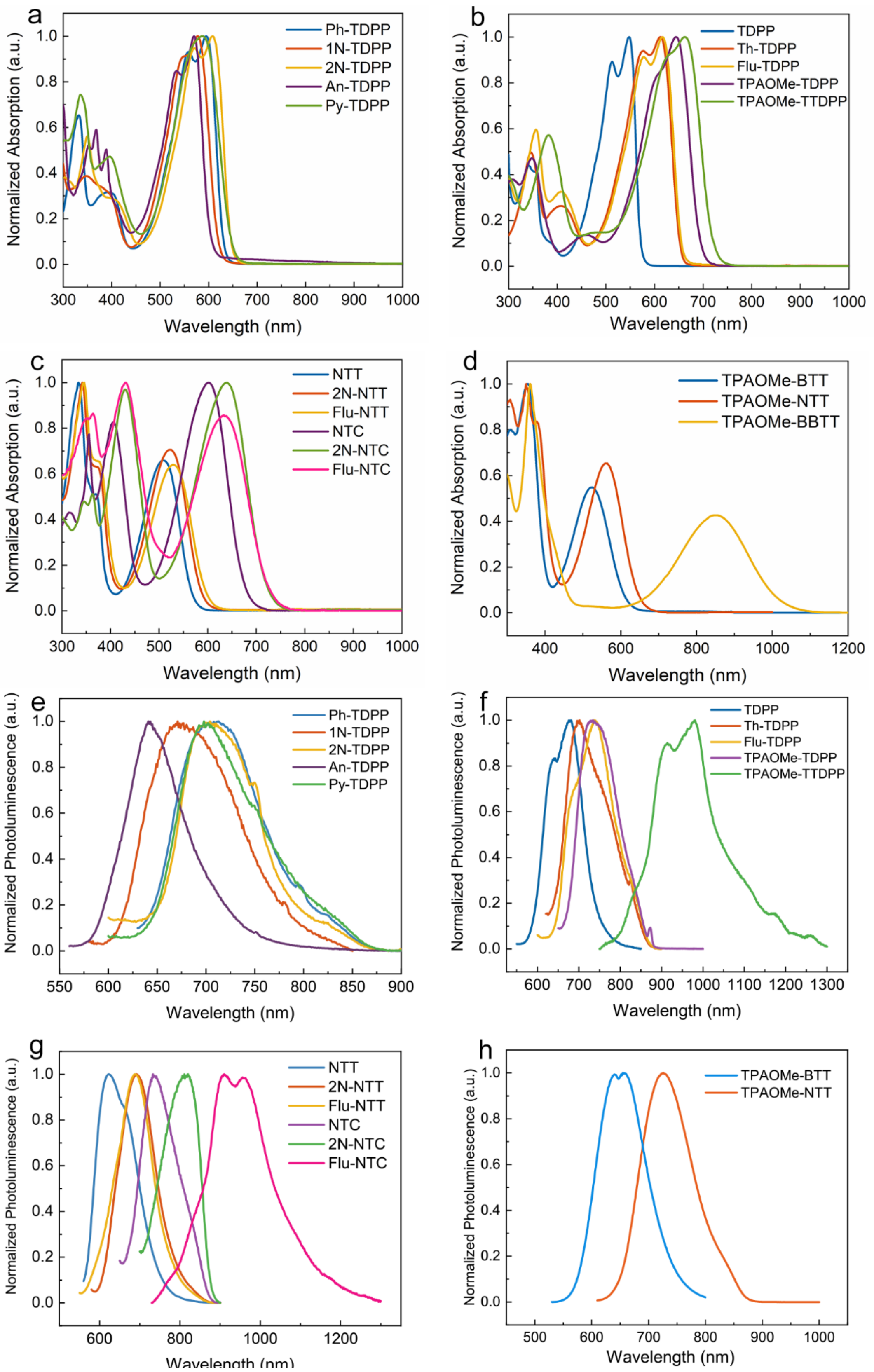

Supplementary Figure 4. The UV-vis-NIR absorption spectra of (a) DPP-based small molecules with fused-phenyl groups, (b) TDPP, Th-TDPP, Flu-TDPP, TPAOMe-TDPP and TPAOMe-TTDPP, (c) NT-based small molecules, and (d) BT-based small molecules in solution. The photoluminescence spectra of (e) DPP-based small molecules with fused-phenyl groups, (f) TDPP, Th-TDPP, Flu-TDPP, TPAOMe-TDPP and TPAOMe-TTDPP, (g) NT-based small molecules, and (h) TPAOMe-based small molecules in film.

Supplementary Table 1. Optical properties of the materials.

Materials	λ^{abs}_{max}[a] [nm]	λ^{abs}_{max}[b] [nm]	λ^{pl}_{max}[c] [nm]	λ_{inter}[d] [nm]	E_g^{opt}[e] [eV]	HOMO[f] [eV]	LUMO[g] [eV]
Ph-TDPP	595	633	712	662	1.87	-5.38	-3.51
1N-TDPP	577	600	671	634	1.96	-5.44	-3.48
2N-TDPP	608	650	704	676	1.84	-5.23	-3.39
An-TDPP	570	582	642	614	2.02	-5.31	-3.29
Py-TDPP	587	630	698	668	1.86	-5.14	-3.28
TDPP	548	565	679	592	2.09	-5.36	-3.27
Th-TDPP	612	639	700	666	1.86	-5.14	-3.28
Flu-TDPP	617	639	740	670	1.85	-5.25	-3.40
TPAOMe-TDPP	644	660	730	694	1.79	-4.94	-3.15
TPAOMe-TTDPP	663	693	978	784	1.58	-4.94	-3.36
NTT	508	543	623	603	2.06	-5.80	-3.74
2N-NTT	524	562	692	626	1.98	-5.50	-3.52
Flu-NTT	530	554	690	610	2.03	-5.52	-3.49
NTC	602	623	735	685	1.81	-5.26	-3.45
2N-NTC	640	652	817	738	1.68	-5.28	-3.60
Flu-NTC	633	672	909	778	1.59	-5.35	-3.76
TPAOMe-BTT	523	541	655	591	2.10	-5.07	-2.97
TPAOMe-NTT	560	574	725	646	1.92	-4.98	-3.06
TPAOMe-BBTT	854	879	/	1133[h]	1.09	-4.67	-3.58

[a]Wavelength of maximum absorption in chloroform solutions, [b]wavelength of maximum absorption in thin films, [c]wavelength of maximum emission in thin films, [d]calculated from the intersection of absorption and emission curves of pristine thin films, [e]E_g^{opt}=1240/λ_{inter}. [f]E_{HOMO}=-e(E_{ox} + 4.67). [g]E_{LUMO}= E_{HOMO} + E_g. [h]The absorption wavelength of TPAOMe-BBTT was determined as the onset of the absorption curve because of the weak fluorescence.

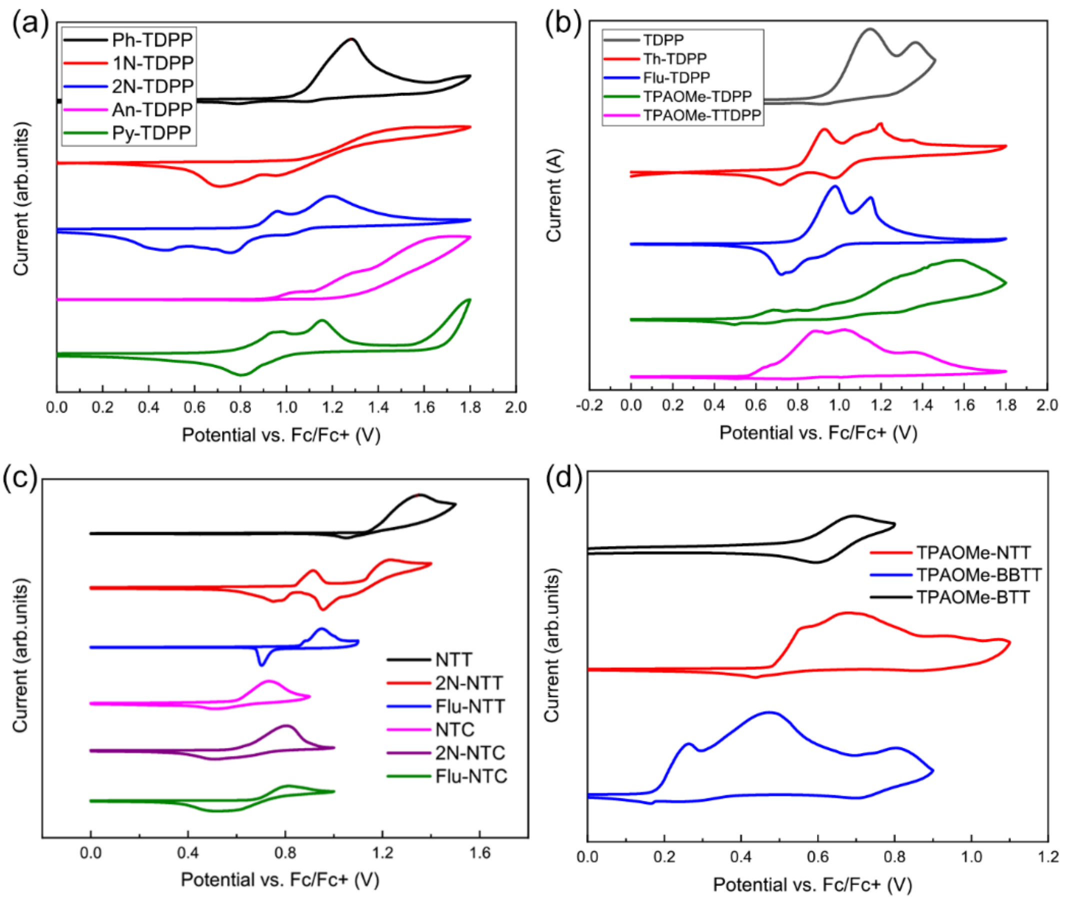

Supplementary Figure 5. Cyclic voltammetry curves of (a, b) DPP, (c) NT, (d) TPA-based molecules in thin films using *n*-Bu_4NPF_6 as electrolytes.

2.2. Theoretical calculations

The diradical index of organic materials is sensitive to computational methods utilized.[20] We have analyzed the open-shell character of the materials with several methods to confirm their diradical and polyradical characters. Using the spin-projected unrestricted Hartree-Fock (PUHF) method we find that all the materials possess variable open-shell character (Supplementary Table 2), which correlates with their bandgap. As the PUHF method prone to a large spin contamination, we have tested the widely accepted broken symmetry[21] (BS) approach with different density functionals. However, unlike the other open-shell small materials[22-24] or polymers[25-26], the conventional BS approach did not predict open-shell character in the current set of molecules, except for the TPAOMe-BBTT with very small diradical character (y_0 = 0.03, using Yamaguchi formula). For example, Rudebusch *et al*. predicted diradical character (y_0 = 0.088 to 0.273) for benzothiophene-based acenes using tuned LC-RBLYP CASCI(2,2) (Complete Active Space Configuration Interaction).[22] With the same approach, 2N-NTC and TPAOMe-TTDPP molecules provide a negligible diradical character (y_0 = 0.003 and 0.008, respectively) indicating diradical character is not as pronounced in the current set of molecules than the ones reported by Haley and coworkers.[22] We believe this to be due to the presence of numerous heteroatoms in the current set of molecules.

Another simple but elegant method to estimate the open-shell character is proposed by Grimme *et al*., which is the fractional occupation number weighted electron density (N_{FOD}).[27] Fractional

orbital density (FOD) is an extremely simple and cost-effective method based on smearing the electrons over the molecular orbitals using finite temperature DFT (FT-DFT). N_{FOD} accurately quantifies the static electron correlation and molecules with a delocalized FOD and a large N_{FOD} have multireference character. Supplementary Table 3 shows the N_{FOD} values of the molecules studied in this work. A large N_{FOD} values reveal that the electrons are strongly correlated, and different materials provide different N_{FOD} values. Interestingly, the trends are qualitatively consistent with diradical index computed from PUHF (see Supplementary Table 2), showing good linear correlation between N_{FOD} and y_0.

To better understand the radical nature for these molecules, we explore their open-shell characters (y_i) using fractional orbital occupancy. Supplementary Table 4 shows the y_0, y_1, y_2 and y_3 values for all the molecules computed using the FT-DFT at B3LYP/6-31G(d,p) level of theory and basis set. Our result reveals polyradical character in all the molecules.

The spatial distribution of unpaired electrons in these molecules are evaluated using FOD plots (see Fig. S14-S16). FOD plots show partially delocalized/localized electron density distribution along the molecular backbones, disclosing strongly correlated electrons.

We have also computed the vertical singlet-triplet energy gap (ΔE_{ST}) using FT-DFT with B3LYP/6-31G(d,p) and the results are presented in Supplementary Table 3. It was shown that the ΔE_{ST} gap computed using FT-DFT is comparable to that of CASPT2 (complete active space perturbation theory) method.[28] The computed ΔE_{ST} gap for 2N-NTC (11.03 kcal/mol) and TPAOMe-TTDPP (10.65 kcal/mol) is overestimated compared to the experimental gap, 4.76 and 5.52 kcal/mol, respectively. We believe this is due to the medium effects that we are not able to capture in the isolated molecule calculations. However, we find a good correlation between y_0 (and N_{FOD}) and ΔE_{ST} gap using FT-DFT method, a larger N_{FOD} value indicates a smaller ΔE_{ST} gap.

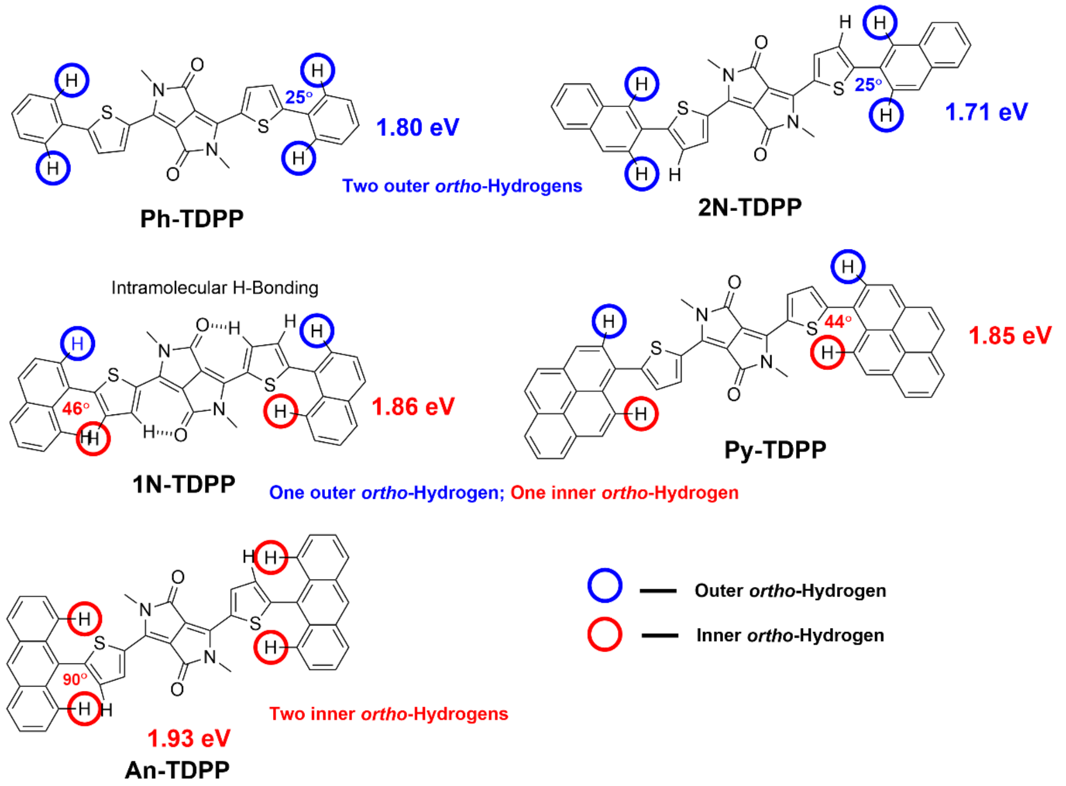

Supplementary Figure 6. Intramolecular twist angles calculated at RB3LYP/6-31G(d,p) level of theory and basis set of DPP molecules indicated by the outer and inner ortho-hydrogens (blue and red circles,

respectively).

Supplementary Table 2. Calculated electronic and optical properties of the molecules.

Materials	PUHF[a]		FT-DFT[b]		λ[c]	Transition[d]	Contribution[e]	HOMO[f]	LUMO[g]	E_g[h]
	y_0	y_1	y_0	y_1	(nm)		(%)	(eV)	(eV)	(eV)
Ph-TDPP	0.295	0.048	0.481	0.118	556.85	H → L	97.5	-4.81	-2.58	2.23
1N-TDPP	0.283	0.072	0.476	0.136	546.88	H → L	96.8	-4.82	-2.55	2.27
2N-TDPP	0.313	0.078	0.581	0.163	567.68	H → L	96.6	-4.78	-2.60	2.18
An-TDPP	0.237	0.156	0.443	0.218	514.57	H → L	97.7	-4.91	-2.48	2.43
Py-TDPP	0.310	0.142	0.495	0.205	563.49	H → L	92.6	-4.78	-2.59	2.19
TDPP	0.223	0.019	0.431	0.070	502.31	H → L	99.0	-4.97	-2.52	2.45
Th-TDPP	0.335	0.067	0.519	0.150	589.68	H → L	96.7	-4.78	-2.68	2.10
Flu-TDPP	0.317	0.073	0.511	0.155	576.86	H → L	95.9	-4.71	-2.56	2.15
TPAOMe-TDPP	0.309	0.056	0.538	0.148	593.29	H → L	89.1	-4.35	-2.29	2.06
TPAOMe-TTDPP	0.379	0.112	0.539	0.182	639.63	H → L	84.5	-4.40	-2.50	1.90
NTT	0.269	0.054	0.391	0.116	500.04	H → L	96.9	-5.36	-2.88	2.48
2N-NTT	0.306	0.084	0.434	0.132	539.84	H → L	93.0	-5.12	-2.87	2.25
Flu-NTT	0.306	0.080	0.443	0.136	546.80	H → L	91.3	-5.04	-2.83	2.21
NTC	0.357	0.105	0.466	0.145	616.24	H → L	94.3	-4.80	-2.83	1.97
2N-NTC	0.396	0.160	0.493	0.189	651.90	H → L	91.1	-4.70	-2.86	1.84
Flu-NTC	0.396	0.159	0.500	0.194	658.24	H → L	90.2	-4.64	-2.83	1.81
TPAOMe-BTT	0.264	0.047	0.445	0.102	536.80	H → L	75.7	-4.47	-2.37	2.10
TPAOMe-NTT	0.304	0.071	0.480	0.145	570.34	H → L	71.3	-4.55	-2.68	1.87
TPAOMe-BBTT	0.665	0.047	0.827	0.094	729.64	H → L	43.4	-4.34	-3.04	1.30

[a]Diradical character index (y_0) and tetraradical character index (y_1) calculated with PUHF/6-31G(d,p). [b]Diradical character index (y_0) and tetraradical character index (y_1) calculated with FT-DFT/B3LYP/6-31G(d,p). [c]Wavelength of the excitation from the ground to the first excited state. [d]Orbitals involved in the transition. [e]Contribution of individual orbitals in the transition. [f]The highest occupied molecular orbital (HOMO) and the [g]lowest unoccupied molecular orbital (LUMO) energies calculated at (U)B3LYP/6-31G(d,p) level theory and basis set. [h]The calculated energy gap (E_g) between the HOMO and LUMO. The excited state calculations are performed on the ground state geometry with PCM(chloroform)/TDDFT/BHandHLYP/6-31G(d,p) level of theory and basis set. All energies are in eV, λ is in nm and y_0 and y_1 are dimensionless quantity. H = HOMO, L = LUMO.

Supplementary Table 3. $\boldsymbol{N_{FOD}}$ **and vertical singlet-triplet energy gap for the molecules computed using FT-DFT at B3LYP/6-31G(d,p) level.**

Materials	N_{FOD}	Vertical ΔE_{ST} gap (kcal/mol)
TDPP	1.104	23.02
1N-TDPP	1.801	15.47
2N-TDPP	2.081	13.64
An-TDPP	2.261	12.21
Th-TDPP	1.653	16.82
Flu-TDPP	1.946	14.75
Ph-TDPP	1.517	18.13
Py-TDPP	2.362	12.14
TPAOMe-TDPP	2.470	11.92
TPAOMe-TTDPP	2.816	10.65
NTT	1.311	20.72
2N-NTT	2.016	14.40
Flu-NTT	2.093	14.07
NTC	1.909	14.95
2N-NTC	2.689	11.03
Flu-NTC	2.769	10.84
TPAOMe-BTT	2.277	13.08
TPAOMe-NTT	2.636	11.40
TPAOMe-BBTT	3.017	10.06

Supplementary Table 4. Computed radical indices (y_i) using FT-DFT at B3LYP/6-31G(d,p) level.

Materials	Fractional Orbital Occupancy			
	y_0	y_1	y_2	y_3
TDPP	0.431	0.070	0.018	0.009
1N-TDPP	0.476	0.136	0.095	0.055
2N-TDPP	0.581	0.163	0.094	0.070
An-TDPP	0.443	0.218	0.218	0.074
Th-TDPP	0.519	0.150	0.063	0.026
Flu-TDPP	0.511	0.155	0.084	0.043
Ph-TDPP	0.481	0.118	0.047	0.026
Py-TDPP	0.495	0.205	0.161	0.072
TPAOMe-TDPP	0.538	0.148	0.069	0.069
TPAOMe-TTDPP	0.539	0.182	0.108	0.069
NTT	0.391	0.116	0.066	0.028
2N-NTT	0.434	0.133	0.112	0.095
Flu-NTT	0.443	0.136	0.115	0.094
NTC	0.466	0.145	0.121	0.088
2N-NTC	0.493	0.189	0.170	0.121
Flu-NTC	0.500	0.194	0.174	0.123
TPAOMe-BTT	0.445	0.102	0.089	0.068
TPAOMe-NTT	0.480	0.145	0.112	0.083
TPAOMe-BBTT	0.827	0.094	0.075	0.065

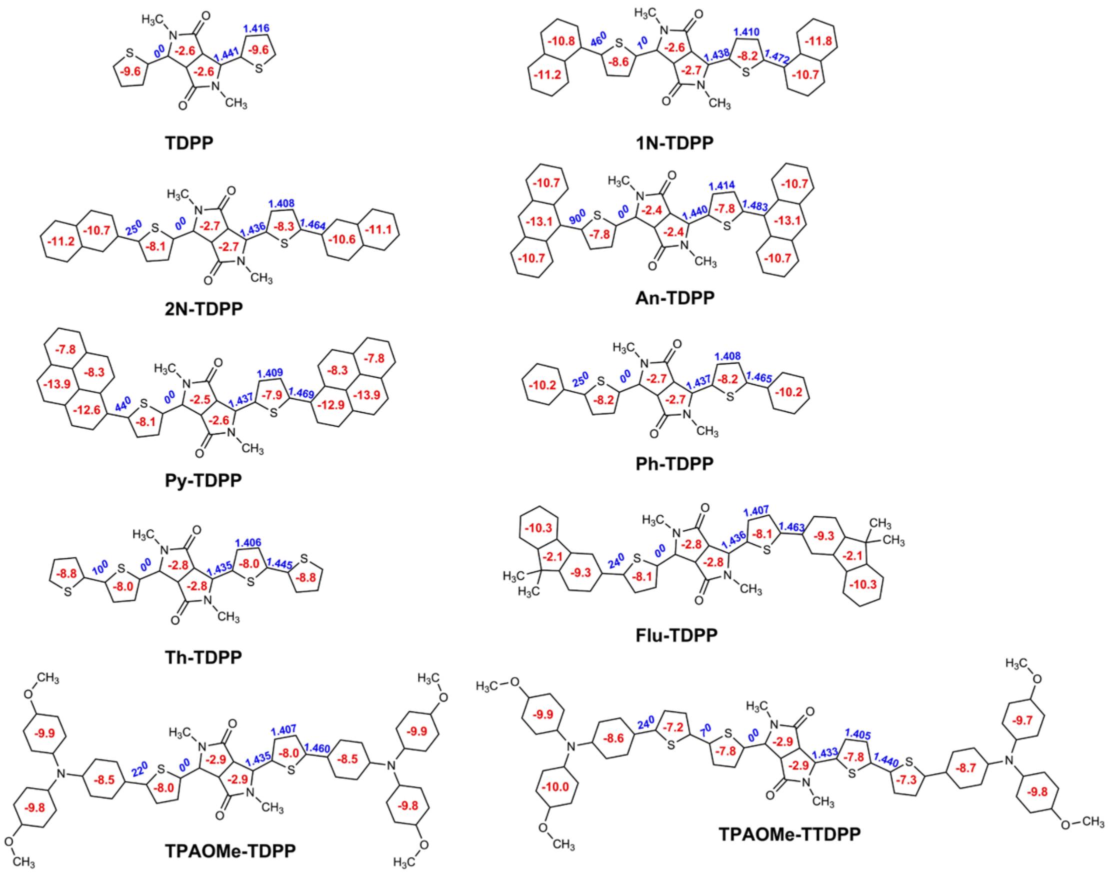

Supplementary Figure 7. Optimized geometric parameters and NICS$_{iso}$(1) (ppm) values of the DPP-based molecules calculated at RB3LYP/6-31G*(*d,p*)* level of theory and basis set. Bond lengths are provided in Å.

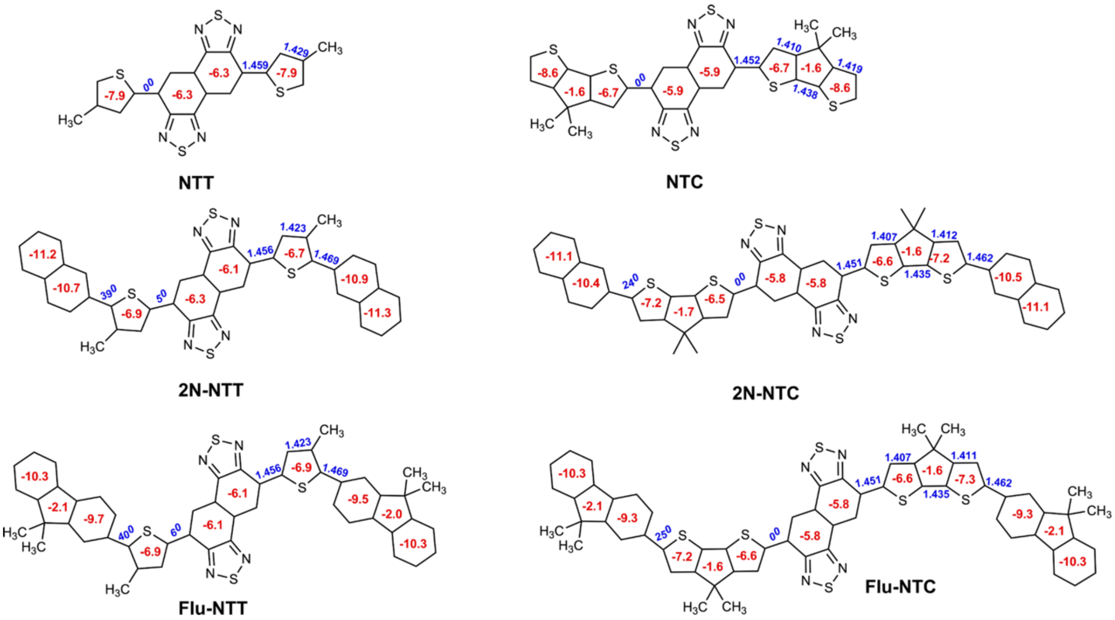

Supplementary Figure 8. Optimized geometric parameters and NICS$_{iso}$(1) (ppm) values of the NT-based molecules calculated at RB3LYP/6-31G*(*d,p*)* level of theory and basis set. Bond lengths are provided in Å.

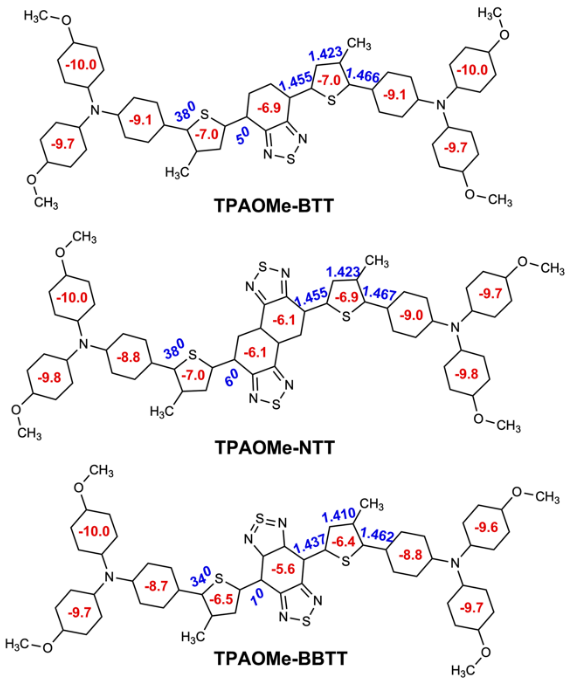

Supplementary Figure 9. Optimized geometric parameters and NICS$_{iso}$(1) (ppm) values of the BT, NT, and BBT analogues calculated at (U)B3LYP/6-31G*(d*,p*)* level of theory and basis set. Bond lengths are provided in Å.

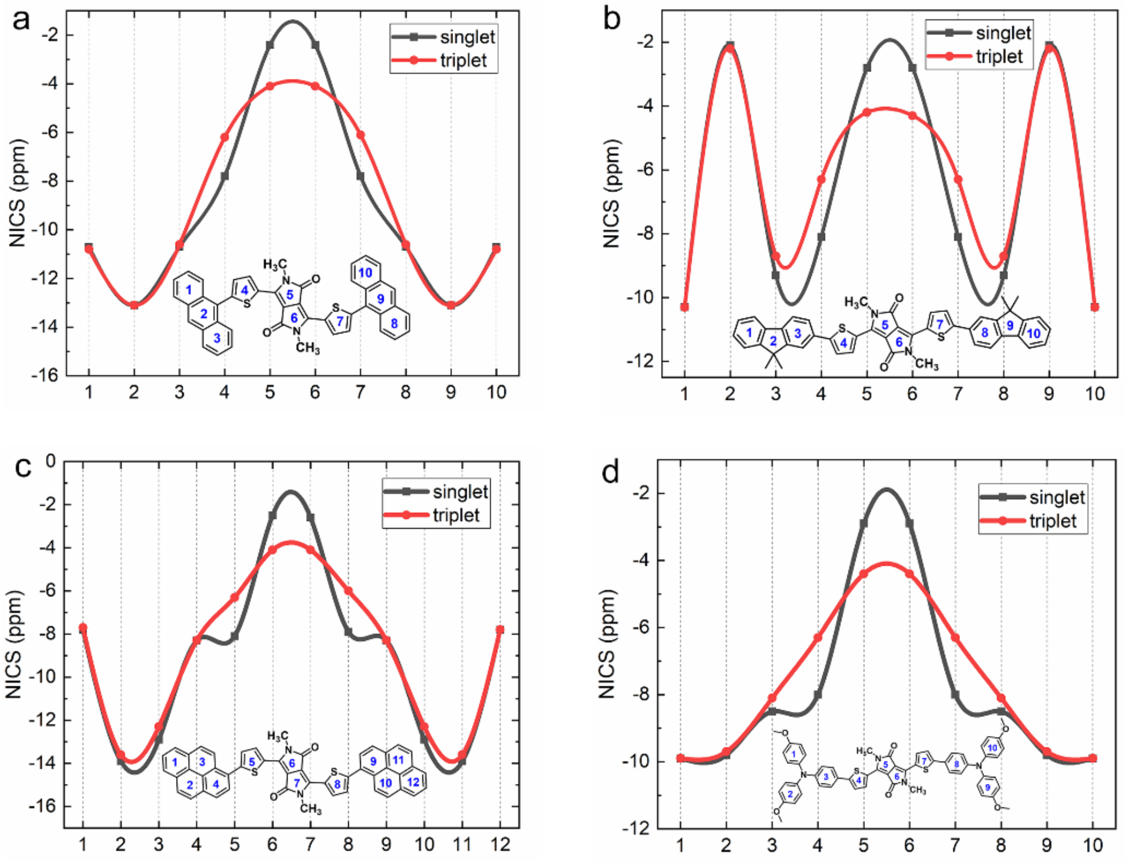

Supplementary Figure 10. The numerical trend of NICS$_{iso}$(1) values (singlet and triplet) of (a) An-TDPP, (b) Flu-TDPP, (c) Py-TDPP, (d) TPAOMe-TDPP calculated at RB3LYP/6-31G*(d,p)* level of theory and basis set. All of these molecules show an increase of aromaticity from singlet to triplet state.

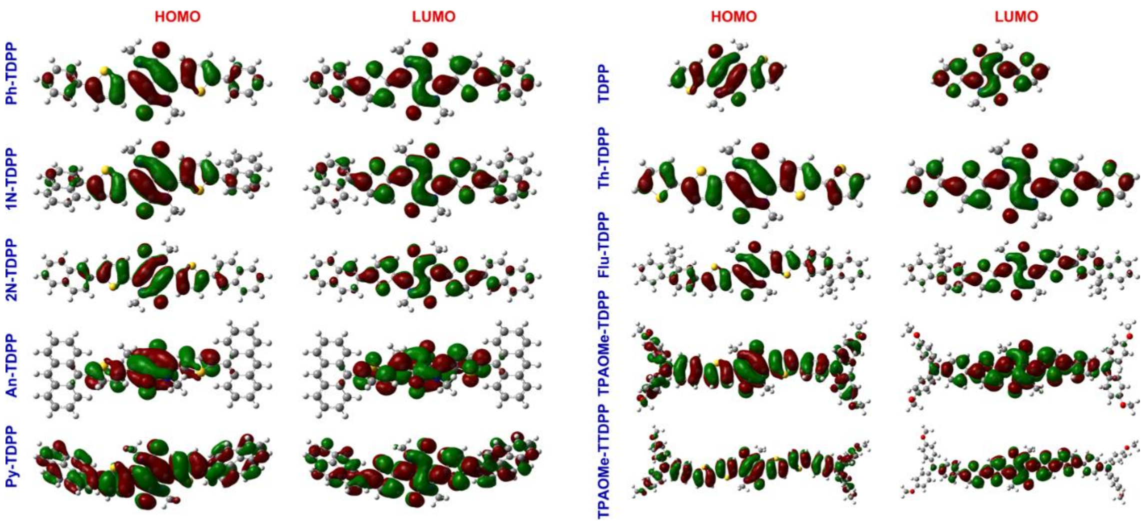

Supplementary Figure 11. The optimized ground-state geometry and molecular orbital (MO) diagrams of the DPP-based molecules calculated at RB3LYP/6-31G*(*d,p*)* level of theory and basis set. The green and red surfaces are drawn at isovalue = |0.02| au, which represents the positive and negative contributions on the wavefunctions, respectively. Color code for the atoms: grey for C, blue for N, red for O, and yellow for S.

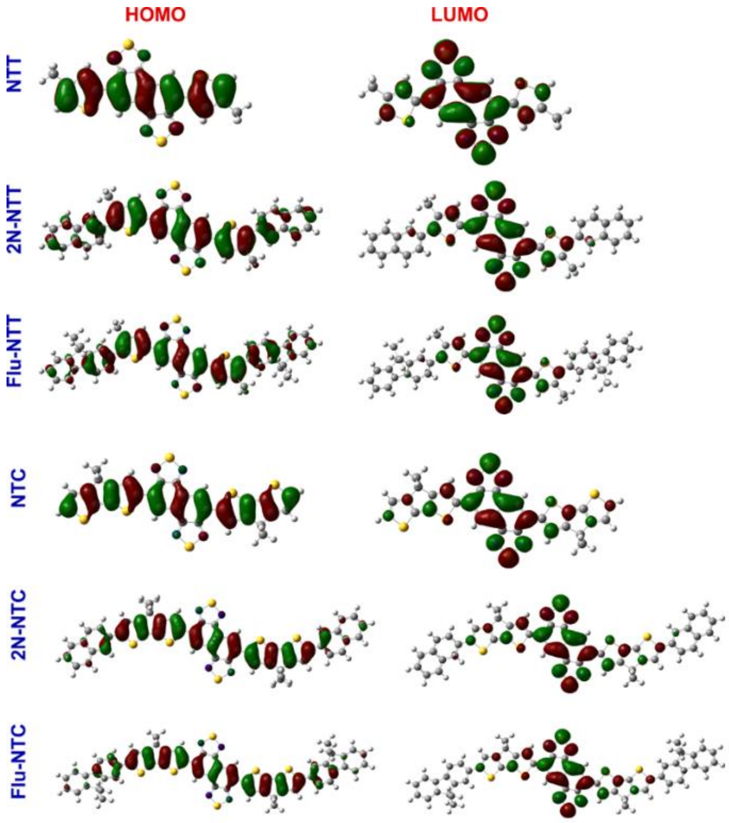

Supplementary Figure 12. The optimized ground-state geometry and molecular orbital (MO) diagrams of the NT-based molecules calculated at RB3LYP/6-31G*(*d,p*)* level of theory and basis set. The green and red surfaces are drawn at isovalue = |0.02| au, which represents the positive and negative contributions on the wavefunctions, respectively. Color code for the atoms: grey for C, blue for N, red for O, and yellow for S.

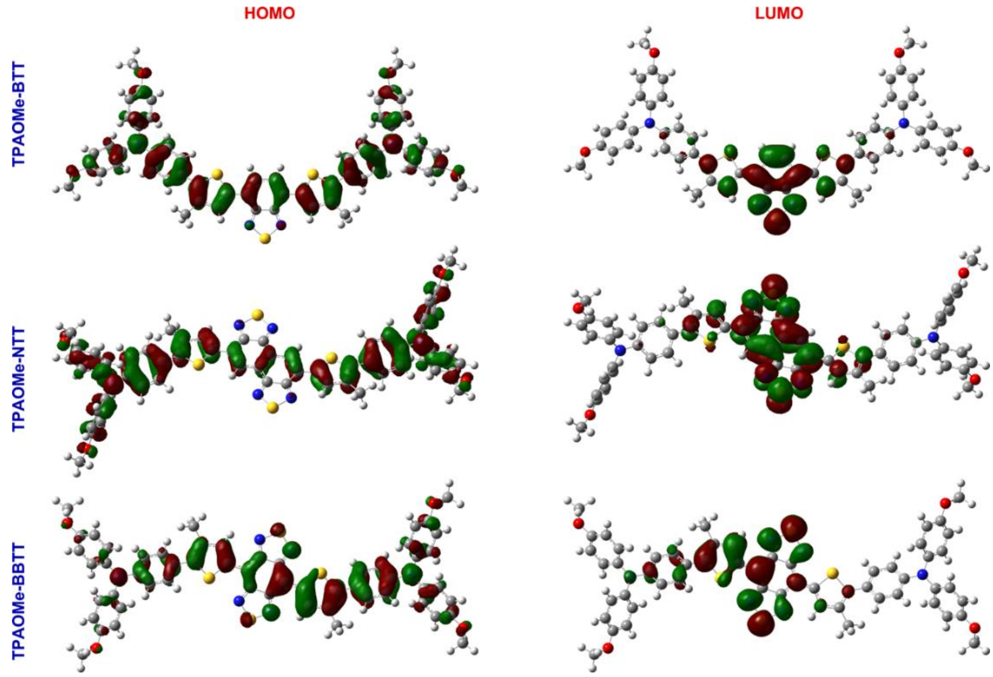

Supplementary Figure 13. The optimized ground-state geometry and molecular orbital (MO) diagrams of the BT, NT, and BBT analogues calculated at (U)B3LYP/6-31G*(*d,p*)* level of theory and basis set. The green and red surfaces are drawn at isovalue = |0.02| au, which represents the positive and negative contributions on the wavefunctions, respectively. Color code for the atoms: grey for C, blue for N, red for O, and yellow for S. In the case of the TPAOMe-BBTT, α-SOMO and α-LUMO is provided.

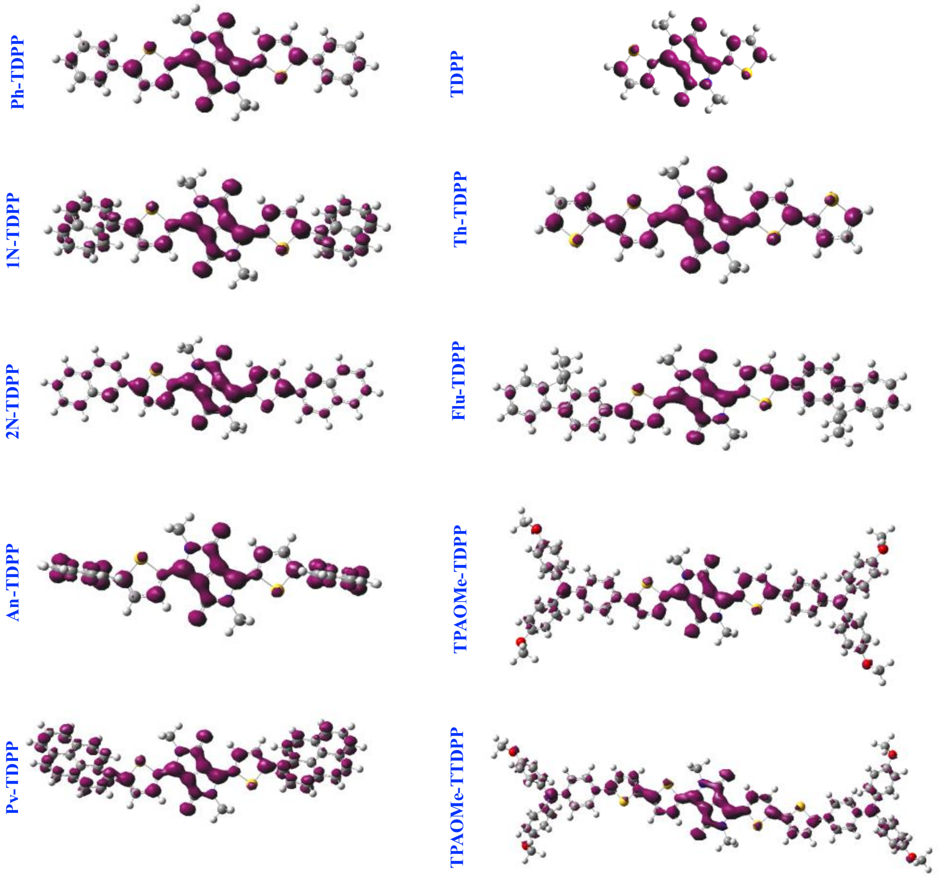

Supplementary Figure 14. FOD plots (σ = 0.002 e/Bohr^3) for DPP-based materials obtained from the FT-DFT at B3LYP/6-31G(*d,p*) level.

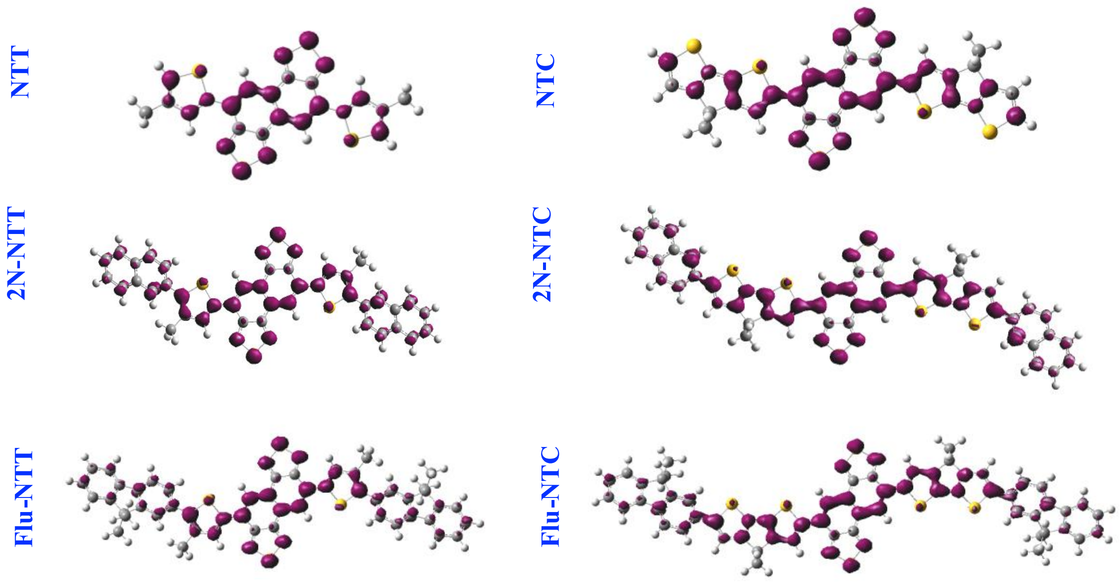

Supplementary Figure 15. FOD plots (σ = 0.002 e/Bohr^3) for NT-based materials obtained from the FT-DFT at B3LYP/6-31G(d,p) level.

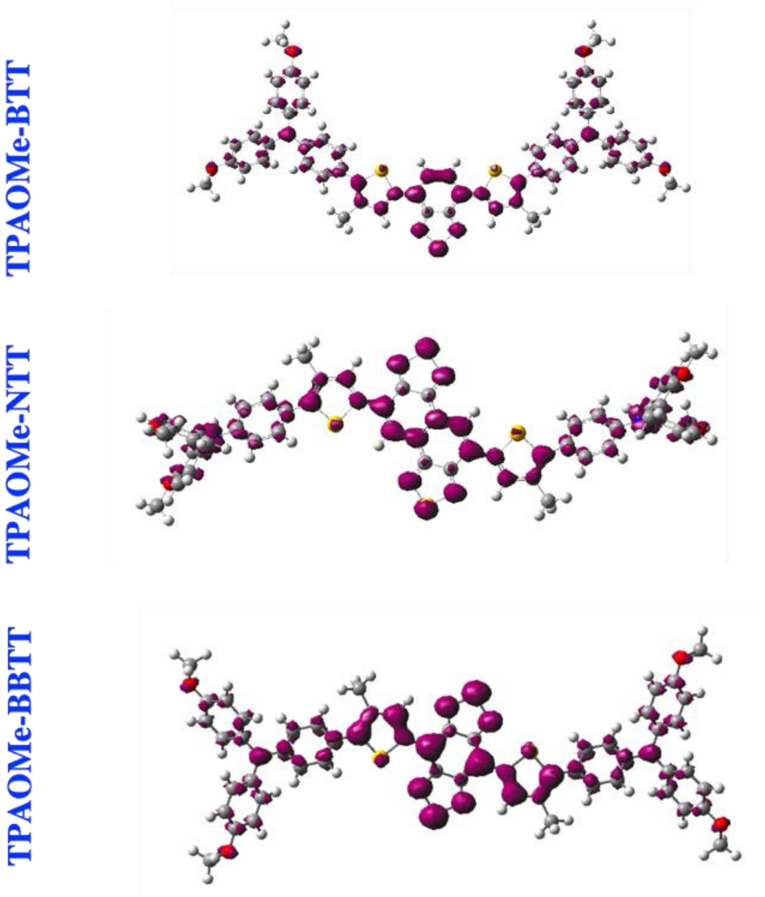

Supplementary Figure 16. FOD plots (σ = 0.002 e/Bohr^3) for BT, NT, and BBT analogues obtained from the FT-DFT at B3LYP/6-31G(d,p) level.

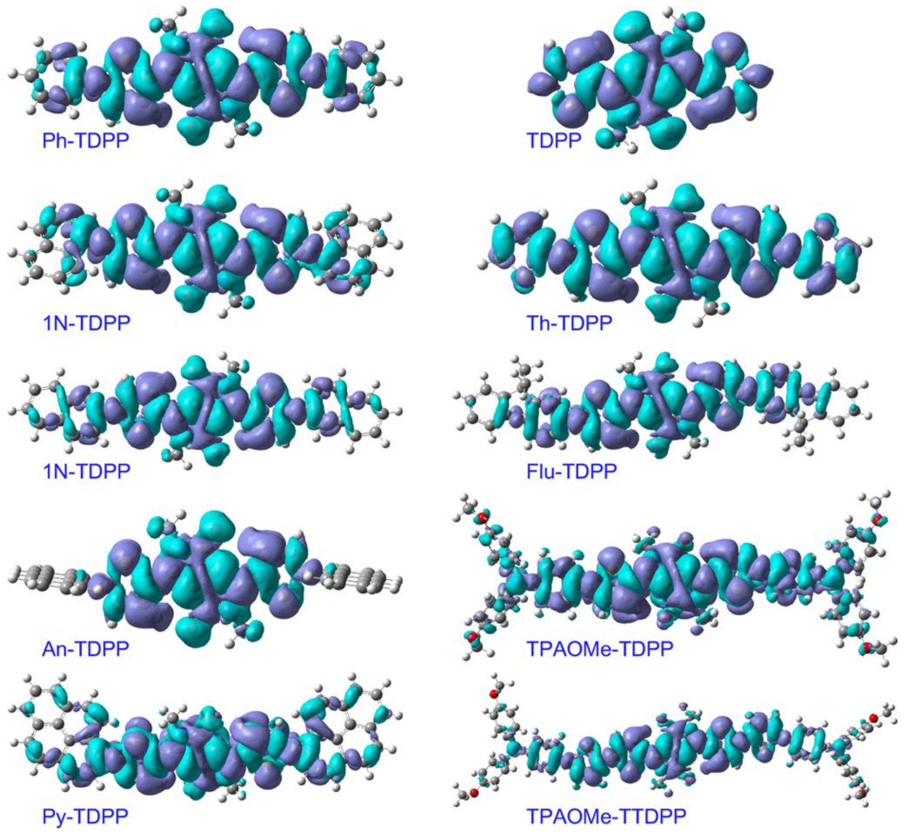

Supplementary Figure 17. Density difference ($\Delta\rho = S_1$-S_0) between the first singlet exited state (S_1) to the ground state (S_0) of the DPP-based molecules calculated at PCM/RBHandHLYP/6-31G(d,p) level of theory and basis set. The purple and blue-green surfaces represent the positive (density accumulation) and negative (density depletion) region, respectively. Isosurface value = 0.0001 au.

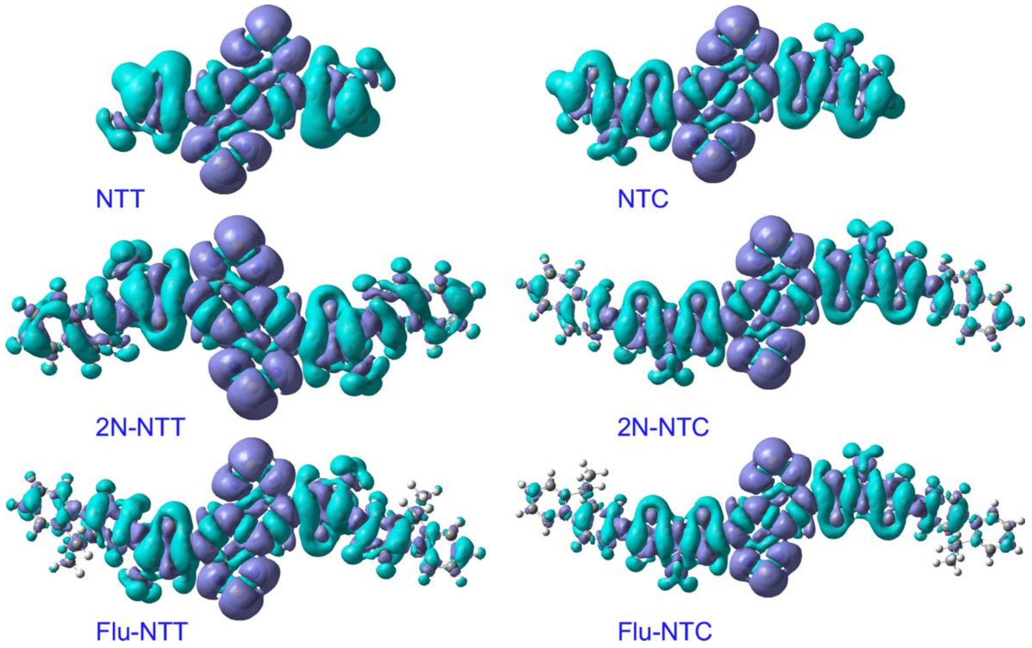

Supplementary Figure 18. Density difference ($\Delta\rho = S_1$-S_0) between the first singlet exited state (S_1) to the ground state (S_0) of the NT-based molecules calculated at PCM/RBHandHLYP/6-31G(*d,p*) level of theory and basis set. The purple and blue-green surfaces represent the positive (density accumulation) and negative (density depletion) region, respectively. Isosurface value = 0.0001 au.

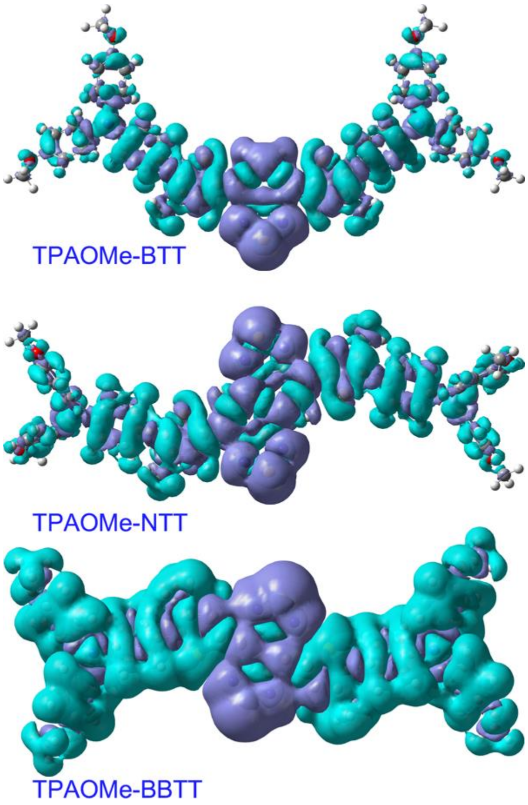

Supplementary Figure 19. Density difference ($\Delta\rho = S_1\text{-}S_0$) between the first singlet exited state (S_1) to the ground state (S_0) of the BT, NT, and BBT analogues calculated at PCM/(U)BHandHLYP/6-31G(d,p) level of theory and basis set. The purplish and blue-green surfaces represent the positive (density accumulation) and negative (density depletion) region, respectively. Isosurface value = 0.00001 au.

2.3. Electron spin resonance spectra

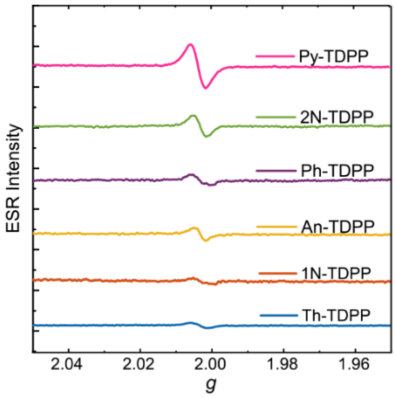

Supplementary Figure 20. ESR spectra measured at room temperature of DPP-based small molecules. The measurements were conducted under the same conditions and using the same molar quantity of each material at 0.02 mmol.

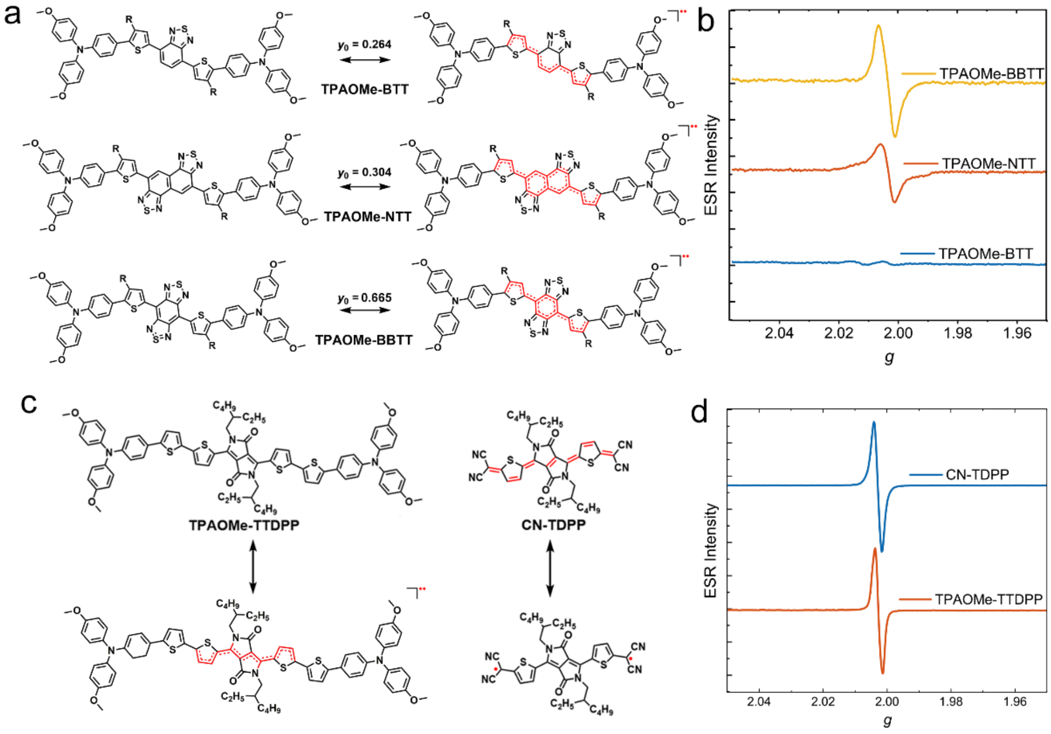

Supplementary Figure 21. (a) Resonance structures of TPAOMe-BTT, TPAOMe-NTT, and TPAOMe-BBTT. (b) ESR spectra of the small molecules. (c) Resonance structure of Chichibabin's analogue CN-TDPP and TPAOMe-TTDPP in this work. (d) ESR spectra of the two small molecules. The measurements were conducted under the same conditions and using the same molar quantity of each material at 0.02 mmol.

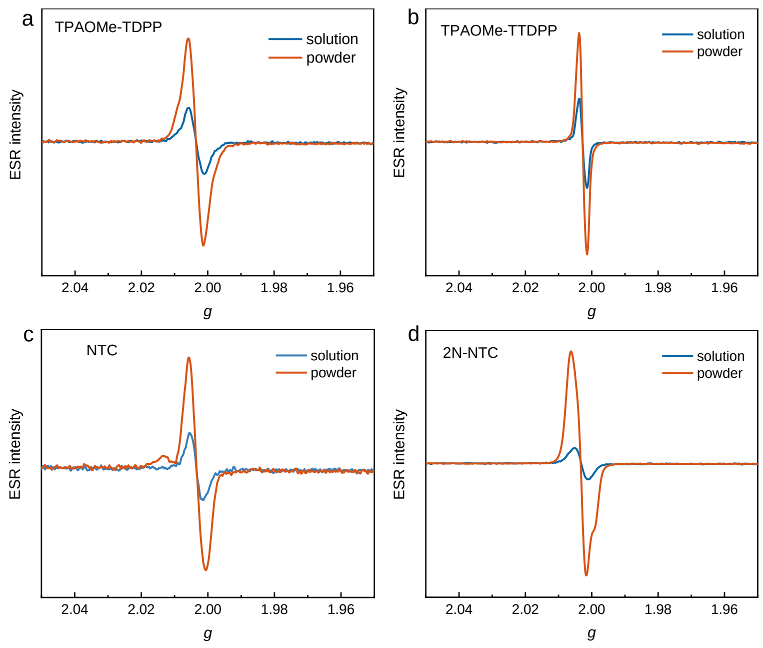

Supplementary Figure 22. ESR spectra of (a) TPAOMe-TDPP, (b)TPAOMe-TTDPP, (c) NTC, (d) 2N-NTC in toluene solutions and in powders. The EPR spectra in powders and saturated solutions were conducted under the same test conditions in EPR sample tube.

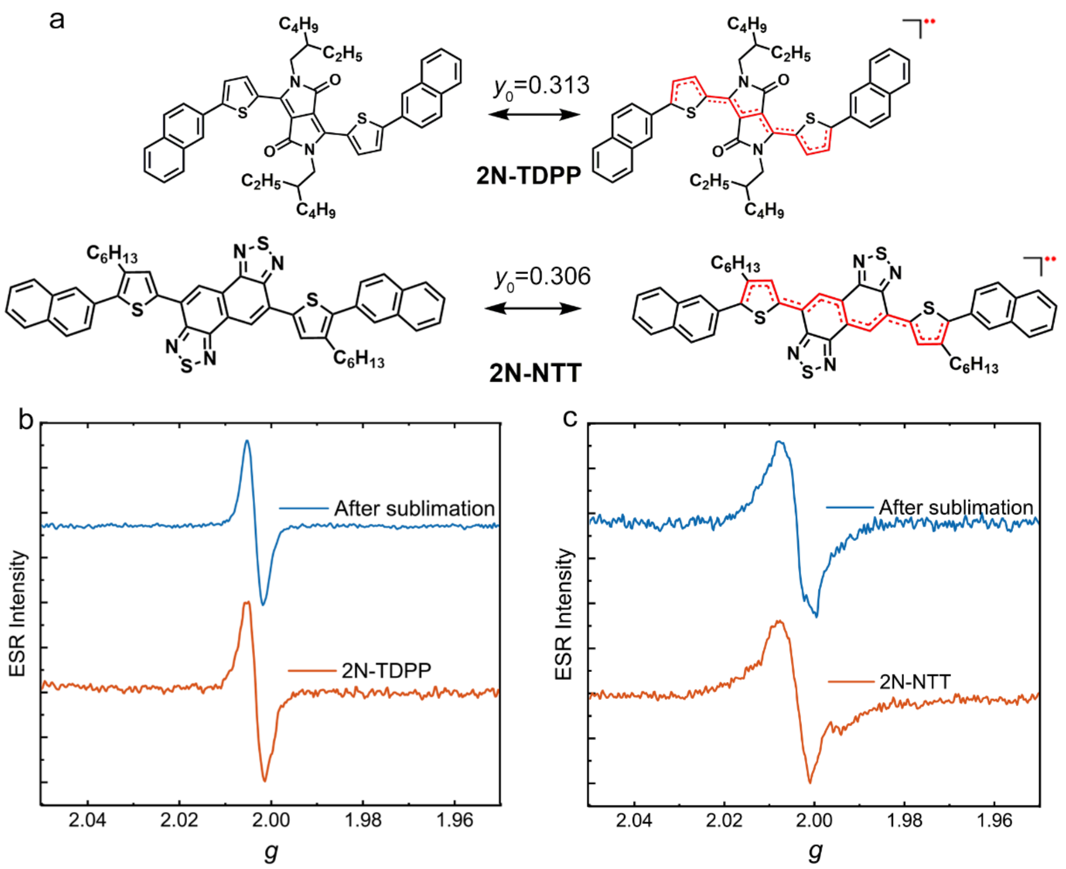

Supplementary Figure 23. (a) Resonance structures of 2N-TDPP and 2N-NTT. (b, c) ESR spectra of the small molecules before and after sublimation in an isolated N_2 environment, respectively. The measurements were conducted under the same conditions and using the same molar quantity of each material at 0.02 mmol.

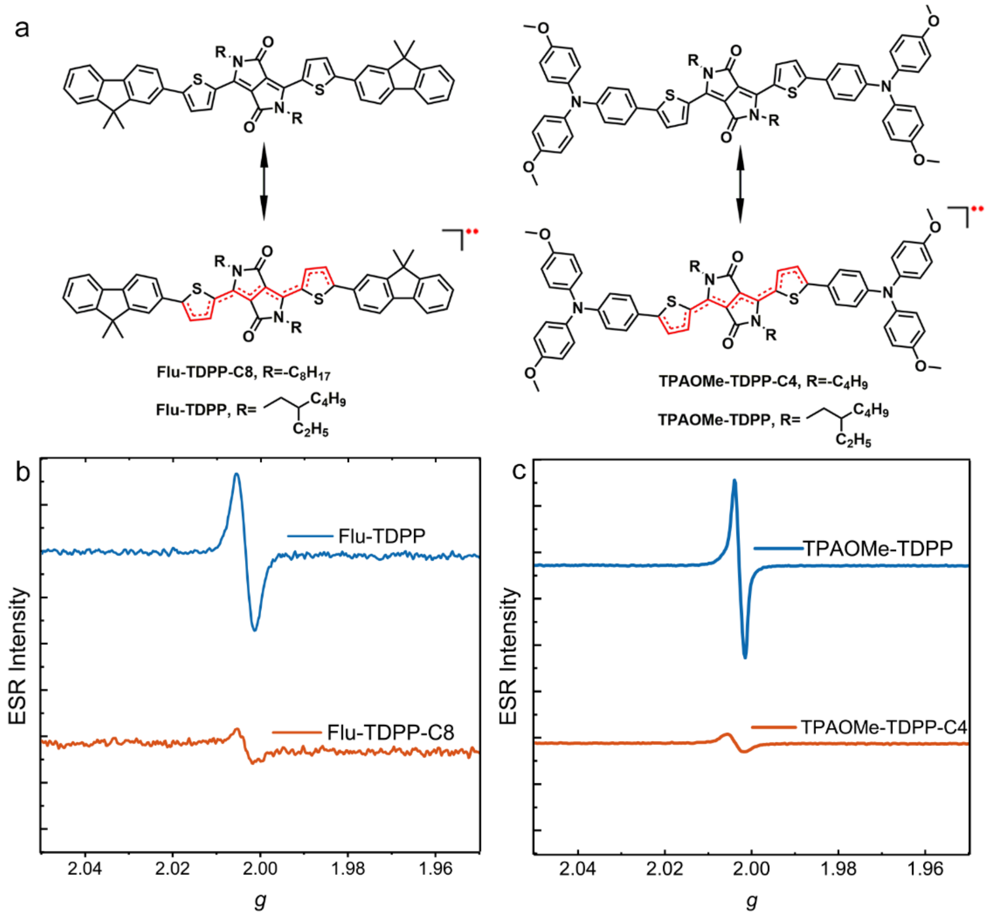

Supplementary Figure 24. (a) Resonance structures of Flu-TDPP with *n*-butyl (C8) and 2-ethylhexyl (EH) alkyl chains and TPAOMe-TDPP with *n*-octyl (C4) and EH alkyl chains. (b) ESR spectra of Flu-TDPP and Flu-TDPP-C8. (c) ESR spectra of TPAOMe-TDPP and TPAOMe-TDPP-C4. The measurements were conducted under the same conditions and using the same molar quantity of each material at 0.02 mmol. The DPP molecules with EH side chains show stronger ESR response compared to derivatives with different alkyl chains (C8), (C4).

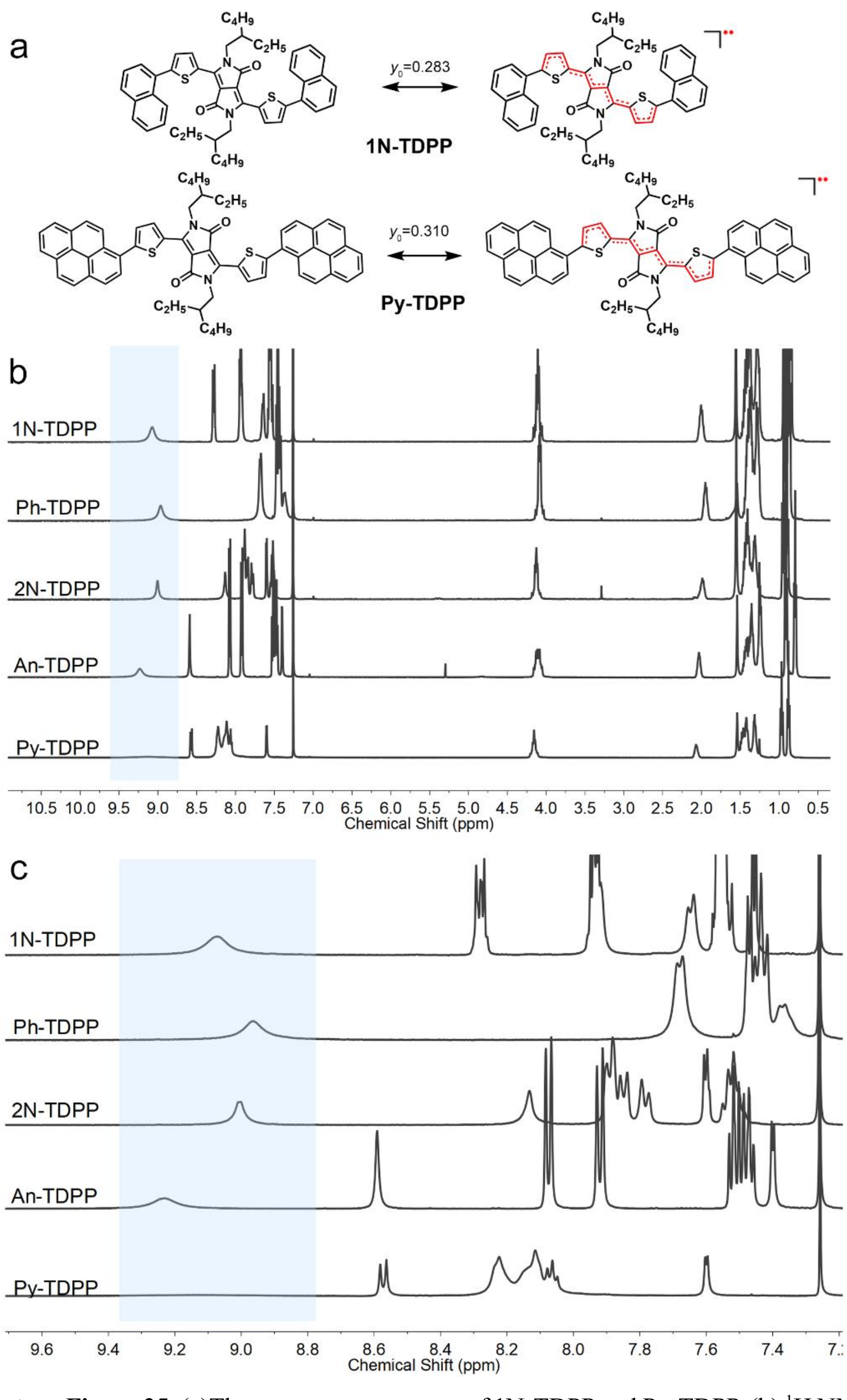

Supplementary Figure 25. (a)The resonance structures of 1N-TDPP and Py-TDPP. (b) ^{1}H NMR spectra and (c) their enlarged spectra in aromatic areas (7.2 to 9.7 ppm) of the DPP molecules in Chloroform-*d* at room temperature.

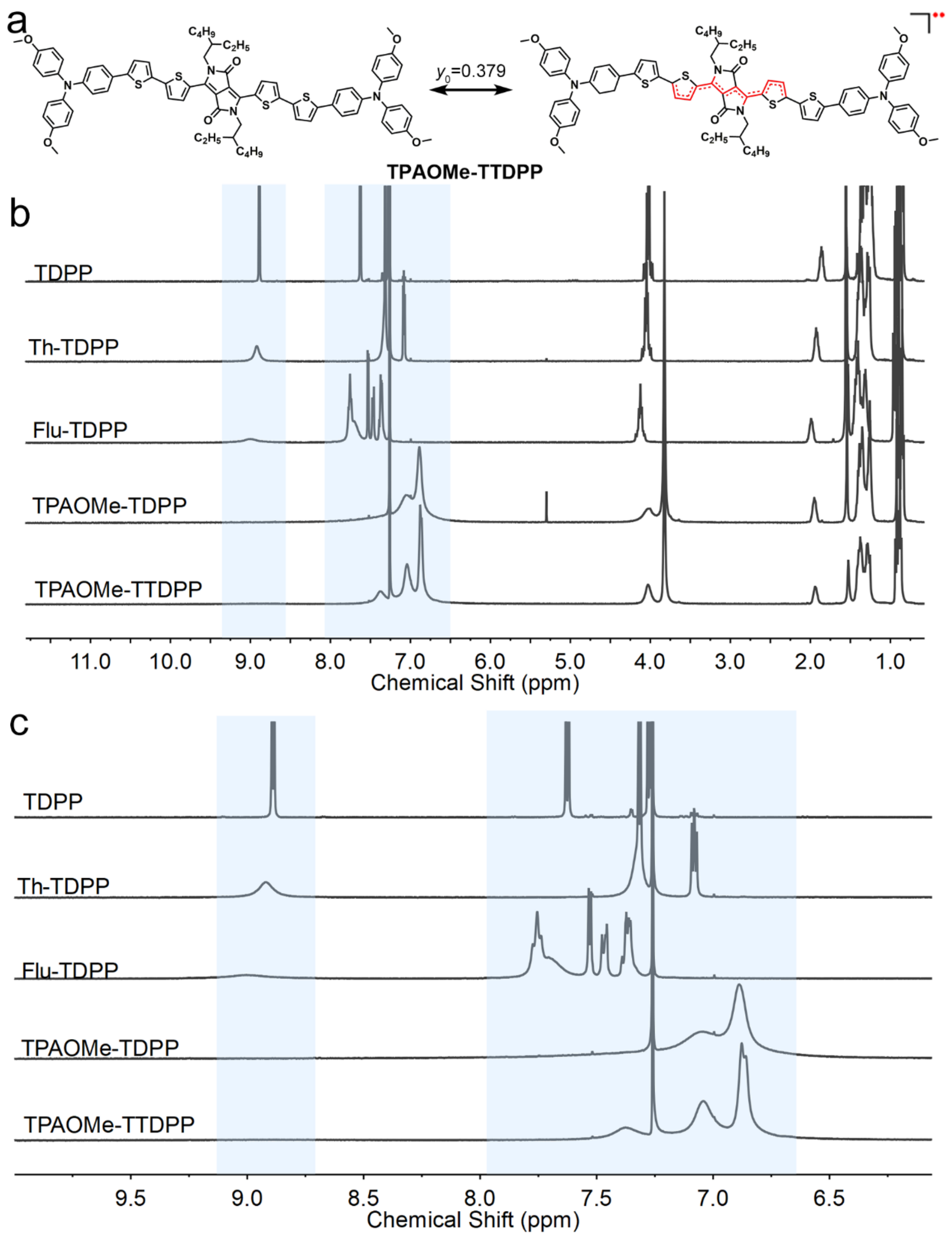

Supplementary Figure 26. (a) The resonance structures of TDPP and TPAOMe-TTDPP. (b) The ^{1}H NMR spectra and (c) their enlarged spectra in aromatic areas (6.0-10.0) of the DPP molecules in Chloroform-*d* at room temperature.

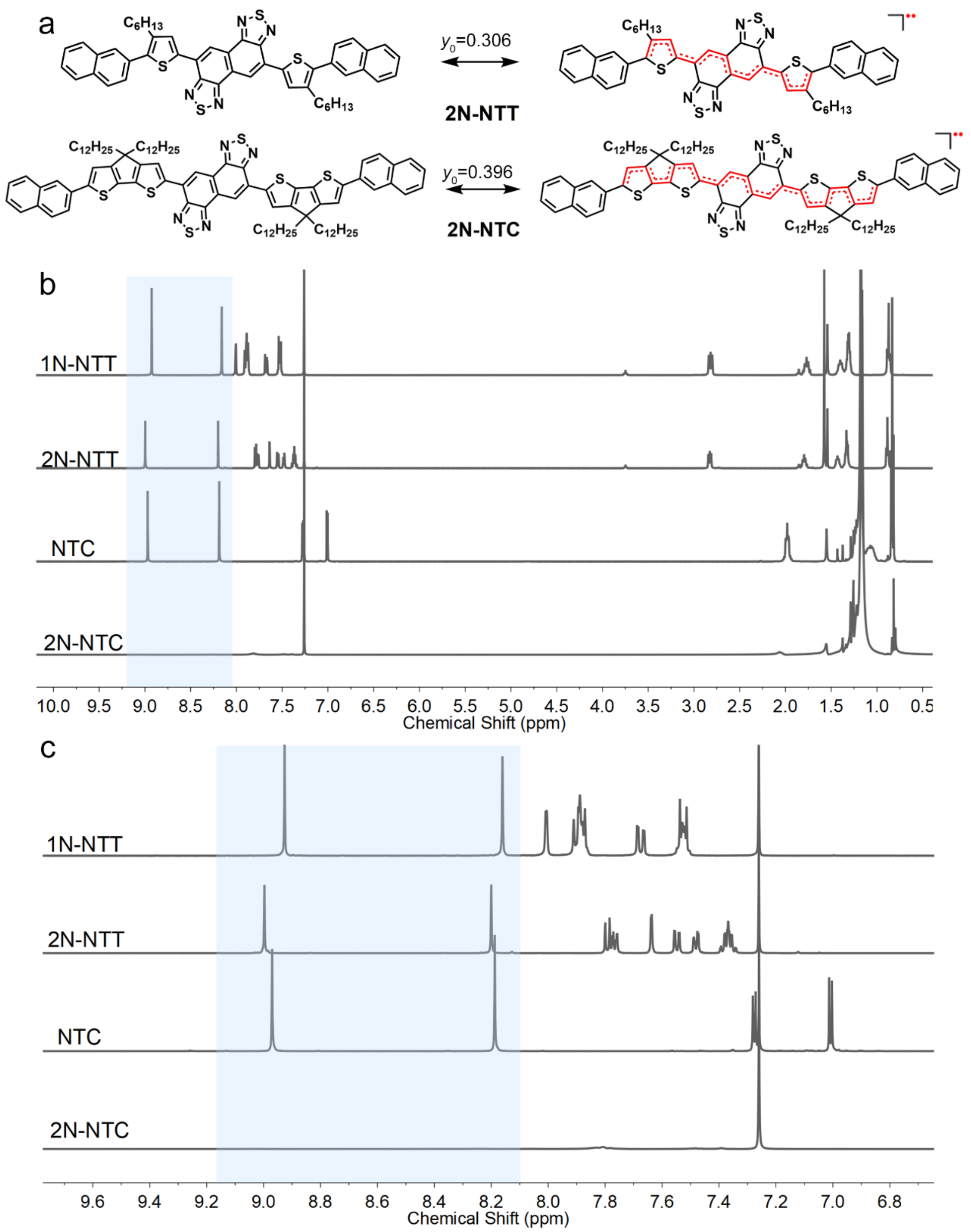

Supplementary Figure 27. (a) The resonance structures of 2N-NTT and 2N-NTC. (b) The ^{1}H NMR spectra and (c) their enlarged spectra in aromatic areas (6.6-9.8 ppm) of the NT molecules in Chloroform-*d* at room temperature.

2.4. X-ray crystallography and packing motifs of the single crystals

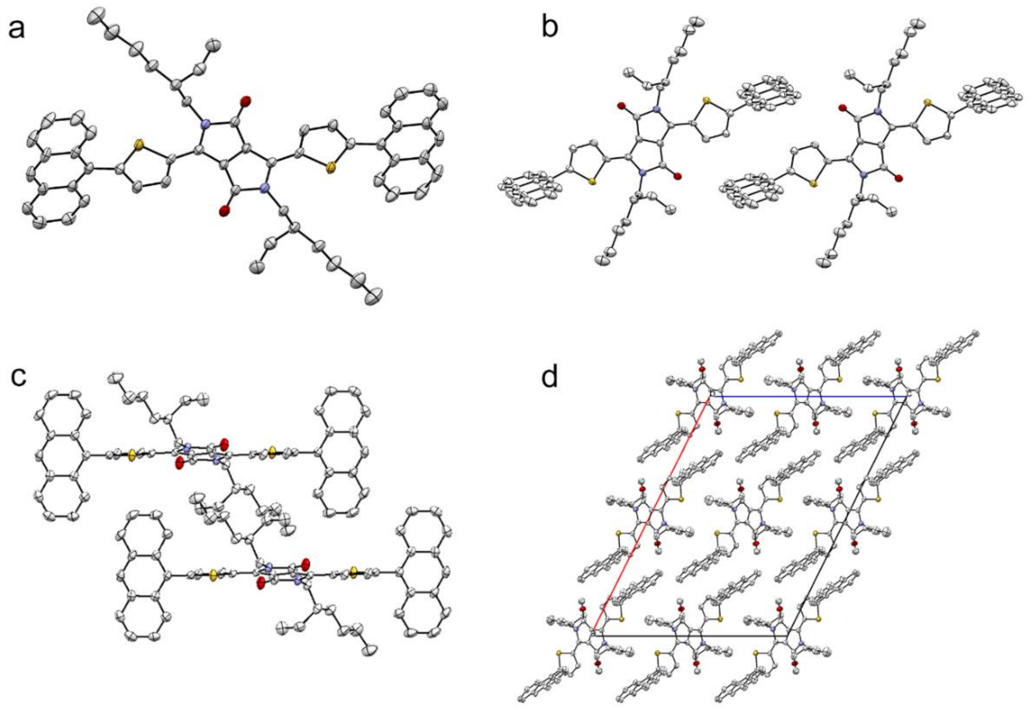

Supplementary Figure 28. (a) The ORTEP images of An-TDPP. (b) The top view and (c) side view of two crystal packing structure. (d) The solid-state molecular packing graphs. Hydrogens are omitted for clarity.

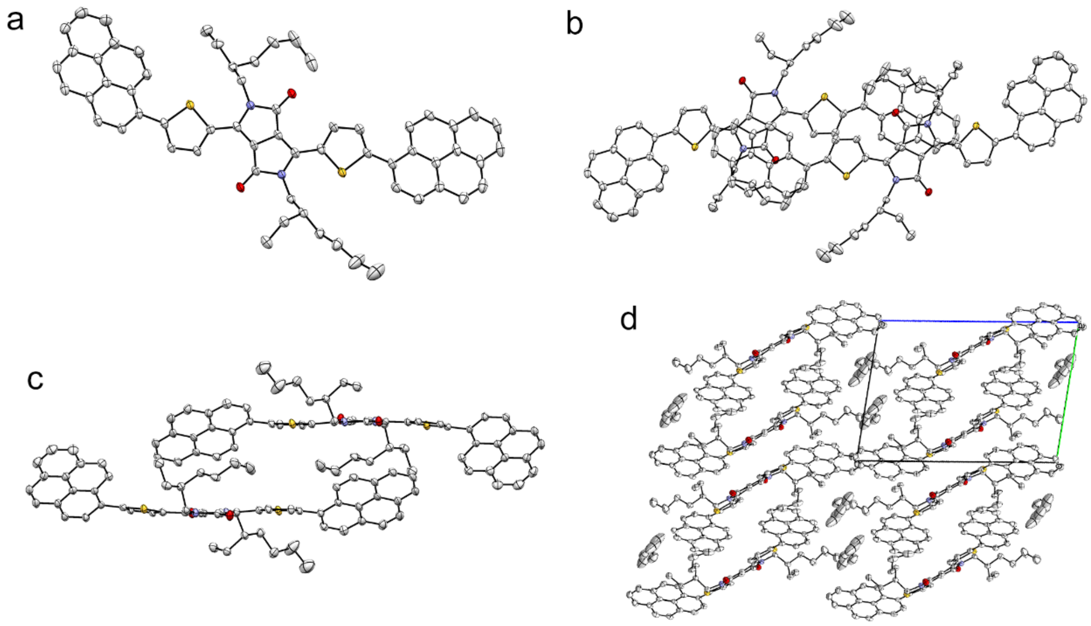

Supplementary Figure 29. (a) The ORTEP images of Py-TDPP. (b) The top view and (c) side view of two crystal packing structure. (d) The solid-state molecular packing graphs. Hydrogens are omitted for clarity.

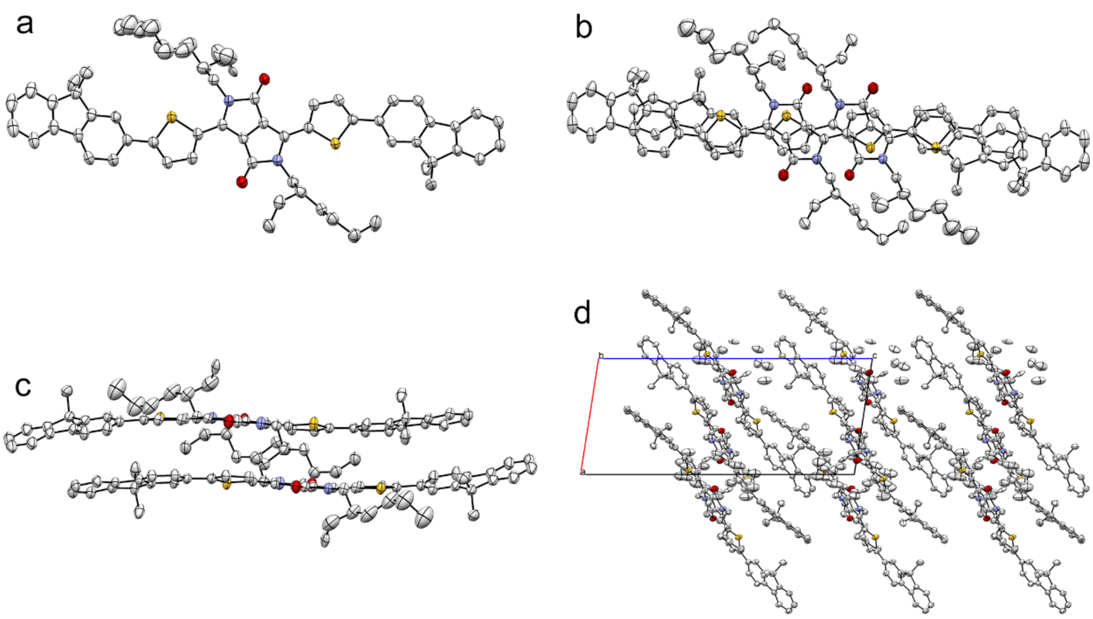

Supplementary Figure 30. (a) The ORTEP images of Flu-TDPP. (b) The top view and (c) side view of two crystal packing structure. (d) The solid-state molecular packing graphs. Hydrogens are omitted for clarity.

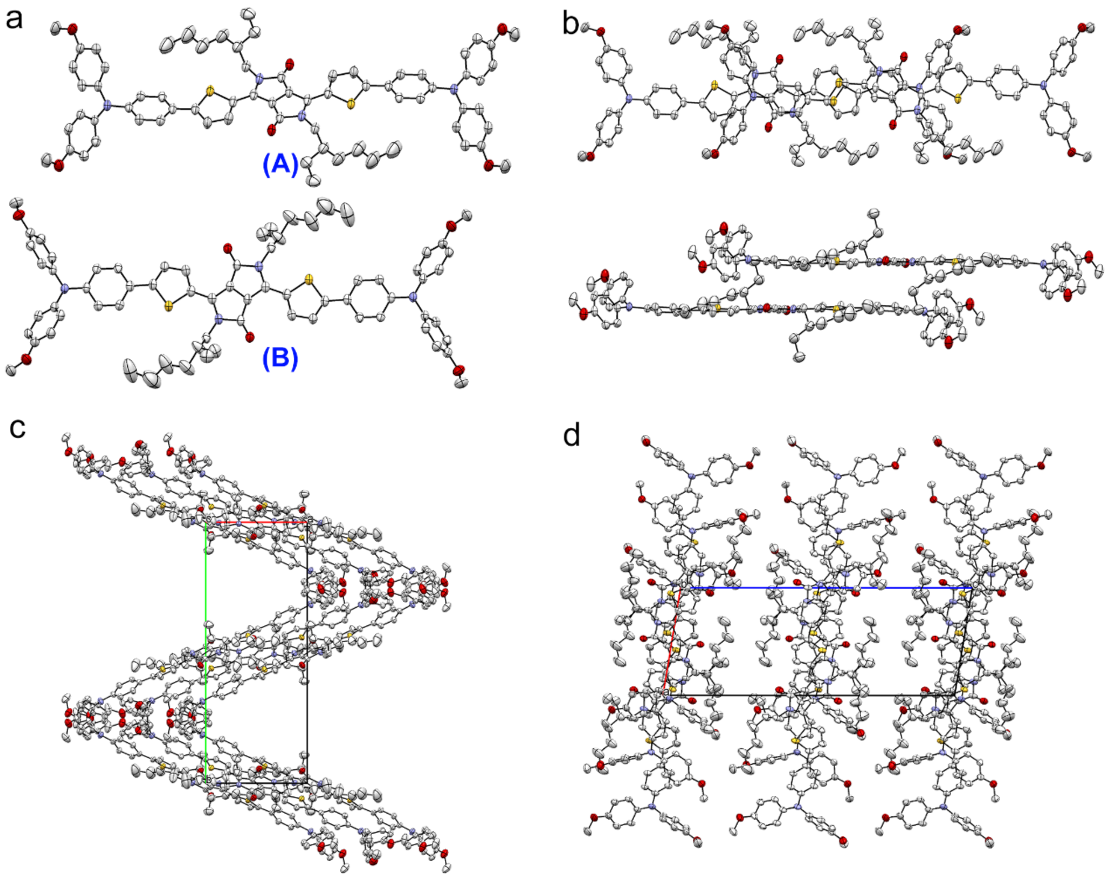

Supplementary Figure 31. (a) The ORTEP images of TPAOMe-TDPP. Two conformations were obtained: (A) dimer conformation (B) monomer conformation. (b) The top view and side view of two crystal packing structure. (c), (d) The solid-state molecular packing graphs. Hydrogens are omitted for clarity.

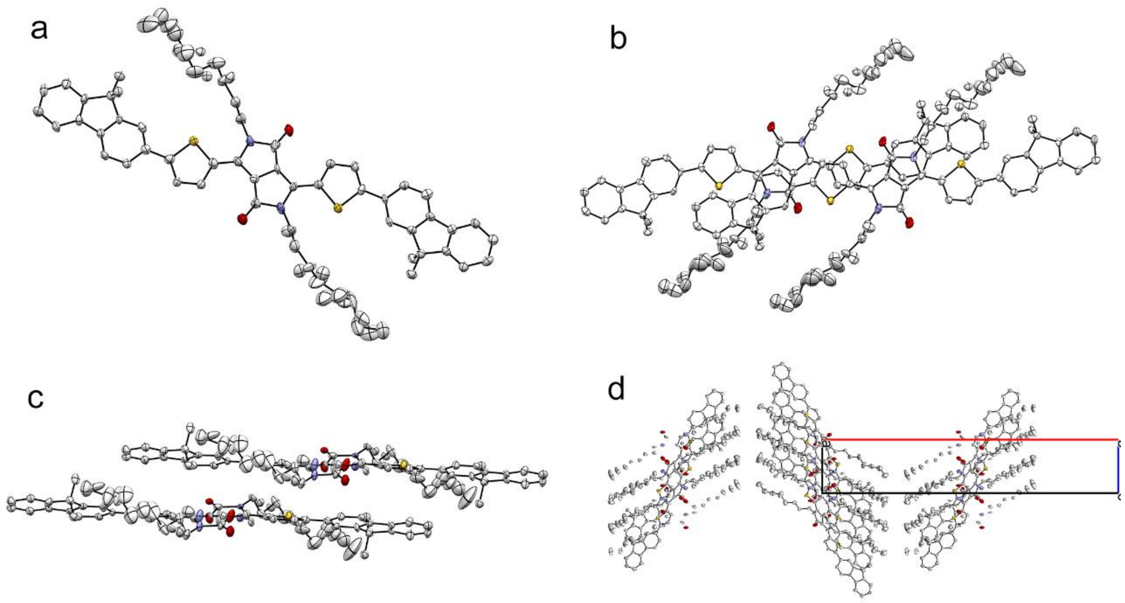

Supplementary Figure 32. (a) The ORTEP images of Flu-TDPP-C8. (b) The top view and (c) side view of two crystal packing structure. (d) The solid-state molecular packing graphs. Hydrogens are omitted for clarity.

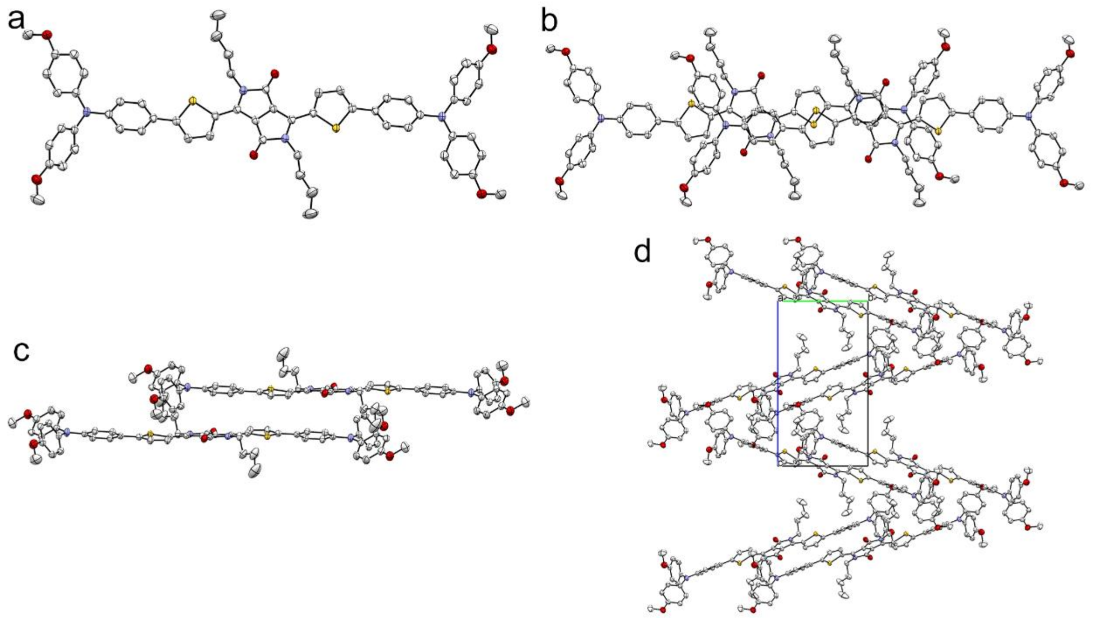

Supplementary Figure 33. (a) The ORTEP images of TPAOMe-TDPP-C4. (b) The top view and (c) side view of two crystal packing structure. (d) The solid-state molecular packing graphs. Hydrogens are omitted for clarity.

Supplementary Table 5. The two conformations of TPAOMe-TDPP

Conformation	*a* (Å)	*b* (Å)	*c* (Å)	*d* (Å)	*e* (Å)	*f* (Å)	*g* (Å)	Φ_α (°)	Φ_β (°)
A	1.461	1.367	1.397	1.376	1.437	1.404	1.406	5.17	14.44
B	1.458	1.374	1.398	1.365	1.446	1.394	1.422	6.50	10.20

Supplementary Table 6. The critical crystal data of DPP derivatives.

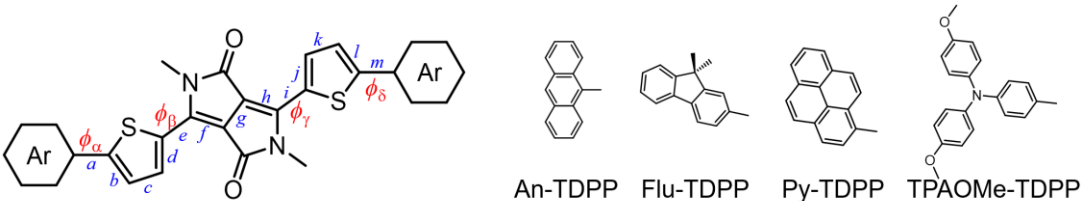

DPP	DFT				XRD					
D (Å) / ϕ (°)	TPAOMe	Flu	An	Py	TPAOMe	TPAOMe-C4	Flu	Flu-C8	An	Py
Φ_α	22.00	24.00	90.00	46.00	40.70	23.44	16.60	20.54	86.60	40.70
a	1.459	1.463	1.484	1.469	1.461	1.465	1.464	1.472	1.475	1.469
b	1.385	1.384	1.376	1.383	1.367	1.374	1.369	1.392	1.356	1.380
c	1.407	1.407	1.414	1.409	1.397	1.400	1.400	1.385	1.403	1.396
d	1.391	1.391	1.388	1.390	1.376	1.380	1.372	1.381	1.371	1.385
e	1.435	1.436	1.440	1.437	1.437	1.440	1.443	1.418	1.442	1.432
Φ_β	0.00	0.00	0.00	0.77	5.17	7.62	11.40	16.26	13.88	5.71
f	1.398	1.397	1.395	1.397	1.404	1.394	1.384	1.380	1.388	1.387
g	1.421	1.420	1.422	1.421	1.406	1.406	1.408	1.422	1.408	1.412
h	1.398	1.397	1.395	1.397	1.404	1.394	1.386	1.377	1.388	1.403
Φ_γ	0.00	0.00	0.00	0.00	5.17	7.62	11.40	18.09	13.88	5.71
i	1.435	1.436	1.440	1.438	1.437	1.440	1.443	1.439	1.442	1.431
j	1.391	1.391	1.388	1.389	1.376	1.380	1.383	1.376	1.371	1.379
k	1.407	1.407	1.414	1.409	1.397	1.400	1.400	1.418	1.403	1.409
l	1.385	1.384	1.376	1.382	1.367	1.374	1.373	1.381	1.356	1.379
m	1.460	1.463	1.484	1.472	1.461	1.465	1.469	1.444	1.475	1.468
Φ_δ	19.91	24.00	90.00	43.95	40.70	23.44	16.60	20.51	86.60	40.70

[a]The data of in the table is one of the two crystal conformations of TPAOMe-TDPP.

2.5. X-ray crystallographic data for TPAOMe-TDPP

Supplementary Table 7. Crystal data and structure refinement for TPAOMe-TDPP

Identification code	TPAOMe-TDPP
Empirical formula	$C_{70}H_{74}N_4O_6S_2$
Formula weight	1131.45
Temperature/K	149.99(10)
Crystal system	monoclinic
Space group	P21/c
a/Å	9.94498(12)
b/Å	25.2472(3)
c/Å	26.5652(3)
α/°	90

β/°	99.3209(10)
γ/°	90
Volume/Å^3	6582.00(13)
Z	4
ρcalcg/cm^3	1.142
μ/mm^{-1}	1.142
F(000)	2408.0
Crystal size/mm^3	0.15 × 0.05 × 0.03
Radiation	CuKα (λ = 1.54184)
2Θ range for data collection/°	4.86 to 134.158
Index ranges	-11 ≤ h ≤ 9, -30 ≤ k ≤ 20, -28 ≤ l ≤ 31
Reflections collected	33582
Independent reflections	11475 [Rint= 0.0307, Rsigma= 0.0370]
Data/restraints/parameters	11475/38/747
Goodness-of-fit on F^2	1.044
Final R indexes [I>=2σ (I)]	R1= 0.0672, wR2= 0.1879
Final R indexes [all data]	R1= 0.0738, wR2= 0.1935
Largest diff. peak/hole / e Å^{-3}	1.54/-0.52

Supplementary Table 8. Fractional Atomic Coordinates (×10^4) and Equivalent Isotropic Displacement Parameters (Å^2×10^3) for TPAOMe-TDPP. Ueq is defined as 1/3 of of the trace of the orthogonalised UIJtensor.

Atom	x	y	z	U(eq)
S01	10554.4(6)	5591.6(2)	4701.8(2)	38.32(1)
S02	5795.6(6)	5640.0(3)	205.2(3)	44.77(1)
O003	14735.7(18)	4444.5(7)	4011.8(7)	43.9(4)
O004	10499(2)	4526.9(8)	1031.6(8)	49.9(5)
O005	-3473(2)	7700.9(10)	-1990.6(8)	63.9(6)
N006	4731(2)	6830.9(9)	5088.7(8)	39.7(5)
O007	1804(2)	7319.2(10)	3171.8(8)	62.7(6)
N008	13264(2)	4945.2(8)	4414.1(8)	36.3(5)
N009	-320(2)	6852.1(9)	-248.7(9)	43.5(5)
O00A	-1346(2)	7439.5(11)	1673.0(8)	70.5(7)
N00B	8727(2)	5015.5(9)	573.8(9)	43.1(5)
O00C	3494(3)	7987.7(10)	6745.9(9)	66.7(6)

C00D	8632(2)	6130.3(9)	5168.3(10)	36.4(5)
C00E	14538(3)	4707.6(10)	4382.6(10)	37.6(5)
C00F	-608(3)	6999.3(10)	240.9(10)	40.2(6)
C00G	14601(2)	5151.1(9)	5147.0(10)	36.6(5)
C00H	2766(3)	6029.1(10)	87.9(11)	39.9(6)
C00I	10002(3)	5911.7(10)	5205.5(10)	37.9(5)
C00J	7588(3)	6026.6(10)	4756.7(10)	38.0(5)
C00K	4390(2)	7114.4(10)	5516.4(10)	36.5(5)
C00L	13285(2)	5204.2(9)	4876.0(10)	36.4(5)
C00M	3954(3)	6949.3(10)	4597.1(10)	38.3(5)
C00N	3478(3)	6129.1(10)	-313.3(11)	41.2(6)
C00O	10024(3)	4762.6(10)	637.5(12)	44.0(6)
C00P	918(3)	6599.4(10)	-284.7(11)	40.2(6)
C00Q	8306(3)	6455.4(10)	5556.6(10)	40.4(6)
C00R	-1129(3)	7082.9(10)	-687.6(10)	39.8(6)
C00S	9499(3)	5141.9(10)	-163.1(11)	42.0(6)
C00T	6010(2)	6588.5(9)	5117.3(10)	35.6(5)
C00U	349(3)	7276.4(11)	583.1(11)	44.5(6)
C00V	2597(3)	6814.1(10)	4486.6(10)	41.1(6)
C00W	6305(3)	6249.5(10)	4734.5(10)	38.7(5)
C00X	12227(3)	5674.5(11)	5530.3(11)	44.9(6)
C00Y	1596(3)	6685.2(11)	-694.7(11)	43.2(6)
C00Z	1506(3)	6255.5(10)	103.3(11)	41.3(6)
C010	2449(3)	7186.6(12)	3651.7(10)	47.6(7)
C011	2847(3)	6457.6(11)	-707.8(11)	44.9(6)
C012	8388(3)	5232.9(10)	90.9(11)	42.5(6)
C013	11030(3)	5921.9(11)	5618.8(11)	45.0(6)
C014	4254(3)	7665.2(10)	5497.7(10)	40.8(6)
C015	-1482(3)	7615.6(11)	-693.9(11)	43.0(6)
C016	7039(3)	6675.6(10)	5536.6(11)	40.7(6)
C017	4833(3)	5913.2(10)	-330.3(11)	44.1(6)
C018	3951(3)	7937.3(11)	5914.2(11)	47.6(7)

C019	-1851(3)	6874.8(11)	379.1(11)	44.8(6)
C01A	1829(3)	6933.8(11)	4014.7(11)	44.3(6)
C01B	4199(3)	6854.6(11)	5955.5(11)	43.3(6)
C01C	12110(3)	4858.0(11)	4004.8(11)	42.2(6)
C01D	12158(3)	5469.9(10)	5051.2(10)	38.0(5)
C01E	-1567(3)	6775.5(12)	-1117.7(11)	48.7(7)
C01F	4556(3)	7216.7(11)	4233.2(10)	43.0(6)
C01G	72(3)	7411.1(12)	1055.9(11)	49.7(7)
C01H	3890(3)	7126.9(12)	6374.8(11)	47.6(6)
C01I	-2274(3)	7833.2(12)	-1122.9(11)	48.0(7)
C01J	-1175(3)	7281.9(12)	1193.2(11)	50.3(7)
C01K	3806(3)	7329.1(12)	3759.1(11)	48.5(7)
C01L	-2696(3)	7525.1(13)	-1547.1(11)	48.7(7)
C01M	-2142(3)	7015.6(11)	854.1(12)	48.0(7)
C01N	7130(3)	5489.1(10)	-116.4(12)	44.4(6)
C01O	3771(3)	7675.1(12)	6355.0(11)	47.1(6)
C01P	7900(3)	4983.9(11)	979.7(12)	47.7(6)
C01Q	-2344(3)	6988.9(13)	-1542.9(12)	51.2(7)
C01R	6812(3)	5656.1(13)	-614.5(13)	56.1(8)
C01S	5531(3)	5894.0(13)	-733.1(13)	56.6(8)
C01T	11930(4)	5291(2)	3590.9(14)	80.2(12)
C01U	8009(4)	5469.7(15)	1330.6(14)	65.2(9)
C01V	426(3)	7149.3(19)	3021.2(13)	74.8(11)
C01W	3193(4)	7736.6(18)	7189.7(13)	76.9(11)
C01X	-3711(4)	8256.1(17)	-2032.6(15)	78.3(11)
C01Y	13043(4)	5930.7(15)	3062.2(15)	72.0(10)
C01Z	-2637(4)	7311(2)	1824.3(15)	82.1(12)
C020	9459(4)	5564.6(16)	1597.2(16)	76.8(11)
C021	13149(4)	5431.8(17)	3375.4(16)	75.7(10)
C022	10701(4)	5129(2)	3184.7(16)	98.5(16)
C023	7010(5)	5411(2)	1707.1(18)	90.3(13)
C024	9698(5)	6080.9(16)	1893.1(17)	86.4(12)

C025	5567(5)	5469(2)	1506(2)	102.6(16)
C026	9340(4)	5182(3)	3345(2)	121(2)
C028	4622(6)	5397(3)	1895(2)	128(2)
C02A	3118(6)	5482(3)	1674(3)	145(3)
C1	6862(6)	5280(3)	3286(3)	150(3)
C0AA	8090(6)	5092(4)	3045(3)	167(3)

Supplementary Table 9. Anisotropic Displacement Parameters (Å^2×10^3) for TPAOMe-TDPP. The Anisotropic displacement factor exponent takes the form: -2π^2[h^{2}a*^{2}U11+2hka*b*U12+…].

Atom	U_{11}	U_{22}	U_{33}	U_{23}	U_{13}	U_{12}
S01	31.5(3)	36.3(3)	48.6(4)	5.0(3)	10.9(3)	5.9(2)
S02	31.1(3)	41.7(4)	62.9(4)	-6.5(3)	11.7(3)	7.3(2)
O003	37.5(10)	42.3(10)	52.5(11)	-1.7(8)	9.0(8)	5.1(8)
O004	41.6(11)	45.3(11)	64.6(13)	-0.1(9)	13.6(9)	11.4(8)
O005	57.1(13)	81.3(16)	54.2(12)	13.8(11)	12.0(10)	22.5(11)
N006	33.4(11)	42.0(12)	44.7(12)	2.4(9)	9.2(9)	9.6(9)
O007	47.1(12)	94.5(18)	45.9(11)	9.2(11)	5.5(9)	11.1(11)
N008	27.4(10)	34.5(11)	47.8(12)	4.3(9)	8.5(8)	2.7(8)
N009	33.2(12)	46.7(13)	51.5(13)	-2.1(10)	9.1(9)	13.4(9)
O00A	57.5(14)	105.4(19)	51.8(12)	0.1(12)	19.1(10)	26.7(13)
N00B	30.8(11)	37.9(11)	62.9(14)	-5.2(10)	14.2(10)	5.3(9)
O00C	77.7(16)	69.1(15)	54.8(13)	-16.0(11)	15.2(11)	10.7(12)
C00D	31.1(13)	30.0(12)	49.9(14)	5.2(10)	12.0(10)	-0.4(9)
C00E	33.5(13)	31.3(12)	49.3(14)	6.4(11)	11.0(11)	2.9(10)
C00F	32.5(13)	38.0(13)	51.4(15)	1.7(11)	11.0(11)	10.0(10)
C00G	32.5(13)	31.6(12)	47.6(14)	5.8(10)	12.1(10)	3.9(10)
C00H	34.4(13)	31.5(12)	54.2(15)	-3.2(11)	7.9(11)	2.8(10)
C00I	32.4(13)	31.9(12)	51.2(14)	5.5(11)	12.0(11)	1.9(10)
C00J	36.4(14)	32.6(12)	47.2(14)	0.8(10)	13.6(11)	1.3(10)
C00K	26.8(12)	37.3(13)	46.3(14)	-0.1(11)	8.7(10)	2.8(10)
C00L	32.1(13)	29.1(12)	49.4(14)	7.5(10)	11.1(10)	0.3(9)
C00M	34.5(13)	35.6(13)	45.4(14)	2.7(11)	8.4(10)	9.0(10)
C00N	31.6(13)	32.4(13)	60.7(16)	-6.6(11)	10.8(11)	3.1(10)
C00O	32.3(14)	33.8(13)	66.9(18)	-7.6(12)	11.0(12)	5.5(10)
C00P	31.0(13)	35.0(13)	55.1(15)	-4.9(11)	8.3(11)	4.5(10)
C00Q	32.8(13)	36.5(13)	51.5(15)	-3.0(11)	5.8(11)	-0.5(10)
C00R	27.8(13)	42.5(14)	50.2(15)	-2.1(11)	9.8(10)	6.3(10)
C00S	29.8(13)	34.2(13)	63.6(17)	-4.8(12)	12.2(11)	6.0(10)
C00T	29.5(12)	31.0(12)	48.4(14)	3.7(10)	12.2(10)	1.3(9)

C00U	33.4(14)	46.2(15)	55.1(16)	1.2(12)	11.3(11)	6.7(11)
C00V	37.1(14)	38.5(14)	49.9(15)	2.2(11)	13.8(11)	3.2(11)
C00W	34.1(13)	37.5(13)	45.0(14)	1.5(11)	7.3(10)	1.1(10)
C00X	33.0(14)	45.5(15)	55.8(16)	2.7(12)	6.0(11)	6.3(11)
C00Y	35.9(14)	40.1(14)	54.4(16)	2.2(12)	9.9(11)	5.7(11)
C00Z	33.4(13)	38.4(14)	53.1(15)	-1.7(11)	10.3(11)	2.8(10)
C010	45.5(16)	55.3(17)	42.4(14)	2.1(12)	8.5(12)	14.2(13)
C011	37.4(14)	42.5(14)	56.7(16)	-2.2(12)	13.6(12)	4.1(11)
C012	31.2(13)	31.2(13)	66.7(18)	-8.6(12)	13.0(12)	0.8(10)
C013	34.5(14)	49.3(16)	51.9(15)	-3.2(12)	9.1(11)	6.9(11)
C014	35.4(14)	38.6(14)	48.4(14)	3.3(11)	6.2(11)	3.1(10)
C015	36.8(14)	44.1(15)	50.6(15)	-4.5(12)	14.3(11)	7.1(11)
C016	35.4(14)	36.1(13)	51.5(15)	-4.6(11)	9.7(11)	0.6(10)
C017	32.3(14)	35.5(13)	65.1(17)	-6.2(12)	9.9(12)	4.2(10)
C018	45.8(16)	39.0(14)	56.1(16)	-3.8(12)	2.2(12)	7.0(11)
C019	33.9(14)	40.5(14)	60.5(17)	4.8(12)	9.4(12)	4.6(11)
C01A	30.9(13)	50.3(15)	52.5(15)	-0.9(12)	8.6(11)	4.6(11)
C01B	40.1(15)	37.9(14)	52.7(15)	3.0(12)	9.9(11)	1.4(11)
C01C	31.3(13)	42.3(14)	53.2(15)	-1.8(12)	7.6(11)	-1.5(11)
C01D	36.8(14)	29.6(12)	49.3(14)	5.9(10)	12.2(11)	2.4(10)
C01E	44.5(16)	42.1(15)	60.2(17)	-3.7(13)	11.1(13)	4.0(12)
C01F	33.2(14)	46.4(15)	51.5(15)	2.3(12)	13.4(11)	3.5(11)
C01G	40.9(16)	54.3(17)	53.4(16)	-4.3(13)	5.7(12)	9.6(12)
C01H	43.1(15)	56.2(17)	45.0(15)	3.3(13)	12.1(12)	-2.9(12)
C01I	45.6(16)	47.7(15)	54.9(16)	5.3(13)	20.8(13)	16.5(12)
C01J	47.6(17)	58.1(18)	48.3(15)	7.6(13)	16.7(13)	20.3(13)
C01K	44.5(16)	53.4(17)	50.2(16)	9.1(13)	15.8(12)	8.4(12)
C01L	35.5(15)	63.3(18)	50.0(16)	7.2(13)	14.8(12)	11.3(12)
C01M	36.6(15)	49.1(16)	61.3(17)	11.8(13)	16.9(12)	10.5(12)
C01N	33.3(14)	33.6(13)	68.2(18)	-8.7(12)	13.7(12)	3.5(10)
C01O	40.5(15)	51.5(16)	48.7(15)	-10.4(13)	5.4(12)	6.3(12)
C01P	38.3(15)	44.8(15)	62.6(17)	-5.0(13)	16.4(12)	5.0(11)
C01Q	41.5(16)	57.9(18)	54.3(17)	-6.7(14)	8.1(12)	2.6(13)
C01R	44.7(17)	59.1(19)	69(2)	0.5(15)	21.5(14)	13.5(13)
C01S	43.2(17)	65(2)	64.6(19)	4.7(15)	16.8(14)	18.1(14)
C01T	51(2)	120(3)	65(2)	35(2)	-5.3(16)	-25(2)
C01U	62(2)	64(2)	73(2)	-13.1(17)	22.2(17)	5.5(16)
C01V	48(2)	120(3)	54.4(19)	4(2)	2.8(15)	19(2)
C01W	88(3)	93(3)	52.8(19)	-13.7(19)	18.4(18)	7(2)
C01X	81(3)	88(3)	69(2)	24(2)	21.4(19)	38(2)

C01Y	83(3)	63(2)	71(2)	13.8(18)	15.8(19)	-5.3(18)
C01Z	68(2)	119(3)	68(2)	15(2)	36.9(19)	30(2)
C020	78(3)	69(2)	80(2)	-16.3(19)	3(2)	11.5(19)
C021	68(2)	82(3)	78(2)	15(2)	15.1(19)	-0.4(19)
C022	62(2)	147(4)	78(3)	41(3)	-12.0(19)	-26(2)
C023	88(3)	99(3)	95(3)	-20(3)	47(2)	6(2)
C024	106(3)	63(2)	83(3)	-9(2)	-6(2)	2(2)
C025	88(3)	104(4)	129(4)	-29(3)	58(3)	-9(3)
C026	68(3)	162(5)	128(4)	79(4)	1(2)	-5(3)
C028	103(4)	149(5)	151(5)	-30(4)	75(4)	-1(4)
C02A	93(4)	186(7)	172(6)	-72(5)	68(4)	-25(4)
C1	75(3)	192(7)	188(6)	108(5)	34(4)	25(4)
C0AA	96(3)	254(8)	143(5)	11(5)	-8(3)	-27(4)

Supplementary Table 10. Bond Lengths for TPAOMe-TDPP.

tom	Atom	Length/Å	Atom	Atom	Length/Å
S01	C00I	1.728(3)	C00P	C00Y	1.388(4)
S01	C01D	1.738(3)	C00P	C00Z	1.401(4)
S02	C017	1.725(3)	C00Q	C016	1.370(4)
S02	C01N	1.733(3)	C00R	C015	1.390(4)
O003	C00E	1.230(3)	C00R	C01E	1.392(4)
O004	C00O	1.230(4)	C00S	C00S2	1.406(5)
O005	C01L	1.374(3)	C00S	C012	1.404(4)
O005	C01X	1.423(5)	C00T	C00W	1.396(4)
N006	C00K	1.429(3)	C00T	C016	1.402(4)
N006	C00M	1.437(3)	C00U	C01G	1.372(4)
N006	C00T	1.403(3)	C00V	C01A	1.392(4)
O007	C010	1.372(3)	C00X	C013	1.398(4)
O007	C01V	1.430(4)	C00X	C01D	1.365(4)
N008	C00E	1.417(3)	C00Y	C011	1.376(4)
N008	C00L	1.388(3)	C010	C01A	1.382(4)
N008	C01C	1.464(3)	C010	C01K	1.382(4)
N009	C00F	1.426(3)	C012	C01N	1.436(4)
N009	C00P	1.403(3)	C014	C018	1.377(4)
N009	C00R	1.429(3)	C015	C01I	1.389(4)
O00A	C01J	1.372(4)	C017	C01S	1.368(4)
O00A	C01Z	1.443(4)	C018	C01O	1.382(4)
N00B	C00O	1.425(3)	C019	C01M	1.386(4)
N00B	C012	1.386(4)	C01B	C01H	1.385(4)
N00B	C01P	1.460(4)	C01C	C01T	1.540(5)

O00C	C01O	1.368(3)	C01E	C01Q	1.371(4)
O00C	C01W	1.413(4)	C01F	C01K	1.385(4)
C00D	C00I	1.458(3)	C01G	C01J	1.386(4)
C00D	C00J	1.405(4)	C01H	C01O	1.389(4)
C00D	C00Q	1.397(4)	C01I	C01L	1.378(4)
C00E	C00G1	1.441(4)	C01J	C01M	1.381(4)
C00F	C00U	1.394(4)	C01L	C01Q	1.398(4)
C00F	C019	1.381(4)	C01N	C01R	1.376(4)
C00G	C00G1	1.422(5)	C01P	C01U	1.534(4)
C00G	C00L	1.394(4)	C01R	C01S	1.397(4)
C00H	C00N	1.395(4)	C01T	C021	1.466(5)
C00H	C00Z	1.384(4)	C01T	C022	1.549(5)
C00I	C013	1.374(4)	C01U	C020	1.519(5)
C00J	C00W	1.388(4)	C01U	C023	1.527(5)
C00K	C014	1.397(4)	C01Y	C021	1.504(5)
C00K	C01B	1.378(4)	C020	C024	1.521(5)
C00L	C01D	1.446(3)	C022	C026	1.491(6)
C00M	C00V	1.376(4)	C023	C025	1.456(7)
C00M	C01F	1.392(4)	C025	C028	1.516(6)
C00N	C011	1.403(4)	C026	C0AA	1.382(7)
C00N	C017	1.461(4)	C028	C02A	1.531(8)
C00O	C00S2	1.437(4)	C1	C0AA	1.542(8)

Supplementary Table 11. Bond Angles for TPAOMe-TDPP.

Atom	Atom	Atom	Angle/˚	Atom	Atom	Atom	Angle/˚
C00I	S01	C01D	92.78(12)	C00M	C00V	C01A	121.0(3)
C017	S02	C01N	92.79(14)	C00J	C00W	C00T	121.2(2)
C01L	O005	C01X	116.7(3)	C01D	C00X	C013	114.3(3)
C00K	N006	C00M	117.15(19)	C011	C00Y	C00P	120.7(3)
C00T	N006	C00K	120.3(2)	C00H	C00Z	C00P	120.4(3)
C00T	N006	C00M	119.3(2)	O007	C010	C01A	124.5(3)
C010	O007	C01V	118.0(3)	O007	C010	C01K	115.0(3)
C00E	N008	C01C	119.6(2)	C01K	C010	C01A	120.5(3)
C00L	N008	C00E	111.5(2)	C00Y	C011	C00N	121.7(3)
C00L	N008	C01C	128.7(2)	N00B	C012	C00S	106.7(2)
C00F	N009	C00R	117.9(2)	N00B	C012	C01N	126.8(2)
C00P	N009	C00F	119.3(2)	C00S	C012	C01N	126.4(3)
C00P	N009	C00R	120.9(2)	C00I	C013	C00X	113.6(3)
C01J	O00A	C01Z	116.3(3)	C018	C014	C00K	120.0(3)
C00O	N00B	C01P	119.7(2)	C01I	C015	C00R	120.1(3)

C012	N00B	C00O	111.5(2)	C00Q	C016	C00T	121.0(2)
C012	N00B	C01P	128.6(2)	C00N	C017	S02	121.0(2)
C01O	O00C	C01W	118.1(3)	C01S	C017	S02	110.3(2)
C00J	C00D	C00I	123.1(2)	C01S	C017	C00N	128.7(3)
C00Q	C00D	C00I	119.9(2)	C014	C018	C01O	121.1(3)
C00Q	C00D	C00J	117.0(2)	C00F	C019	C01M	120.8(3)
O003	C00E	N008	122.5(2)	C010	C01A	C00V	119.1(3)
O003	C00E	C00G1	132.7(2)	C00K	C01B	C01H	121.6(3)
N008	C00E	C00G1	104.8(2)	N008	C01C	C01T	114.2(2)
C00U	C00F	N009	120.6(2)	C00L	C01D	S01	127.0(2)
C019	C00F	N009	120.3(3)	C00X	C01D	S01	109.51(19)
C019	C00F	C00U	119.1(3)	C00X	C01D	C00L	123.5(2)
C00G1	C00G	C00E1	107.6(3)	C01Q	C01E	C00R	121.1(3)
C00L	C00G	C00E1	143.2(2)	C01K	C01F	C00M	120.1(3)
C00L	C00G	C00G1	109.2(3)	C00U	C01G	C01J	120.2(3)
C00Z	C00H	C00N	121.5(3)	C01B	C01H	C01O	119.5(3)
C00D	C00I	S01	122.4(2)	C01L	C01I	C015	120.3(3)
C013	C00I	S01	109.82(19)	O00A	C01J	C01G	115.3(3)
C013	C00I	C00D	127.8(2)	O00A	C01J	C01M	124.7(3)
C00W	C00J	C00D	121.1(2)	C01M	C01J	C01G	120.0(3)
C014	C00K	N006	120.3(2)	C010	C01K	C01F	119.9(3)
C01B	C00K	N006	121.2(2)	O005	C01L	C01I	125.0(3)
C01B	C00K	C014	118.5(2)	O005	C01L	C01Q	115.2(3)
N008	C00L	C00G	106.8(2)	C01I	C01L	C01Q	119.8(3)
N008	C00L	C01D	127.0(2)	C01J	C01M	C019	119.5(3)
C00G	C00L	C01D	126.2(2)	C012	C01N	S02	126.8(2)
C00V	C00M	N006	120.4(2)	C01R	C01N	S02	109.3(2)
C00V	C00M	C01F	119.3(2)	C01R	C01N	C012	123.9(3)
C01F	C00M	N006	120.2(2)	O00C	C01O	C018	115.9(3)
C00H	C00N	C011	117.2(2)	O00C	C01O	C01H	124.9(3)
C00H	C00N	C017	122.8(3)	C018	C01O	C01H	119.2(3)
C011	C00N	C017	120.1(3)	N00B	C01P	C01U	114.4(2)
O004	C00O	N00B	122.3(3)	C01E	C01Q	C01L	119.6(3)
O004	C00O	C00S2	133.8(2)	C01N	C01R	C01S	114.1(3)
N00B	C00O	C00S2	104.0(2)	C017	C01S	C01R	113.5(3)

C00Y	C00P	N009	121.7(2)	C01C	C01T	C022	107.3(3)
C00Y	C00P	C00Z	118.4(2)	C021	C01T	C01C	116.3(3)
C00Z	C00P	N009	119.9(2)	C021	C01T	C022	113.7(3)
C016	C00Q	C00D	122.1(2)	C020	C01U	C01P	112.2(3)
C015	C00R	N009	120.8(2)	C020	C01U	C023	112.3(4)
C015	C00R	C01E	119.0(3)	C023	C01U	C01P	109.3(3)
C01E	C00R	N009	120.2(2)	C01U	C020	C024	115.7(3)
C00S2	C00S	C00O2	108.9(3)	C01T	C021	C01Y	115.5(4)
C012	C00S	C00O2	142.3(3)	C026	C022	C01T	115.3(4)
C012	C00S	C00S2	108.7(3)	C025	C023	C01U	117.2(4)
C00W	C00T	N006	121.6(2)	C023	C025	C028	114.9(5)
C00W	C00T	C016	117.6(2)	C0AA	C026	C022	126.4(6)
C016	C00T	N006	120.9(2)	C025	C028	C02A	113.4(5)
C01G	C00U	C00F	120.3(3)	C026	C0AA	C1	114.1(6)

Supplementary Table 12. Hydrogen Atom Coordinates (Å×10^4) and Isotropic Displacement Parameters (Å^2×10^3) for **TPAOMe-TDPP.**

Atom	x	y	z	U(eq)
H00H	3146.32	5805.22	350.82	48
H00J	7760.68	5804.89	4494.86	46
H00Q	8970.03	6524.62	5837.73	48
H00U	1179.82	7370.34	490.59	53
H00V	2188.44	6640.3	4730.94	49
H00W	5628.34	6171.7	4459.61	46
H00X	13003.31	5651.29	5776.68	54
H00Y	1199.83	6898.85	-963.57	52
H00Z	1046.94	6178.87	372.96	50
H011	3284.89	6523.35	-985.35	54
H013	10937.46	6078.5	5928.27	54
H014	4368.19	7848.13	5203.63	49
H015	-1188.52	7826.76	-410.58	52
H016	6858.39	6885.96	5805.15	49
H018	3865.97	8304.04	5898.69	57
H019	-2499.71	6694.51	150.77	54
H01A	910.12	6844.79	3944.54	53
H01B	4278.9	6487.77	5970.7	52
H01C	11282.34	4837.16	4153.25	51
H01D	12229.79	4519.61	3844.93	51

H01Q	-1328.36	6419.28	-1116.79	58
H01F	5464.32	7320.12	4308.49	52
H01R	721.78	7589.75	1285.12	60
H01H	3763.8	6944.18	6667.44	57
H01S	-2520.75	8188.61	-1123.65	58
H01K	4216	7500.2	3513.36	58
H01U	-2981.79	6931.29	943.85	58
H01V	8173.14	4672.65	1185.15	57
H01W	6954.09	4937.44	826.35	57
H01X	-2634.41	6778.18	-1826.82	61
H01Y	7397.03	5614.22	-851.33	67
H01Z	5187.09	6026.54	-1054.84	68
H01T	11653.68	5612.35	3753.91	96
H01	7729.3	5779.96	1117.52	78
H01E	107.72	7259.01	2676.55	112
H01G	-136.65	7304.41	3243.31	112
H01I	382.15	6770.27	3042.78	112
H01J	2470.58	7485.62	7097.07	115
H01L	2915.32	7997.52	7414.85	115
H01M	3989.85	7556.12	7358.77	115
H1AA	-4235.19	8334.61	-2360.23	118
H	-2854.45	8439.18	-1996.88	118
HA	-4202.67	8369.19	-1769.18	118
H01N	12194.68	5932.53	2831.16	108
H01O	13784.6	5945.48	2871.72	108
H01P	13083.66	6232.52	3283.95	108
H2AA	-3357.03	7486.91	1602.24	123
HB	-2634.65	7425.01	2169.08	123
HC	-2778	6934.78	1802.1	123
H02G	10056.63	5556.84	1342.79	92
H02H	9720.13	5273.57	1830.92	92
H02A	13362.74	5140.78	3163.33	91
H02B	13906.46	5469.25	3653.28	91
H02C	10709.77	5344.25	2882.87	118

H02D	10821.95	4763.05	3089.48	118
H02I	7145	5063.34	1863.27	108
H02J	7247.68	5670.52	1975.69	108
H02K	9510.78	6374.23	1662.5	130
H02L	10629.46	6097.27	2059.22	130
H02M	9106.03	6097.01	2144.39	130
H02N	5323.1	5213.54	1234.24	123
H02O	5419.12	5819.8	1357.37	123
H02E	9365.01	4951.91	3639.47	145
H02F	9302	5541	3471.83	145
H02P	4880.5	5643.82	2173.09	154
H02Q	4736.91	5041.19	2033.87	154
H02R	2894.24	5284.49	1362.43	218
H02S	2955.85	5851.73	1605.13	218
H02T	2561.15	5362.87	1914.56	218
H1A	6053.09	5270.27	3034.24	226
H1B	7018.71	5635.6	3409.72	226
H1C	6750.05	5050.67	3564.75	226
H0AA	7995.11	4716.02	2975.39	201
H0AB	8071.68	5272.73	2721.8	201

2.6. X-ray crystallographic data for Py-TDPP

Supplementary Table 13. Crystal data and structure refinement for Py-TDPP.

Identification code	**Py-TDPP**
Empirical formula	$C_{63}H_{58}Cl_2N_2O_2S_2$
Formula weight	1010.13
Temperature/K	99.99(10)
Crystal system	triclinic
Space group	P-1
a/Å	10.3358(6)

b/Å	13.2950(9)
c/Å	18.9731(13)
α/°	80.781(6)
β/°	80.217(5)
γ/°	85.950(5)
Volume/Å^3	2533.6(3)
Z	2
ρcalcg/cm^3	1.324
μ/mm^{-1}	2.295
F(000)	1064.0
Crystal size/mm^3	0.12 × 0.11 × 0.1
Radiation	CuKα (λ = 1.54178)
2Θ range for data collection/°	7.656 to 150.006
Index ranges	$-12 \le h \le 12$, $-8 \le k \le 16$, $-23 \le l \le 23$
Reflections collected	15267
Independent reflections	9842 [Rint = 0.0571, Rsigma = 0.0982]
Data/restraints/parameters	9842/22/654
Goodness-of-fit on F^2	0.999
Final R indexes [I>=2σ (I)]	R1 = 0.0778, wR2 = 0.2030
Final R indexes [all data]	R1 = 0.1124, wR2 = 0.2359
Largest diff. peak/hole / e Å^3	0.57/-0.78

Supplementary Table 14. Fractional Atomic Coordinates (×10^4) and Equivalent Isotropic Displacement Parameters (Å^2×10^3) for **Py-TDPP**. Ueqis defined as 1/3 of of the trace of the orthogonalised UIJtensor.

Atom	x	y	z	U(eq)
Cl1	1956(5)	6250(4)	9492(2)	178(2)
Cl2	4714(3)	6395(3)	9587.6(18)	135.0(15)
C63	3281(10)	7033(8)	9391(6)	104(3)
S1	10333.8(8)	9496.9(7)	6410.4(5)	27.4(2)
S2	6490.0(8)	6345.7(7)	3419.7(5)	29.3(2)
O1	11093(2)	7620(2)	4075.0(15)	31.1(6)
O2	5760(2)	8586(2)	5557.8(15)	32.8(6)
N1	8992(3)	7238(2)	3949.3(16)	26.2(6)
N2	7864(3)	8840(2)	5748.9(16)	26.1(6)
C1	9899(3)	7681(3)	4280.7(19)	26.7(7)
C2	9093(3)	8148(3)	4847.1(19)	24.5(7)
C3	9154(3)	8668(2)	5417.6(19)	24.9(7)
C4	6953(3)	8459(3)	5389(2)	27.3(7)

C5	7765(3)	7980(3)	4831(2)	26.8(7)
C6	7702(3)	7395(3)	4285.3(19)	26.4(7)
C7	9440(3)	6738(3)	3316.5(19)	26.5(7)
C8	9203(3)	7404(3)	2605(2)	28.4(7)
C9	9263(4)	6717(3)	2025(2)	34.0(8)
C10	8743(5)	7239(4)	1345(3)	45.4(10)
C11	8592(8)	6443(6)	852(4)	85(2)
C12	7988(9)	6906(6)	193(4)	91(2)
C13	10127(4)	8303(3)	2384(2)	31.6(8)
C14	11573(4)	8007(3)	2170(3)	42.6(10)
C15	10313(3)	8964(3)	5637.1(19)	25.1(7)
C16	11590(3)	8815(3)	5293.9(19)	26.2(7)
C17	12541(3)	9108(3)	5653.0(19)	25.8(7)
C18	12024(3)	9478(3)	6286.3(19)	26.4(7)
C19	12735(3)	9872(2)	6785(2)	25.6(7)
C20	12389(3)	9692(3)	7546(2)	26.8(7)
C21	11359(4)	9033(3)	7931(2)	32.8(8)
C22	11039(4)	8900(3)	8663(2)	37.7(9)
C23	11732(4)	9377(3)	9100(2)	38.1(9)
C24	11427(5)	9235(4)	9860(2)	47.8(11)
C25	12155(5)	9681(4)	10264(3)	49.8(11)
C26	13199(5)	10266(4)	9929(2)	44.1(10)
C27	13540(4)	10438(3)	9173(2)	35.9(8)
C28	14603(4)	11065(3)	8804(2)	37.7(9)
C29	14907(4)	11216(3)	8083(2)	35.6(8)
C30	14193(3)	10753(3)	7637(2)	30.2(7)
C31	14528(3)	10893(3)	6891(2)	31.0(8)
C32	13820(3)	10456(3)	6467(2)	28.9(7)
C33	13117(4)	10142(3)	7979(2)	28.6(7)
C34	12798(4)	9983(3)	8746(2)	32.6(8)
C35	7397(3)	9254(3)	6418.0(19)	26.8(7)
C36	7520(3)	8465(3)	7096(2)	29.3(7)
C37	6599(4)	7588(3)	7184(2)	31.4(8)
C38	5129(4)	7865(3)	7354(3)	39.5(9)
C39	4298(5)	6949(4)	7390(4)	60.9(15)
C40	4375(5)	6580(4)	6663(4)	67.0(17)
C41	7359(4)	9015(3)	7759(2)	35.8(8)
C42	7578(5)	8303(3)	8452(2)	43.7(10)
C43	6541(3)	7006(3)	4131(2)	27.9(7)
C44	5287(3)	7154(3)	4496(2)	29.9(7)

C45	4307(4)	6780(3)	4186(2)	31.6(8)
C46	4791(3)	6322(3)	3586(2)	29.0(7)
C47	4140(3)	5917(3)	3068(2)	28.7(7)
C48	2986(3)	5349(3)	3294(2)	26.5(7)
C49	2435(4)	5036(3)	4035(2)	31.5(8)
C50	1325(4)	4495(3)	4218(2)	33.1(8)
C51	683(4)	4206(3)	3683(2)	33.2(8)
C52	-467(4)	3646(3)	3867(3)	40.8(10)
C53	-1039(4)	3350(3)	3320(3)	46.9(11)
C54	-520(4)	3600(3)	2610(3)	44.7(11)
C55	622(4)	4154(3)	2392(3)	36.0(9)
C56	1203(5)	4412(3)	1657(3)	42.6(10)
C57	2313(4)	4920(3)	1462(2)	39.0(9)
C58	2935(4)	5253(3)	2000(2)	32.7(8)
C59	4105(4)	5791(3)	1810(2)	34.9(8)
C60	4674(4)	6108(3)	2337(2)	31.4(8)
C61	1218(4)	4469(3)	2950(2)	30.8(8)
C62	2392(3)	5031(3)	2743(2)	29.1(7)
Cl2A	4304(14)	7297(14)	9864(8)	135.0(15)
C63A	2850(30)	6760(16)	9853(17)	104(3)
Cl1A	3290(20)	5752(15)	9324(11)	178(2)

Supplementary Table 15. Anisotropic Displacement Parameters ($Å^2×10^3$) for **Py-TDPP**. The Anisotropic displacement factor exponent takes the form: $-2\pi^2[h^2a^{*2}U11+2hka^*b^*U12+…]$.

Atom	U11	U22	U33	U23	U13	U12
Cl1	187(4)	167(4)	181(4)	88(3)	-104(4)	-88(3)
Cl2	82.3(17)	173(3)	116(2)	39(2)	8.5(14)	30.7(18)
C63	93(7)	96(7)	100(7)	25(6)	1(5)	21(5)
S1	19.1(4)	29.3(4)	35.8(5)	-8.5(3)	-5.8(3)	-2.5(3)
S2	19.7(4)	30.3(4)	39.8(5)	-10.3(3)	-3.9(3)	-5.0(3)
O1	18.1(11)	37.1(14)	40.0(14)	-11.2(11)	-4.2(10)	-3.4(10)
O2	18.8(12)	41.5(15)	40.3(14)	-12.7(11)	-5.1(10)	0.1(10)
N1	20.3(13)	27.9(14)	31.4(15)	-6.9(11)	-3.5(11)	-3.8(11)
N2	18.9(13)	30.1(15)	30.2(15)	-6.5(11)	-3.8(11)	-2.2(11)
C1	20.5(16)	27.3(17)	32.7(17)	-4.0(13)	-3.4(13)	-6.1(13)
C2	16.6(15)	23.6(15)	33.4(17)	-2.1(13)	-5.5(12)	-4.4(12)
C3	20.8(15)	20.0(15)	33.0(17)	0.0(13)	-5.0(13)	-2.0(12)
C4	19.1(15)	28.2(17)	34.6(18)	-3.3(14)	-5.0(13)	-1.7(13)
C5	16.5(15)	28.0(17)	35.4(18)	-3.9(14)	-3.3(12)	-1.8(12)
C6	19.4(15)	24.5(16)	34.2(18)	0.0(13)	-4.8(13)	-2.6(12)

C7	22.6(15)	24.2(16)	33.6(18)	-6.5(13)	-4.4(13)	-3.0(13)
C8	23.0(16)	25.1(16)	37.8(19)	-6.7(14)	-4.4(13)	-1.8(13)
C9	30.2(18)	33.6(19)	40(2)	-8.5(15)	-8.3(15)	0.8(15)
C10	47(2)	48(2)	45(2)	-10.4(19)	-18.9(19)	6.9(19)
C11	105(5)	97(5)	63(4)	-39(3)	-41(4)	45(4)
C12	128(7)	85(5)	67(4)	-22(4)	-36(4)	17(5)
C13	29.9(18)	26.2(17)	37.9(19)	-2.7(14)	-5.2(14)	-1.9(14)
C14	28.8(19)	37(2)	59(3)	-0.7(19)	-4.2(18)	-2.8(16)
C15	22.8(16)	21.6(15)	31.8(17)	-5.8(12)	-5.6(13)	-0.2(12)
C16	22.3(16)	23.8(16)	32.2(17)	-2.5(13)	-4.0(13)	-4.8(12)
C17	20.8(15)	22.5(16)	34.4(18)	-2.9(13)	-5.3(13)	-3.4(12)
C18	23.3(16)	22.1(15)	33.5(18)	0.5(13)	-7.0(13)	-3.3(12)
C19	20.4(15)	20.4(15)	38.0(18)	-4.1(13)	-11.3(13)	0.1(12)
C20	22.7(16)	20.0(15)	39.1(19)	-4.3(13)	-9.4(14)	0.7(12)
C21	30.5(18)	33.0(19)	35.9(19)	-3.3(15)	-7.5(15)	-8.0(15)
C22	35(2)	37(2)	40(2)	1.6(16)	-6.9(16)	-7.2(16)
C23	35(2)	38(2)	40(2)	-2.3(16)	-5.4(16)	-2.6(16)
C24	48(3)	55(3)	39(2)	-4.4(19)	-3.3(19)	-9(2)
C25	57(3)	56(3)	36(2)	-6.5(19)	-9.6(19)	4(2)
C26	47(2)	50(2)	42(2)	-15.7(19)	-17.6(18)	5.5(19)
C27	36(2)	34.1(19)	42(2)	-12.6(16)	-15.3(16)	4.6(16)
C28	29.9(19)	32.8(19)	57(3)	-16.2(17)	-18.6(17)	0.5(15)
C29	26.7(18)	28.0(18)	57(2)	-12.5(16)	-15.5(16)	0.7(14)
C30	20.3(16)	25.9(17)	47(2)	-8.7(15)	-9.8(14)	2.4(13)
C31	21.9(16)	23.6(16)	49(2)	-5.8(15)	-9.8(14)	-0.3(13)
C32	21.6(16)	29.0(17)	37.7(19)	-7.0(14)	-6.9(13)	-1.8(13)
C33	25.3(16)	23.4(16)	38.3(19)	-6.6(13)	-8.2(14)	2.7(13)
C34	26.5(17)	29.3(18)	43(2)	-6.4(15)	-10.0(15)	2.4(14)
C35	23.3(16)	25.5(16)	32.2(18)	-5.6(13)	-5.1(13)	-1.0(13)
C36	25.2(16)	29.3(17)	33.4(18)	-6.5(14)	-3.8(13)	0.8(14)
C37	24.5(17)	28.4(18)	40(2)	-3.3(14)	-5.1(14)	0.5(14)
C38	27.6(19)	29.8(19)	54(2)	3.9(17)	3.4(16)	-3.8(15)
C39	31(2)	47(3)	100(4)	12(3)	-13(2)	-10(2)
C40	35(2)	40(3)	129(6)	-12(3)	-22(3)	-2(2)
C41	42(2)	28.5(18)	36(2)	-8.7(15)	-1.0(16)	1.3(15)
C42	54(3)	42(2)	35(2)	-6.5(17)	-5.2(18)	-5.0(19)
C43	24.0(17)	23.1(16)	38.2(19)	-6.0(14)	-7.3(14)	-2.2(13)
C44	20.9(16)	30.3(18)	37.9(19)	-3.7(14)	-2.0(13)	-6.2(13)
C45	24.4(17)	34.0(19)	36.2(19)	-4.0(15)	-4.3(14)	-4.6(14)
C46	21.9(16)	25.5(16)	38.6(19)	-1.5(14)	-3.4(13)	-6.1(13)

C47	21.2(16)	25.6(17)	39.5(19)	-2.7(14)	-5.6(14)	-5.1(13)
C48	20.3(15)	22.6(16)	36.7(18)	-4.8(13)	-3.5(13)	-2.5(12)
C49	28.6(18)	24.2(17)	42(2)	-3.4(14)	-7.6(15)	-5.5(14)
C50	27.0(17)	26.8(17)	43(2)	-0.7(15)	-1.4(15)	-3.2(14)
C51	24.5(17)	20.2(16)	55(2)	-0.5(15)	-9.8(15)	-3.4(13)
C52	28.4(19)	30.5(19)	62(3)	4.8(18)	-10.6(17)	-8.4(15)
C53	27.3(19)	28.6(19)	85(3)	4(2)	-18(2)	-11.2(16)
C54	36(2)	28.3(19)	75(3)	-1.5(19)	-26(2)	-7.7(16)
C55	28.2(18)	23.3(17)	61(3)	-8.6(16)	-17.3(17)	-0.2(14)
C56	45(2)	32(2)	60(3)	-14.5(18)	-27(2)	2.9(17)
C57	40(2)	37(2)	45(2)	-14.5(17)	-16.1(17)	5.1(17)
C58	30.3(18)	27.3(17)	43(2)	-10.1(15)	-10.4(15)	1.6(14)
C59	33.3(19)	36(2)	36.1(19)	-9.5(15)	-2.9(15)	-1.4(15)
C60	24.6(16)	29.5(18)	39(2)	-4.8(14)	-1.6(14)	-5.8(14)
C61	24.7(17)	16.7(15)	53(2)	-4.0(14)	-11.3(15)	-2.0(12)
C62	23.5(16)	21.9(16)	44(2)	-9.0(14)	-7.4(14)	0.2(13)
Cl2A	82.3(17)	173(3)	116(2)	39(2)	8.5(14)	30.7(18)
C63A	93(7)	96(7)	100(7)	25(6)	1(5)	21(5)
Cl1A	187(4)	167(4)	181(4)	88(3)	-104(4)	-88(3)

Supplementary Table 16. Bond Lengths for **Py-TDPP**.

tom	Atom	Length/Å	Atom	Atom	Length/Å
Cl1	C63	1.745(11)	C26	C27	1.402(6)
Cl2	C63	1.721(10)	C27	C28	1.439(6)
S1	C15	1.732(4)	C27	C34	1.430(5)
S1	C18	1.722(4)	C28	C29	1.335(6)
S2	C43	1.734(4)	C29	C30	1.444(5)
S2	C46	1.732(4)	C30	C31	1.385(6)
O1	C1	1.231(4)	C30	C33	1.425(5)
O2	C4	1.227(4)	C31	C32	1.389(5)
N1	C1	1.420(4)	C33	C34	1.421(6)
N1	C6	1.394(4)	C35	C36	1.541(5)
N1	C7	1.457(5)	C36	C37	1.528(5)
N2	C3	1.394(4)	C36	C41	1.534(5)
N2	C4	1.417(5)	C37	C38	1.529(5)
N2	C35	1.458(5)	C38	C39	1.525(6)
C1	C2	1.438(5)	C39	C40	1.524(9)
C2	C3	1.387(5)	C41	C42	1.532(6)
C2	C5	1.413(4)	C43	C44	1.379(5)
C3	C15	1.432(5)	C44	C45	1.410(5)
C4	C5	1.439(5)	C45	C46	1.378(6)

C5	C6	1.402(5)	C46	C47	1.468(5)
C6	C43	1.431(5)	C47	C48	1.424(5)
C7	C8	1.539(5)	C47	C60	1.393(5)
C8	C9	1.528(5)	C48	C49	1.430(5)
C8	C13	1.543(5)	C48	C62	1.431(5)
C9	C10	1.528(6)	C49	C50	1.362(5)
C10	C11	1.550(8)	C50	C51	1.416(6)
C11	C12	1.516(9)	C51	C52	1.410(5)
C13	C14	1.522(5)	C51	C61	1.405(6)
C15	C16	1.385(5)	C52	C53	1.397(7)
C16	C17	1.395(5)	C53	C54	1.361(7)
C17	C18	1.380(5)	C54	C55	1.402(6)
C18	C19	1.469(5)	C55	C56	1.419(7)
C19	C20	1.414(5)	C55	C61	1.442(6)
C19	C32	1.402(5)	C56	C57	1.340(6)
C20	C21	1.448(5)	C57	C58	1.435(6)
C20	C33	1.425(5)	C58	C59	1.412(5)
C21	C22	1.358(6)	C58	C62	1.418(6)
C22	C23	1.426(6)	C59	C60	1.376(6)
C23	C24	1.408(6)	C61	C62	1.434(5)
C23	C34	1.420(5)	Cl2A	C63A	1.71(2)
C24	C25	1.382(7)	C63A	Cl1A	1.79(2)
C25	C26	1.378(7)			

Supplementary Table 17. Bond Angles for **Py-TDPP**.

Atom	Atom	Atom	Angle/˚	Atom	Atom	Atom	Angle/˚
Cl2	C63	Cl1	114.1(6)	C29	C28	C27	121.6(4)
C18	S1	C15	93.16(17)	C28	C29	C30	121.6(4)
C46	S2	C43	93.15(18)	C31	C30	C29	121.5(3)
C1	N1	C7	120.8(3)	C31	C30	C33	119.8(3)
C6	N1	C1	111.5(3)	C33	C30	C29	118.7(4)
C6	N1	C7	127.6(3)	C30	C31	C32	121.0(3)
C3	N2	C4	111.4(3)	C31	C32	C19	120.7(4)
C3	N2	C35	128.7(3)	C30	C33	C20	119.4(3)
C4	N2	C35	119.6(3)	C34	C33	C20	121.2(3)
O1	C1	N1	122.3(3)	C34	C33	C30	119.4(3)
O1	C1	C2	133.1(3)	C23	C34	C27	119.0(4)
N1	C1	C2	104.6(3)	C23	C34	C33	120.5(4)
C3	C2	C1	142.7(3)	C33	C34	C27	120.5(4)
C3	C2	C5	109.0(3)	N2	C35	C36	112.4(3)
C5	C2	C1	108.2(3)	C37	C36	C35	112.6(3)

N2	C3	C15	126.0(3)	C37	C36	C41	113.7(3)
C2	C3	N2	106.9(3)	C41	C36	C35	109.2(3)
C2	C3	C15	127.1(3)	C36	C37	C38	116.1(3)
O2	C4	N2	122.6(3)	C39	C38	C37	111.8(4)
O2	C4	C5	133.2(3)	C40	C39	C38	113.4(4)
N2	C4	C5	104.1(3)	C42	C41	C36	113.1(3)
C2	C5	C4	108.5(3)	C6	C43	S2	124.9(3)
C6	C5	C2	109.3(3)	C44	C43	S2	109.8(3)
C6	C5	C4	142.3(3)	C44	C43	C6	125.2(4)
N1	C6	C5	106.3(3)	C43	C44	C45	113.5(4)
N1	C6	C43	127.3(3)	C46	C45	C44	113.8(3)
C5	C6	C43	126.3(3)	C45	C46	S2	109.7(3)
N1	C7	C8	113.0(3)	C45	C46	C47	132.2(3)
C7	C8	C13	112.5(3)	C47	C46	S2	118.0(3)
C9	C8	C7	108.7(3)	C48	C47	C46	121.8(3)
C9	C8	C13	114.1(3)	C60	C47	C46	118.3(3)
C10	C9	C8	113.8(3)	C60	C47	C48	119.9(3)
C9	C10	C11	110.3(4)	C47	C48	C49	123.9(3)
C12	C11	C10	112.7(6)	C47	C48	C62	117.3(3)
C14	C13	C8	115.3(3)	C49	C48	C62	118.6(3)
C3	C15	S1	124.8(3)	C50	C49	C48	121.2(4)
C16	C15	S1	109.3(3)	C49	C50	C51	121.4(4)
C16	C15	C3	125.7(3)	C52	C51	C50	121.9(4)
C15	C16	C17	113.8(3)	C61	C51	C50	119.1(3)
C18	C17	C16	113.6(3)	C61	C51	C52	119.0(4)
C17	C18	S1	110.0(3)	C53	C52	C51	119.7(4)
C17	C18	C19	127.9(3)	C54	C53	C52	121.4(4)
C19	C18	S1	122.0(3)	C53	C54	C55	121.7(4)
C20	C19	C18	123.9(3)	C54	C55	C56	123.1(4)
C32	C19	C18	116.4(3)	C54	C55	C61	117.4(4)
C32	C19	C20	119.7(3)	C56	C55	C61	119.4(4)
C19	C20	C21	124.2(3)	C57	C56	C55	122.1(4)
C19	C20	C33	119.4(3)	C56	C57	C58	120.3(4)
C33	C20	C21	116.4(3)	C59	C58	C57	121.5(4)
C22	C21	C20	122.2(4)	C59	C58	C62	118.3(4)
C21	C22	C23	121.7(4)	C62	C58	C57	120.2(4)
C24	C23	C22	122.5(4)	C60	C59	C58	120.2(4)
C24	C23	C34	119.6(4)	C59	C60	C47	122.4(3)
C34	C23	C22	117.9(4)	C51	C61	C55	120.7(3)
C25	C24	C23	120.6(4)	C51	C61	C62	120.6(4)

C26	C25	C24	120.5(4)	C62	C61	C55	118.6(4)
C25	C26	C27	121.4(4)	C48	C62	C61	118.9(3)
C26	C27	C28	122.9(4)	C58	C62	C48	121.8(3)
C26	C27	C34	119.0(4)	C58	C62	C61	119.3(4)
C34	C27	C28	118.1(4)	Cl2A	C63A	Cl1A	104.9(17)

Supplementary Table 18. Torsion Angles for **Py-TDPP**.

A	B	C	D	Angle/˚	A	B	C	D	Angle/˚
S1	C15	C16	C17	0.9(4)	C24	C23	C34	C33	-179.6(4)
S1	C18	C19	C20	42.6(4)	C24	C25	C26	C27	0.9(8)
S1	C18	C19	C32	-137.7(3)	C25	C26	C27	C28	178.5(4)
S2	C43	C44	C45	-2.5(4)	C25	C26	C27	C34	-0.9(7)
S2	C46	C47	C48	144.6(3)	C26	C27	C28	C29	180.0(4)
S2	C46	C47	C60	-35.9(4)	C26	C27	C34	C23	0.5(6)
O1	C1	C2	C3	-3.7(8)	C26	C27	C34	C33	180.0(4)
O1	C1	C2	C5	179.7(4)	C27	C28	C29	C30	-0.5(6)
O2	C4	C5	C2	174.4(4)	C28	C27	C34	C23	-178.9(4)
O2	C4	C5	C6	-6.8(8)	C28	C27	C34	C33	0.6(6)
N1	C1	C2	C3	176.1(4)	C28	C29	C30	C31	-178.6(4)
N1	C1	C2	C5	-0.5(4)	C28	C29	C30	C33	1.7(6)
N1	C6	C43	S2	-6.8(5)	C29	C30	C31	C32	-179.0(3)
N1	C6	C43	C44	177.7(3)	C29	C30	C33	C20	179.1(3)
N1	C7	C8	C9	-161.2(3)	C29	C30	C33	C34	-1.7(5)
N1	C7	C8	C13	71.5(4)	C30	C31	C32	C19	0.8(5)
N2	C3	C15	S1	6.1(5)	C30	C33	C34	C23	-179.9(3)
N2	C3	C15	C16	-178.1(3)	C30	C33	C34	C27	0.6(5)
N2	C4	C5	C2	-3.8(4)	C31	C30	C33	C20	-0.7(5)
N2	C4	C5	C6	175.0(5)	C31	C30	C33	C34	178.6(3)
N2	C35	C36	C37	-67.3(4)	C32	C19	C20	C21	-174.7(3)
N2	C35	C36	C41	165.4(3)	C32	C19	C20	C33	2.6(5)
C1	N1	C6	C5	1.9(4)	C33	C20	C21	C22	4.3(6)
C1	N1	C6	C43	-175.6(3)	C33	C30	C31	C32	0.8(5)
C1	N1	C7	C8	-101.7(4)	C34	C23	C24	C25	0.0(7)
C1	C2	C3	N2	-177.6(4)	C34	C27	C28	C29	-0.7(6)
C1	C2	C3	C15	1.6(7)	C35	N2	C3	C2	172.5(3)
C1	C2	C5	C4	-179.1(3)	C35	N2	C3	C15	-6.8(6)
C1	C2	C5	C6	1.7(4)	C35	N2	C4	O2	10.3(5)
C2	C3	C15	S1	-173.0(3)	C35	N2	C4	C5	-171.3(3)
C2	C3	C15	C16	2.8(6)	C35	C36	C37	C38	-67.4(4)
C2	C5	C6	N1	-2.2(4)	C35	C36	C41	C42	-175.3(3)

C2	C5	C6	C43	175.3(3)	C36	C37	C38	C39	176.9(4)
C3	N2	C4	O2	-175.2(3)	C37	C36	C41	C42	58.0(5)
C3	N2	C4	C5	3.3(4)	C37	C38	C39	C40	-66.9(5)
C3	N2	C35	C36	-77.0(4)	C41	C36	C37	C38	57.5(5)
C3	C2	C5	C4	3.1(4)	C43	S2	C46	C45	-2.0(3)
C3	C2	C5	C6	-176.1(3)	C43	S2	C46	C47	173.6(3)
C3	C15	C16	C17	-175.4(3)	C43	C44	C45	C46	1.1(5)
C4	N2	C3	C2	-1.5(4)	C44	C45	C46	S2	0.9(4)
C4	N2	C3	C15	179.3(3)	C44	C45	C46	C47	-173.9(4)
C4	N2	C35	C36	96.6(4)	C45	C46	C47	C48	-41.0(6)
C4	C5	C6	N1	179.0(5)	C45	C46	C47	C60	138.5(4)
C4	C5	C6	C43	-3.4(7)	C46	S2	C43	C6	-173.6(3)
C5	C2	C3	N2	-1.0(4)	C46	S2	C43	C44	2.6(3)
C5	C2	C3	C15	178.2(3)	C46	C47	C48	C49	-6.4(6)
C5	C6	C43	S2	176.2(3)	C46	C47	C48	C62	177.1(3)
C5	C6	C43	C44	0.6(6)	C46	C47	C60	C59	-177.6(4)
C6	N1	C1	O1	179.0(3)	C47	C48	C49	C50	179.8(4)
C6	N1	C1	C2	-0.8(4)	C47	C48	C62	C58	1.0(5)
C6	N1	C7	C8	75.2(4)	C47	C48	C62	C61	-179.1(3)
C6	C43	C44	C45	173.6(3)	C48	C47	C60	C59	2.0(6)
C7	N1	C1	O1	-3.7(5)	C48	C49	C50	C51	1.0(6)
C7	N1	C1	C2	176.5(3)	C49	C48	C62	C58	-175.7(3)
C7	N1	C6	C5	-175.2(3)	C49	C48	C62	C61	4.1(5)
C7	N1	C6	C43	7.3(6)	C49	C50	C51	C52	180.0(4)
C7	C8	C9	C10	167.0(3)	C49	C50	C51	C61	1.2(6)
C7	C8	C13	C14	67.5(4)	C50	C51	C52	C53	-177.9(4)
C8	C9	C10	C11	-169.3(5)	C50	C51	C61	C55	177.6(3)
C9	C8	C13	C14	-56.8(5)	C50	C51	C61	C62	-0.6(5)
C9	C10	C11	C12	175.9(6)	C51	C52	C53	C54	-0.7(7)
C13	C8	C9	C10	-66.6(4)	C51	C61	C62	C48	-2.0(5)
C15	S1	C18	C17	2.2(3)	C51	C61	C62	C58	177.8(3)
C15	S1	C18	C19	178.8(3)	C52	C51	C61	C55	-1.3(5)
C15	C16	C17	C18	0.8(4)	C52	C51	C61	C62	-179.5(4)
C16	C17	C18	S1	-2.1(4)	C52	C53	C54	C55	0.8(7)
C16	C17	C18	C19	-178.4(3)	C53	C54	C55	C56	178.9(4)
C17	C18	C19	C20	-141.5(4)	C53	C54	C55	C61	-1.2(6)
C17	C18	C19	C32	38.2(5)	C54	C55	C56	C57	-178.3(4)
C18	S1	C15	C3	174.6(3)	C54	C55	C61	C51	1.4(5)
C18	S1	C15	C16	-1.8(3)	C54	C55	C61	C62	179.6(4)
C18	C19	C20	C21	5.1(5)	C55	C56	C57	C58	-2.0(6)

C18	C19	C20	C33	-177.7(3)	C55	C61	C62	C48	179.8(3)
C18	C19	C32	C31	177.7(3)	C55	C61	C62	C58	-0.4(5)
C19	C20	C21	C22	-178.4(4)	C56	C55	C61	C51	-178.6(4)
C19	C20	C33	C30	-1.0(5)	C56	C55	C61	C62	-0.4(5)
C19	C20	C33	C34	179.8(3)	C56	C57	C58	C59	-179.9(4)
C20	C19	C32	C31	-2.5(5)	C56	C57	C58	C62	1.1(6)
C20	C21	C22	C23	-2.2(6)	C57	C58	C59	C60	179.5(4)
C20	C33	C34	C23	-0.7(6)	C57	C58	C62	C48	179.9(3)
C20	C33	C34	C27	179.8(3)	C57	C58	C62	C61	0.1(5)
C21	C20	C33	C30	176.5(3)	C58	C59	C60	C47	0.1(6)
C21	C20	C33	C34	-2.8(5)	C59	C58	C62	C48	1.0(5)
C21	C22	C23	C24	-179.0(4)	C59	C58	C62	C61	-178.9(3)
C21	C22	C23	C34	-1.4(6)	C60	C47	C48	C49	174.1(3)
C22	C23	C24	C25	177.5(4)	C60	C47	C48	C62	-2.5(5)
C22	C23	C34	C27	-177.7(4)	C61	C51	C52	C53	0.9(6)
C22	C23	C34	C33	2.8(6)	C61	C55	C56	C57	1.7(6)
C23	C24	C25	C26	-0.5(8)	C62	C48	C49	C50	-3.7(5)
C24	C23	C34	C27	0.0(6)	C62	C58	C59	C60	-1.5(6)

Supplementary Table 19. Hydrogen Atom Coordinates (Å×10^4) and Isotropic Displacement Parameters (Å^2×10^3) for **Py-TDPP.**

Atom	x	y	z	U(eq)
H63A	3440.02	7387.83	8886.43	124
H63B	3043.78	7559.39	9711.55	124
H7A	8975.99	6095.71	3373.95	32
H7B	10391.16	6558.1	3289.01	32
H8	8284.65	7703.24	2688.29	34
H9A	10186.28	6476.74	1887.95	41
H9B	8745.19	6110.06	2234.21	41
H10A	9357.86	7758.46	1076.72	54
H10B	7880.3	7587.94	1482.56	54
H11A	9468.02	6129.12	691.98	102
H11B	8034.13	5896.5	1136.53	102
H12A	8002.2	6391.43	-124.98	136
H12B	8494	7486	-68.19	136
H12C	7078.2	7139.07	346.58	136
H13A	9829.54	8762.75	1972.13	38
H13B	10039.22	8694.08	2793.34	38
H14A	11686.53	7673.18	1737.48	64
H14B	11876.64	7536.53	2567.67	64

H14C	12087.6	8620.09	2066.86	64
H16	11800.07	8539.5	4854.37	31
H17	13456.86	9057.24	5478.18	31
H21	10892.5	8681.13	7662.67	39
H22	10335.04	8478.52	8890.74	45
H24	10713.62	8829.04	10097.3	57
H25	11935.33	9582.87	10776.49	60
H26	13696.36	10558.28	10215.18	53
H28	15099.42	11378.73	9079.47	45
H29	15611.12	11639.13	7858.82	43
H31	15252.92	11293.4	6665.24	37
H32	14073.28	10554.39	5956.12	35
H35A	6464.88	9490.23	6429.12	32
H35B	7910	9851.97	6427	32
H36	8438.4	8161.77	7029.26	35
H37A	6831.54	7057.42	7577.75	38
H37B	6762.74	7282.31	6732.91	38
H38A	4939.15	8125.49	7822.61	47
H38B	4887.89	8414.71	6975.16	47
H39A	4592.55	6382.21	7741.23	73
H39B	3370.3	7134.54	7571.16	73
H40A	4002.28	7113.51	6325.5	101
H40B	3876.46	5962.8	6728.24	101
H40C	5295.25	6427.96	6467.65	101
H41A	7991.7	9564.81	7663.86	43
H41B	6462.17	9336.58	7834.01	43
H42A	8367.02	7862.69	8349.12	66
H42B	6815.52	7880.94	8626.69	66
H42C	7692.75	8709.79	8822.45	66
H44	5100.51	7477.77	4916.05	36
H45	3396.34	6837.75	4372.5	38
H49	2850.62	5208.66	4406.19	38
H50	973.9	4307.5	4714.02	40
H52	-850.25	3470.38	4359.32	49
H53	-1807.99	2966.63	3447.18	56
H54	-942.02	3394.52	2251.98	54
H56	791.14	4218	1290.82	51
H57	2689.54	5060.88	966.14	47
H59	4499.39	5935.09	1316.81	42
H60	5460.25	6469.04	2197.28	38

H63C	2234.69	7268.06	9629.19	124
H63D	2432.41	6489.46	10349.65	124

2.7 X-ray crystallographic data for An-TDPP

Supplementary Table 20. Crystal data and structure refinement for **An-TDPP**.

Identification code	**An-TDPP**
Empirical formula	$C_{58}H_{56}N_2O_2S_2$
Formula weight	877.16
Temperature/K	150.00(10)
Crystal system	monoclinic
Space group	C2/c
a/Å	27.4422(14)
b/Å	9.1811(3)
c/Å	20.1727(9)
α/°	90
β/°	116.452(4)
γ/°	90
Volume/Å^3	4550.4(4)
Z	4
ρcalcg/cm^3	1.280
μ/mm^{-1}	1.419
F(000)	1864.0
Crystal size/mm^3	0.13 × 0.11 × 0.1
Radiation	CuKα (λ = 1.54178)
2Θ range for data collection/°	7.196 to 134.122
Index ranges	-29 ≤ h ≤ 32, -10 ≤ k ≤ 9, -23 ≤ l ≤ 24
Reflections collected	8591
Independent reflections	4059 [Rint = 0.0625, Rsigma = 0.0656]
Data/restraints/parameters	4059/0/291
Goodness-of-fit on F^2	1.113
Final R indexes [I>=2σ (I)]	R1 = 0.0963, wR2 = 0.2655
Final R indexes [all data]	R1 = 0.1059, wR2 = 0.2785
Largest diff. peak/hole / e Å^{-3}	0.85/-0.47

Supplementary Table 21. Fractional Atomic Coordinates (×10^4) and Equivalent Isotropic Displacement Parameters (Å^2×10^3) for **An-TDPP**. Ueqis defined as 1/3 of of the trace of the orthogonalised UIJtensor.

Atom	x	y	z	U(eq)

S1	4293.0(3)	3029.1(11)	6167.2(5)	34.4(4)
O1	6134.1(11)	626(3)	6069.3(15)	39.4(7)
N1	5293.7(12)	1521(3)	5895.6(16)	28.9(7)
C1	3606.1(15)	3396(4)	5692.0(19)	28.3(8)
C2	3392.0(15)	2729(4)	5022(2)	33.2(9)
C3	3770.7(16)	1929(4)	4879(2)	32.9(8)
C4	4288.9(15)	1985(4)	5444.3(18)	25.7(7)
C5	3312.2(14)	4348(4)	5981.9(18)	29.0(8)
C6	3285.8(16)	5849(4)	5844(2)	34.2(9)
C7	3591(2)	6551(5)	5516(2)	42.5(10)
C8	3544(3)	8003(5)	5379(3)	53.4(13)
C9	3199(2)	8873(5)	5562(3)	54.7(13)
C10	2897(2)	8271(5)	5869(2)	48.8(12)
C11	2926.4(18)	6746(5)	6021(2)	37.5(9)
C12	2614.9(17)	6099(5)	6324(2)	38.8(10)
C13	2655.7(14)	4636(5)	6493.6(18)	32.5(9)
C14	2337.9(16)	3967(5)	6809(2)	38.3(10)
C15	2391.7(18)	2534(6)	6990(2)	42.9(10)
C16	2765.0(18)	1656(5)	6859(2)	40.2(10)
C17	3063.9(17)	2244(5)	6541(2)	34.4(8)
C18	3018.0(14)	3726(4)	6335.6(18)	29.0(8)
C19	4749.1(14)	1264(4)	5424.8(19)	25.6(7)
C20	5636.3(15)	617(4)	5715.8(19)	29.3(8)
C21	5266.6(14)	-220(4)	5082(2)	27.8(8)
C22	5523.9(14)	2556(4)	6505.4(19)	29.9(8)
C23	5489.5(16)	4153(4)	6241(2)	35.7(9)
C24	5628.4(19)	5129(5)	6921(2)	43.6(10)
C25	5585(2)	6766(5)	6791(3)	52.6(12)
C26	5706(2)	7619(6)	7491(3)	59.2(13)
C27	5654(3)	9203(6)	7377(4)	73.1(17)
C28	5842.4(18)	4413(5)	5844(2)	41.5(10)
C29	6457(2)	4281(6)	6322(3)	54.6(12)

Supplementary Table 22. Anisotropic Displacement Parameters (Å^2×10^3) for **An-TDPP**. The Anisotropic displacement factor exponent takes the form: $-2\pi^2[h^2a^{*2}U11+2hka^*b^*U12+\ldots]$.

Atom	U11	U22	U33	U23	U13	U12
S1	31.4(5)	40.6(6)	27.2(5)	-12.7(4)	9.6(4)	4.9(4)
O1	29.5(14)	41.7(17)	39.1(15)	-18.8(12)	8.1(11)	1.1(11)
N1	29.2(15)	25.1(16)	28.0(15)	-7.1(12)	8.6(12)	2.0(12)
C1	34.0(18)	23.3(18)	26.6(17)	1.9(14)	12.8(14)	4.2(14)

C2	32.1(18)	32(2)	28.5(18)	-3.4(15)	7.2(15)	9.3(15)
C3	39(2)	27(2)	27.4(18)	-8.0(14)	10.5(15)	4.8(15)
C4	34.9(18)	17.6(17)	22.8(16)	-5.1(12)	11.2(14)	0.5(13)
C5	33.9(17)	29.1(19)	21.1(16)	-3.5(14)	9.6(14)	4.8(14)
C6	47(2)	30(2)	24.2(17)	-5.1(14)	15.0(15)	3.2(16)
C7	61(3)	36(2)	35(2)	-0.4(17)	25.7(19)	2.4(19)
C8	88(4)	31(2)	45(3)	-2.0(18)	34(3)	-6(2)
C9	93(4)	24(2)	43(2)	0.9(18)	27(2)	6(2)
C10	74(3)	31(2)	37(2)	-2.9(18)	21(2)	18(2)
C11	54(2)	30(2)	27.4(19)	-3.3(15)	17.2(17)	11.6(17)
C12	48(2)	44(2)	25.0(17)	-2.9(16)	16.1(16)	17.1(18)
C13	30.5(17)	43(2)	18.7(16)	-6.2(15)	6.2(13)	6.1(15)
C14	33.8(18)	58(3)	22.2(17)	-8.9(17)	11.9(14)	0.1(18)
C15	47(2)	60(3)	22.8(18)	-3.9(18)	15.9(16)	-11(2)
C16	51(2)	39(2)	26.4(18)	1.4(16)	14.0(17)	-4.7(18)
C17	44(2)	34(2)	22.8(17)	-2.8(15)	12.5(15)	1.8(16)
C18	31.5(17)	32(2)	18.6(15)	-3.7(14)	6.3(13)	4.9(15)
C19	28.9(17)	20.1(17)	25.5(16)	-2.1(13)	10.2(13)	0.8(13)
C20	32.7(18)	25.3(18)	27.2(17)	-6.3(14)	10.9(14)	2.2(14)
C21	29.6(17)	22.5(17)	29.3(17)	-4.7(14)	11.5(13)	3.6(13)
C22	33.2(18)	26.1(19)	26.8(17)	-8.1(14)	10.2(14)	0.0(14)
C23	37.8(19)	29(2)	34(2)	-5.7(16)	11.1(16)	2.3(15)
C24	51(2)	31(2)	44(2)	-9.8(18)	16.5(19)	-3.1(18)
C25	73(3)	31(2)	49(3)	-0.1(19)	22(2)	5(2)
C26	69(3)	54(3)	45(3)	-4(2)	16(2)	3(3)
C27	104(5)	45(3)	65(3)	-8(3)	33(3)	-7(3)
C28	51(2)	38(2)	34(2)	-0.7(17)	16.8(18)	-1.1(18)
C29	48(3)	63(3)	48(3)	-8(2)	16(2)	-2(2)

Supplementary Table 23. Bond Lengths for **An-TDPP**.

Atom	Atom	Length/Å	Atom	Atom	Length/Å
S1	C1	1.725(4)	C11	C12	1.387(7)
S1	C4	1.741(3)	C12	C13	1.378(6)
O1	C20	1.228(5)	C13	C14	1.427(6)
N1	C19	1.388(4)	C13	C18	1.439(5)
N1	C20	1.417(5)	C14	C15	1.356(7)
N1	C22	1.458(5)	C15	C16	1.417(7)
C1	C2	1.356(5)	C16	C17	1.359(6)
C1	C5	1.476(5)	C17	C18	1.411(6)
C2	C3	1.404(5)	C19	C211	1.388(5)

C3	C4	1.371(5)	C20	C21	1.449(5)
C4	C19	1.442(5)	C21	C211	1.408(7)
C5	C6	1.401(6)	C22	C23	1.548(6)
C5	C18	1.414(5)	C23	C24	1.537(6)
C6	C7	1.431(6)	C23	C28	1.526(6)
C6	C11	1.446(6)	C24	C25	1.521(6)
C7	C8	1.356(6)	C25	C26	1.516(7)
C8	C9	1.407(8)	C26	C27	1.469(8)
C9	C10	1.353(8)	C28	C29	1.530(6)
C10	C11	1.428(6)			

Supplementary Table 24. Bond Angles for **An-TDPP.**

Atom	Atom	Atom	Angle/˚	Atom	Atom	Atom	Angle/˚
C1	S1	C4	92.54(17)	C12	C13	C18	119.9(4)
C19	N1	C20	111.1(3)	C14	C13	C18	118.0(4)
C19	N1	C22	128.2(3)	C15	C14	C13	121.5(4)
C20	N1	C22	120.7(3)	C14	C15	C16	120.2(4)
C2	C1	S1	110.3(3)	C17	C16	C15	120.0(4)
C2	C1	C5	126.3(3)	C16	C17	C18	121.8(4)
C5	C1	S1	123.4(3)	C5	C18	C13	118.9(4)
C1	C2	C3	114.2(3)	C17	C18	C5	122.7(3)
C4	C3	C2	113.4(3)	C17	C18	C13	118.4(4)
C3	C4	S1	109.5(3)	N1	C19	C4	126.3(3)
C3	C4	C19	123.8(3)	N1	C19	C211	107.0(3)
C19	C4	S1	126.6(3)	C211	C19	C4	126.6(3)
C6	C5	C1	119.5(3)	O1	C20	N1	122.8(3)
C6	C5	C18	120.4(3)	O1	C20	C21	132.6(3)
C18	C5	C1	119.8(3)	N1	C20	C21	104.6(3)
C5	C6	C7	123.0(4)	C191	C21	C20	142.8(3)
C5	C6	C11	119.5(4)	C191	C21	C211	109.7(4)
C7	C6	C11	117.5(4)	C211	C21	C20	107.5(4)
C8	C7	C6	121.0(4)	N1	C22	C23	113.0(3)
C7	C8	C9	121.3(5)	C24	C23	C22	107.0(3)
C10	C9	C8	120.5(4)	C28	C23	C22	112.0(3)
C9	C10	C11	120.8(4)	C28	C23	C24	114.4(4)
C10	C11	C6	119.0(4)	C25	C24	C23	117.0(4)
C12	C11	C6	119.1(4)	C26	C25	C24	112.5(4)
C12	C11	C10	121.9(4)	C27	C26	C25	113.6(5)
C13	C12	C11	121.9(4)	C23	C28	C29	115.9(4)
C12	C13	C14	122.1(4)				

Supplementary Table 25. Torsion Angles for **An-TDPP**.

A	B	C	D	Angle/˚	A	B	C	D	Angle/˚
S1	C1	C2	C3	-0.3(5)	C8	C9	C10	C11	0.7(7)
S1	C1	C5	C6	88.3(4)	C9	C10	C11	C6	0.2(7)
S1	C1	C5	C18	-97.6(4)	C9	C10	C11	C12	-179.0(4)
S1	C4	C19	N1	-15.3(5)	C10	C11	C12	C13	-177.9(4)
S1	C4	C19	C211	167.1(3)	C11	C6	C7	C8	0.1(6)
O1	C20	C21	C191	-2.8(9)	C11	C12	C13	C14	179.7(4)
O1	C20	C21	C211	179.2(5)	C11	C12	C13	C18	-1.8(6)
N1	C20	C21	C191	178.1(5)	C12	C13	C14	C15	-178.3(4)
N1	C20	C21	C211	0.1(5)	C12	C13	C18	C5	-2.3(5)
N1	C22	C23	C24	168.0(3)	C12	C13	C18	C17	177.8(3)
N1	C22	C23	C28	-65.9(4)	C13	C14	C15	C16	-0.7(6)
C1	S1	C4	C3	-1.1(3)	C14	C13	C18	C5	176.2(3)
C1	S1	C4	C19	179.4(3)	C14	C13	C18	C17	-3.7(5)
C1	C2	C3	C4	-0.6(5)	C14	C15	C16	C17	-1.1(6)
C1	C5	C6	C7	-8.5(6)	C15	C16	C17	C18	0.5(6)
C1	C5	C6	C11	170.0(3)	C16	C17	C18	C5	-177.9(4)
C1	C5	C18	C13	-168.8(3)	C16	C17	C18	C13	2.0(5)
C1	C5	C18	C17	11.1(5)	C18	C5	C6	C7	177.4(3)
C2	C1	C5	C6	-89.5(5)	C18	C5	C6	C11	-4.2(5)
C2	C1	C5	C18	84.7(5)	C18	C13	C14	C15	3.2(5)
C2	C3	C4	S1	1.1(4)	C19	N1	C20	O1	-178.1(4)
C2	C3	C4	C19	-179.4(4)	C19	N1	C20	C21	1.1(4)
C3	C4	C19	N1	165.3(4)	C19	N1	C22	C23	-74.1(5)
C3	C4	C19	C211	-12.3(6)	C20	N1	C19	C4	-179.9(3)
C4	S1	C1	C2	0.8(3)	C20	N1	C19	C211	-1.9(4)
C4	S1	C1	C5	-177.3(3)	C20	N1	C22	C23	105.6(4)
C5	C1	C2	C3	177.7(4)	C22	N1	C19	C4	-0.1(6)
C5	C6	C7	C8	178.6(4)	C22	N1	C19	C211	177.9(4)
C5	C6	C11	C10	-179.1(4)	C22	N1	C20	O1	2.2(6)
C5	C6	C11	C12	0.0(6)	C22	N1	C20	C21	-178.7(3)
C6	C5	C18	C13	5.3(5)	C22	C23	C24	C25	-176.0(4)
C6	C5	C18	C17	-174.8(3)	C22	C23	C28	C29	-66.2(5)
C6	C7	C8	C9	0.8(8)	C23	C24	C25	C26	177.4(4)
C6	C11	C12	C13	3.0(6)	C24	C23	C28	C29	55.7(5)
C7	C6	C11	C10	-0.6(6)	C24	C25	C26	C27	-178.7(5)
C7	C6	C11	C12	178.6(4)	C28	C23	C24	C25	59.4(5)
C7	C8	C9	C10	-1.2(8)					

Supplementary Table 26. Hydrogen Atom Coordinates (Å×10^4) and Isotropic Displacement Parameters (Å^2×10^3) for **An-TDPP.**

Atom	x	y	z	U(eq)
H2	3018.17	2797.55	4679.62	40
H3	3677.09	1399.69	4434.19	39
H7	3830.25	5990.92	5392.85	51
H8	3747.62	8441.45	5155.84	64
H9	3178.07	9891.18	5469.65	66
H10	2663.14	8870.24	5984.42	59
H12	2366.21	6680.73	6418.37	47
H14	2082.8	4539.09	6893.91	46
H15	2178.39	2116.77	7204.2	51
H16	2806.46	656.76	6995.01	48
H17	3310.57	1640.3	6453.31	41
H22A	5910.07	2304.24	6819.46	36
H22B	5327.95	2470.07	6813.75	36
H23	5103.03	4350.42	5880.32	43
H24A	5384.93	4857.33	7144.18	52
H24B	6005.34	4904.99	7289.31	52
H25A	5843.41	7065.32	6596.78	63
H25B	5212.9	7005.45	6411.76	63
H26A	5452.91	7297.01	7690.7	71
H26B	6080.37	7390.67	7865.21	71
H27A	5284.77	9439.34	7005.38	110
H27B	5917.29	9539.69	7204.87	110
H27C	5726.72	9685.73	7845.07	110
H28A	5763.6	5401.93	5625.24	50
H28B	5732.94	3709.26	5430.78	50
H29A	6647.62	4581.71	6032.48	82
H29B	6549.71	3267.58	6480.51	82
H29C	6568.48	4909.09	6757.74	82

2.8. X-ray crystallographic data for Flu-TDPP

Supplementary Table 27. Crystal data and structure refinement for **Flu-TDPP**.

Identification code	**Flu-TDPP**
Empirical formula	$C_{60}H_{64}N_2O_2S_2$
Formula weight	909.25
Temperature/K	149.99(10)

Crystal system	monoclinic
Space group	$P2_1/c$
a/Å	11.97270(10)
b/Å	14.9060(2)
c/Å	27.9247(3)
α/°	90
β/°	98.8940(10)
γ/°	90
Volume/Å^3	4923.66(10)
Z	4
ρ_{calc}g/cm^3	1.227
μ/mm^{-1}	1.327
F(000)	1944.0
Crystal size/mm^3	0.1 × 0.05 × 0.02
Radiation	CuKα (λ = 1.54184)
2Θ range for data collection/°	6.408 to 147.462
Index ranges	$-14 \le h \le 7$, $-18 \le k \le 18$, $-34 \le l \le 34$
Reflections collected	52024
Independent reflections	9658 [R_{int} = 0.0384, R_{sigma} = 0.0284]
Data/restraints/parameters	9658/262/660
Goodness-of-fit on F^2	1.036
Final R indexes [I>=2σ (I)]	R_1 = 0.0729, wR_2 = 0.2080
Final R indexes [all data]	R_1 = 0.0872, wR_2 = 0.2213
Largest diff. peak/hole / e Å^{-3}	1.29/-0.66

Supplementary Table 28. Fractional Atomic Coordinates (×10^4) and Equivalent Isotropic Displacement Parameters (Å^2×10^3) for **Flu-TDPP**. U_{eq} is defined as 1/3 of of the trace of the orthogonalised U_{IJ} tensor.

Atom	x	y	z	U(eq)
S1	4601.9(6)	6053.6(5)	3863.1(3)	47.6(2)
S2	10361.3(6)	3764.1(5)	6071.5(3)	45.9(2)
O1	8790.9(18)	6611.2(15)	5030.1(9)	55.1(6)
O2	6220.1(17)	3216.0(14)	4900.2(8)	47.0(5)
N1	7048(2)	6160.1(17)	4612.1(10)	46.4(6)
N2	7964.4(19)	3667.8(16)	5315.3(8)	40.2(5)
C1	6381(2)	5387(2)	4565.9(10)	41.7(6)
C01B	6596(3)	7902(2)	5157.5(12)	55.3(8)
C2	6988(2)	4733.4(19)	4846.7(10)	38.8(6)
C3	6930(2)	3809(2)	4996.5(10)	40.1(6)
C4	8613(2)	4449.1(19)	5370.7(10)	38.2(6)
C5	8017(2)	5095(2)	5078.9(10)	39.7(6)
C6	8078(2)	6020(2)	4930.8(11)	46.8(7)
C7	6795(3)	7039(2)	4388.2(11)	46.0(7)
C8	6022(3)	7608(2)	4651.7(12)	49.4(7)
C9	5556(3)	8396(2)	4332.0(12)	50.8(7)
C10	4697(3)	8966(2)	4529.5(13)	57.2(8)
C11	4184(3)	9718(3)	4185.5(13)	64.9(10)
C12	3542(4)	9378(3)	3711.3(14)	73.5(11)
C13	7558(3)	8594(3)	5159.5(14)	65.5(10)
C14	5264(2)	5298(2)	4289.3(10)	41.8(6)
C15	4570(2)	4575(2)	4321.8(11)	44.2(6)
C16	3533(2)	4627(2)	4013.0(11)	45.1(7)
C17	3406(2)	5389.1(19)	3736.5(10)	41.4(6)
C18	2454(2)	5652.3(19)	3369.5(10)	40.0(6)
C19	1504(2)	5093(2)	3271.8(11)	45.1(7)

C20	599(2)	5307(2)	2916.2(11)	45.1(7)
C21	635(2)	6088.4(19)	2649.8(10)	39.3(6)
C22	1571(2)	6659.0(19)	2742.8(11)	40.7(6)
C23	2472(2)	6447(2)	3100.4(11)	43.1(6)
C24	-147(2)	6461(2)	2242.3(10)	40.3(6)
C25	327(2)	7245(2)	2086.8(11)	41.9(6)
C26	1458(2)	7450(2)	2398.8(11)	45.3(7)
C27	-1190(3)	6155(2)	2007.6(11)	48.6(7)
C28	-1728(3)	6628(2)	1608.2(12)	53.8(8)
C29	-1234(3)	7392(2)	1445.9(12)	52.8(8)
C30	-215(3)	7711(2)	1686.0(11)	47.6(7)
C31	1421(3)	8340(2)	2667.0(15)	67.6(11)
C32	2413(3)	7455(3)	2092.1(14)	63.6(10)
C33	8180(3)	2808(2)	5559.9(12)	46.7(7)
C34	8968(3)	2170(2)	5352.8(15)	67.9(10)
C35	9199(15)	1232(7)	5581(4)	101(2)
C35A	9198(8)	1439(6)	5762(4)	99.0(19)
C36	9780(11)	1362(7)	6101(5)	98(2)
C36A	10388(6)	1048(5)	5850(3)	89(2)
C37	9888(12)	429(9)	6343(5)	127(3)
C37A	10669(8)	290(7)	6202(5)	119(2)
C38	11086(14)	44(12)	6364(9)	146(4)
C38A	11912(7)	-85(7)	6193(5)	141(3)
C39	8159(10)	1899(10)	4866(3)	104(3)
C39A	8564(5)	1856(6)	4840(3)	84.4(18)
C40	8696(7)	2334(5)	4499(3)	56(2)
C40A	9278(7)	1584(8)	4463(4)	122(3)

C41	9678(2)	4568(2)	5685.1(10)	40.7(6)
C42	10279(2)	5364(2)	5730.1(11)	43.9(6)
C43	11267(2)	5314(2)	6070.4(11)	44.3(6)
C44	11435(2)	4493(2)	6292.5(11)	44.2(6)
C45	12318(2)	4208(2)	6688.1(11)	45.4(7)
C46	12982(3)	4846(2)	6963.7(12)	52.8(8)
C47	13739(3)	4601(2)	7368.6(13)	56.3(8)
C48	13849(2)	3706(2)	7498.0(11)	48.3(7)
C49	13230(2)	3051(2)	7213.7(11)	45.9(7)
C50	12462(2)	3300(2)	6814.5(11)	44.3(6)
C51	14504(3)	3258(3)	7918.3(12)	53.9(8)
C52	14304(3)	2335(3)	7875.9(12)	54.1(8)
C53	13508(3)	2116(2)	7408.9(12)	51.2(8)
C54	15199(3)	3609(3)	8320.9(13)	64.1(10)
C55	15673(3)	3020(4)	8682.3(14)	74.5(12)
C56	15479(3)	2109(4)	8638.4(14)	77.4(13)
C57	14803(3)	1758(3)	8237.8(13)	66.1(10)
C58	12468(3)	1593(3)	7497.8(14)	60.1(9)
C59	14138(3)	1598(3)	7055.5(13)	68.6(11)

Supplementary Table 29. Anisotropic Displacement Parameters (Å^2×10^3) for **Flu-TDPP**. The Anisotropic displacement factor exponent takes the form: $-2\pi^2[h^2a^{*2}U_{11}+2hka^*b^*U_{12}+\ldots]$.

Atom	U_{11}	U_{22}	U_{33}	U_{23}	U_{13}	U_{12}
S1	36.2(4)	46.0(4)	55.2(4)	7.0(3)	-9.9(3)	-1.6(3)
S2	37.2(4)	45.9(4)	50.1(4)	-0.6(3)	-7.2(3)	4.1(3)
O1	41.8(12)	52.5(13)	67.2(14)	7.7(11)	-3.8(10)	-4.0(10)
O2	37.3(11)	48.2(12)	52.6(12)	4.2(9)	-2.3(9)	-2.7(9)
N1	38.3(13)	45.1(14)	52.5(14)	6.6(11)	-4.2(11)	-0.4(10)

N2	34.4(12)	42.5(13)	41.5(12)	2.9(10)	-0.9(9)	1.6(9)
C1	32.8(14)	47.4(16)	42.9(14)	1.1(12)	-0.8(11)	-0.2(11)
C01B	55(2)	63(2)	46.9(17)	1.2(15)	5.4(14)	6.1(15)
C2	31.9(14)	45.4(15)	37.9(13)	0.5(11)	2.0(10)	-0.3(11)
C3	33.9(14)	45.6(15)	39.8(14)	0.1(11)	3.0(11)	1.3(11)
C4	33.0(14)	44.9(15)	36.3(13)	-0.1(11)	4.4(10)	1.8(11)
C5	30.7(14)	46.9(15)	40.3(14)	2.6(12)	1.7(11)	-0.7(11)
C6	36.4(15)	49.7(17)	50.1(17)	2.6(13)	-6.0(12)	-0.6(12)
C7	40.3(16)	50.3(17)	46.8(16)	5.0(13)	4.6(12)	1.0(12)
C8	39.2(16)	53.1(18)	55.0(18)	-6.1(14)	4.3(13)	1.5(13)
C9	47.3(17)	52.5(18)	48.9(17)	-0.8(14)	-4.4(13)	-1.9(14)
C10	56(2)	58(2)	54.5(19)	-8.6(15)	0.1(15)	3.5(15)
C11	69(2)	59(2)	61(2)	-10.6(17)	-4.3(17)	16.3(18)
C12	75(3)	78(3)	61(2)	-3.3(19)	-11.8(19)	26(2)
C13	59(2)	70(2)	63(2)	-15.2(18)	-6.7(17)	1.2(18)
C14	33.8(14)	46.7(15)	42.4(15)	0.2(12)	-1.6(11)	3.8(11)
C15	38.2(15)	46.4(16)	45.5(15)	2.7(12)	-1.2(12)	0.7(12)
C16	38.6(15)	47.2(16)	47.1(16)	0.1(13)	-0.9(12)	-2.8(12)
C17	36.6(15)	42.1(15)	44.0(15)	-3.0(12)	0.9(11)	3.1(11)
C18	31.8(14)	43.1(15)	43.3(14)	-4.1(12)	0.2(11)	1.9(11)
C19	40.0(16)	45.7(16)	47.2(16)	1.7(13)	-0.5(12)	-3.5(12)
C20	36.0(15)	48.2(16)	48.9(16)	-0.9(13)	0.2(12)	-7.1(12)
C21	30.1(13)	46.7(15)	40.1(14)	-4.7(12)	1.8(10)	-0.3(11)
C22	32.1(14)	41.6(15)	46.0(15)	-2.2(12)	-1.0(11)	1.5(11)
C23	30.4(14)	42.8(15)	52.7(16)	-0.7(13)	-4.1(12)	-1.9(11)
C24	31.2(14)	48.4(16)	40.1(14)	-4.8(12)	1.4(11)	0.0(11)
C25	31.4(14)	48.1(16)	44.8(15)	-3.9(12)	1.2(11)	4.0(11)

C26	33.1(15)	45.0(16)	53.7(17)	4.0(13)	-6.4(12)	-1.5(11)
C27	37.2(16)	56.8(18)	48.5(16)	-3.0(14)	-3.3(12)	-6.1(13)
C28	38.2(16)	65(2)	53.5(18)	-7.9(15)	-8.6(13)	-2.2(14)
C29	45.8(18)	61(2)	47.7(17)	-1.2(14)	-6.4(13)	5.9(14)
C30	38.4(16)	51.4(17)	50.6(17)	3.7(13)	-0.6(12)	2.6(13)
C31	68(2)	45.5(18)	77(2)	-4.3(17)	-27.1(19)	1.8(16)
C32	35.2(17)	79(2)	75(2)	23.4(19)	4.1(15)	-3.1(15)
C33	39.5(16)	44.4(16)	54.2(17)	6.1(13)	0.3(13)	-0.1(12)
C34	46.5(19)	48.8(19)	108(3)	-6.3(19)	11.7(19)	1.0(15)
C35	73(4)	69(4)	165(5)	-2(4)	28(4)	14(4)
C35A	74(3)	70(3)	161(5)	16(3)	44(4)	17(3)
C36	63(4)	77(4)	158(5)	4(4)	26(4)	10(4)
C36A	105(5)	77(5)	83(5)	14(4)	3(4)	-7(4)
C37	98(5)	101(5)	181(6)	20(5)	22(5)	12(5)
C37A	94(4)	93(4)	175(5)	33(4)	35(4)	27(4)
C38	111(7)	124(8)	199(9)	22(7)	14(8)	18(7)
C38A	70(5)	117(6)	229(9)	69(6)	1(6)	-7(4)
C39	72(5)	92(5)	150(5)	-23(4)	25(4)	6(5)
C39A	28(3)	85(4)	144(4)	-61(3)	25(3)	-9(3)
C40	40(4)	40(4)	76(5)	-18(4)	-27(4)	-2(3)
C40A	72(5)	155(7)	139(6)	-75(6)	13(4)	12(5)
C41	33.4(14)	47.0(15)	40.3(14)	-0.7(12)	1.6(11)	3.3(11)
C42	36.2(15)	49.4(16)	44.6(15)	1.4(13)	1.1(11)	2.9(12)
C43	34.1(14)	50.8(17)	46.1(15)	-0.3(13)	0.6(11)	0.7(12)
C44	36.7(15)	48.3(16)	45.6(15)	-4.5(13)	-0.3(12)	5.0(12)
C45	35.7(15)	51.1(17)	46.6(16)	-1.5(13)	-2.2(12)	4.6(12)
C46	42.2(17)	50.0(17)	61.1(19)	0.7(15)	-8.0(14)	-0.2(13)

C47	39.5(17)	64(2)	59.6(19)	-0.8(16)	-9.8(14)	-4.8(14)
C48	30.4(15)	66(2)	46.8(16)	3.2(14)	-1.3(12)	3.1(13)
C49	33.8(15)	56.7(18)	47.4(16)	4.8(14)	7.2(12)	6.9(12)
C50	35.1(15)	49.5(16)	46.8(15)	-1.0(13)	0.9(12)	5.1(12)
C51	34.6(16)	80(2)	47.0(17)	8.4(16)	4.8(12)	5.5(15)
C52	37.7(16)	78(2)	48.2(17)	13.8(16)	10.6(13)	9.0(15)
C53	43.2(17)	61.3(19)	49.5(17)	9.5(15)	8.6(13)	11.5(14)
C54	39.3(18)	95(3)	54.9(19)	5.1(19)	-1.6(14)	1.2(17)
C55	45(2)	125(4)	51(2)	15(2)	-1.1(15)	0(2)
C56	54(2)	119(4)	57(2)	35(2)	4.5(17)	8(2)
C57	50(2)	90(3)	58(2)	25(2)	9.9(16)	7.2(18)
C58	58(2)	60(2)	62(2)	3.3(17)	11.6(16)	-0.5(16)
C59	72(2)	79(3)	58(2)	13.3(18)	18.0(18)	35(2)

Supplementary Table 30. Bond Lengths for **Flu-TDPP**.

Atom	Atom	Length/Å	Atom	Atom	Length/Å
S1	C14	1.738(3)	C25	C30	1.390(4)
S1	C17	1.732(3)	C26	C31	1.527(5)
S2	C41	1.731(3)	C26	C32	1.531(5)
S2	C44	1.723(3)	C27	C28	1.391(4)
O1	C6	1.229(4)	C28	C29	1.391(5)
O2	C3	1.227(3)	C29	C30	1.382(4)
N1	C1	1.397(4)	C33	C34	1.515(5)
N1	C6	1.420(4)	C34	C35	1.543(8)
N1	C7	1.463(4)	C34	C35A	1.573(7)
N2	C3	1.424(3)	C34	C39	1.594(9)
N2	C4	1.395(4)	C34	C39A	1.514(7)

N2	C33	1.457(4)	C35	C36	1.522(9)
C1	C2	1.384(4)	C35A	C36A	1.523(8)
C1	C14	1.444(4)	C36	C37	1.543(9)
C01B	C8	1.536(4)	C36A	C37A	1.501(8)
C01B	C13	1.544(5)	C37	C38	1.537(9)
C2	C3	1.445(4)	C37A	C38A	1.593(8)
C2	C5	1.408(4)	C39	C40	1.445(9)
C4	C5	1.386(4)	C39A	C40A	1.511(7)
C4	C41	1.443(4)	C41	C42	1.383(4)
C5	C6	1.444(4)	C42	C43	1.401(4)
C7	C8	1.526(4)	C43	C44	1.372(4)
C8	C9	1.527(4)	C44	C45	1.469(4)
C9	C10	1.503(5)	C45	C46	1.393(4)
C10	C11	1.540(5)	C45	C50	1.402(4)
C11	C12	1.512(5)	C46	C47	1.385(4)
C14	C15	1.373(4)	C47	C48	1.382(5)
C15	C16	1.400(4)	C48	C49	1.397(4)
C16	C17	1.369(4)	C48	C51	1.468(4)
C17	C18	1.463(4)	C49	C50	1.382(4)
C18	C19	1.403(4)	C49	C53	1.514(4)
C18	C23	1.405(4)	C51	C52	1.398(5)
C19	C20	1.389(4)	C51	C54	1.393(5)
C20	C21	1.386(4)	C52	C53	1.527(5)
C21	C22	1.399(4)	C52	C57	1.389(5)
C21	C24	1.467(4)	C53	C58	1.522(5)
C22	C23	1.388(4)	C53	C59	1.540(5)
C22	C26	1.514(4)	C54	C55	1.390(6)

C24	C25	1.397(4)	C55	C56	1.380(7)
C24	C27	1.394(4)	C56	C57	1.379(6)
C25	C26	1.524(4)			

Supplementary Table 31. Bond Angles for **Flu-TDPP**.

Atom	Atom	Atom	Angle/˚	Atom	Atom	Atom	Angle/˚
C17	S1	C14	92.54(14)	C22	C26	C31	111.8(3)
C44	S2	C41	92.37(14)	C22	C26	C32	110.9(3)
C1	N1	C6	111.4(2)	C25	C26	C31	111.4(3)
C1	N1	C7	128.4(2)	C25	C26	C32	110.8(3)
C6	N1	C7	120.2(2)	C31	C26	C32	110.6(3)
C3	N2	C33	119.8(2)	C28	C27	C24	118.8(3)
C4	N2	C3	111.2(2)	C27	C28	C29	120.6(3)
C4	N2	C33	128.9(2)	C30	C29	C28	120.8(3)
N1	C1	C14	126.4(3)	C29	C30	C25	119.1(3)
C2	C1	N1	106.6(2)	N2	C33	C34	116.7(3)
C2	C1	C14	126.9(3)	C33	C34	C35	119.5(7)
C8	C01B	C13	114.9(3)	C33	C34	C35A	101.9(4)
C1	C2	C3	142.2(3)	C33	C34	C39	98.8(6)
C1	C2	C5	109.7(3)	C35	C34	C39	99.7(6)
C5	C2	C3	108.0(2)	C39A	C34	C33	115.3(4)
O2	C3	N2	122.5(3)	C39A	C34	C35A	117.9(6)
O2	C3	C2	133.3(3)	C36	C35	C34	107.8(8)
N2	C3	C2	104.2(2)	C36A	C35A	C34	115.9(6)
N2	C4	C41	126.5(3)	C35	C36	C37	107.4(8)
C5	C4	N2	106.7(2)	C37A	C36A	C35A	120.2(7)
C5	C4	C41	126.7(3)	C38	C37	C36	111.5(9)

C2	C5	C6	108.2(3)	C36A	C37A	C38A	111.6(7)
C4	C5	C2	109.8(3)	C40	C39	C34	102.3(8)
C4	C5	C6	142.0(3)	C40A	C39A	C34	127.6(6)
O1	C6	N1	122.8(3)	C4	C41	S2	125.6(2)
O1	C6	C5	133.2(3)	C42	C41	S2	110.4(2)
N1	C6	C5	104.1(2)	C42	C41	C4	124.0(3)
N1	C7	C8	113.1(3)	C41	C42	C43	112.9(3)
C7	C8	C01B	112.5(3)	C44	C43	C42	113.8(3)
C7	C8	C9	109.8(3)	C43	C44	S2	110.6(2)
C9	C8	C01B	112.9(3)	C43	C44	C45	129.5(3)
C10	C9	C8	115.4(3)	C45	C44	S2	119.8(2)
C9	C10	C11	114.3(3)	C46	C45	C44	120.1(3)
C12	C11	C10	113.7(3)	C46	C45	C50	118.9(3)
C1	C14	S1	126.4(2)	C50	C45	C44	120.9(3)
C15	C14	S1	109.7(2)	C47	C46	C45	121.1(3)
C15	C14	C1	123.8(3)	C48	C47	C46	119.5(3)
C14	C15	C16	113.8(3)	C47	C48	C49	120.3(3)
C17	C16	C15	113.8(3)	C47	C48	C51	131.7(3)
C16	C17	S1	110.1(2)	C49	C48	C51	107.9(3)
C16	C17	C18	128.6(3)	C48	C49	C53	111.8(3)
C18	C17	S1	121.3(2)	C50	C49	C48	119.9(3)
C19	C18	C17	119.8(3)	C50	C49	C53	128.3(3)
C19	C18	C23	118.4(3)	C49	C50	C45	120.2(3)
C23	C18	C17	121.8(3)	C52	C51	C48	108.5(3)
C20	C19	C18	121.4(3)	C54	C51	C48	130.8(4)
C21	C20	C19	119.4(3)	C54	C51	C52	120.7(3)
C20	C21	C22	120.1(3)	C51	C52	C53	111.0(3)

C20	C21	C24	131.7(3)	C57	C52	C51	119.9(4)
C22	C21	C24	108.1(3)	C57	C52	C53	129.1(4)
C21	C22	C26	111.4(2)	C49	C53	C52	100.6(3)
C23	C22	C21	120.4(3)	C49	C53	C58	113.0(3)
C23	C22	C26	128.1(3)	C49	C53	C59	109.5(3)
C22	C23	C18	120.2(3)	C52	C53	C59	110.6(3)
C25	C24	C21	108.4(2)	C58	C53	C52	112.9(3)
C27	C24	C21	131.2(3)	C58	C53	C59	110.0(3)
C27	C24	C25	120.3(3)	C55	C54	C51	118.4(4)
C24	C25	C26	111.0(2)	C56	C55	C54	120.8(4)
C30	C25	C24	120.4(3)	C57	C56	C55	121.0(4)
C30	C25	C26	128.5(3)	C56	C57	C52	119.2(4)
C22	C26	C25	101.0(2)				

Supplementary Table 32. Torsion Angles for **Flu-TDPP**.

A	B	C	D	Angle/˚	A	B	C	D	Angle/˚
S1	C14	C15	C16	-0.1(3)	C22	C21	C24	C27	179.6(3)
S1	C17	C18	C19	179.2(2)	C23	C18	C19	C20	0.5(5)
S1	C17	C18	C23	0.7(4)	C23	C22	C26	C25	176.3(3)
S2	C41	C42	C43	0.0(3)	C23	C22	C26	C31	-65.2(4)
S2	C44	C45	C46	-161.0(3)	C23	C22	C26	C32	58.7(4)
S2	C44	C45	C50	15.0(4)	C24	C21	C22	C23	-177.4(3)
N1	C1	C2	C3	179.6(4)	C24	C21	C22	C26	0.1(3)
N1	C1	C2	C5	1.5(3)	C24	C25	C26	C22	1.6(3)
N1	C1	C14	S1	13.0(5)	C24	C25	C26	C31	-117.3(3)
N1	C1	C14	C15	-168.4(3)	C24	C25	C26	C32	119.2(3)
N1	C7	C8	C01B	67.7(3)	C24	C25	C30	C29	0.5(5)

N1	C7	C8	C9	-165.6(3)	C24	C27	C28	C29	0.1(5)
N2	C4	C5	C2	1.1(3)	C25	C24	C27	C28	2.0(5)
N2	C4	C5	C6	-179.6(4)	C26	C22	C23	C18	-176.5(3)
N2	C4	C41	S2	-1.5(4)	C26	C25	C30	C29	178.3(3)
N2	C4	C41	C42	-179.2(3)	C27	C24	C25	C26	179.5(3)
N2	C33	C34	C35	-177.6(6)	C27	C24	C25	C30	-2.3(4)
N2	C33	C34	C35A	168.3(5)	C27	C28	C29	C30	-1.9(5)
N2	C33	C34	C39	-71.1(5)	C28	C29	C30	C25	1.6(5)
N2	C33	C34	C39A	-62.6(5)	C30	C25	C26	C22	-176.5(3)
C1	N1	C6	O1	-179.2(3)	C30	C25	C26	C31	64.7(4)
C1	N1	C6	C5	0.6(3)	C30	C25	C26	C32	-58.8(4)
C1	N1	C7	C8	80.3(4)	C33	N2	C3	O2	-3.3(4)
C1	C2	C3	O2	1.6(7)	C33	N2	C3	C2	176.9(2)
C1	C2	C3	N2	-178.6(4)	C33	N2	C4	C5	-176.6(3)
C1	C2	C5	C4	178.4(2)	C33	N2	C4	C41	1.3(5)
C1	C2	C5	C6	-1.2(4)	C33	C34	C35	C36	-63.2(14)
C1	C14	C15	C16	-178.9(3)	C33	C34	C35A	C36A	-143.7(8)
C01B	C8	C9	C10	-58.8(4)	C33	C34	C39	C40	109.9(8)
C2	C1	C14	S1	-168.8(2)	C33	C34	C39A	C40A	151.0(9)
C2	C1	C14	C15	9.7(5)	C34	C35	C36	C37	173.8(10)
C2	C5	C6	O1	-179.9(4)	C34	C35A	C36A	C37A	-174.6(9)
C2	C5	C6	N1	0.3(3)	C35	C34	C39	C40	-128.0(11)
C3	N2	C4	C5	-1.5(3)	C35	C36	C37	C38	100.7(17)
C3	N2	C4	C41	176.5(3)	C35A	C34	C39A	C40A	-88.4(11)
C3	N2	C33	C34	102.2(3)	C35A	C36A	C37A	C38A	173.5(10)
C3	C2	C5	C4	-0.4(3)	C39	C34	C35	C36	-169.1(12)
C3	C2	C5	C6	-179.9(2)	C39A	C34	C35A	C36A	88.9(10)

C4	N2	C3	O2	-178.9(3)	C41	S2	C44	C43	-0.6(2)
C4	N2	C3	C2	1.2(3)	C41	S2	C44	C45	175.7(2)
C4	N2	C33	C34	-83.1(4)	C41	C4	C5	C2	-176.8(3)
C4	C5	C6	O1	0.8(7)	C41	C4	C5	C6	2.5(6)
C4	C5	C6	N1	-179.0(4)	C41	C42	C43	C44	-0.5(4)
C4	C41	C42	C43	178.0(3)	C42	C43	C44	S2	0.8(3)
C5	C2	C3	O2	179.7(3)	C42	C43	C44	C45	-175.1(3)
C5	C2	C3	N2	-0.5(3)	C43	C44	C45	C46	14.6(5)
C5	C4	C41	S2	176.1(2)	C43	C44	C45	C50	-169.4(3)
C5	C4	C41	C42	-1.6(5)	C44	S2	C41	C4	-177.6(3)
C6	N1	C1	C2	-1.3(3)	C44	S2	C41	C42	0.4(2)
C6	N1	C1	C14	177.1(3)	C44	C45	C46	C47	173.4(3)
C6	N1	C7	C8	-98.3(3)	C44	C45	C50	C49	-174.4(3)
C7	N1	C1	C2	180.0(3)	C45	C46	C47	C48	0.8(5)
C7	N1	C1	C14	-1.6(5)	C46	C45	C50	C49	1.6(5)
C7	N1	C6	O1	-0.4(5)	C46	C47	C48	C49	2.2(5)
C7	N1	C6	C5	179.4(3)	C46	C47	C48	C51	-174.9(3)
C7	C8	C9	C10	174.7(3)	C47	C48	C49	C50	-3.2(5)
C8	C9	C10	C11	-176.2(3)	C47	C48	C49	C53	177.9(3)
C9	C10	C11	C12	61.9(5)	C47	C48	C51	C52	179.3(4)
C13	C01B	C8	C7	69.3(4)	C47	C48	C51	C54	1.5(6)
C13	C01B	C8	C9	-55.7(4)	C48	C49	C50	C45	1.3(4)
C14	S1	C17	C16	-0.1(2)	C48	C49	C53	C52	4.9(3)
C14	S1	C17	C18	-178.5(2)	C48	C49	C53	C58	125.5(3)
C14	C1	C2	C3	1.1(6)	C48	C49	C53	C59	-111.6(3)
C14	C1	C2	C5	-176.9(3)	C48	C51	C52	C53	1.3(4)
C14	C15	C16	C17	0.1(4)	C48	C51	C52	C57	-178.1(3)

C15	C16	C17	S1	0.0(3)	C48	C51	C54	C55	176.5(3)
C15	C16	C17	C18	178.3(3)	C49	C48	C51	C52	1.9(4)
C16	C17	C18	C19	1.2(5)	C49	C48	C51	C54	-175.8(3)
C16	C17	C18	C23	-177.4(3)	C50	C45	C46	C47	-2.7(5)
C17	S1	C14	C1	178.9(3)	C50	C49	C53	C52	-173.9(3)
C17	S1	C14	C15	0.1(2)	C50	C49	C53	C58	-53.3(4)
C17	C18	C19	C20	-178.1(3)	C50	C49	C53	C59	69.6(4)
C17	C18	C23	C22	177.6(3)	C51	C48	C49	C50	174.5(3)
C18	C19	C20	C21	0.3(5)	C51	C48	C49	C53	-4.5(3)
C19	C18	C23	C22	-0.9(4)	C51	C52	C53	C49	-3.6(3)
C19	C20	C21	C22	-0.7(4)	C51	C52	C53	C58	-124.3(3)
C19	C20	C21	C24	176.3(3)	C51	C52	C53	C59	112.0(3)
C20	C21	C22	C23	0.2(4)	C51	C52	C57	C56	0.9(5)
C20	C21	C22	C26	177.7(3)	C51	C54	C55	C56	1.4(6)
C20	C21	C24	C25	-176.3(3)	C52	C51	C54	C55	-1.0(5)
C20	C21	C24	C27	2.4(5)	C53	C49	C50	C45	-180.0(3)
C21	C22	C23	C18	0.6(4)	C53	C52	C57	C56	-178.4(3)
C21	C22	C26	C25	-1.0(3)	C54	C51	C52	C53	179.3(3)
C21	C22	C26	C31	117.6(3)	C54	C51	C52	C57	-0.1(5)
C21	C22	C26	C32	-118.5(3)	C54	C55	C56	C57	-0.7(6)
C21	C24	C25	C26	-1.6(3)	C55	C56	C57	C52	-0.5(6)
C21	C24	C25	C30	176.6(3)	C57	C52	C53	C49	175.7(3)
C21	C24	C27	C28	-176.6(3)	C57	C52	C53	C58	55.0(5)
C22	C21	C24	C25	1.0(3)	C57	C52	C53	C59	-68.7(4)

Supplementary Table 33. Hydrogen Atom Coordinates (Å×10^4) and Isotropic Displacement Parameters (Å^2×10^3) for **Flu-TDPP**.

Atom	*x*	*y*	*z*	U(eq)

H01A	6907.16	7363.82	5338.57	66
H01B	6015.03	8161.71	5333.22	66
H7A	6430.52	6952.56	4048.18	55
H7B	7512.01	7366.73	4383.13	55
H8	5362.86	7223.43	4698.48	59
H9A	6196.63	8783.49	4278.19	61
H9B	5207.86	8158.19	4012.62	61
H10A	5058.4	9240.47	4837.62	69
H10B	4078	8574.43	4603.69	69
H11A	3665.45	10079.65	4351.61	78
H11B	4799.29	10117.2	4115.87	78
H12A	4058.49	9048.84	3534.82	110
H12B	3217.53	9887.09	3515.4	110
H12C	2933.53	8977.6	3776.33	110
H13A	8121.86	8357.81	4972.95	98
H13B	7914.7	8706.73	5494.04	98
H13C	7246.03	9155.27	5012.82	98
H15	4774.53	4083.6	4534.25	53
H16	2968.57	4174.85	3996.86	54
H19	1478.9	4555.45	3452.76	54
H20	-39.31	4921.98	2856.13	54
H23	3101.31	6840.79	3163.45	52
H27	-1526.52	5633.21	2118.42	58
H28	-2439.7	6428.26	1444.94	65
H29	-1600.27	7697.71	1166.7	63
H30	110.75	8240.75	1578.64	57
H31A	786.75	8334.16	2850.84	101

H31B 1321.99	8833.02	2432.32	101
H31C 2129.91	8422.77	2890.04	101
H32A 3136.03	7560.33	2301.99	95
H32B 2278.07	7933.06	1849.18	95
H32C 2436.52	6874.75	1928.96	95
H33A 7446.13	2500.22	5557.33	56
H33B 8498.28	2925.45	5902.81	56
H34A 9675.27	2472.58	5288.35	81
H34 9695.1	2497.96	5348.73	81
H35A 8479.76	902.42	5576.1	122
H35B 9690.05	882.4	5395.53	122
H35C 9039.71	1706.63	6069.29	119
H35D 8656.18	940.47	5679.23	119
H36A 9328.14	1766.11	6276.75	118
H36B 10537.76	1631.13	6105.07	118
H36C 10565.44	844.9	5533.06	107
H36D 10913.78	1546.57	5959.04	107
H37A 9706.69	477.48	6676.47	152
H37B 9335.96	13.87	6158.95	152
H37C 10115.79	-201.09	6119.36	143
H37D 10607.72	500.42	6533.03	143
H38A 11063.08	-472.98	6146.17	219
H38B 11374.12	-145.93	6696.5	219
H38C 11584.9	505.31	6263.69	219
H38D 12309.52	-142.04	6525.55	212
H38E 12325.01	331.27	6012.22	212
H38F 11866.02	-673.73	6035.07	212

H39A	8132.86	1240.44	4820.57	125
H39B	7382.14	2127.38	4864.34	125
H39C	8065.78	1336.17	4871.71	101
H39D	8063.53	2339.5	4687.34	101
H40A	8503.84	2012.76	4190.98	83
H40B	9517.48	2329.47	4598.02	83
H40C	8429.87	2955.43	4458.57	83
H40D	9718.85	2100.51	4381.59	183
H40E	8786.73	1374.04	4170.96	183
H40F	9792.64	1099.9	4591.83	183
H42	10045.78	5889.37	5549.23	53
H43	11774.98	5802.71	6140.99	53
H46	12914.16	5460.34	6872.42	63
H47	14179.92	5043.38	7556.03	68
H50	12029.42	2855.37	6625.11	53
H54	15346.11	4234.3	8347.78	77
H55	16135.3	3248.51	8963.03	89
H56	15817.22	1717.91	8888.06	93
H57	14679.37	1129.33	8209.51	79
H58A	12067.68	1929.43	7720.41	90
H58B	12701.15	1008.6	7640.66	90
H58C	11965.74	1502.56	7189.58	90
H59A	13643.55	1529.04	6743.58	103
H59B	14353.6	1004.29	7190.03	103
H59C	14818.74	1930.2	7007.95	103

Supplementary Table 34. Atomic Occupancy for **Flu-TDPP**.

Atom	*Occupancy*	Atom	*Occupancy*	Atom	*Occupancy*
H34A	0.383(5)	H34	0.617(5)	C35	0.383(5)
H35A	0.383(5)	H35B	0.383(5)	C35A	0.617(5)
H35C	0.617(5)	H35D	0.617(5)	C36	0.383(5)
H36A	0.383(5)	H36B	0.383(5)	C36A	0.617(5)
H36C	0.617(5)	H36D	0.617(5)	C37	0.383(5)
H37A	0.383(5)	H37B	0.383(5)	C37A	0.617(5)
H37C	0.617(5)	H37D	0.617(5)	C38	0.383(5)
H38A	0.383(5)	H38B	0.383(5)	H38C	0.383(5)
C38A	0.617(5)	H38D	0.617(5)	H38E	0.617(5)
H38F	0.617(5)	C39	0.383(5)	H39A	0.383(5)
H39B	0.383(5)	C39A	0.617(5)	H39C	0.617(5)
H39D	0.617(5)	C40	0.383(5)	H40A	0.383(5)
H40B	0.383(5)	H40C	0.383(5)	C40A	0.617(5)
H40D	0.617(5)	H40E	0.617(5)	H40F	0.617(5)

2.9. X-ray crystallographic data for TPAOMe-TDPP-C4

Supplementary Table 35. Crystal data and structure refinement for TPAOMe-TDPP-C4.

Identification code	TPAOMe-TDPP-C4
Empirical formula	$C_{62}H_{58}N_4O_6S_2$
Formula weight	1019.24
Temperature/K	150.00(10)
Crystal system	monoclinic
Space group	$P2_1/n$
a/Å	14.3751(2)
b/Å	10.10090(10)
c/Å	18.6369(3)

α/°	90
β/°	97.4310(10)
γ/°	90
Volume/Å^3	2683.38(6)
Z	2
ρ_{calc}g/cm^3	1.261
μ/mm^{-1}	1.347
F(000)	1076.0
Crystal size/mm^3	0.1 × 0.06 × 0.01
Radiation	CuKα (λ = 1.54184)
2Θ range for data collection/°	7.326 to 134.148
Index ranges	-16 ≤ h ≤ 17, -12 ≤ k ≤ 12, -20 ≤ l ≤ 22
Reflections collected	13509
Independent reflections	4779 [R_{int} = 0.0221, R_{sigma} = 0.0305]
Data/restraints/parameters	4779/0/337
Goodness-of-fit on F^2	1.071
Final R indexes [I>=2σ (I)]	R_1 = 0.0353, wR_2 = 0.0929
Final R indexes [all data]	R_1 = 0.0414, wR_2 = 0.0961
Largest diff. peak/hole / e Å^{-3}	0.29/-0.25

Supplementary Table 36. Fractional Atomic Coordinates (×10^4) and Equivalent Isotropic Displacement Parameters (Å^2×10^3) for TPAOMe-TDPP-C4. U_{eq} is defined as 1/3 of of the trace of the orthogonalised U_{IJ}tensor.

Atom	*x*	*y*	*z*	U(eq)
S01	5151.2(2)	4234.1(4)	4114.7(2)	30.72(11)
O002	2982.7(7)	-246.3(11)	4461.3(6)	36.3(3)
O003	4280.9(8)	12729.7(12)	1277.6(7)	48.9(3)
O004	10403.4(8)	12589.6(13)	4005.6(7)	51.2(3)
N005	4077.9(8)	1434.3(12)	4452.7(7)	30.3(3)

N006	6953.0(9)	10130.9(13)	3222.0(7)	38.4(3)
C007	5364.0(10)	468.5(15)	5016.3(8)	29.5(3)
C008	6378.3(10)	6352.4(15)	4033.4(8)	30.4(3)
C009	5022.0(10)	1644.0(15)	4690.4(8)	28.9(3)
C00A	3795.3(10)	133.9(15)	4621.6(8)	29.8(3)
C00B	6130.9(10)	12164.3(16)	2811.2(8)	34.6(3)
C00C	5656.7(10)	7243.4(15)	3814.4(8)	31.8(3)
C00D	5539.2(10)	2846.7(14)	4618.4(8)	30.1(3)
C00E	6756.0(11)	8886.3(15)	3496.2(8)	32.9(3)
C00F	7841.5(11)	10740.2(15)	3440.0(9)	33.6(3)
C00G	6215.0(10)	5031.4(15)	4316.0(8)	31.3(3)
C00H	6455.6(11)	3030.2(16)	4926.3(9)	35.9(4)
C00I	7297.0(11)	6762.8(15)	3974.8(8)	32.4(3)
C00J	6285.6(11)	10822.9(15)	2728.8(8)	35.3(4)
C00K	8412.4(11)	11103.9(15)	2925.9(9)	35.2(3)
C00L	5835.7(11)	8488.7(15)	3555.7(8)	33.6(3)
C00M	4961.1(11)	12170.8(17)	1769.1(9)	38.6(4)
C00N	9260.4(11)	11712.2(16)	3134.9(9)	37.0(4)
C00O	7483.6(11)	7997.9(15)	3714.4(8)	34.1(3)
C00P	5481.2(10)	12840.3(16)	2331.2(8)	35.3(3)
C00Q	3367.9(10)	2343.5(16)	4107.6(9)	35.6(4)
C00R	9551.7(11)	11974.1(16)	3859.2(9)	37.2(4)
C00S	6830.4(11)	4252.6(15)	4756.4(9)	36.4(4)
C00T	8140.5(12)	10989.6(17)	4160.5(9)	40.5(4)
C00U	8990.6(12)	11605.2(17)	4376.9(9)	41.6(4)
C00V	5121.6(14)	10829.9(18)	1677.4(10)	48.6(5)
C00W	5782.4(14)	10172.2(17)	2146.0(10)	47.5(4)

C00X	3162.3(12)	2159(2)	3294.8(9)	47.5(4)
C00Y	4104.9(13)	14098.1(19)	1362.4(12)	56.8(5)
C00Z	2382.9(14)	3054(3)	2956.2(12)	69.8(7)
C010	10752.9(16)	12797(3)	4736.9(12)	76.5(7)
C011	2015.5(18)	2677(4)	2178.1(14)	103.7(11)

Supplementary Table 37. Anisotropic Displacement Parameters (Å^2×10^3) for TPAOMe-TDPP-C4. The Anisotropic displacement factor exponent takes the form: $-2\pi^2[h^2a^{*2}U_{11}+2hka^*b^*U_{12}+\ldots]$.

Atom	U_{11}	U_{22}	U_{33}	U_{23}	U_{13}	U_{12}
S01	35.5(2)	26.6(2)	30.3(2)	2.42(14)	5.07(14)	-4.23(14)
O002	32.3(5)	36.5(6)	39.6(6)	5.9(5)	3.1(4)	-6.8(5)
O003	48.2(7)	44.5(7)	50.1(7)	6.4(6)	-8.8(6)	-2.8(6)
O004	42.9(6)	57.0(8)	53.1(8)	2.2(6)	3.4(5)	-13.8(6)
N005	32.7(6)	27.6(7)	30.8(6)	3.6(5)	4.8(5)	-2.5(5)
N006	42.2(7)	29.7(7)	42.0(8)	9.2(6)	0.9(6)	-6.6(6)
C007	32.7(7)	26.9(7)	29.8(7)	-0.2(6)	7.2(6)	-5.2(6)
C008	38.0(8)	27.7(8)	26.2(7)	-0.5(6)	7.1(6)	-4.5(6)
C009	33.6(7)	28.4(8)	25.5(7)	-0.9(6)	7.0(6)	-3.6(6)
C00A	34.2(7)	29.9(8)	26.2(7)	-0.1(6)	7.0(6)	-5.3(6)
C00B	35.6(8)	34.4(8)	34.2(8)	-1.5(7)	5.4(6)	-4.3(7)
C00C	35.4(8)	31.0(8)	29.7(8)	-1.4(6)	6.8(6)	-4.9(6)
C00D	36.8(7)	27.3(8)	27.0(7)	1.3(6)	6.9(6)	-3.3(6)
C00E	42.1(8)	27.9(8)	28.7(7)	2.1(6)	4.8(6)	-5.3(6)
C00F	40.2(8)	24.7(7)	36.3(8)	4.9(6)	6.2(6)	-1.9(6)
C00G	37.2(8)	28.5(8)	29.3(7)	-1.7(6)	8.6(6)	-3.0(6)
C00H	39.4(8)	29.8(8)	38.2(8)	6.4(7)	3.4(7)	-3.5(7)
C00I	36.8(8)	29.2(8)	31.5(8)	2.3(6)	5.9(6)	-1.4(6)
C00J	40.5(8)	31.8(8)	33.5(8)	6.5(6)	5.1(7)	-5.0(7)

C00K	44.8(8)	29.4(8)	31.6(8)	3.9(6)	5.9(6)	2.2(7)
C00L	38.5(8)	29.5(8)	33.0(8)	2.2(6)	4.8(6)	-0.4(6)
C00M	39.0(8)	39.3(9)	37.0(9)	7.4(7)	3.3(7)	-5.4(7)
C00N	42.2(8)	34.4(8)	36.5(9)	7.7(7)	12.4(7)	1.4(7)
C00O	34.9(8)	33.2(8)	35.0(8)	2.4(6)	7.8(6)	-5.1(6)
C00P	35.6(8)	32.2(8)	39.0(9)	1.5(7)	8.2(7)	-0.7(6)
C00Q	31.4(7)	32.9(8)	42.9(9)	6.1(7)	6.2(6)	-1.4(6)
C00R	37.5(8)	30.4(8)	44.0(9)	2.6(7)	6.6(7)	-2.3(7)
C00S	36.1(8)	31.9(8)	40.6(9)	4.1(7)	3.3(7)	-7.0(7)
C00T	47.8(9)	40.5(9)	35.4(9)	2.2(7)	13.7(7)	-7.8(7)
C00U	50.4(9)	42.2(10)	32.5(8)	-3.3(7)	6.9(7)	-6.1(8)
C00V	66.1(11)	37.2(10)	38.8(9)	1.4(7)	-7.7(8)	-11.7(8)
C00W	67.3(11)	28.7(8)	43.9(10)	2.7(7)	-2.7(8)	-4.5(8)
C00X	43.2(9)	56.6(11)	41.8(10)	12.2(9)	1.4(7)	-4.9(8)
C00Y	48.7(10)	47.9(11)	69.1(13)	4.6(9)	-10.4(9)	8.3(9)
C00Z	51.2(11)	86.3(16)	68.8(14)	33.7(13)	-4.3(10)	-0.4(11)
C010	59.6(13)	103(2)	63.4(14)	-10.6(13)	-3.4(11)	-30.3(13)
C011	72.1(16)	156(3)	74.0(17)	49.9(19)	-26.0(13)	-27.0(18)

Supplementary Table 38. Bond Lengths for TPAOMe-TDPP-C4

Atom	Atom	Length/Å	Atom	Atom	Length/Å
S01	C00D	1.7380(15)	C00B	C00P	1.386(2)
S01	C00G	1.7261(15)	C00C	C00L	1.383(2)
O002	C00A	1.2292(17)	C00D	C00H	1.380(2)
O003	C00M	1.3721(19)	C00E	C00L	1.400(2)
O003	C00Y	1.418(2)	C00E	C00O	1.398(2)
O004	C00R	1.3687(19)	C00F	C00K	1.389(2)

O004	C010	1.406(2)	C00F	C00T	1.379(2)
N005	C009	1.3882(18)	C00G	C00S	1.374(2)
N005	C00A	1.4222(19)	C00H	C00S	1.400(2)
N005	C00Q	1.4591(19)	C00I	C00O	1.377(2)
N006	C00E	1.3998(19)	C00J	C00W	1.390(2)
N006	C00F	1.429(2)	C00K	C00N	1.376(2)
N006	C00J	1.423(2)	C00M	C00P	1.382(2)
C007	C007[1]	1.407(3)	C00M	C00V	1.388(3)
C007	C009	1.394(2)	C00N	C00R	1.386(2)
C007	C00A[1]	1.441(2)	C00Q	C00X	1.517(2)
C008	C00C	1.394(2)	C00R	C00U	1.386(2)
C008	C00G	1.465(2)	C00T	C00U	1.384(2)
C008	C00I	1.402(2)	C00V	C00W	1.375(2)
C009	C00D	1.440(2)	C00X	C00Z	1.513(3)
C00B	C00J	1.385(2)	C00Z	C011	1.526(3)

Supplementary Table 39. Bond Angles for TPAOMe-TDPP-C4.

Atom	Atom	Atom	Angle/˚	Atom	Atom	Atom	Angle/˚
C00G	S01	C00D	92.60(7)	C00T	C00F	N006	120.67(14)
C00M	O003	C00Y	116.67(14)	C00T	C00F	C00K	119.06(15)
C00R	O004	C010	117.33(15)	C008	C00G	S01	121.71(11)
C009	N005	C00A	111.45(12)	C00S	C00G	S01	110.28(11)
C009	N005	C00Q	130.08(12)	C00S	C00G	C008	128.01(14)
C00A	N005	C00Q	118.38(12)	C00D	C00H	C00S	113.52(14)

C00E	N006	C00F	119.92(12)	C00O	C00I	C008	121.58(14)
C00E	N006	C00J	121.70(13)	C00B	C00J	N006	120.89(14)
C00J	N006	C00F	118.38(12)	C00B	C00J	C00W	118.33(15)
C007[1]	C007	C00A[1]	108.09(16)	C00W	C00J	N006	120.78(15)
C009	C007	C007[1]	109.62(16)	C00N	C00K	C00F	120.25(15)
C009	C007	C00A[1]	142.30(13)	C00C	C00L	C00E	120.57(14)
C00C	C008	C00G	123.07(13)	O003	C00M	C00P	125.00(15)
C00C	C008	C00I	117.33(14)	O003	C00M	C00V	115.76(15)
C00I	C008	C00G	119.60(13)	C00P	C00M	C00V	119.24(15)
N005	C009	C007	106.52(12)	C00K	C00N	C00R	120.39(15)
N005	C009	C00D	126.47(13)	C00I	C00O	C00E	120.75(14)
C007	C009	C00D	127.01(13)	C00M	C00P	C00B	120.08(15)
O002	C00A	N005	121.63(13)	N005	C00Q	C00X	113.43(14)
O002	C00A	C007[1]	134.04(14)	O004	C00R	C00N	115.50(15)
N005	C00A	C007[1]	104.33(12)	O004	C00R	C00U	124.71(15)
C00J	C00B	C00P	120.97(15)	C00U	C00R	C00N	119.79(15)
C00L	C00C	C008	121.62(14)	C00G	C00S	C00H	113.79(14)
C009	C00D	S01	126.67(11)	C00F	C00T	C00U	121.20(15)
C00H	C00D	S01	109.80(11)	C00T	C00U	C00R	119.30(15)
C00H	C00D	C009	123.47(14)	C00W	C00V	C00M	120.34(16)

N006	C00E	C00L	121.62(14)	C00V	C00W	C00J	120.98(16)
C00O	C00E	N006	120.23(14)	C00Z	C00X	C00Q	112.42(17)
C00O	C00E	C00L	118.15(14)	C00X	C00Z	C011	113.2(2)
C00K	C00F	N006	120.27(14)				

Supplementary Table 40. Hydrogen Atom Coordinates (Å×10^4) and Isotropic Displacement Parameters (Å^2×10^3) for TPAOMe-TDPP-C4.

Atom	*x*	*y*	*z*	U(eq)
H00B	6467.88	12618.53	3194.07	42
H00C	5039.77	6994.35	3842.99	38
H00H	6791.97	2402.12	5218.64	43
H00I	7792.34	6187.56	4115.13	39
H00K	8220.53	10935.57	2438.63	42
H00L	5340.76	9067.07	3420.15	40
H00N	9640.53	11949.06	2788.08	44
H00O	8100.63	8243.57	3683.37	41
H00P	5395.18	13745.04	2387.55	42
H00A	3577.73	3245.18	4207.98	43
H00D	2792.21	2218.22	4319.18	43
H00S	7439.66	4513.44	4926.04	44
H00T	7763.9	10739.57	4507.29	49
H00U	9183.4	11769.57	4864.48	50
H00V	4780.54	10373.93	1297.07	58
H00W	5894.17	9278.86	2071.77	57
H00E	3726.91	2340.76	3077.85	57
H00F	2987.64	1244.41	3191.4	57

H00G	3592.26	14368.21	1010.92	85
H00J	4655.43	14595.57	1293.48	85
H00M	3947.77	14257.17	1840.11	85
H00Q	2611.87	3957.73	2964.36	84
H00R	1868.66	3021.07	3245.33	84
H01A	11353.01	13225.08	4769.21	115
H01B	10819.4	11960.67	4983.11	115
H01C	10324.82	13346.18	4957.87	115
H01D	2526.6	2661.37	1893.7	156
H01E	1558.65	3316.38	1979.99	156
H01F	1730.2	1817.31	2170.97	156

Supplementary Table 41. Solvent masks information for TPAOMe-TDPP-C4.

Number	X	Y	Z	Volume	Electron count	Content
1	0.205	0.556	0.377	11.3	0.1	
2	0.295	0.056	0.123	11.3	0.1	
3	0.705	0.944	0.877	11.3	0.1	
4	0.795	0.444	0.623	11.3	0.1	

2.10. X-ray crystallographic data for Flu-TDPP-C8

Supplementary Table 42. Crystal data and structure refinement for Flu-TDPP-C8.

Identification code	Flu-TDPP-C8
Empirical formula	$C_{60}H_{64}N_2O_2S_2$
Formula weight	909.25
Temperature/K	103(1)
Crystal system	orthorhombic
Space group	$P2_12_12_1$

a/Å	46.2686(12)
b/Å	14.7572(3)
c/Å	8.3418(2)
α/°	90
β/°	90
γ/°	90
Volume/Å^3	5695.7(2)
Z	4
ρ_{calc}g/cm^3	1.060
μ/mm^{-1}	1.147
F(000)	1944.0
Crystal size/mm^3	0.12 × 0.11 × 0.1
Radiation	CuKα (λ = 1.54184)
2Θ range for data collection/°	6.286 to 148.264
Index ranges	-53 ≤ h ≤ 57, -13 ≤ k ≤ 18, -9 ≤ l ≤ 5
Reflections collected	18598
Independent reflections	10160 [R_{int} = 0.0423, R_{sigma} = 0.0587]
Data/restraints/parameters	10160/270/796
Goodness-of-fit on F^2	1.042
Final R indexes [I>=2σ (I)]	R_1 = 0.0963, wR_2 = 0.2599
Final R indexes [all data]	R_1 = 0.1105, wR_2 = 0.2711
Largest diff. peak/hole / e Å^{-3}	0.71/-0.63
Flack parameter	0.487(15)

Supplementary Table 43. Fractional Atomic Coordinates (×10^4) and Equivalent Isotropic Displacement Parameters (Å^2×10^3) for Flu-TDPP-C8. $U_{eq}$is defined as 1/3 of of the trace of the orthogonalised U_{IJ} tensor.

Atom	*x*	*y*	*z*	U(eq)

S1	5527.3(4)	7280.5(12)	9587(2)	37.5(5)
S2	4459.8(4)	7024.7(12)	376(2)	37.5(5)
C48	4068.4(15)	5838(4)	-5296(9)	26.9(14)
C52	3606.6(16)	6213(5)	-6103(8)	27.4(14)
C47	4348.1(15)	5520(4)	-5191(8)	26.6(14)
C49	3937.9(16)	6283(5)	-3999(8)	28.5(15)
C23	6048.4(16)	7988(4)	13935(9)	28.7(15)
C51	3857.5(16)	5827(5)	-6615(8)	28.7(15)
C21	5639.9(16)	8750(5)	15166(10)	37.7(18)
C50	4094.8(16)	6413(5)	-2592(9)	30.8(15)
O1	5238(3)	5381(7)	4068(12)	44(2)
C45	4372.5(16)	6090(5)	-2487(9)	31.4(16)
C19	5609.5(15)	8212(4)	12391(8)	23.9(14)
C26	6360.3(16)	7706(5)	14358(9)	31.5(16)
C20	5487.3(16)	8662(5)	13714(9)	29.2(15)
C53	3632.2(17)	6593(5)	-4392(9)	34.0(16)
C2	4982.9(16)	7461(4)	5648(8)	25.9(15)
C18	5439.0(15)	8104(5)	10954(9)	28.9(15)
C27	6112.2(18)	8831(5)	18086(9)	34.7(17)
C22	5920.1(15)	8410(4)	15235(9)	26.6(14)
C24	6132.4(16)	8462(5)	16548(9)	30.3(15)
C57	3368.1(18)	6235(7)	-7161(10)	42(2)
C46	4499.7(16)	5639(5)	-3806(9)	30.9(15)
C28	6358.1(17)	8772(5)	19109(10)	38.4(18)
C5	4999.1(15)	6883(5)	4287(10)	31.0(16)
C41	4741.9(17)	6747(5)	1639(10)	34.4(17)
C00T	5895.1(16)	7889(5)	12521(9)	31.1(16)

C54	3884.3(18)	5450(5)	-8140(10)	37.2(18)
C58	3410.6(18)	6141(5)	-3268(9)	35.8(17)
C29	6608.6(18)	8375(6)	18600(10)	41.5(19)
C1	5181.5(17)	7191(5)	6780(9)	32.9(16)
C17	5198.0(17)	8581(5)	10463(10)	34.7(16)
C31	6387.4(17)	6644(5)	14326(10)	36.3(17)
C16	5086.3(18)	8295(5)	8963(9)	34.5(17)
C43	4787.4(16)	5736(5)	-481(9)	33.9(16)
C42	4892.8(16)	6030(5)	983(9)	32.8(16)
C25	6389.8(16)	8054(5)	16011(10)	32.0(16)
C15	5244.8(17)	7598(5)	8308(9)	33.2(16)
C4	4803.8(19)	7158(5)	3136(10)	37.2(18)
C44	4545.2(16)	6206(5)	-1020(10)	34.6(17)
C32	6580.3(17)	8106(6)	13183(10)	39.1(18)
C55	3649.7(19)	5478(5)	-9171(11)	41.7(19)
C30	6629.8(18)	8010(5)	17006(10)	35.7(17)
C56	3394(2)	5867(7)	-8635(10)	46(2)
C59	3601.5(18)	7599(5)	-4368(11)	41.0(19)
N1	5272(3)	6305(8)	6310(16)	31(3)
C6	5168(3)	6125(8)	4735(17)	31(3)
O2	4751.8(16)	8944(5)	5878(9)	23.7(17)
C3A	4724(6)	7910(30)	5500(40)	54(8)
C33	4542(2)	8673(8)	2719(15)	33(3)
C7	5443(2)	5669(8)	7224(15)	33(3)
C8	5767(2)	5771(9)	6981(15)	37(3)
N2	4712(2)	8022(6)	3597(12)	23(2)
C35A	4018(3)	9263(11)	2280(30)	40(5)

O2A	4605(4)	8461(10)	6423(17)	43(4)
C34A	4322(3)	8888(11)	2550(20)	35(4)
C9A	5939(4)	4973(13)	7640(30)	53(6)
C10A	6137(4)	5547(18)	8700(30)	67(5)
C13A	6811(5)	5337(18)	10850(30)	72(7)
C12A	6657(5)	5820(20)	9480(50)	114(11)
C14A	7133(5)	5570(30)	10890(60)	95(15)
N2A	4606(4)	7729(12)	3990(20)	32(4)
C11A	6446(5)	5160(30)	8650(40)	109(6)
C33A	4320(4)	8054(10)	3635(19)	30(3)
C36A	3834(3)	8666(12)	1190(20)	36(3)
C37	3655(3)	8310(9)	770(20)	70(5)
C39A	3078(6)	8973(18)	-810(50)	94(13)
C38A	3344(4)	8469(15)	-150(40)	62(8)
C40A	2874(9)	8300(30)	-1640(90)	180(30)
C37A	3537(3)	9072(11)	850(20)	33(3)
C8A	5646(4)	5398(14)	7360(30)	54(5)
C7A	5664(5)	6249(16)	6340(40)	62(7)
C36	3744(3)	9157(10)	1700(30)	82(6)
N1A	5379(5)	6568(17)	5950(30)	47(5)
C6A	5266(5)	6350(15)	4330(30)	27(5)
O1A	5383(4)	5788(12)	3510(20)	48(4)
C3	4817(3)	8245(9)	5220(20)	29(3)
C9	5933(2)	5191(13)	8220(20)	64(5)
C10	6250(3)	5480(20)	8250(20)	116(9)
C11	6409(3)	5263(19)	9750(30)	109(6)
C12	6677(4)	5851(15)	9990(30)	106(7)

C13	6943(3)	5297(18)	10360(30)	113(9)
C14	7127(5)	5740(20)	11670(30)	96(10)
C34	4213(2)	8567(9)	2860(15)	37(3)
C35	4073(2)	9256(10)	1687(18)	52(4)
C38	3324(3)	8274(14)	560(30)	102(9)
C39	3229(4)	9018(15)	-590(30)	109(8)
C40	2922(4)	8902(19)	-1170(30)	103(8)

Supplementary Table 44. Anisotropic Displacement Parameters (Å^2×10^3) for Flu-TDPP-C8. The Anisotropic displacement factor exponent takes the form: $-2\pi^2[h^2a^{*2}U_{11}+2hka^*b^*U_{12}+\ldots]$.

Atom	U_{11}	U_{22}	U_{33}	U_{23}	U_{13}	U_{12}
S1	50.9(11)	27.9(8)	33.6(10)	-5.7(8)	-11.0(9)	4.6(8)
S2	47.0(10)	32.6(9)	32.9(10)	-6.8(8)	-9.0(8)	6.3(8)
C48	36(4)	23(3)	22(3)	-1(3)	-2(3)	-3(3)
C52	41(4)	26(3)	15(3)	7(3)	-2(3)	-3(3)
C47	41(4)	16(3)	22(3)	0(3)	3(3)	0(3)
C49	40(4)	31(3)	15(3)	-5(3)	-5(3)	2(3)
C23	38(4)	14(3)	34(4)	4(3)	-3(3)	-2(3)
C51	41(4)	28(3)	17(3)	3(3)	0(3)	-7(3)
C21	36(4)	38(4)	39(5)	11(3)	3(3)	-3(3)
C50	40(4)	22(3)	31(4)	-4(3)	3(3)	-6(3)
O1	63(7)	32(5)	37(6)	-9(4)	-12(5)	2(5)
C45	39(4)	26(3)	28(4)	4(3)	3(3)	-3(3)
C19	40(4)	17(3)	15(3)	-5(2)	0(3)	-4(3)
C26	39(4)	30(4)	25(4)	0(3)	0(3)	4(3)
C20	38(4)	22(3)	28(4)	5(3)	-2(3)	0(3)
C53	41(4)	31(4)	30(4)	-3(3)	1(3)	1(3)
C2	50(4)	18(3)	10(3)	-3(2)	-1(3)	-5(3)

C18	36(4)	29(3)	22(4)	6(3)	-4(3)	-9(3)
C27	50(4)	24(3)	30(4)	0(3)	-3(3)	1(3)
C22	41(4)	11(3)	28(4)	3(3)	1(3)	-3(2)
C24	42(4)	24(3)	25(4)	6(3)	2(3)	-2(3)
C57	36(4)	57(5)	34(5)	-5(4)	-6(3)	-1(4)
C46	33(4)	30(3)	29(4)	-3(3)	-3(3)	-1(3)
C28	40(2)	39(2)	36(2)	-0.1(13)	-0.7(13)	-0.5(13)
C5	32(4)	27(3)	34(4)	-1(3)	-3(3)	3(3)
C41	46(4)	24(3)	34(4)	0(3)	-2(3)	-8(3)
C00T	40(4)	30(4)	24(4)	-15(3)	-1(3)	1(3)
C54	47(4)	31(4)	33(4)	-12(3)	0(3)	-1(3)
C58	47(4)	30(4)	30(4)	0(3)	-3(3)	-2(3)
C29	42(4)	44(4)	39(5)	11(4)	-6(3)	3(4)
C1	46(4)	29(4)	24(4)	3(3)	-12(3)	5(3)
C17	45(4)	27(3)	32(4)	-7(3)	-8(3)	-5(3)
C31	46(4)	26(3)	37(5)	2(3)	4(3)	3(3)
C16	48(4)	23(3)	33(4)	1(3)	-9(3)	1(3)
C43	39(4)	33(4)	29(4)	-3(3)	-11(3)	4(3)
C42	37(4)	31(4)	30(4)	2(3)	-8(3)	0(3)
C25	38(4)	21(3)	37(4)	-5(3)	-3(3)	0(3)
C15	49(4)	23(3)	28(4)	2(3)	-3(3)	4(3)
C4	55(5)	22(3)	34(4)	-7(3)	-6(4)	2(3)
C44	39(4)	29(4)	37(4)	5(3)	-3(3)	-1(3)
C32	35(4)	50(5)	32(4)	-10(4)	3(3)	6(3)
C55	56(5)	26(4)	43(5)	0(3)	-8(4)	0(3)
C30	43(4)	32(4)	32(4)	0(3)	-4(3)	0(3)
C56	51(5)	57(5)	29(4)	-11(4)	-11(4)	-7(4)

C59	48(4)	31(4)	44(5)	-2(4)	-4(4)	3(3)
N1	38(6)	25(6)	29(6)	-14(4)	-9(5)	10(5)
C6	36(7)	25(6)	31(7)	-16(5)	-2(6)	-4(5)
O2	28(3)	17(2)	26(3)	3(2)	-1(2)	-2(2)
C3A	51(17)	80(20)	33(16)	-27(16)	-22(12)	6(15)
C33	51(5)	21(5)	27(6)	11(4)	-5(4)	0(4)
C7	39(5)	22(5)	38(6)	-11(4)	-13(4)	7(4)
C8	41(5)	42(7)	28(6)	-5(5)	-7(4)	11(4)
N2	40(5)	9(4)	20(5)	4(3)	-5(4)	-9(4)
C35A	49(7)	37(7)	34(10)	9(6)	-7(7)	-7(6)
O2A	65(10)	42(8)	22(7)	-9(6)	1(7)	2(7)
C34A	50(7)	23(7)	31(10)	-4(6)	-5(6)	-7(6)
C9A	74(9)	46(8)	40(11)	19(8)	5(8)	-6(7)
C10A	67(5)	67(5)	66(5)	0.2(14)	-0.4(14)	-0.5(13)
C13A	50(11)	50(13)	115(19)	-38(13)	7(10)	-23(9)
C12A	59(12)	165(19)	120(20)	-14(17)	10(14)	9(13)
C14A	53(11)	60(20)	170(50)	30(30)	-10(14)	-30(12)
N2A	55(8)	18(8)	23(9)	-7(7)	1(6)	-4(6)
C11A	73(6)	145(12)	108(13)	5(13)	-15(6)	18(6)
C33A	55(8)	20(7)	15(8)	-10(5)	2(6)	-2(5)
C36A	36(3)	35(3)	36(4)	0.1(13)	-0.4(13)	1.1(13)
C37	76(8)	39(7)	96(13)	31(7)	6(8)	1(6)
C39A	67(15)	84(18)	130(30)	10(18)	-47(18)	-11(13)
C38A	65(11)	52(10)	69(17)	-11(11)	-25(11)	-7(10)
C40A	110(30)	120(30)	300(80)	-60(40)	-120(40)	10(20)
C37A	33(3)	32(3)	33(3)	0.6(13)	0.5(13)	0.3(13)
C8A	77(9)	43(10)	44(13)	-10(8)	-7(8)	-4(8)

C7A	56(10)	59(12)	71(16)	12(11)	0(9)	5(8)
C36	61(6)	58(8)	128(16)	8(9)	-12(7)	12(6)
N1A	56(10)	46(13)	38(13)	-22(9)	-8(9)	4(9)
C6A	27(5)	27(5)	27(5)	-0.5(14)	-0.4(13)	0.3(13)
O1A	57(10)	34(8)	54(11)	-27(8)	-18(8)	11(8)
C3	31(7)	18(6)	38(8)	6(6)	-4(6)	1(5)
C9	49(6)	103(13)	40(9)	22(8)	5(6)	40(7)
C10	43(6)	210(30)	99(13)	24(14)	7(6)	32(9)
C11	73(6)	145(12)	108(13)	5(13)	-15(6)	18(6)
C12	96(9)	160(15)	62(13)	-79(12)	10(8)	4(9)
C13	89(9)	170(20)	84(14)	-61(14)	8(11)	-3(12)
C14	116(16)	100(20)	77(15)	-23(14)	-11(12)	-2(14)
C34	52(5)	32(6)	29(7)	5(5)	1(5)	4(5)
C35	60(6)	64(9)	32(8)	12(6)	-3(6)	18(5)
C38	84(9)	91(14)	130(20)	0(13)	-22(11)	-15(9)
C39	117(15)	88(14)	120(20)	-14(13)	-32(14)	3(12)
C40	116(15)	100(18)	93(18)	11(15)	-25(13)	-8(13)

Supplementary Table 45. Bond Lengths for Flu-TDPP-C8.

Atom	Atom	Length/Å	Atom	Atom	Length/Å
S1	C18	1.716(8)	C1	N1	1.428(14)
S1	C15	1.751(8)	C1	N1A	1.47(2)
S2	C41	1.727(8)	C17	C16	1.418(11)
S2	C44	1.724(8)	C16	C15	1.377(11)
C48	C47	1.379(10)	C43	C42	1.385(10)
C48	C49	1.402(10)	C43	C44	1.392(10)
C48	C51	1.471(10)	C25	C30	1.388(11)

C52	C51	1.362(10)	C4	N2	1.398(12)
C52	C53	1.537(10)	C4	N2A	1.433(18)
C52	C57	1.414(10)	C55	C56	1.388(12)
C47	C46	1.363(10)	N1	C6	1.424(19)
C49	C50	1.393(10)	N1	C7	1.445(14)
C49	C53	1.522(10)	O2	C3	1.207(17)
C23	C26	1.543(10)	C3A	O2A	1.25(3)
C23	C22	1.384(10)	C3A	N2A	1.40(3)
C23	C00T	1.384(10)	C33	N2	1.441(12)
C51	C54	1.394(10)	C33	C34	1.537(11)
C21	C20	1.409(11)	C7	C8	1.520(11)
C21	C22	1.391(10)	C8	C9	1.548(11)
C50	C45	1.373(11)	N2	C3	1.48(2)
O1	C6	1.273(15)	C35A	C34A	1.532(12)
C45	C46	1.414(10)	C35A	C36A	1.522(12)
C45	C44	1.471(11)	C34A	C33A	1.526(12)
C19	C20	1.407(9)	C9A	C10A	1.527(13)
C19	C18	1.444(9)	C9A	C8A	1.514(12)
C19	C00T	1.409(10)	C10A	C11A	1.540(13)
C26	C31	1.571(10)	C13A	C12A	1.526(13)
C26	C25	1.478(11)	C13A	C14A	1.531(13)
C26	C32	1.532(11)	C12A	C11A	1.536(13)
C53	C58	1.541(11)	N2A	C33A	1.438(19)
C53	C59	1.492(10)	C36A	C37A	1.529(11)
C2	C5	1.422(9)	C37	C36	1.527(12)
C2	C1	1.376(10)	C37	C38	1.543(12)
C2	C3A	1.37(3)	C39A	C38A	1.540(13)

C2	C3	1.435(15)	C39A	C40A	1.539(13)
C18	C17	1.380(10)	C38A	C37A	1.510(12)
C27	C24	1.397(11)	C8A	C7A	1.520(13)
C27	C28	1.425(11)	C7A	N1A	1.44(2)
C22	C24	1.473(10)	C36	C35	1.527(11)
C24	C25	1.408(10)	N1A	C6A	1.49(3)
C57	C56	1.350(12)	C6A	O1A	1.20(3)
C28	C29	1.366(12)	C9	C10	1.527(12)
C5	C4	1.380(11)	C10	C11	1.489(12)
C5	C6	1.415(14)	C11	C12	1.526(12)
C5	C6A	1.46(2)	C12	C13	1.512(13)
C41	C42	1.380(11)	C13	C14	1.532(12)
C41	C4	1.418(11)	C34	C35	1.553(11)
C54	C55	1.386(12)	C38	C39	1.526(13)
C29	C30	1.438(12)	C39	C40	1.508(12)
C1	C15	1.439(11)			

Supplementary Table 46. Bond Angles for Flu-TDPP-C8.

Atom	Atom	Atom	Angle/˚	Atom	Atom	Atom	Angle/˚
C18	S1	C15	92.2(3)	C18	C17	C16	113.9(7)
C44	S2	C41	94.1(4)	C15	C16	C17	112.2(7)
C47	C48	C49	120.9(7)	C42	C43	C44	114.3(7)
C47	C48	C51	131.8(7)	C41	C42	C43	114.3(7)
C49	C48	C51	107.3(6)	C24	C25	C26	111.5(7)
C51	C52	C53	112.2(6)	C30	C25	C26	128.0(7)
C51	C52	C57	118.6(7)	C30	C25	C24	120.4(7)
C57	C52	C53	129.2(7)	C1	C15	S1	125.5(6)

C46	C47	C48	119.5(7)	C16	C15	S1	110.8(6)
C48	C49	C53	112.0(6)	C16	C15	C1	123.7(7)
C50	C49	C48	119.4(7)	C5	C4	C41	128.2(7)
C50	C49	C53	128.6(7)	C5	C4	N2	106.0(7)
C22	C23	C26	110.1(6)	C5	C4	N2A	104.2(9)
C22	C23	C00T	119.7(7)	C41	C4	N2A	124.1(9)
C00T	C23	C26	130.2(7)	N2	C4	C41	124.9(8)
C52	C51	C48	109.0(6)	C45	C44	S2	121.2(6)
C52	C51	C54	122.0(7)	C43	C44	S2	108.4(6)
C54	C51	C48	128.9(7)	C43	C44	C45	130.4(7)
C22	C21	C20	118.0(8)	C54	C55	C56	118.7(8)
C45	C50	C49	119.6(7)	C25	C30	C29	118.8(7)
C50	C45	C46	120.2(7)	C57	C56	C55	122.4(8)
C50	C45	C44	121.4(7)	C1	N1	C7	127.6(11)
C46	C45	C44	118.4(7)	C6	N1	C1	108.9(9)
C20	C19	C18	118.9(6)	C6	N1	C7	123.4(11)
C20	C19	C00T	118.5(6)	O1	C6	C5	135.0(12)
C00T	C19	C18	122.6(6)	O1	C6	N1	118.6(12)
C23	C26	C31	109.9(6)	C5	C6	N1	106.4(9)
C25	C26	C23	101.9(6)	C2	C3A	N2A	109(2)
C25	C26	C31	110.8(6)	O2A	C3A	C2	130(2)
C25	C26	C32	113.7(6)	O2A	C3A	N2A	120(2)
C32	C26	C23	111.8(6)	N2	C33	C34	115.6(9)
C32	C26	C31	108.6(6)	N1	C7	C8	113.9(10)
C19	C20	C21	121.1(7)	C7	C8	C9	110.2(9)
C52	C53	C58	110.9(6)	C4	N2	C33	129.3(10)
C49	C53	C52	99.3(6)	C4	N2	C3	110.9(9)

C49	C53	C58	110.9(6)	C33	N2	C3	119.8(10)
C59	C53	C52	111.6(7)	C36A	C35A	C34A	113.1(11)
C59	C53	C49	112.6(7)	C33A	C34A	C35A	111.9(11)
C59	C53	C58	111.0(7)	C8A	C9A	C10A	113.3(12)
C5	C2	C3	108.2(8)	C9A	C10A	C11A	109.5(12)
C1	C2	C5	109.8(6)	C12A	C13A	C14A	111.2(13)
C1	C2	C3	139.6(9)	C13A	C12A	C11A	110.0(13)
C3A	C2	C5	105.3(12)	C4	N2A	C33A	133.0(14)
C3A	C2	C1	141.4(13)	C3A	N2A	C4	108.1(17)
C19	C18	S1	120.0(5)	C3A	N2A	C33A	118.7(18)
C17	C18	S1	110.9(5)	C12A	C11A	C10A	110.2(13)
C17	C18	C19	129.2(7)	N2A	C33A	C34A	112.6(14)
C24	C27	C28	118.2(7)	C35A	C36A	C37A	112.7(11)
C23	C22	C21	122.0(7)	C36	C37	C38	110.6(11)
C23	C22	C24	108.7(6)	C40A	C39A	C38A	109.8(13)
C21	C22	C24	129.3(7)	C37A	C38A	C39A	112.5(12)
C27	C24	C22	131.2(7)	C38A	C37A	C36A	113.8(11)
C27	C24	C25	121.0(7)	C9A	C8A	C7A	112.3(12)
C25	C24	C22	107.8(6)	N1A	C7A	C8A	110.2(19)
C56	C57	C52	119.3(8)	C37	C36	C35	110.0(10)
C47	C46	C45	120.4(7)	C1	N1A	C6A	110.3(15)
C29	C28	C27	121.1(8)	C7A	N1A	C1	132(2)
C2	C5	C6A	110.4(10)	C7A	N1A	C6A	117(2)
C4	C5	C2	110.1(6)	C5	C6A	N1A	101.6(15)
C4	C5	C6	141.1(8)	O1A	C6A	C5	137.5(19)
C4	C5	C6A	136.6(11)	O1A	C6A	N1A	120.5(19)
C6	C5	C2	107.0(7)	C2	C3	N2	103.0(10)

C42	C41	S2	108.8(6)	O2	C3	C2	135.2(15)
C42	C41	C4	125.1(7)	O2	C3	N2	121.8(12)
C4	C41	S2	126.0(6)	C10	C9	C8	109.3(10)
C23	C00T	C19	120.7(6)	C11	C10	C9	115.3(12)
C55	C54	C51	119.0(8)	C10	C11	C12	112.7(12)
C28	C29	C30	120.4(8)	C13	C12	C11	112.5(12)
C2	C1	C15	128.5(7)	C12	C13	C14	111.5(12)
C2	C1	N1	105.8(8)	C33	C34	C35	107.4(8)
C2	C1	N1A	105.9(10)	C36	C35	C34	110.4(10)
C15	C1	N1A	123.5(10)	C39	C38	C37	109.5(11)
N1	C1	C15	124.4(8)	C40	C39	C38	113.2(12)

Supplementary Table 47. Torsion Angles for Flu-TDPP-C8.

A	B	C	D	Angle/˚	A	B	C	D	Angle/˚
S1	C18	C17	C16	-1.2(8)	C41	S2	C44	C43	0.2(6)
S2	C41	C42	C43	1.1(8)	C41	C4	N2	C33	-0.3(18)
S2	C41	C4	C5	170.0(7)	C41	C4	N2	C3	-179.3(10)
S2	C41	C4	N2	-22.0(13)	C41	C4	N2A	C3A	172.0(19)
S2	C41	C4	N2A	14.4(15)	C41	C4	N2A	C33A	-2(3)
C48	C47	C46	C45	-0.4(10)	C00T	C23	C26	C31	-65.4(10)
C48	C49	C50	C45	-1.2(11)	C00T	C23	C26	C25	177.0(7)
C48	C49	C53	C52	2.3(7)	C00T	C23	C26	C32	55.3(10)
C48	C49	C53	C58	119.0(7)	C00T	C23	C22	C21	-0.6(10)
C48	C49	C53	C59	-115.9(8)	C00T	C23	C22	C24	-177.5(6)
C48	C51	C54	C55	176.6(7)	C00T	C19	C20	C21	-3.2(10)
C52	C51	C54	C55	0.8(11)	C00T	C19	C18	S1	21.0(9)
C52	C57	C56	C55	-1.3(14)	C00T	C19	C18	C17	-159.6(7)

C47	C48	C49	C50	0.8(10)	C54	C55	C56	C57	1.5(13)
C47	C48	C49	C53	178.8(6)	C1	C2	C5	C4	-179.0(7)
C47	C48	C51	C52	178.7(7)	C1	C2	C5	C6	12.6(10)
C47	C48	C51	C54	2.5(13)	C1	C2	C5	C6A	-15.0(13)
C49	C48	C47	C46	0.0(10)	C1	C2	C3A	O2A	22(6)
C49	C48	C51	C52	-2.6(8)	C1	C2	C3A	N2A	-164.4(13)
C49	C48	C51	C54	-178.8(7)	C1	C2	C3	O2	-16(3)
C49	C50	C45	C46	0.9(11)	C1	C2	C3	N2	166.4(10)
C49	C50	C45	C44	-179.5(7)	C1	N1	C6	O1	176.7(13)
C23	C26	C25	C24	1.7(8)	C1	N1	C6	C5	-3.5(15)
C23	C26	C25	C30	-178.8(7)	C1	N1	C7	C8	-89.1(16)
C23	C22	C24	C27	179.1(7)	C1	N1A	C6A	C5	0(2)
C23	C22	C24	C25	-0.6(8)	C1	N1A	C6A	O1A	-173(2)
C51	C48	C47	C46	178.6(7)	C17	C16	C15	S1	1.5(8)
C51	C48	C49	C50	-178.1(6)	C17	C16	C15	C1	-178.9(7)
C51	C48	C49	C53	-0.1(8)	C31	C26	C25	C24	-115.2(7)
C51	C52	C53	C49	-4.0(7)	C31	C26	C25	C30	64.4(10)
C51	C52	C53	C58	-120.8(7)	C42	C41	C4	C5	-7.4(14)
C51	C52	C53	C59	115.0(7)	C42	C41	C4	N2	160.6(9)
C51	C52	C57	C56	0.7(12)	C42	C41	C4	N2A	-163.0(12)
C51	C54	C55	C56	-1.3(12)	C42	C43	C44	S2	0.4(9)
C21	C22	C24	C27	2.4(12)	C42	C43	C44	C45	-179.4(7)
C21	C22	C24	C25	-177.3(7)	C15	S1	C18	C19	-178.9(6)
C50	C49	C53	C52	-179.9(7)	C15	S1	C18	C17	1.7(6)
C50	C49	C53	C58	-63.2(10)	C15	C1	N1	C6	179.4(10)
C50	C49	C53	C59	61.9(11)	C15	C1	N1	C7	-1.2(19)
C50	C45	C46	C47	-0.1(11)	C15	C1	N1A	C7A	-3(3)

C50	C45	C44	S2	-20.3(10)	C15	C1	N1A	C6A	-173.6(14)
C50	C45	C44	C43	159.5(8)	C4	C5	C6	O1	12(3)
C19	C18	C17	C16	179.4(7)	C4	C5	C6	N1	-167.7(11)
C26	C23	C22	C21	178.7(6)	C4	C5	C6A	N1A	166.4(14)
C26	C23	C22	C24	1.7(7)	C4	C5	C6A	O1A	-22(4)
C26	C23	C00T	C19	-179.2(7)	C4	C41	C42	C43	178.9(8)
C26	C25	C30	C29	-178.4(7)	C4	N2	C3	C2	1.5(13)
C20	C21	C22	C23	-0.6(10)	C4	N2	C3	O2	-176.8(12)
C20	C21	C22	C24	175.6(6)	C4	N2A	C33A	C34A	-83(2)
C20	C19	C18	S1	-159.4(5)	C44	S2	C41	C42	-0.8(6)
C20	C19	C18	C17	19.9(11)	C44	S2	C41	C4	-178.5(7)
C20	C19	C00T	C23	2.0(10)	C44	C45	C46	C47	-179.8(6)
C53	C52	C51	C48	4.3(8)	C44	C43	C42	C41	-1.0(10)
C53	C52	C51	C54	-179.2(7)	C32	C26	C25	C24	122.1(7)
C53	C52	C57	C56	179.2(8)	C32	C26	C25	C30	-58.3(10)
C53	C49	C50	C45	-178.8(7)	N1	C1	C15	S1	21.6(13)
C2	C5	C4	C41	-176.9(8)	N1	C1	C15	C16	-158.0(10)
C2	C5	C4	N2	13.3(10)	N1	C7	C8	C9	169.7(13)
C2	C5	C4	N2A	-17.6(11)	C6	C5	C4	C41	-15(2)
C2	C5	C6	O1	174.5(17)	C6	C5	C4	N2	175.4(14)
C2	C5	C6	N1	-5.2(13)	C6	N1	C7	C8	90.3(16)
C2	C5	C6A	N1A	8.5(19)	C3A	C2	C5	C4	17.1(19)
C2	C5	C6A	O1A	-180(3)	C3A	C2	C5	C6A	-179(2)
C2	C1	C15	S1	-172.9(7)	C3A	C2	C1	C15	-28(3)
C2	C1	C15	C16	7.5(13)	C3A	C2	C1	N1A	169(3)
C2	C1	N1	C6	11.1(13)	C3A	N2A	C33A	C34A	104(3)
C2	C1	N1	C7	-169.4(12)	C33	N2	C3	C2	-177.6(10)

C2	C1	N1A	C7A	162(2)	C33	N2	C3	O2	4.2(19)
C2	C1	N1A	C6A	-9(2)	C33	C34	C35	C36	177.2(12)
C2	C3A	N2A	C4	-1(3)	C7	N1	C6	O1	-3(2)
C2	C3A	N2A	C33A	173.7(19)	C7	N1	C6	C5	177.0(12)
C18	S1	C15	C1	178.6(7)	C7	C8	C9	C10	-165.4(16)
C18	S1	C15	C16	-1.8(6)	C8	C9	C10	C11	158(2)
C18	C19	C20	C21	177.2(6)	N2	C33	C34	C35	-173.7(11)
C18	C19	C00T	C23	-178.5(6)	C35A	C34A	C33A	N2A	-176.1(14)
C18	C17	C16	C15	-0.2(10)	C35A	C36A	C37A	C38A	-177(2)
C27	C24	C25	C26	179.5(7)	O2A	C3A	N2A	C4	173(3)
C27	C24	C25	C30	-0.1(11)	O2A	C3A	N2A	C33A	-12(5)
C27	C28	C29	C30	0.4(12)	C34A	C35A	C36A	C37A	-176.7(16)
C22	C23	C26	C31	115.4(6)	C9A	C10A	C11A	C12A	170(2)
C22	C23	C26	C25	-2.1(7)	C9A	C8A	C7A	N1A	173(2)
C22	C23	C26	C32	-123.9(6)	C10A	C9A	C8A	C7A	68(3)
C22	C23	C00T	C19	-0.1(10)	C13A	C12A	C11A	C10A	122(3)
C22	C21	C20	C19	2.6(10)	C14A	C13A	C12A	C11A	138(3)
C22	C24	C25	C26	-0.8(8)	C36A	C35A	C34A	C33A	-72(2)
C22	C24	C25	C30	179.6(7)	C37	C36	C35	C34	-75.4(17)
C24	C27	C28	C29	0.6(11)	C37	C38	C39	C40	166(2)
C24	C25	C30	C29	1.1(11)	C39A	C38A	C37A	C36A	-167(3)
C57	C52	C51	C48	-177.0(7)	C40A	C39A	C38A	C37A	-173(4)
C57	C52	C51	C54	-0.5(11)	C8A	C9A	C10A	C11A	-169(3)
C57	C52	C53	C49	177.5(8)	C8A	C7A	N1A	C1	89(3)
C57	C52	C53	C58	60.7(10)	C8A	C7A	N1A	C6A	-101(3)
C57	C52	C53	C59	-63.6(10)	C7A	N1A	C6A	C5	-172(2)
C46	C45	C44	S2	159.4(6)	C7A	N1A	C6A	O1A	14(4)

C46	C45	C44	C43	-20.8(12)	C36	C37	C38	C39	69(2)
C28	C27	C24	C22	179.6(7)	N1A	C1	C15	S1	-12.0(17)
C28	C27	C24	C25	-0.8(11)	N1A	C1	C15	C16	168.5(14)
C28	C29	C30	C25	-1.2(12)	C6A	C5	C4	C41	25(2)
C5	C2	C1	C15	177.9(8)	C6A	C5	C4	N2A	-175.6(17)
C5	C2	C1	N1	-14.5(10)	C3	C2	C5	C4	-12.7(10)
C5	C2	C1	N1A	14.3(13)	C3	C2	C5	C6	178.9(10)
C5	C2	C3A	O2A	177(4)	C3	C2	C1	C15	18.2(17)
C5	C2	C3A	N2A	-9(3)	C3	C2	C1	N1	-174.2(13)
C5	C2	C3	O2	-175.6(15)	C9	C10	C11	C12	-159(2)
C5	C2	C3	N2	6.5(11)	C10	C11	C12	C13	-130(2)
C5	C4	N2	C33	169.9(11)	C11	C12	C13	C14	-139(2)
C5	C4	N2	C3	-9.0(12)	C34	C33	N2	C4	86.4(16)
C5	C4	N2A	C3A	12(2)	C34	C33	N2	C3	-94.7(14)
C5	C4	N2A	C33A	-162.5(19)	C38	C37	C36	C35	-171.7(13)
C41	S2	C44	C45	-179.9(6)					

Supplementary Table 48. Hydrogen Atom Coordinates (Å×10^4) and Isotropic Displacement Parameters (Å^2×10^3) for Flu-TDPP-C8.

Atom	*x*	*y*	*z*	U(eq)
H47	4432.69	5226.75	-6058.44	32
H21	5555.96	9026.56	16053.25	45
H50	4012.03	6716.86	-1730.22	37
H20	5302.54	8905.83	13627.94	35
H27	5942.68	9106.83	18433.83	42
H57	3194.94	6500.92	-6845.16	51
H46	4687.94	5422.87	-3727.53	37
H28	6348	9008.28	20141.41	46

H00T	5981.55	7606.93	11646.88	37
H54	4057.03	5182.83	-8459.79	45
H58A	3220.87	6191.86	-3725.63	54
H58B	3414.06	6437.91	-2244.39	54
H58C	3458.77	5513.07	-3135.16	54
H29	6766.31	8340.87	19288.16	50
H17	5116.29	9046.81	11062.05	42
H31A	6279.32	6391.11	15199.84	54
H31B	6586.98	6475.99	14427.11	54
H31C	6312.5	6417.75	13330.17	54
H16	4924.68	8549.97	8476.99	41
H43	4871.43	5264.82	-1056.61	41
H42	5051.65	5766.49	1482.24	39
H32A	6531.71	7924.64	12110.75	59
H32B	6770.08	7887.9	13444.69	59
H32C	6577.16	8754.68	13256.2	59
H55	3663.2	5241.59	-10202.13	50
H30	6801.15	7750.1	16648.82	43
H56	3235	5873.05	-9316.52	55
H59A	3742.99	7864.8	-5065.45	62
H59B	3630.23	7817.04	-3295.3	62
H59C	3411.33	7762.43	-4726.5	62
H33A	4594.41	8632.93	1594.66	40
H33B	4594.25	9275.18	3085.93	40
H7A	5386.99	5058.29	6927.47	39
H7B	5399.84	5747.36	8353.47	39
H8A	5821.11	6402.13	7097.94	44

H8B	5818.28	5578.1	5905.92	44
H35A	3921.4	9323.49	3308.9	48
H35B	4032.17	9862	1810.75	48
H34A	4407.34	8729.17	1527.7	42
H34B	4441.73	9354	3037.13	42
H9AA	6033.07	4877.2	6617.29	64
H9AB	5912.9	4384.74	8142	64
H10A	6065.62	5543.81	9796.37	80
H10B	6137.78	6168.15	8323.28	80
H13A	6788.68	4686.74	10738.19	86
H13B	6723.07	5514.52	11862.36	86
H12A	6798.13	6028.83	8703.49	137
H12B	6552.09	6335.86	9882.5	137
H14A	7219.45	5411.18	9879.57	142
H14B	7226.01	5247.97	11736.2	142
H14C	7155.56	6213.86	11058.47	142
H11A	6450.32	4578.17	9196.68	130
H11B	6504.24	5066.36	7550.16	130
H33C	4222.76	8203.11	4630.31	36
H33D	4211.2	7575.4	3115.4	36
H36A	3810.09	8077.44	1693.39	43
H36B	3935.54	8576.31	187.24	43
H37A	3720.12	7774.56	1345.35	85
H37B	3747.43	8312.82	-271.49	85
H39A	2977.21	9271.11	67.19	113
H39B	3138.98	9433.11	-1564.19	113
H38A	3280.49	7961.21	498.13	75

H38B	3455.28	8229.32	-1040.61	75
H40A	2678.61	8412.21	-1307.06	264
H40B	2927.08	7690.36	-1347.66	264
H40C	2888.72	8367.98	-2779.27	264
H37C	3441.03	9195	1862.68	39
H37D	3561.83	9645.14	299.91	39
H8AA	5559.6	5549.67	8387.49	65
H8AB	5521.19	4961.2	6836.78	65
H7AA	5768.41	6715.95	6915.57	75
H7AB	5768.83	6117.03	5358.8	75
H36C	3676.04	9112.51	2799.28	99
H36D	3656.11	9687.06	1217.41	99
H9A	5848.15	5270.35	9277.03	77
H9B	5919.61	4554.86	7938.19	77
H10C	6348.34	5198.56	7359.84	139
H10D	6258	6133.97	8086.14	139
H11C	6466.67	4631.33	9724.91	130
H11D	6280.73	5344.3	10661.38	130
H12C	6710.91	6200.93	9022.95	127
H12D	6642.26	6273.07	10859.19	127
H13C	7058.56	5230.52	9398.88	136
H13D	6885.68	4696.86	10713.35	136
H14D	7327.94	5675.65	11416.78	144
H14E	7087.55	5458.56	12684.24	144
H14F	7079.87	6376.08	11738.03	144
H34C	4150.9	8689.5	3950.19	45
H34D	4155.81	7954.57	2582.11	45

H35C 4124.84	9867.36	2000.02	62
H35D 4144.77	9151.3	611.38	62
H38C 3267.87	7686.65	145.3	123
H38D 3230.71	8357.62	1594.05	123
H39C 3246.01	9600.18	-57.92	131
H39D 3357.7	9022.65	-1507.54	131
H40D 2873.13	9394.9	-1872.56	155
H40E 2793.38	8898.51	-271.02	155
H40F 2905.73	8340.05	-1743.23	155

Supplementary Table 49. Atomic Occupancy for Flu-TDPP-C8.

Atom	*Occupancy*	Atom	*Occupancy*	Atom	*Occupancy*
O1	0.612(7)	N1	0.612(7)	C6	0.612(7)
O2	0.612(7)	C3A	0.388(7)	C33	0.612(7)
H33A	0.612(7)	H33B	0.612(7)	C7	0.612(7)
H7A	0.612(7)	H7B	0.612(7)	C8	0.612(7)
H8A	0.612(7)	H8B	0.612(7)	N2	0.612(7)
C35A	0.388(7)	H35A	0.388(7)	H35B	0.388(7)
O2A	0.388(7)	C34A	0.388(7)	H34A	0.388(7)
H34B	0.388(7)	C9A	0.388(7)	H9AA	0.388(7)
H9AB	0.388(7)	C10A	0.388(7)	H10A	0.388(7)
H10B	0.388(7)	C13A	0.388(7)	H13A	0.388(7)
H13B	0.388(7)	C12A	0.388(7)	H12A	0.388(7)
H12B	0.388(7)	C14A	0.388(7)	H14A	0.388(7)
H14B	0.388(7)	H14C	0.388(7)	N2A	0.388(7)
C11A	0.388(7)	H11A	0.388(7)	H11B	0.388(7)
C33A	0.388(7)	H33C	0.388(7)	H33D	0.388(7)

C36A	0.388(7)	H36A	0.388(7)	H36B	0.388(7)
C37	0.612(7)	H37A	0.612(7)	H37B	0.612(7)
C39A	0.388(7)	H39A	0.388(7)	H39B	0.388(7)
C38A	0.388(7)	H38A	0.388(7)	H38B	0.388(7)
C40A	0.388(7)	H40A	0.388(7)	H40B	0.388(7)
H40C	0.388(7)	C37A	0.388(7)	H37C	0.388(7)
H37D	0.388(7)	C8A	0.388(7)	H8AA	0.388(7)
H8AB	0.388(7)	C7A	0.388(7)	H7AA	0.388(7)
H7AB	0.388(7)	C36	0.612(7)	H36C	0.612(7)
H36D	0.612(7)	N1A	0.388(7)	C6A	0.388(7)
O1A	0.388(7)	C3	0.612(7)	C9	0.612(7)
H9A	0.612(7)	H9B	0.612(7)	C10	0.612(7)
H10C	0.612(7)	H10D	0.612(7)	C11	0.612(7)
H11C	0.612(7)	H11D	0.612(7)	C12	0.612(7)
H12C	0.612(7)	H12D	0.612(7)	C13	0.612(7)
H13C	0.612(7)	H13D	0.612(7)	C14	0.612(7)
H14D	0.612(7)	H14E	0.612(7)	H14F	0.612(7)
C34	0.612(7)	H34C	0.612(7)	H34D	0.612(7)
C35	0.612(7)	H35C	0.612(7)	H35D	0.612(7)
C38	0.612(7)	H38C	0.612(7)	H38D	0.612(7)
C39	0.612(7)	H39C	0.612(7)	H39D	0.612(7)
C40	0.612(7)	H40D	0.612(7)	H40E	0.612(7)
H40F	0.612(7)				

Supplementary Table 50. Solvent masks information for Flu-TDPP-C8.

Number	X	Y	Z	Volume	Electron count	Content
1	0.250	0.504	-0.937	601.7	123.0	

2	0.750	0.807	-0.932	601.7	123.0

2.11. ^{1}H, ^{13}C NMR and mass spectra

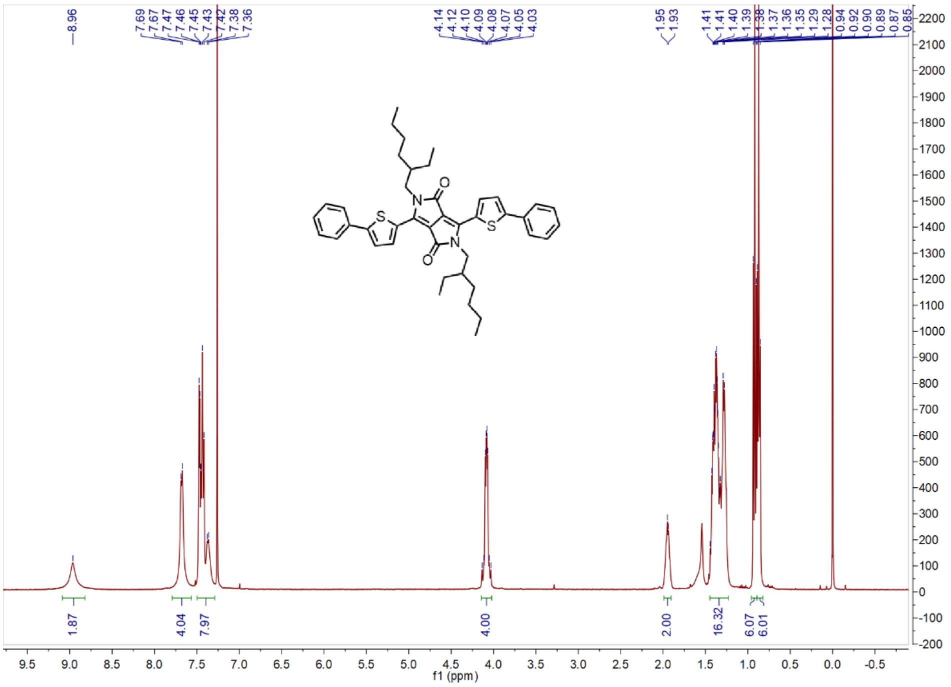

Supplementary Figure 34. ^{1}H NMR of Ph-TDPP in Chloroform-*d*.

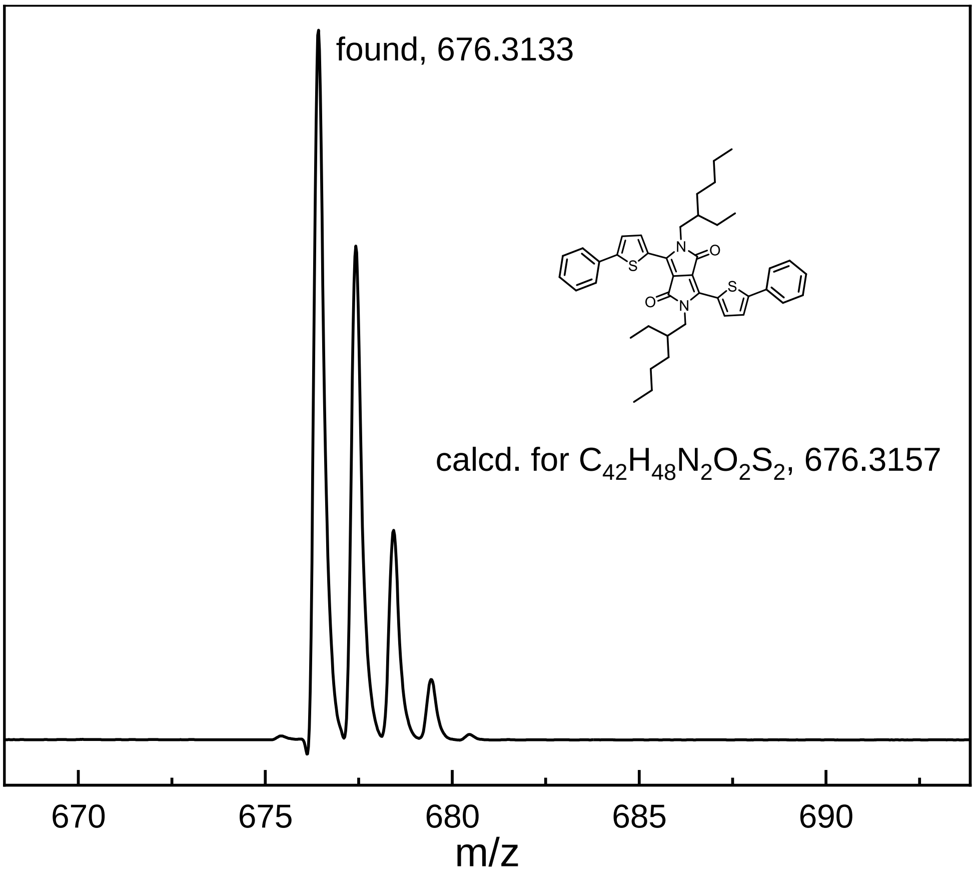

Supplementary Figure 35. MALDI-TOF-MS of Ph-TDPP. Calcd. for $C_{42}H_{48}N_2O_2S_2$: *m/z*: 676.3157. Found: 676.3426.

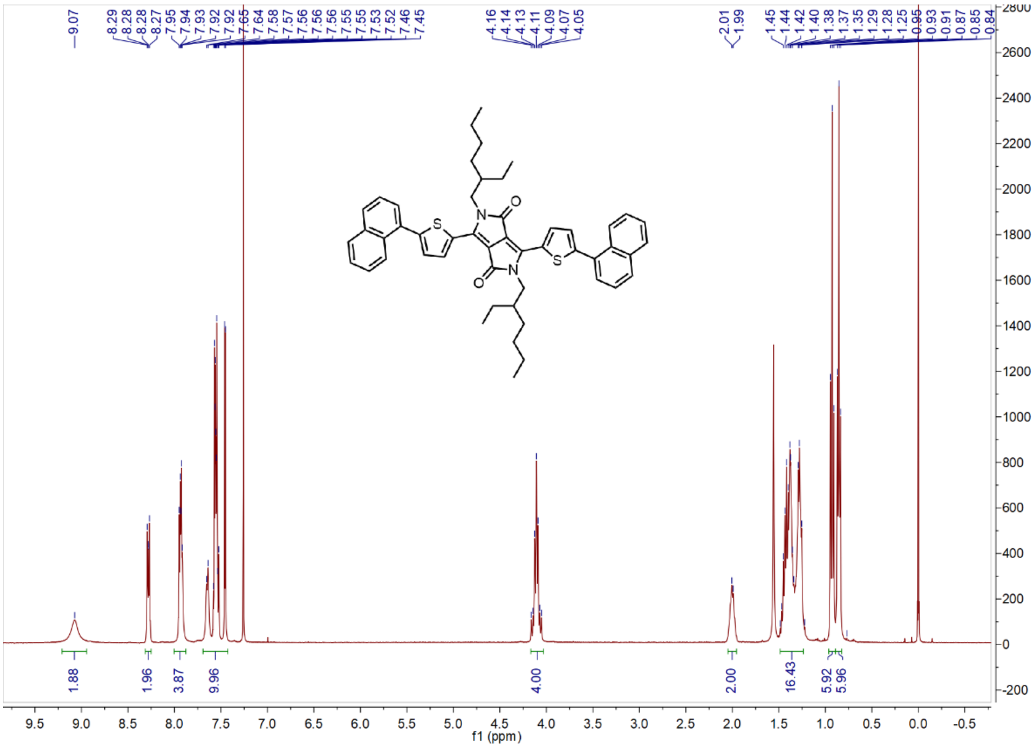

Supplementary Figure 36. ^{13}C NMR of 1N-TDPP in Chloroform-*d*.

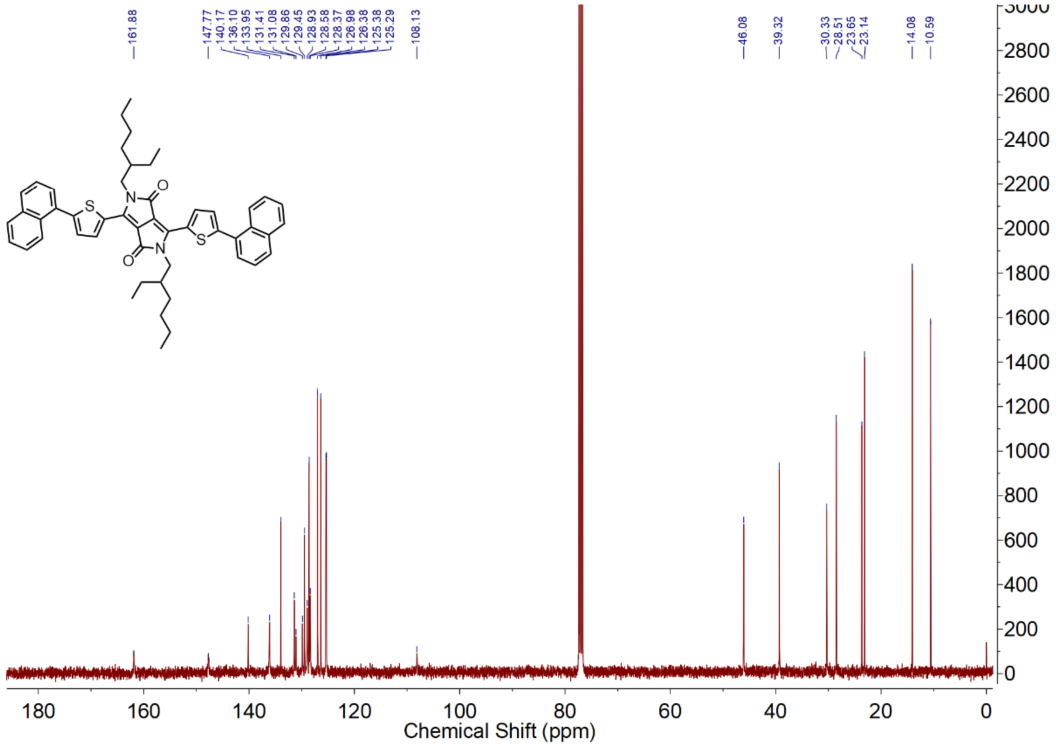

Supplementary Figure 37. ^{13}C NMR of 1N-TDPP in Chloroform-*d*.

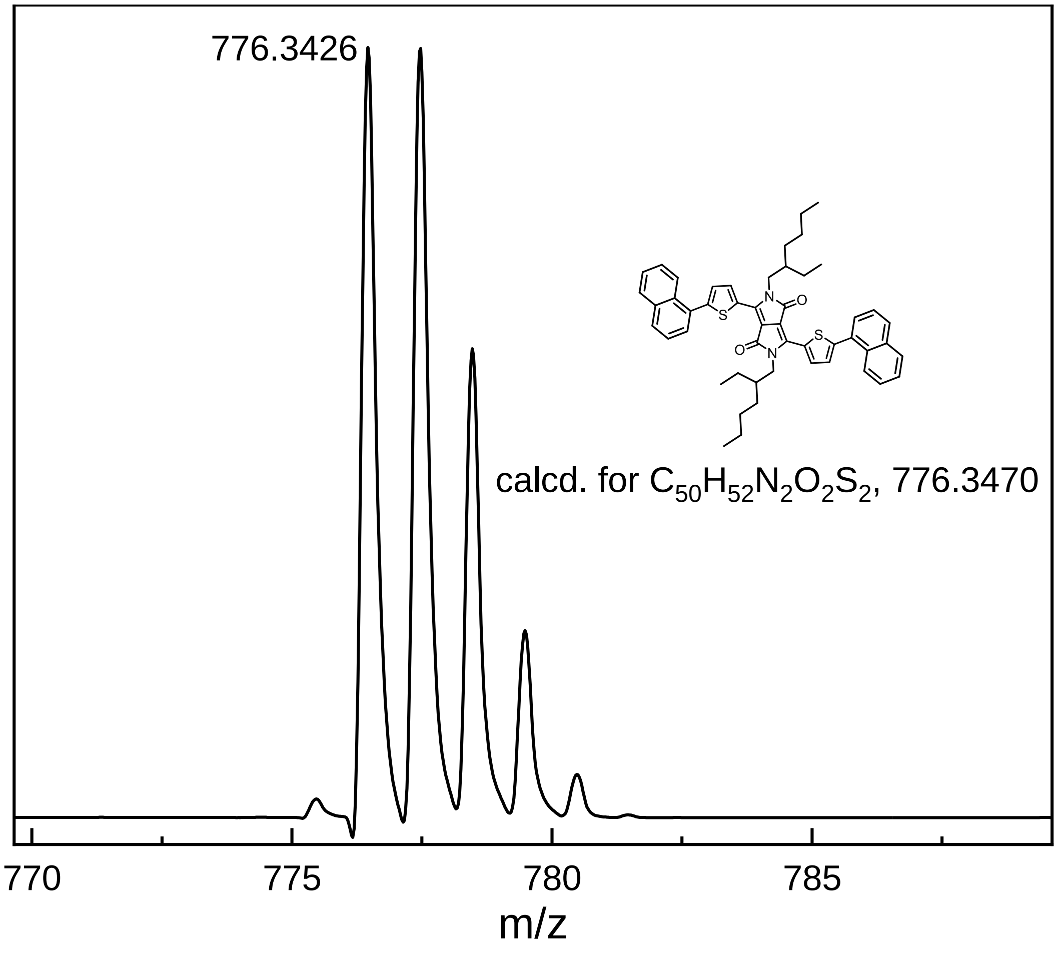

Supplementary Figure 38. MALDI-TOF-MS of 1N-TDPP. Calcd. for $C_{50}H_{52}N_2O_2S_2$: *m/z*: 776.3470. Found: 776.3426.

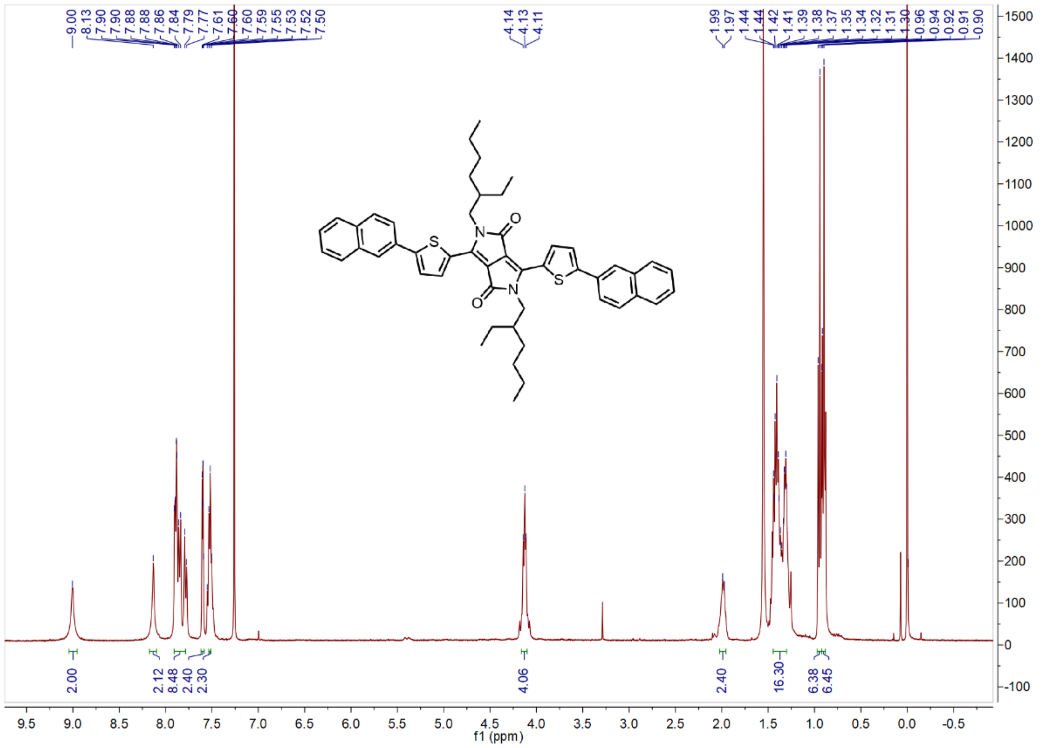

Supplementary Figure 39. ^{1}H NMR of 2N-TDPP in Chloroform-*d*.

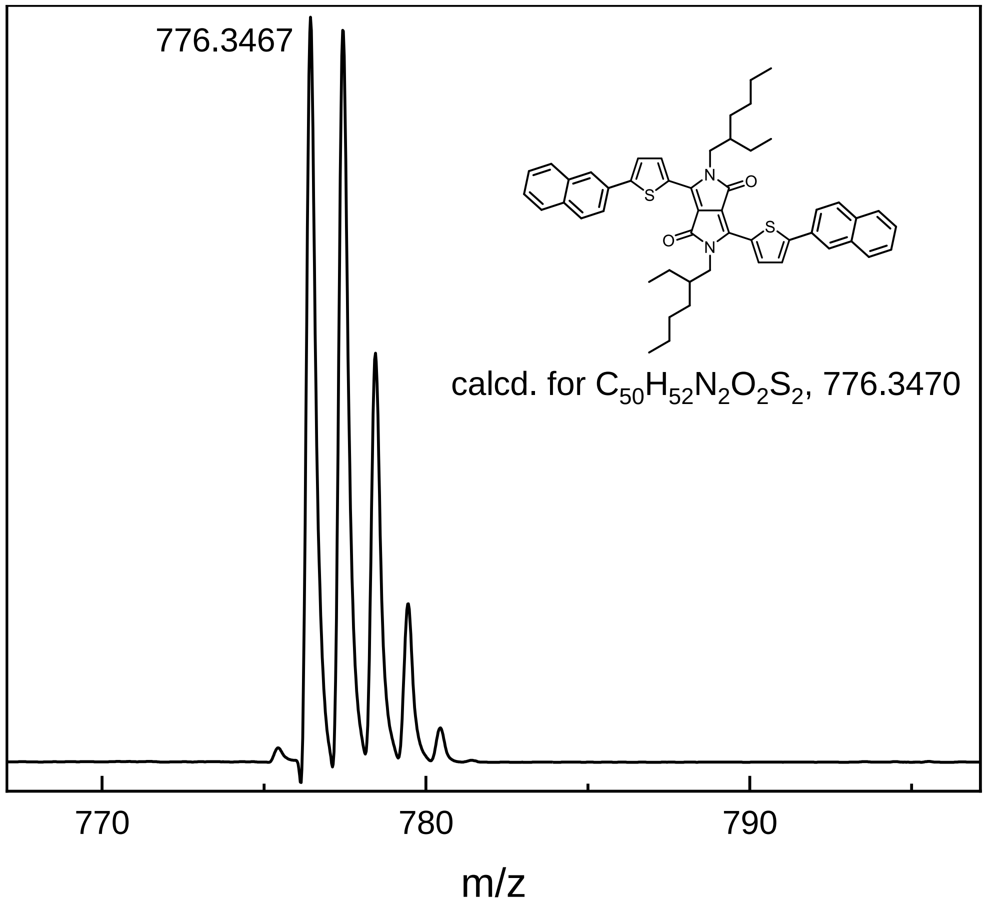

Supplementary Figure 40. MALDI-TOF-MS of 2N-TDPP. Calcd for $C_{50}H_{52}N_2O_2S_2$: *m/z*: 776.3470. Found: 776.3467.

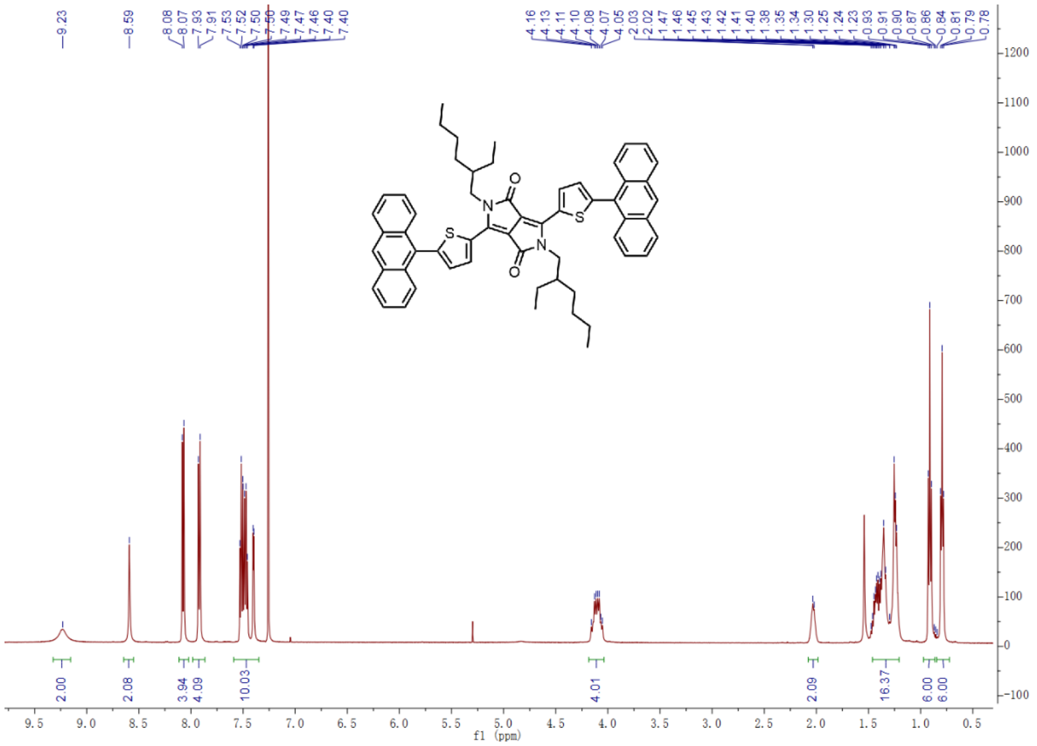

Supplementary Figure 41. ^{1}H NMR of An-TDPP in Chloroform-*d*.

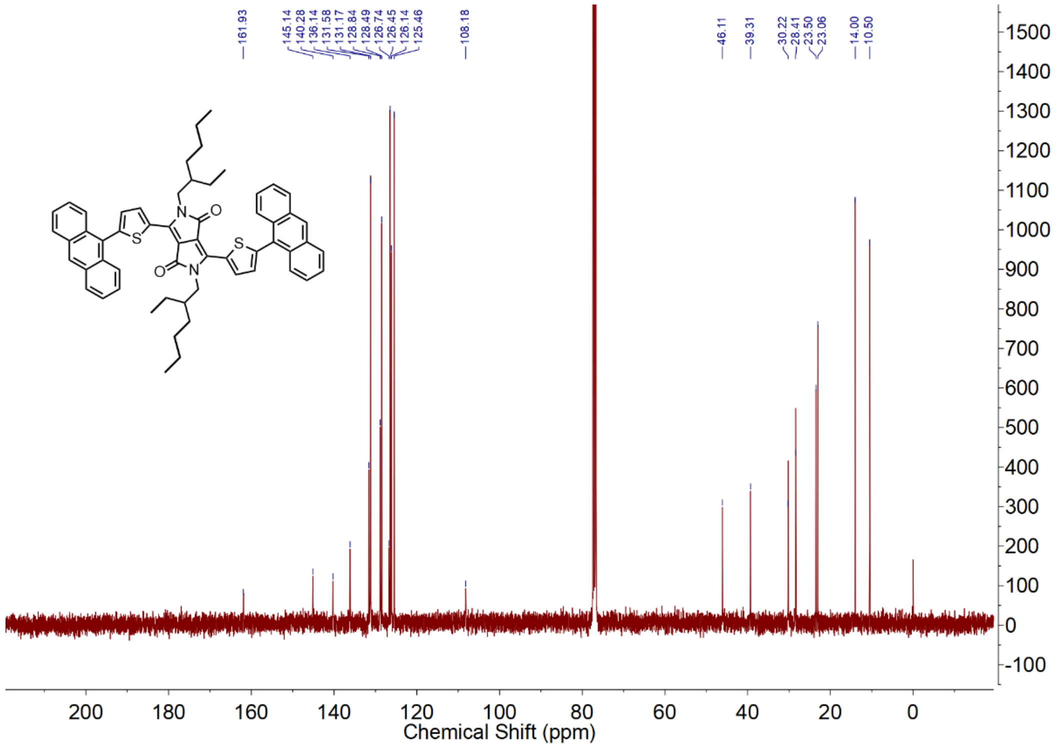

Supplementary Figure 42. ^{13}C NMR of An-TDPP in Chloroform-*d*.

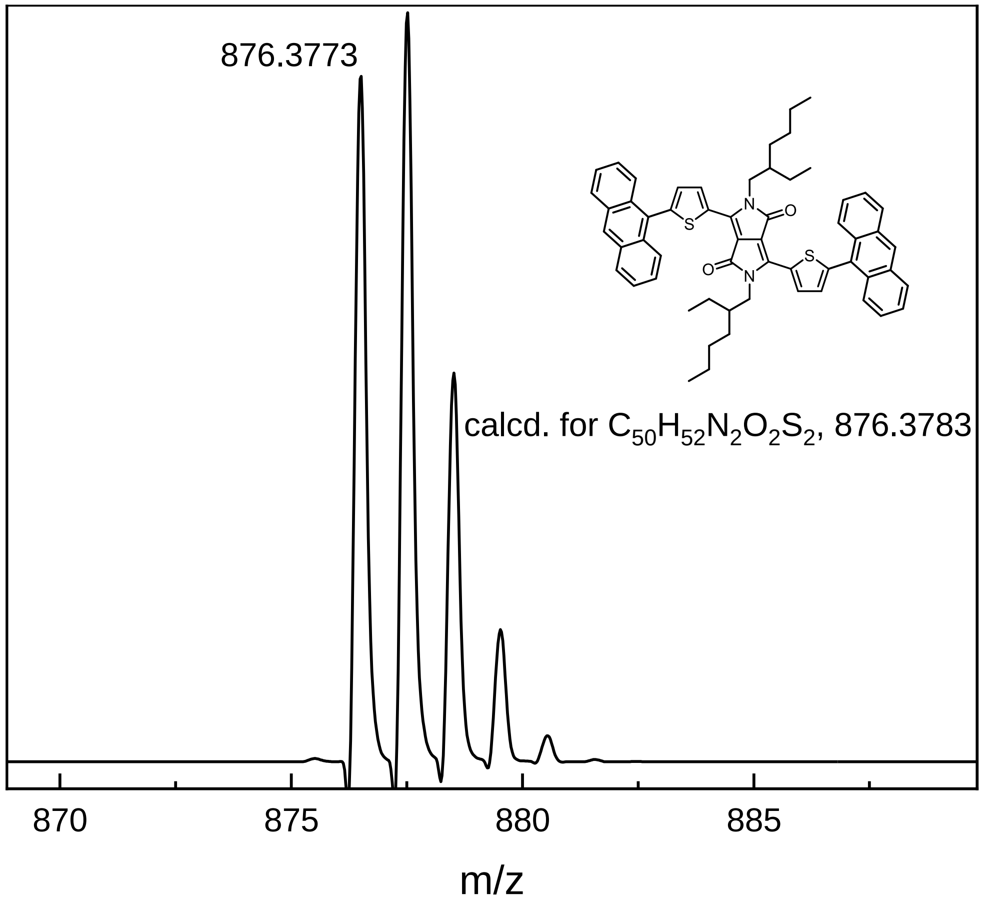

Supplementary Figure 43. MALDI-TOF-MS of An-TDPP. Calcd for $C_{58}H_{56}N_2O_2S_2$: *m/z*: 876.3783. Found:

876.3773.

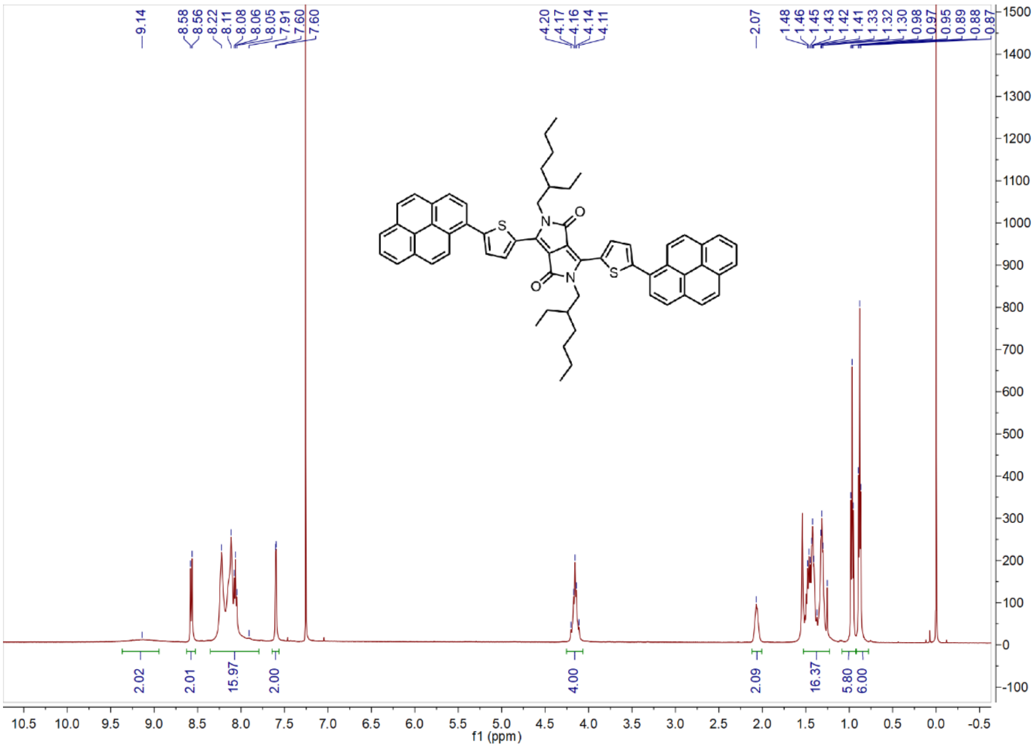

Supplementary Figure 44. ^{1}H NMR of Py-TDPP in Chloroform-*d*.

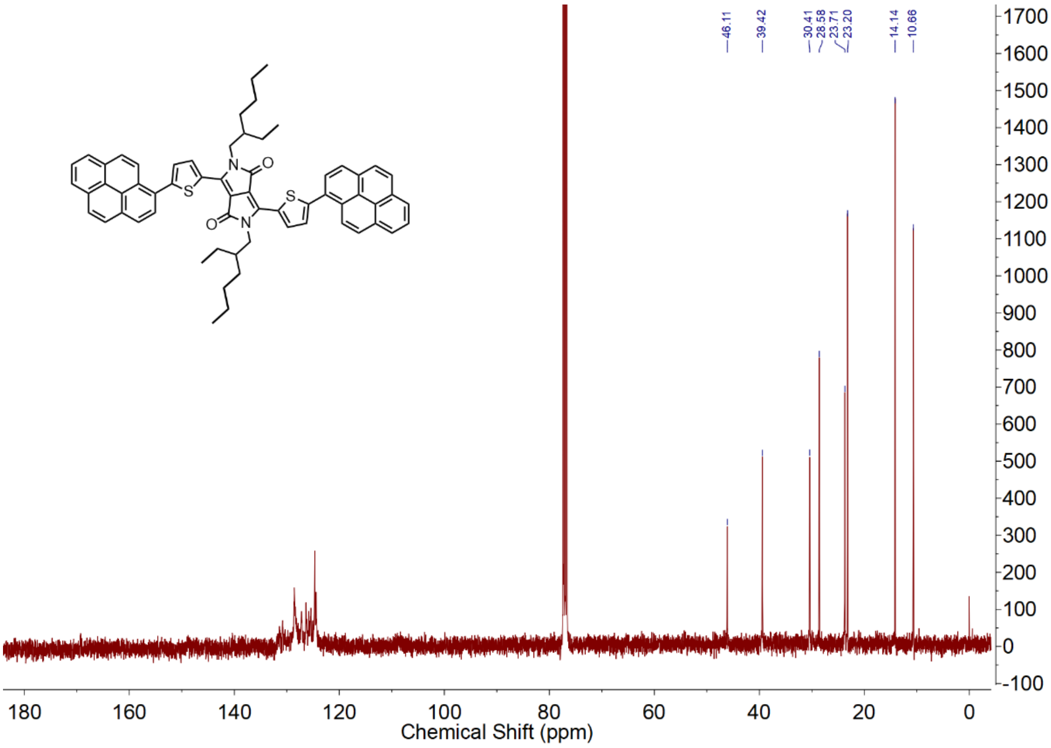

Supplementary Figure 45. ^{13}C NMR of Py-TDPP in Chloroform-*d* (saturated solution).

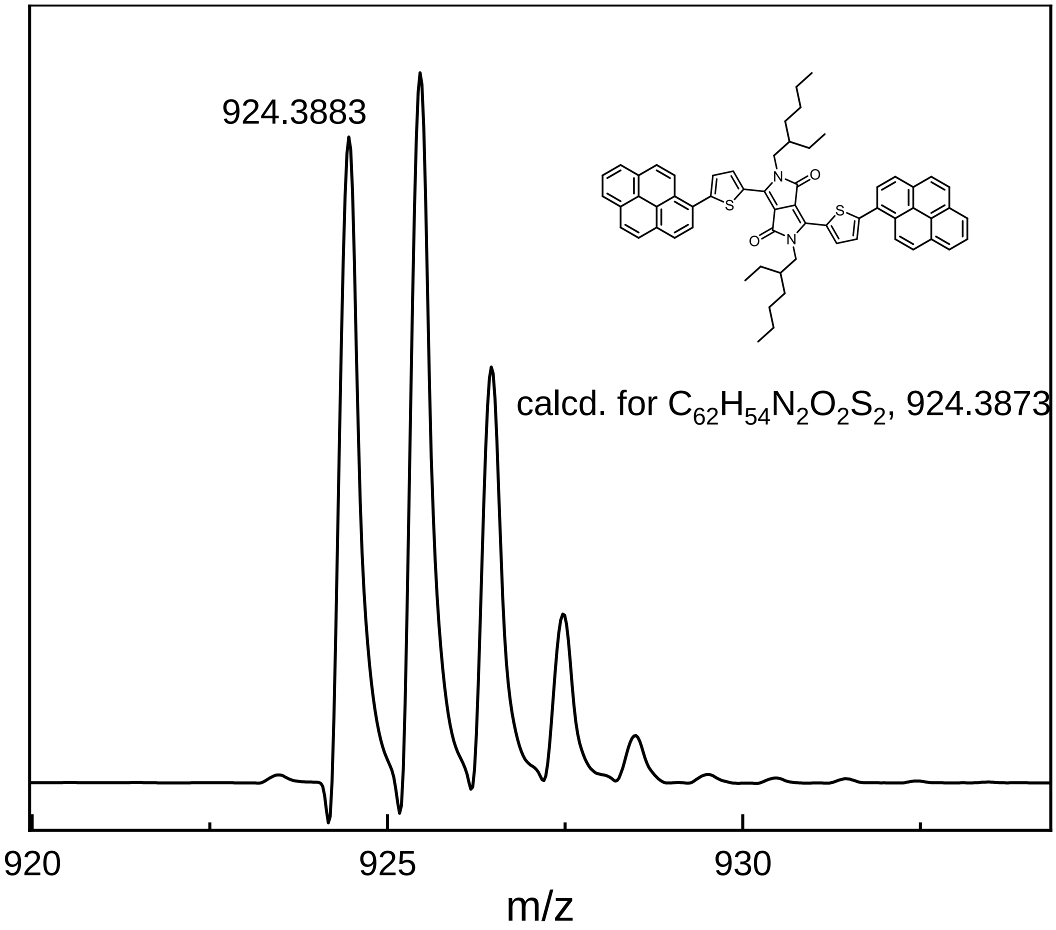

Supplementary Figure 46. MALDI-TOF-MS of Py-TDPP. Calcd. for $C_{62}H_{56}N_2O_2S_2$: *m/z*: 924.3873. Found: 924.3883.

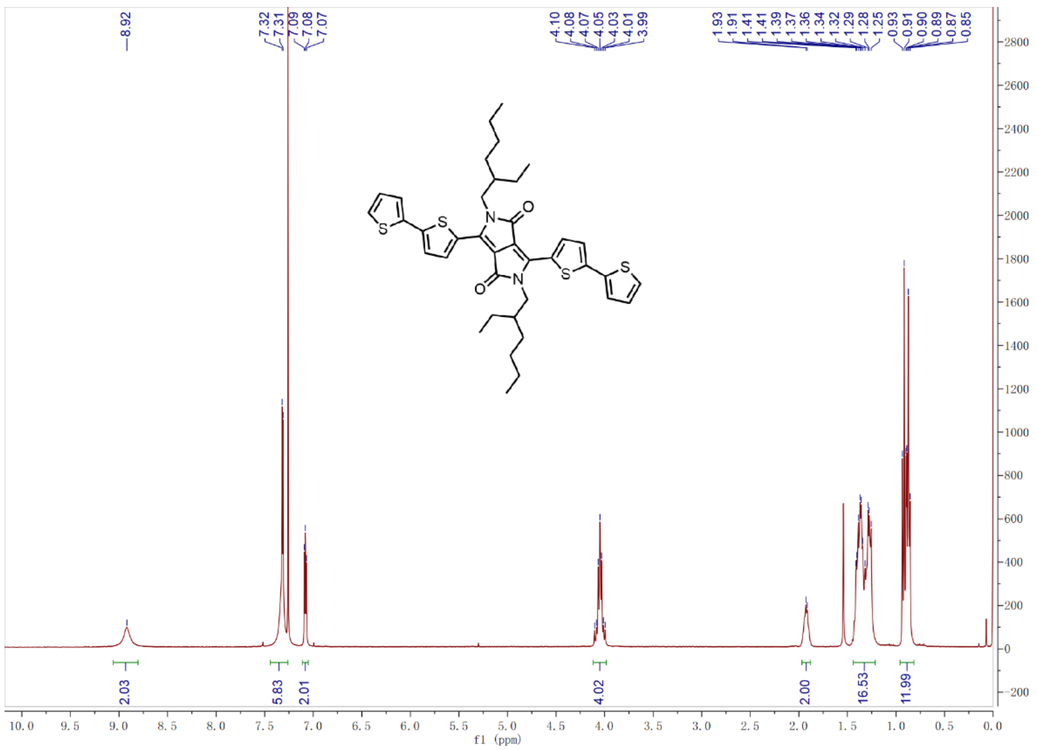

Supplementary Figure 47. ^{1}H NMR of Th-TDPP in Chloroform-*d*.

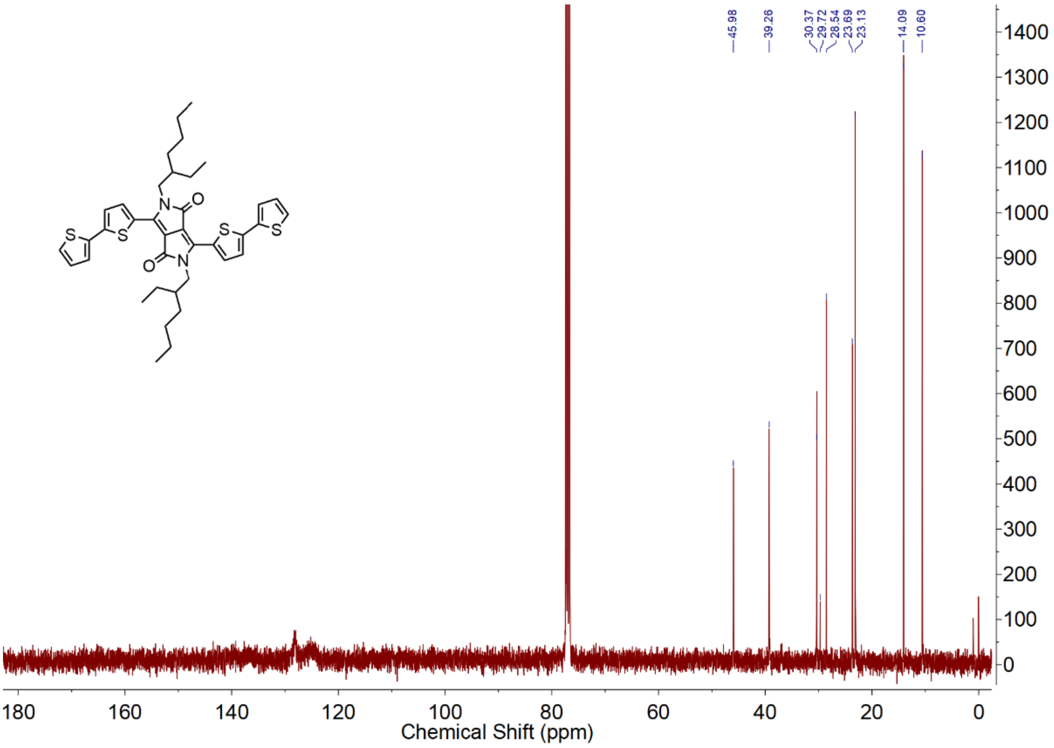

Supplementary Figure 48. ^{13}C NMR of Th-TDPP in Chloroform-*d* (saturated solution).

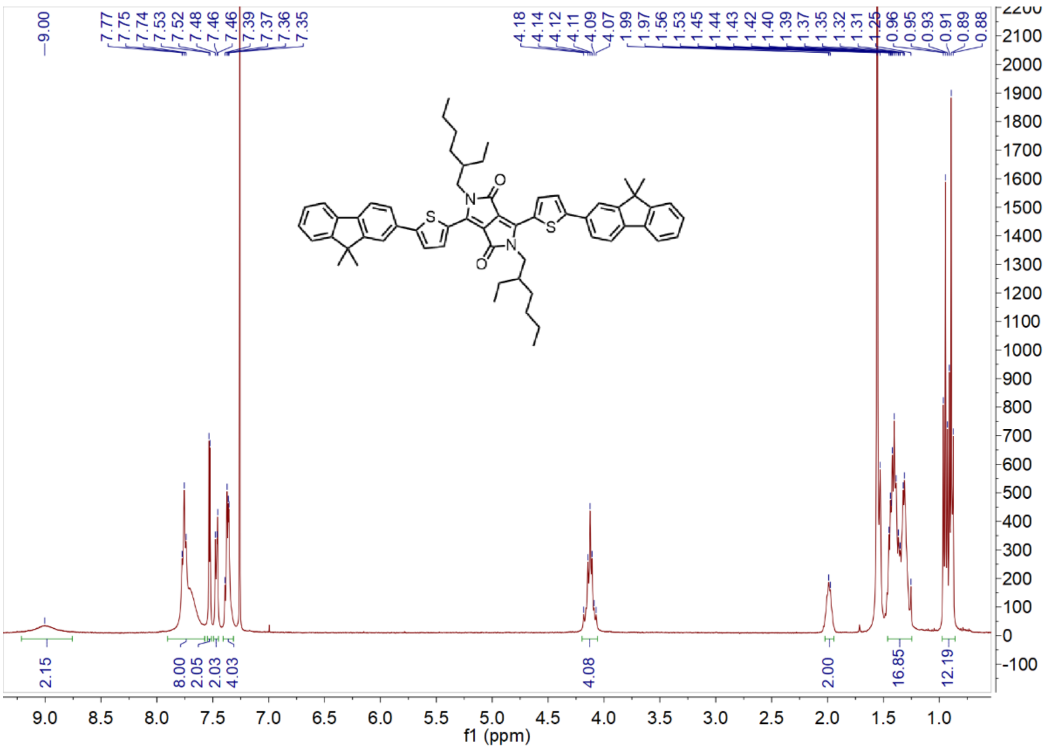

Supplementary Figure 49. ^{1}H NMR of Flu-TDPP in Chloroform-*d*.

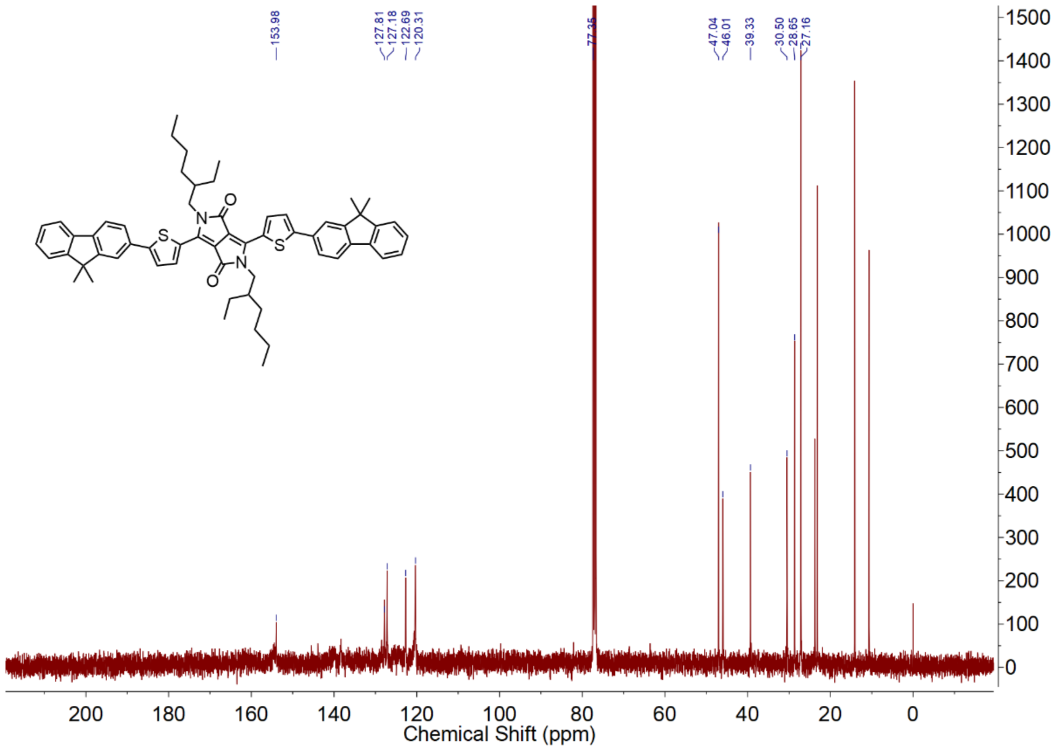

Supplementary Figure 50. ^{13}C NMR of Flu-TDPP in Chloroform-*d* (saturated solution).

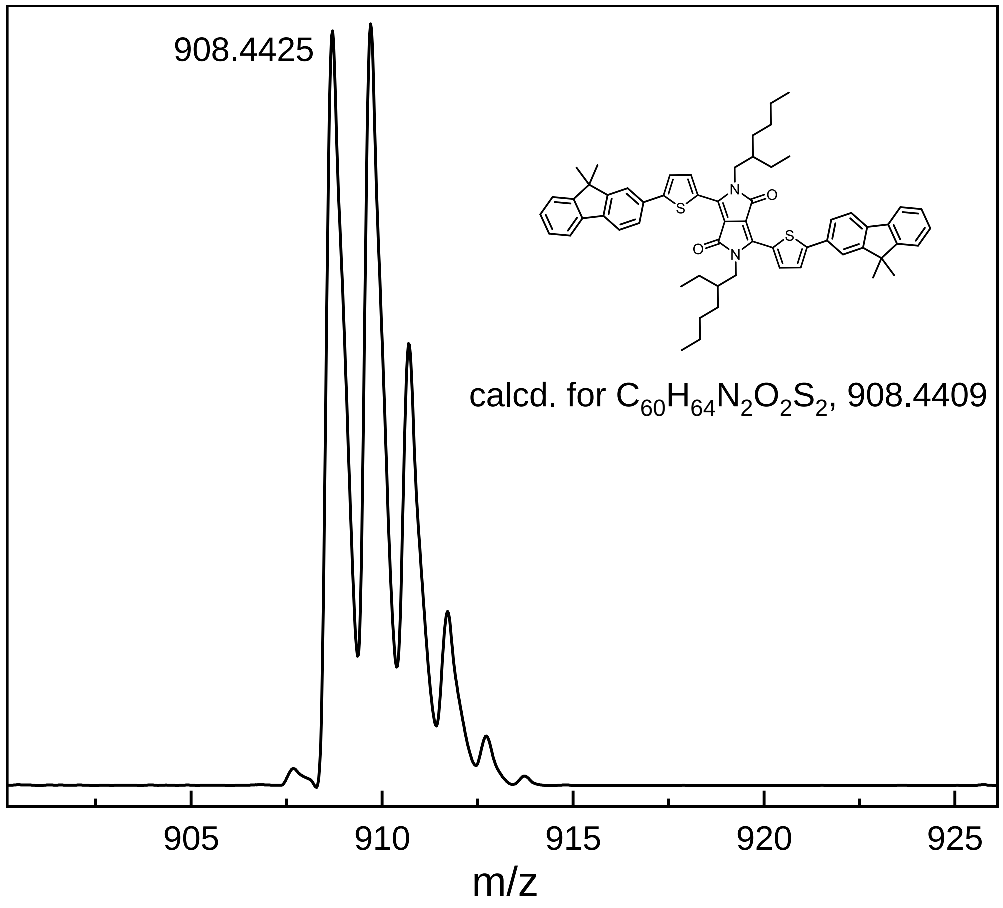

Supplementary Figure 51. MALDI-TOF-MS of Flu-TDPP. Calcd for $C_{60}H_{64}N_2O_2S_2$: *m/z*: 908.4409. Found: 908.4425.

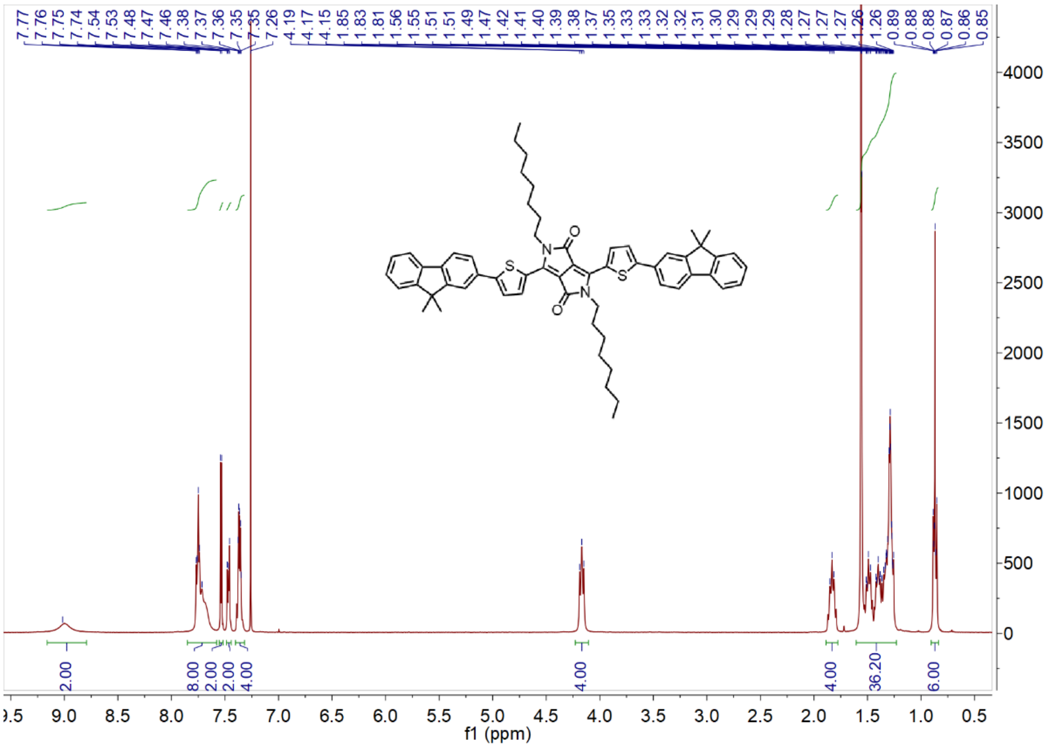

Supplementary Figure 52. ^{1}H NMR of Flu-TDPP-C8 in Chloroform-*d*.

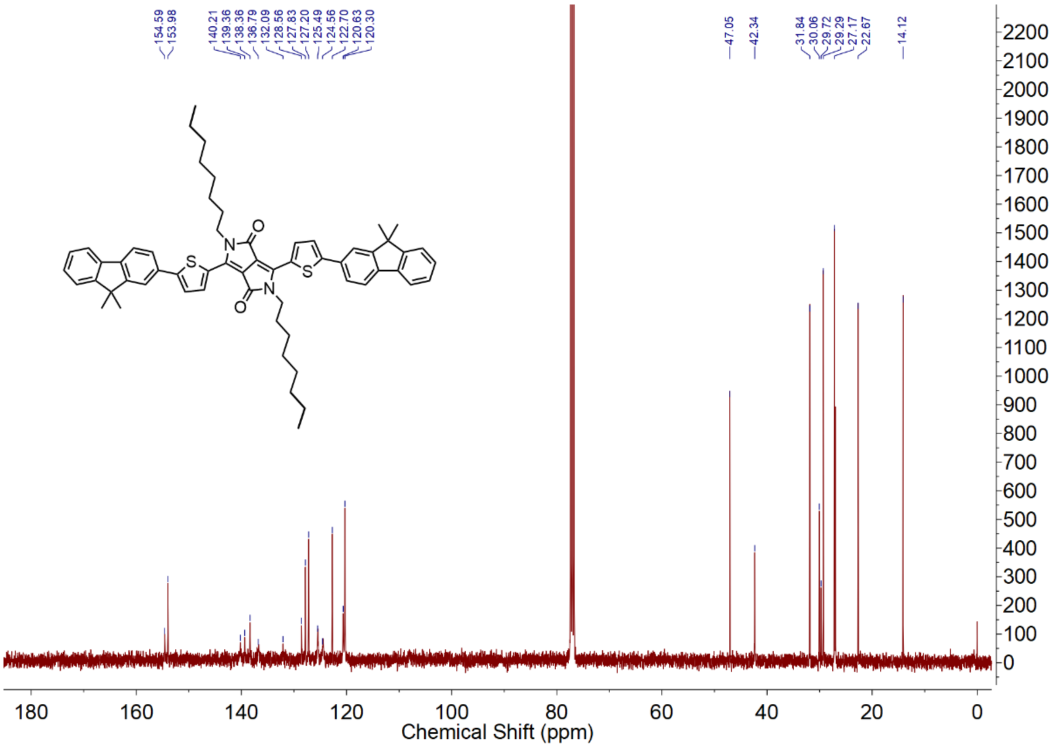

Supplementary Figure 53. ^{13}C NMR of Flu-TDPP-C8 in Chloroform-*d* (saturated solution).

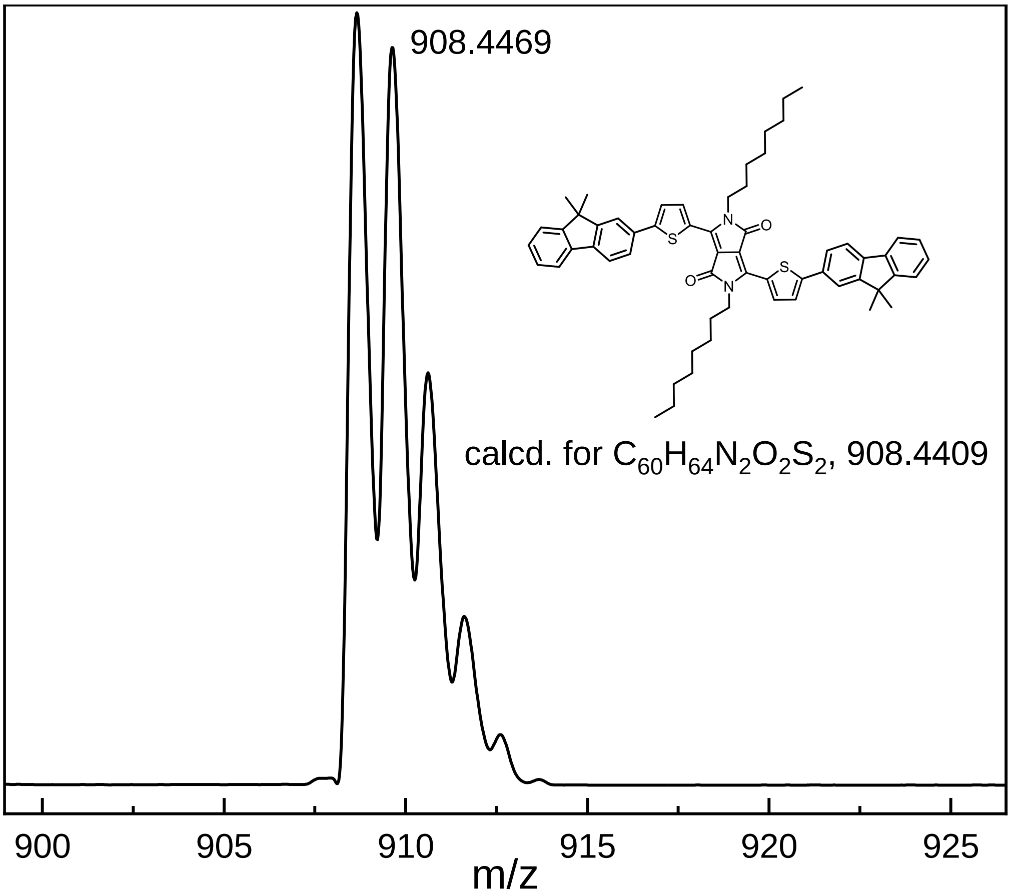

Supplementary Figure 54. MALDI-TOF-MS of Flu-TDPP-C8. Calcd for $C_{60}H_{64}N_2O_2S_2$: *m/z*: 908.4409. Found: 908.4469.

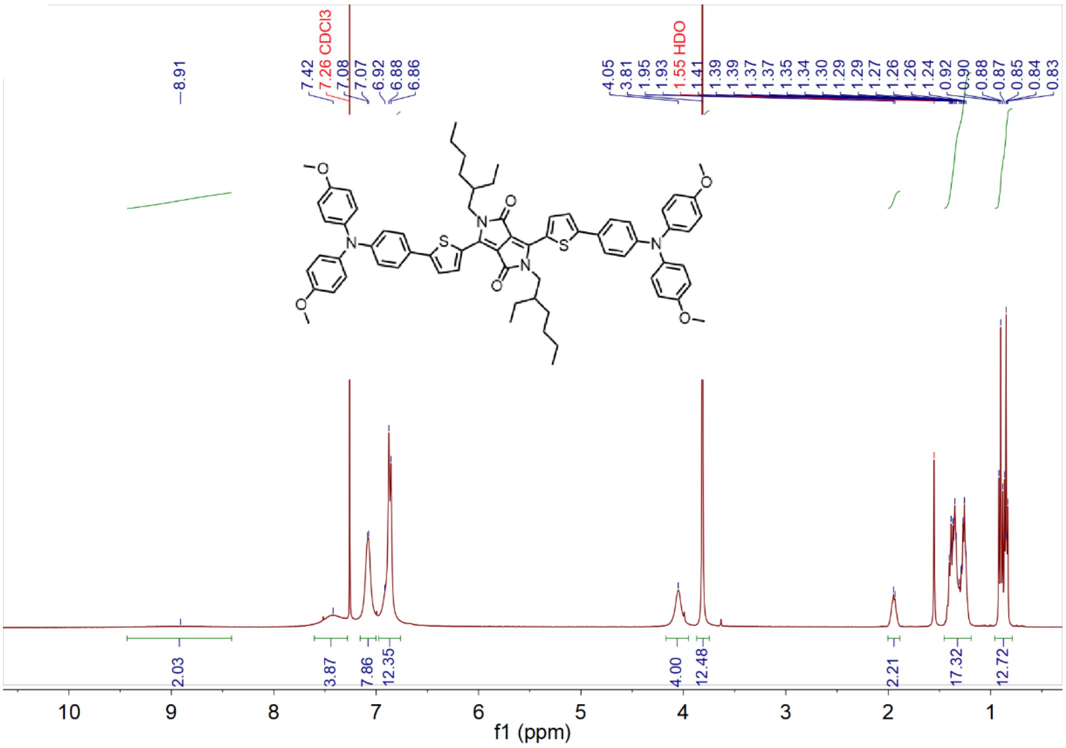

Supplementary Figure 55. ^{1}H NMR of TPAOMe-TDPP in Chloroform-*d*.

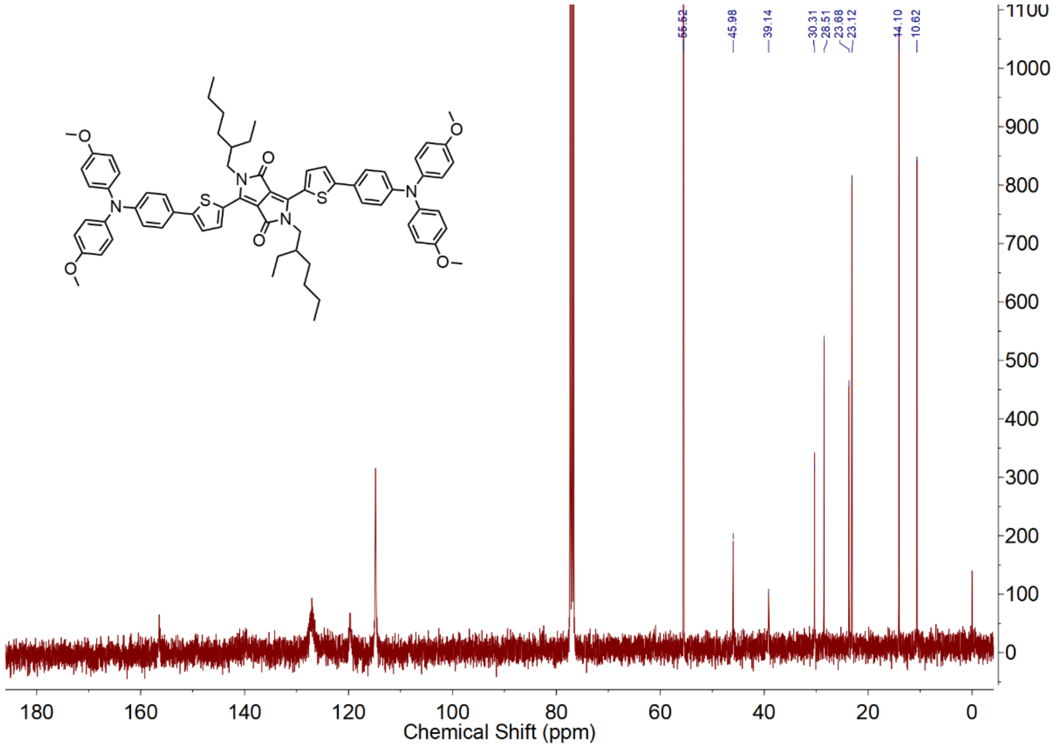

Supplementary Figure 56. ^{13}C NMR of TPAOMe-TDPP in Chloroform-*d* (saturated solution).

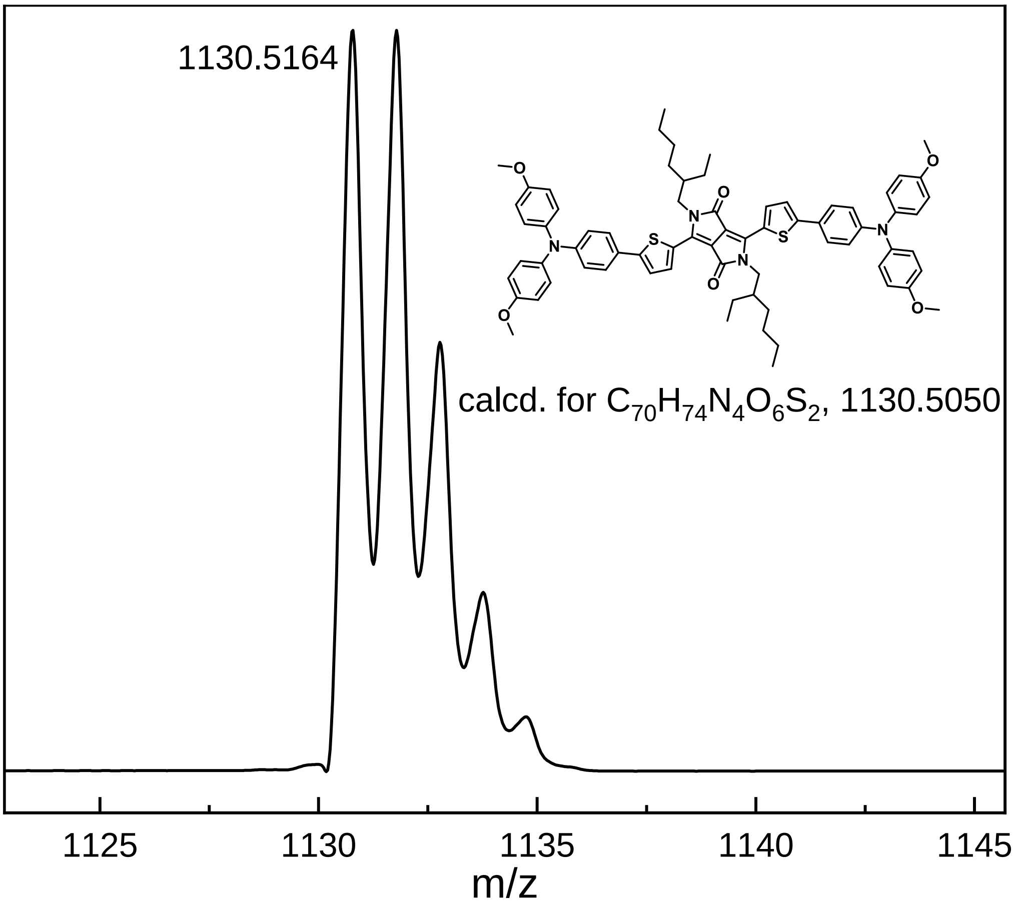

Supplementary Figure 57. MALDI-TOF-MS of TPAOMe-TDPP. Calcd for $C_{70}H_{74}N_4O_6S_2$: *m/z*: 1130.5050. Found:

1130.5164.

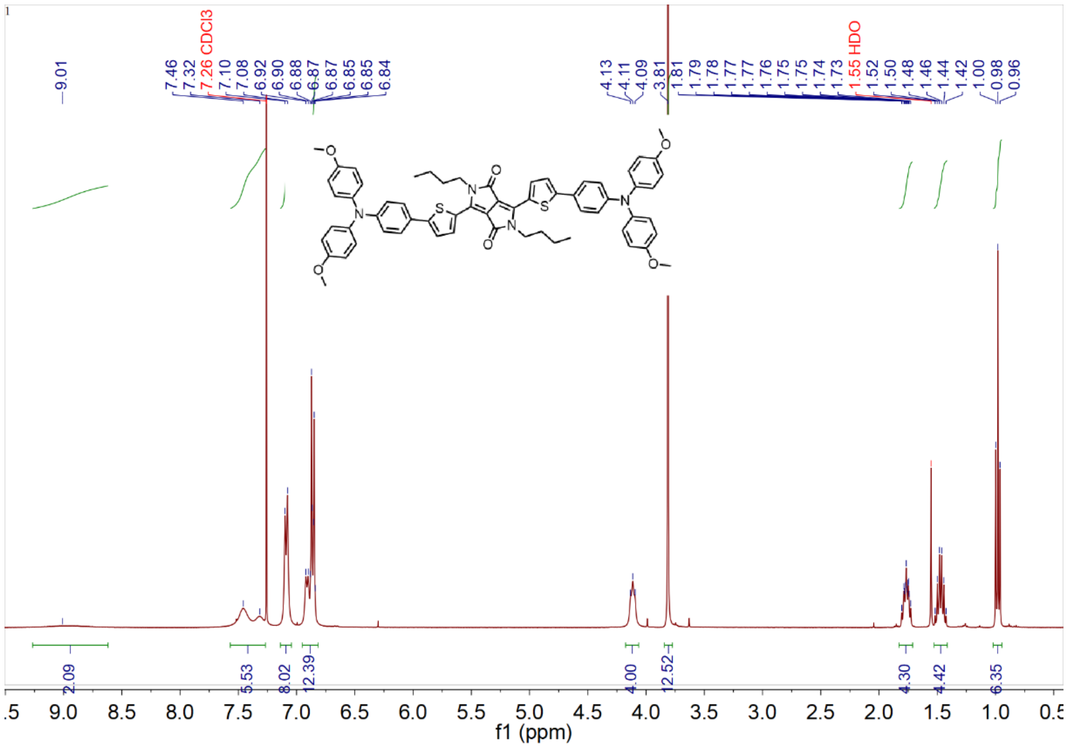

Supplementary Figure 58. ^{1}H NMR of TPAOMe-TDPP-C4 in Chloroform-*d*.

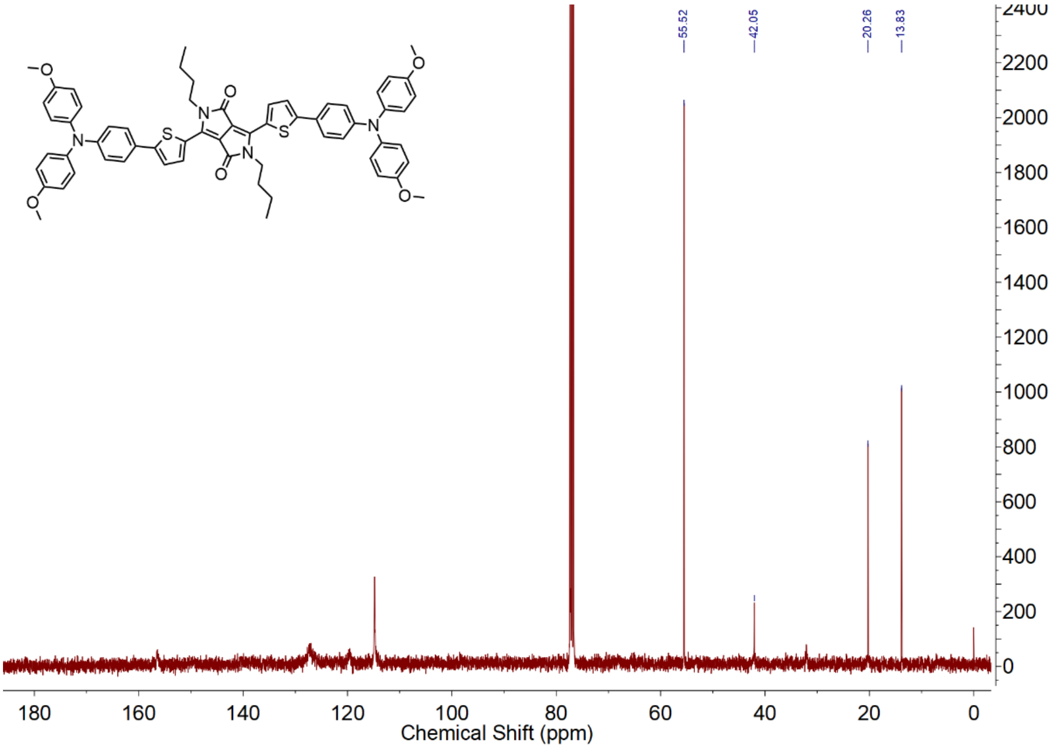

Supplementary Figure 59. ^{13}C NMR of TPAOMe-TDPP-C4 in Chloroform-*d* (saturated solution).

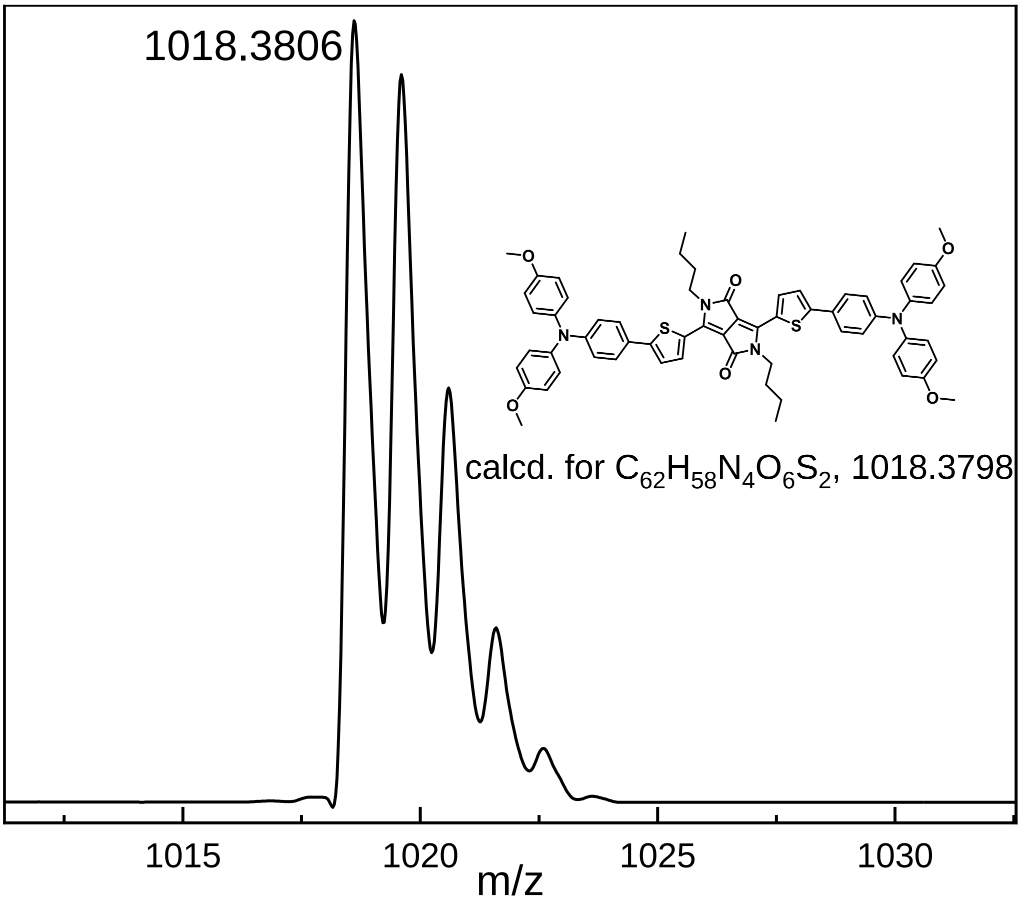

Supplementary Figure 60. MALDI-TOF-MS of TPAOMe-TDPP-C4. Calcd for $C_{62}H_{58}N_4O_6S_2$: *m/z*: 1018.3798. Found:1018.3806.

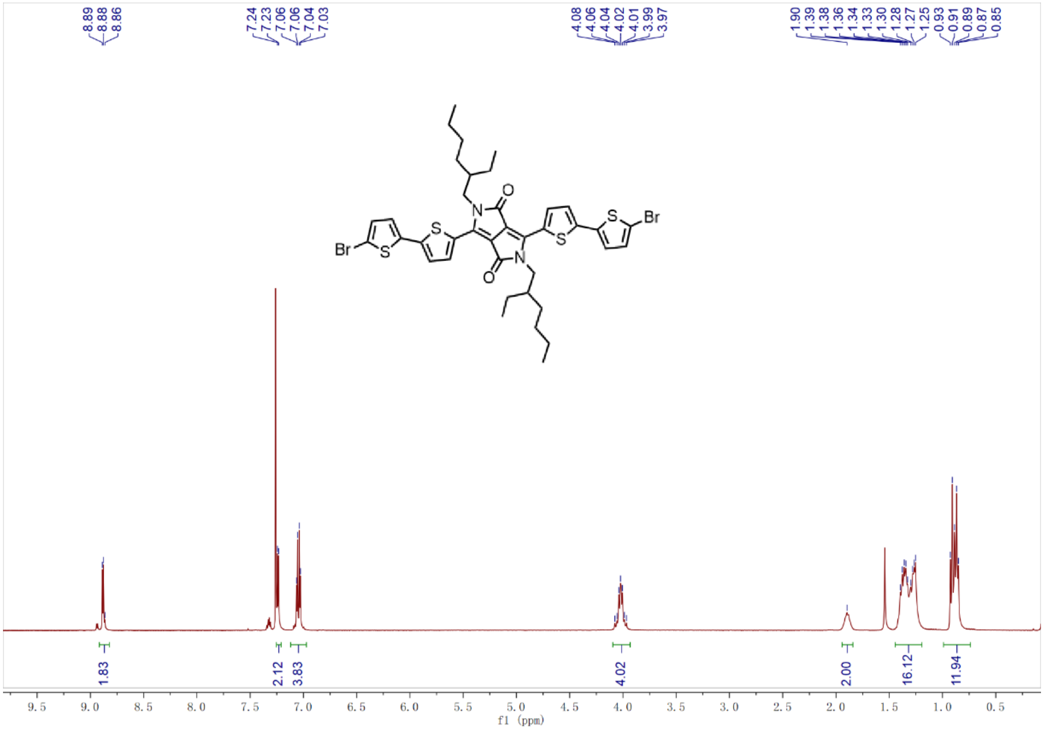

Supplementary Figure 61. The ^{1}H NMR spectrum of Br_2-TTDPP in Chloroform-*d*.

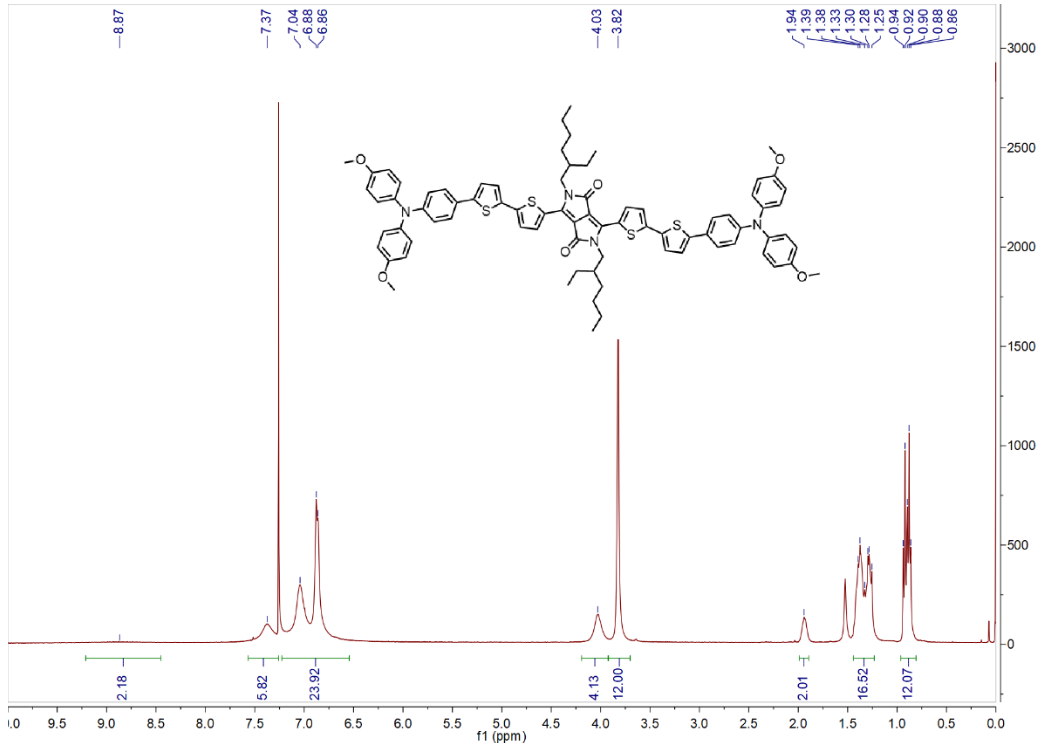

Supplementary Figure 62. ^{1}H NMR of TPAOMe-TTDPP in Chloroform-*d*.

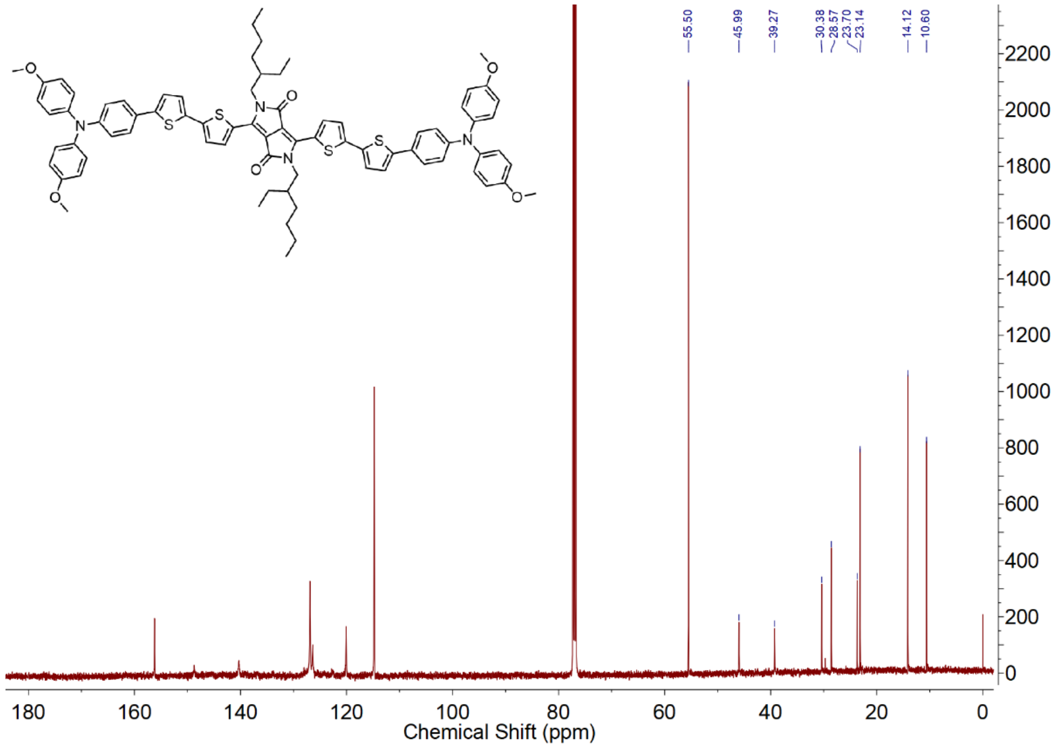

Supplementary Figure 63. ^{1}H NMR of TPAOMe-TTDPP in Chloroform-*d*.

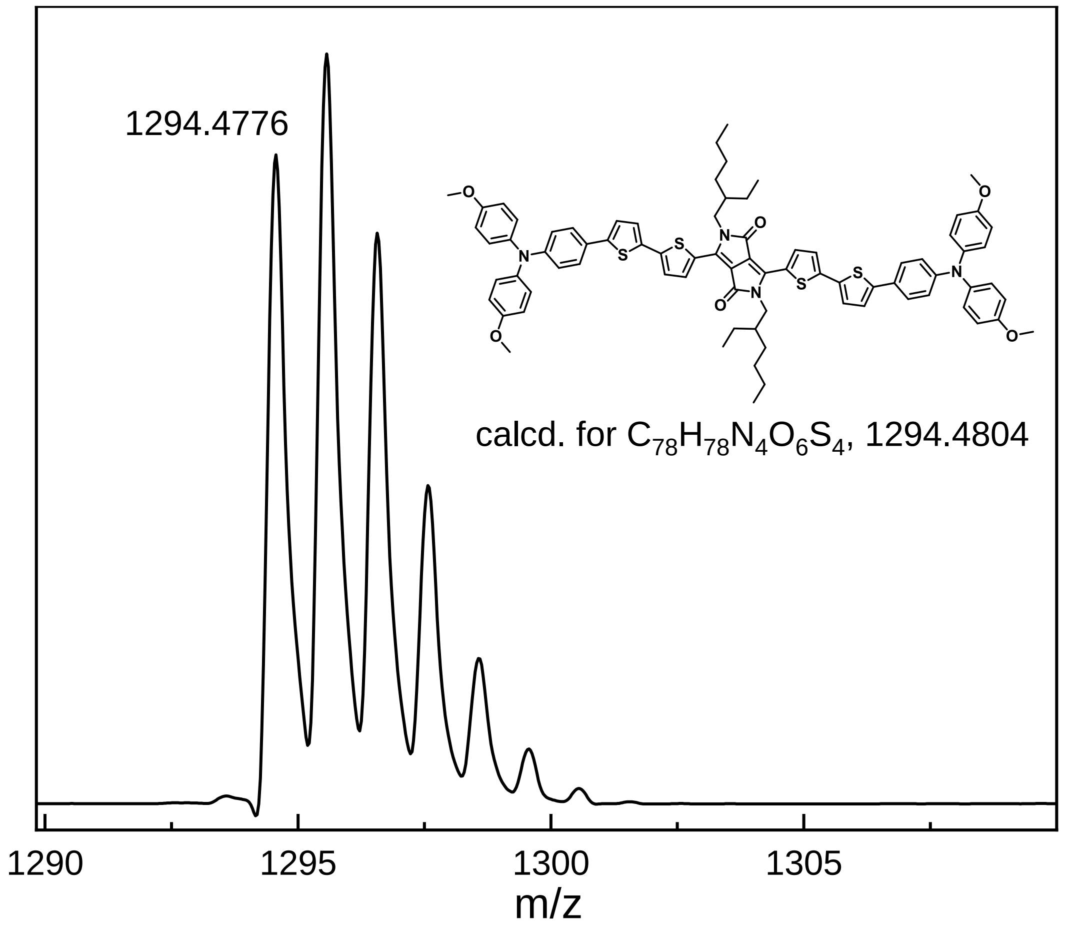

Supplementary Figure 64. MALDI-TOF-MS of TPAOMe-TTDPP. Calcd for $C_{78}H_{78}N_4O_6S_4$: *m/z*: 1294.4804. Found: 1294.4776.

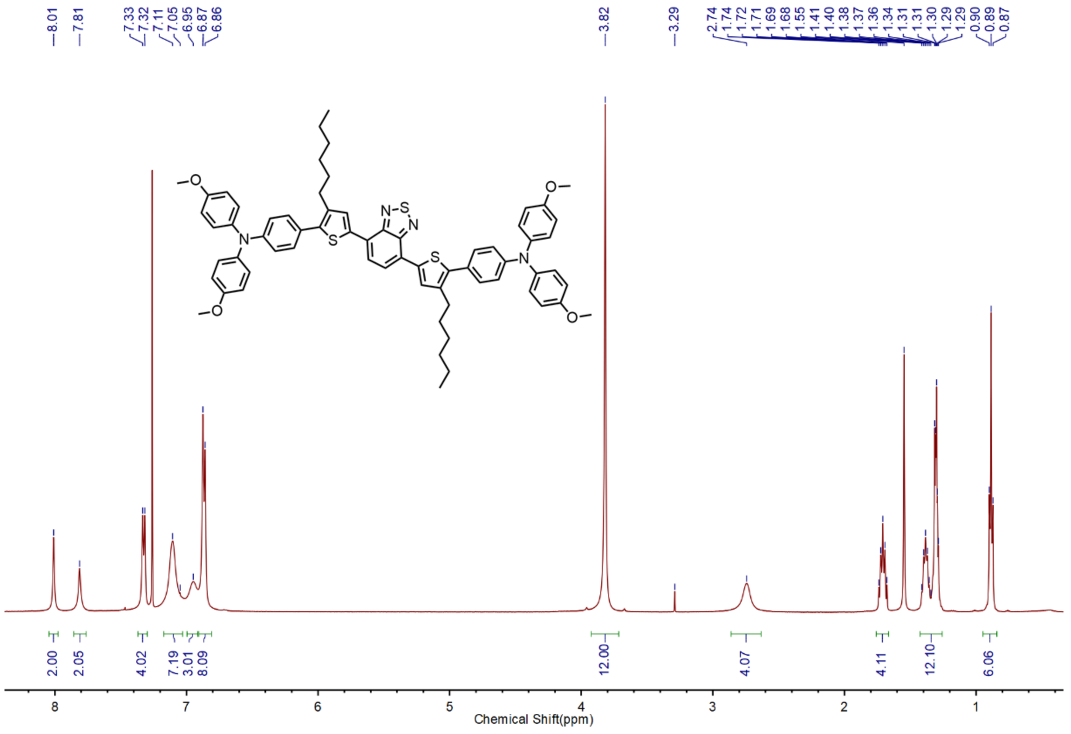

Supplementary Figure 65. ^{1}H NMR of TPAOMe-BTT in Chloroform-*d*.

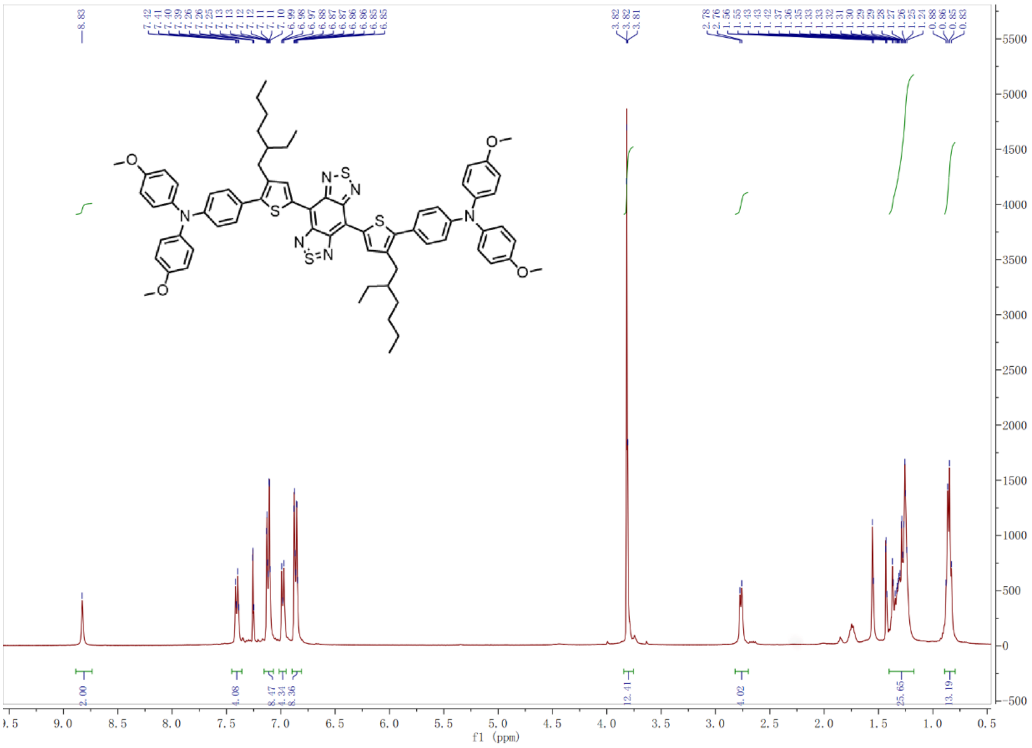

Supplementary Figure 66. ^{1}H NMR of TPAOMe-BBTT in Chloroform-*d*.

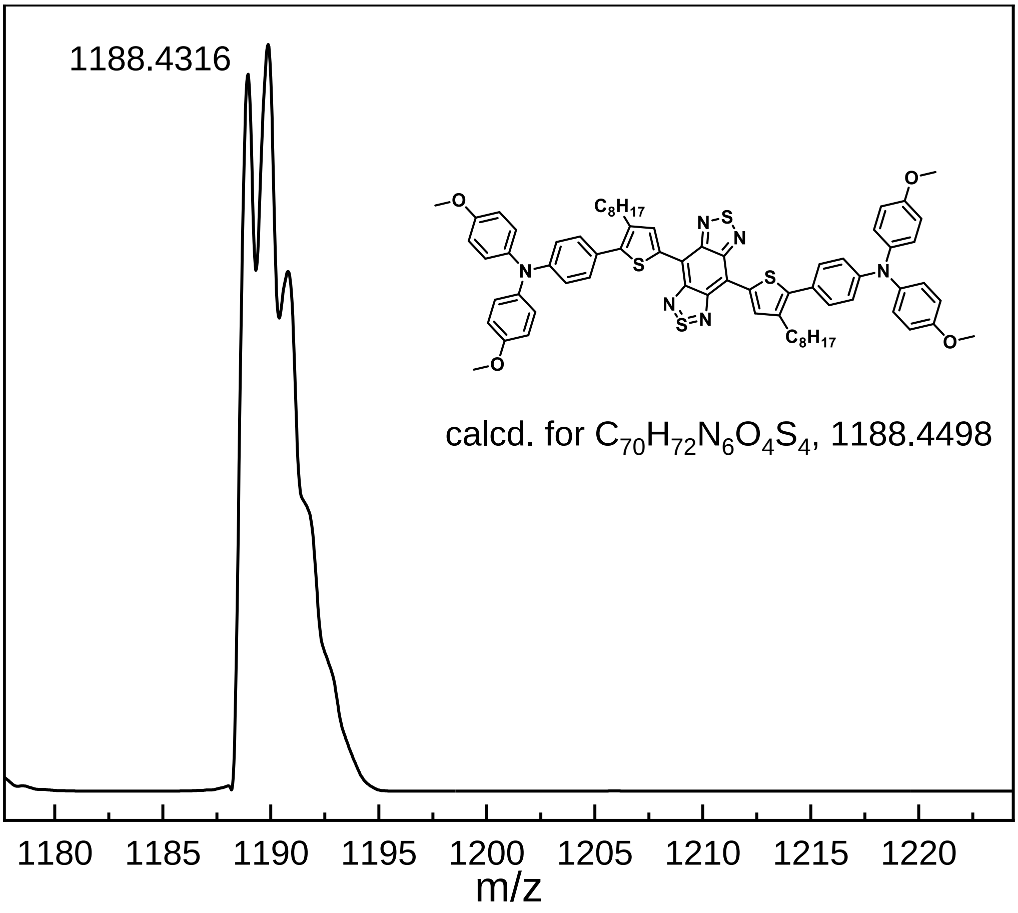

Supplementary Figure 67. MALDI-TOF-MS of TPAOMe-BBTT. Calcd for $C_{78}H_{78}N_4O_6S_4$: *m/z*: 1188.4498. Found: 1188.4316.

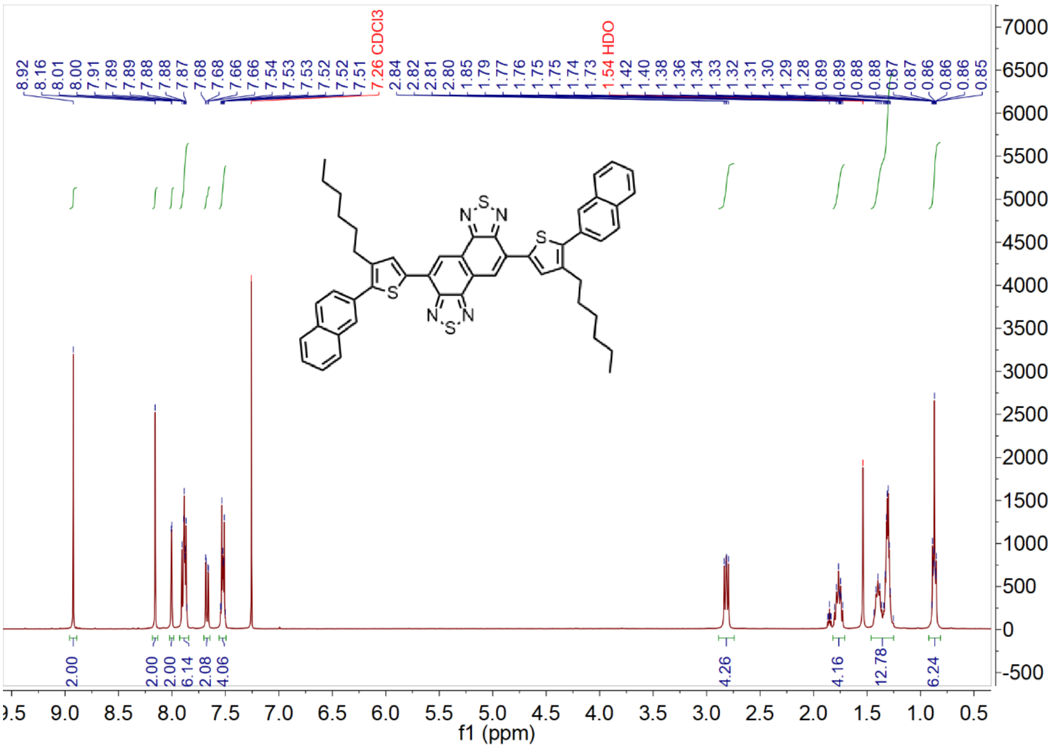

Supplementary Figure 68. ^{1}H NMR of 2N-NTT in Chloroform-*d*.

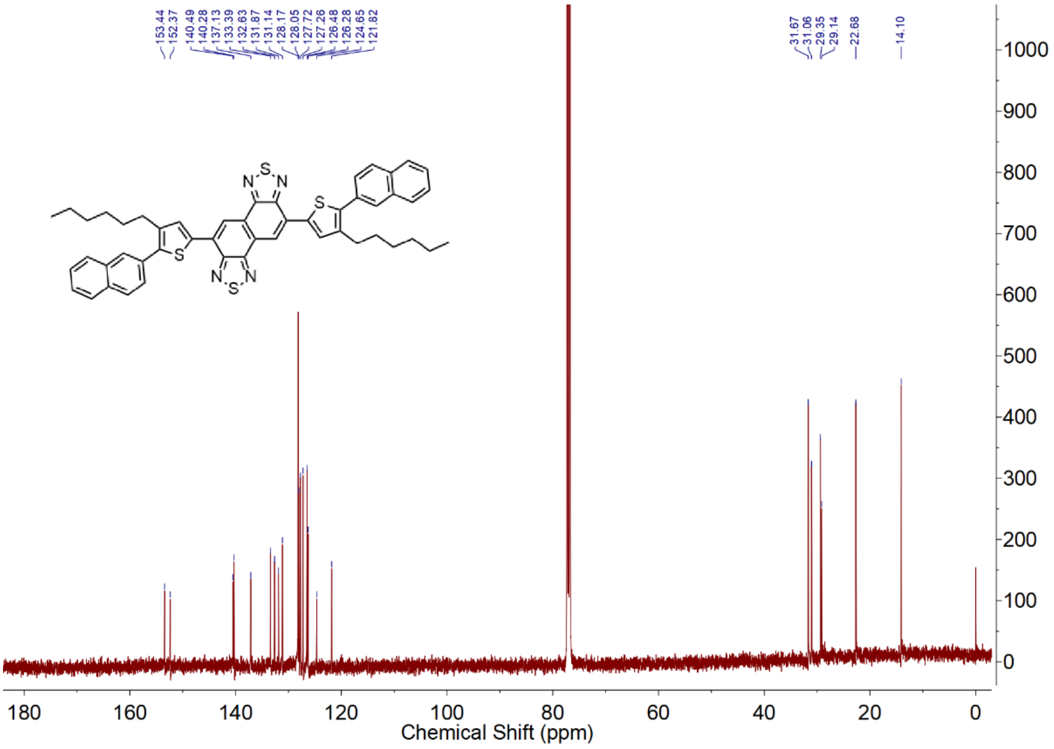

Supplementary Figure 69. ^{13}C NMR of 2N-NTT in Chloroform-*d*.

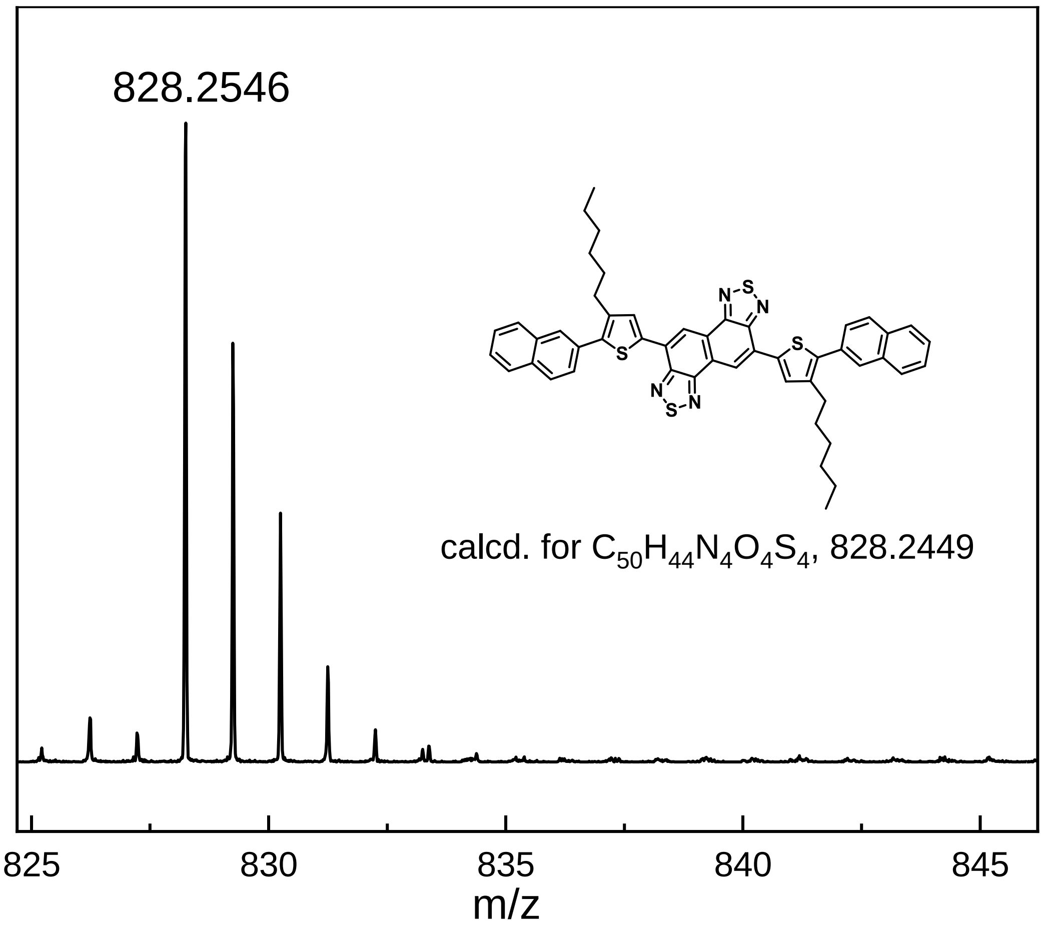

Supplementary Figure 70. MALDI-TOF-MS of 2N-NTT. Calcd for $C_{50}H_{44}N_4S_4$: *m/z*: 828.2449. Found: 828.2546.

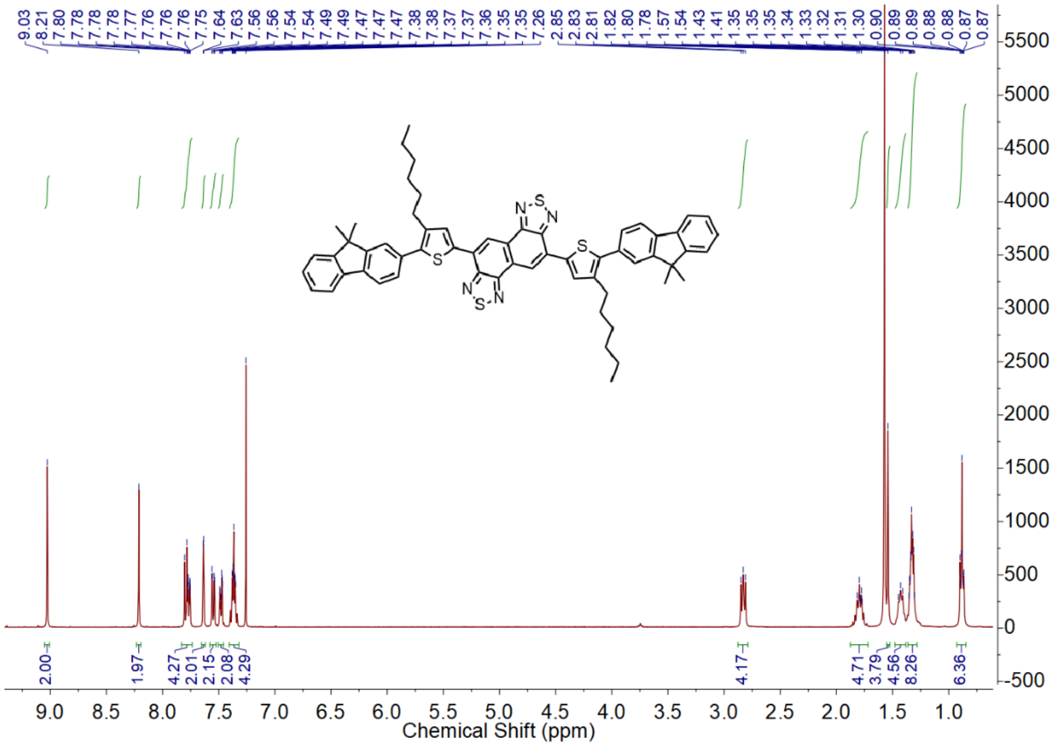

Supplementary Figure 71. ^{1}H NMR of Flu-NTT in Chloroform-*d*.

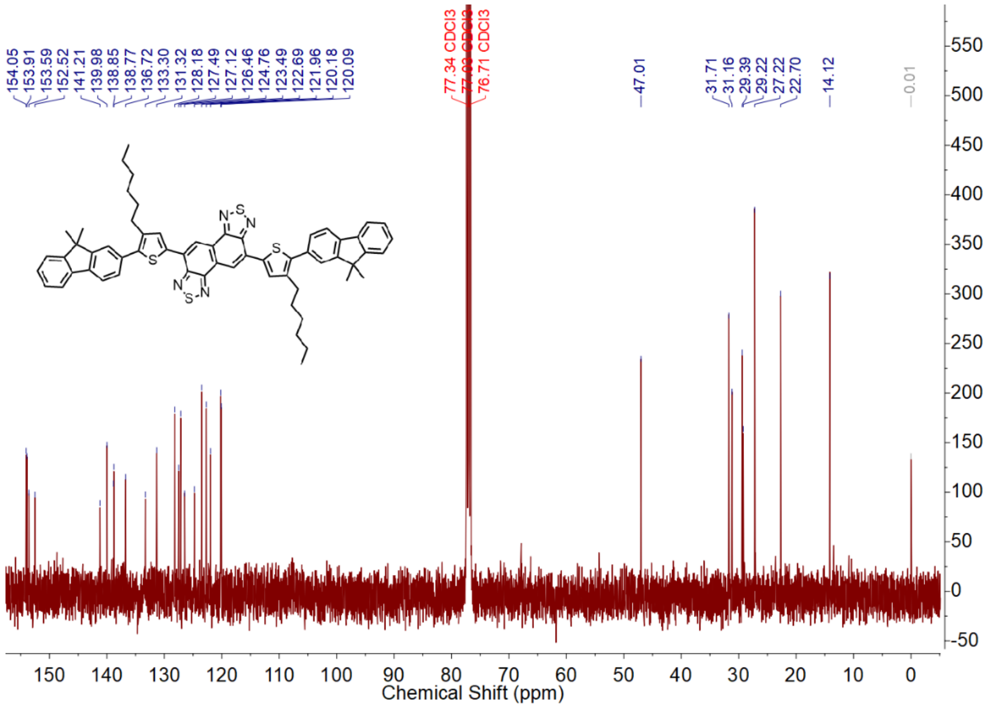

Supplementary Figure 72. ^{13}C NMR of Flu-NTT in Chloroform-*d*.

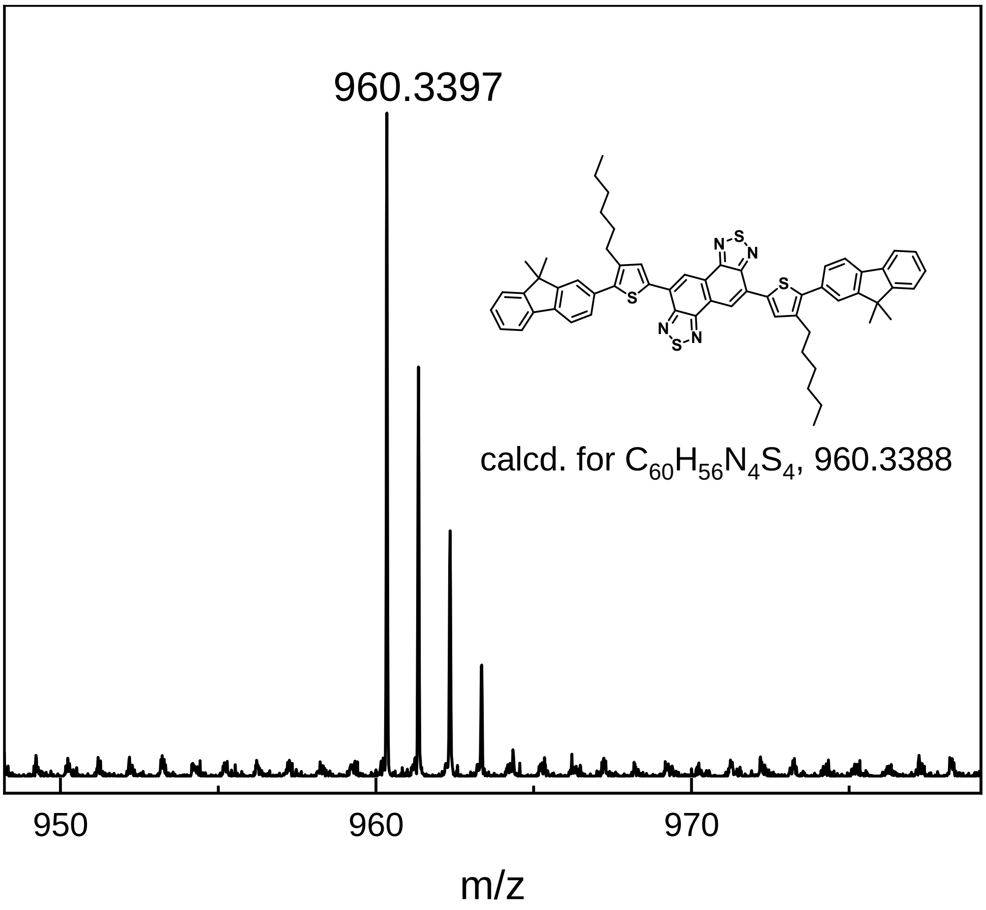

Supplementary Figure 73. MALDI-TOF-MS of Flu-NTT. Calcd for $C_{60}H_{56}N_4S_4$: *m/z*: 960.3397. Found: 960.3388.

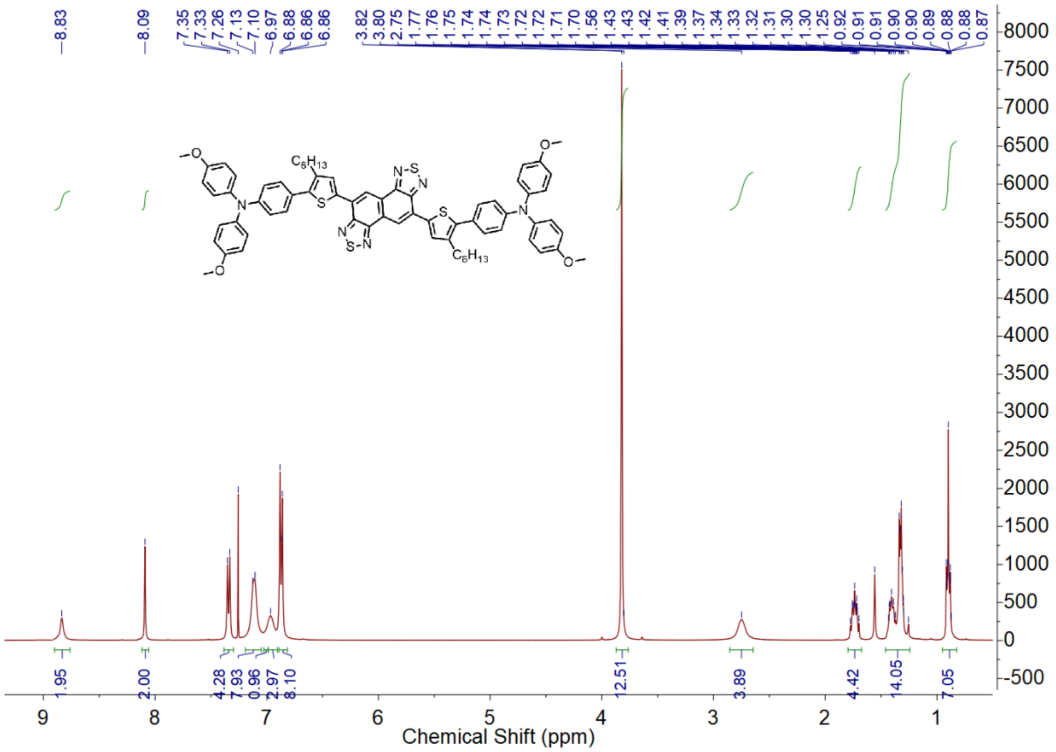

Supplementary Figure 74. The ^{1}H NMR spectrum of TPAOMe-NTT in Chloroform-*d*.

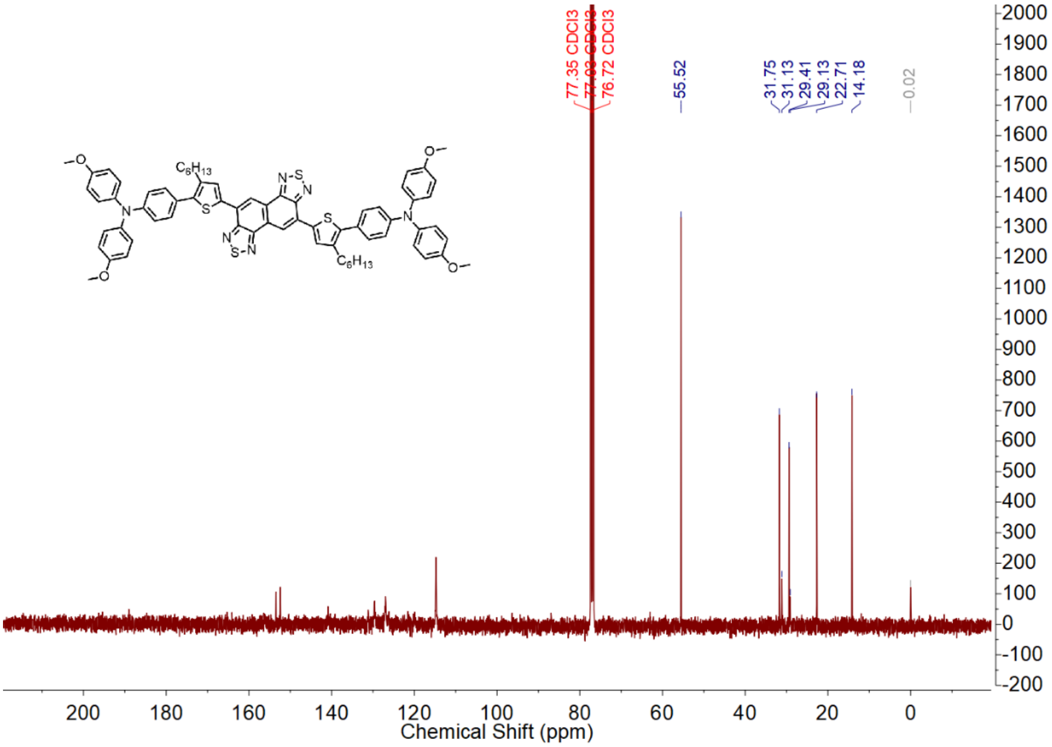

Supplementary Figure 75. The ^{13}C NMR spectrum of TPAOMe-NTT in Chloroform-*d*.

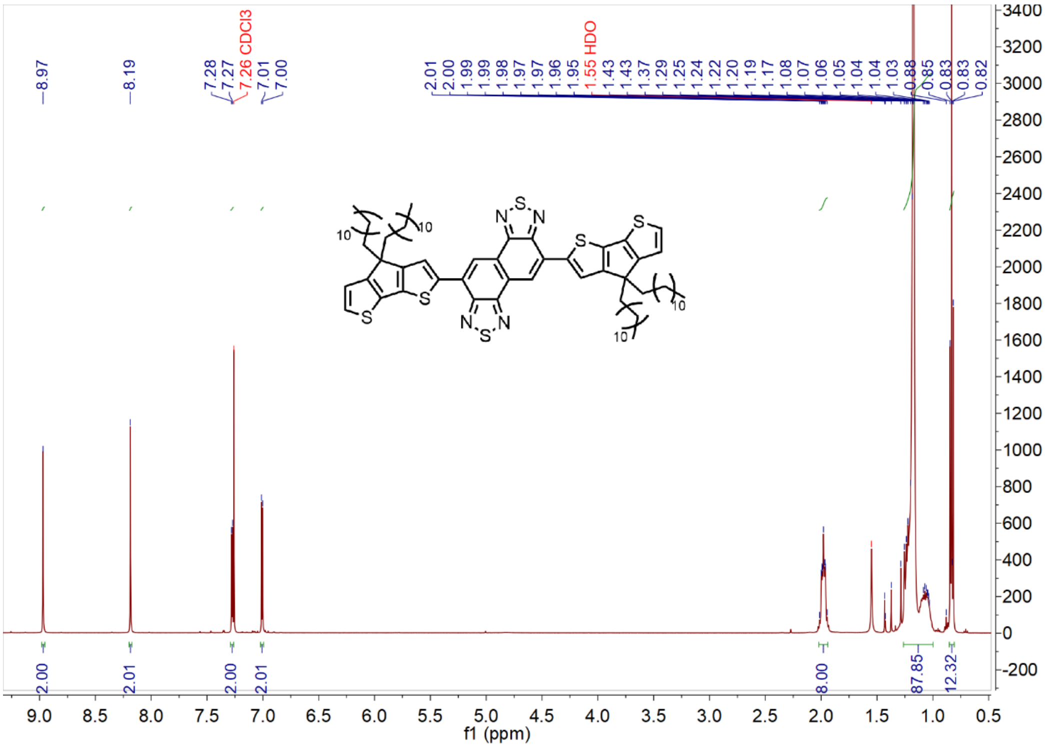

Supplementary Figure 76. ^{1}H NMR of NTC in Chloroform-*d*.

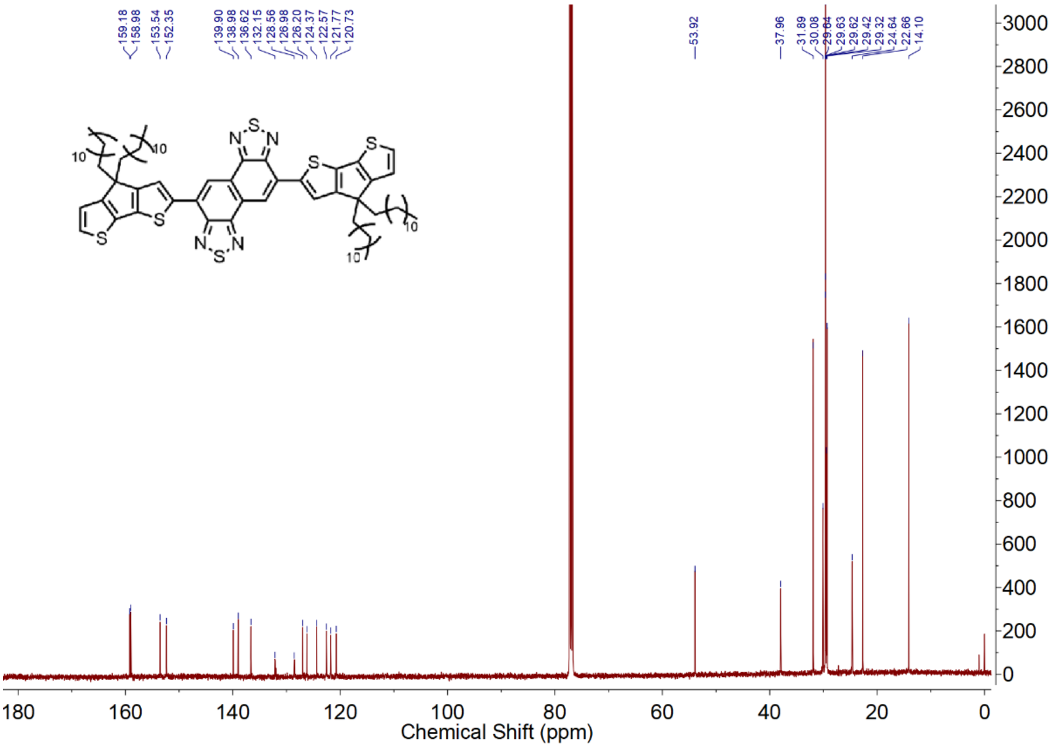

Supplementary Figure 77. ^{13}C NMR of NTC in Chloroform-*d*.

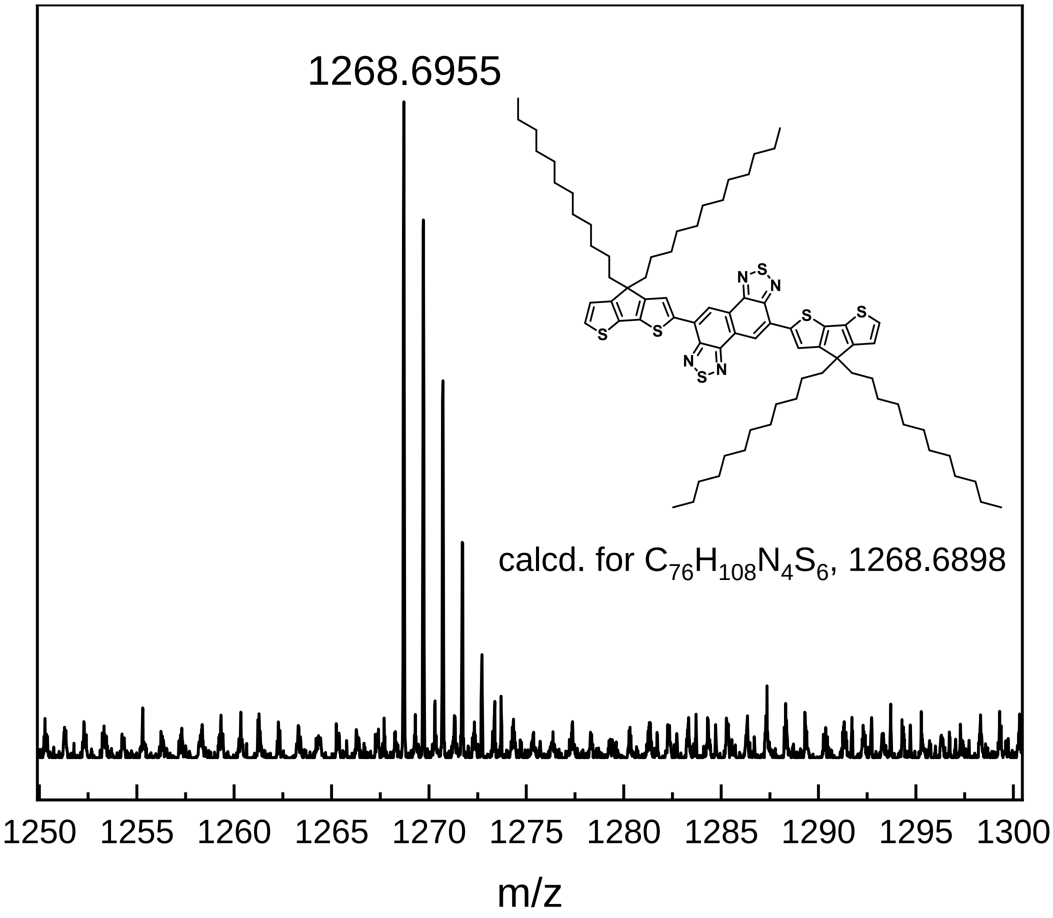

Supplementary Figure 78. MALDI-TOF-MS of NTC. Calcd for $C_{76}H_{108}N_4S_6$: *m/z*: 1268.6898. Found: 1268.6955.

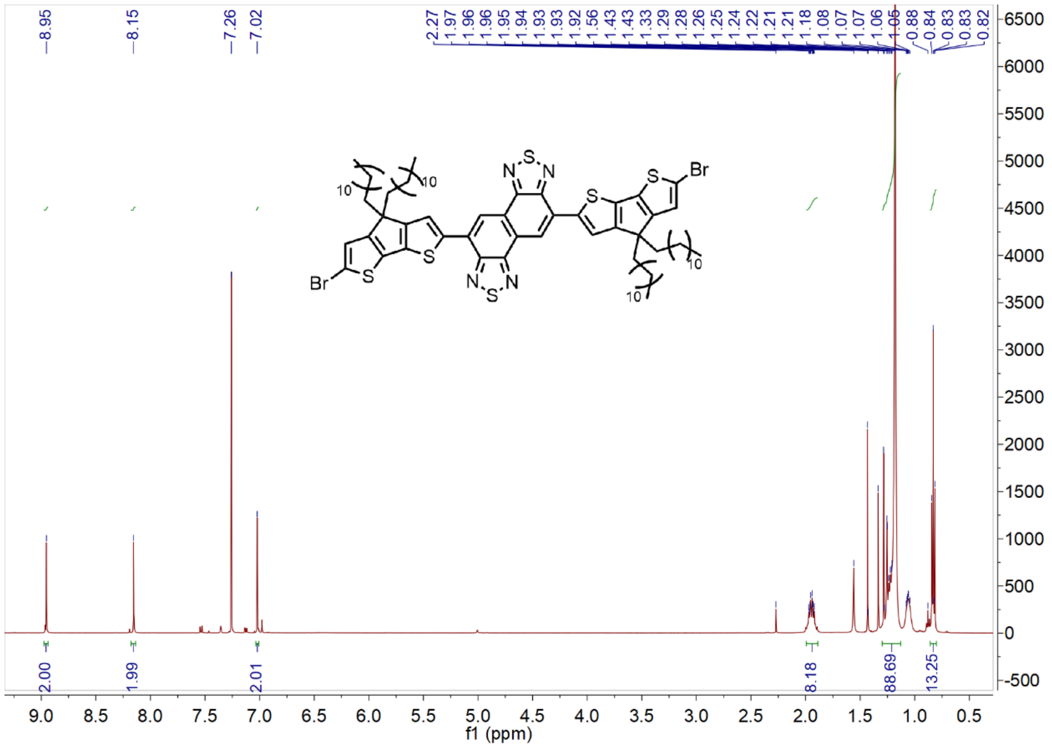

Supplementary Figure 79. ^{1}H NMR of Br_2-NTC in Chloroform-*d*.

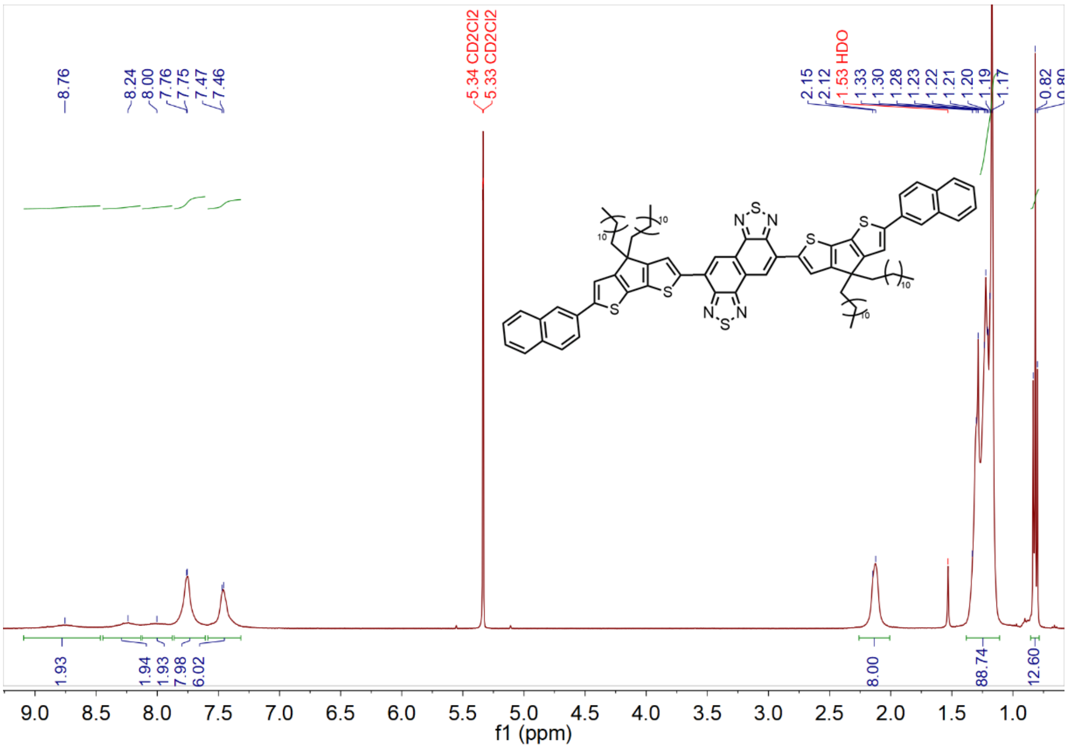

Supplementary Figure 80. ^{1}H NMR of 2N-NTC in dichloromethane-*d2*.

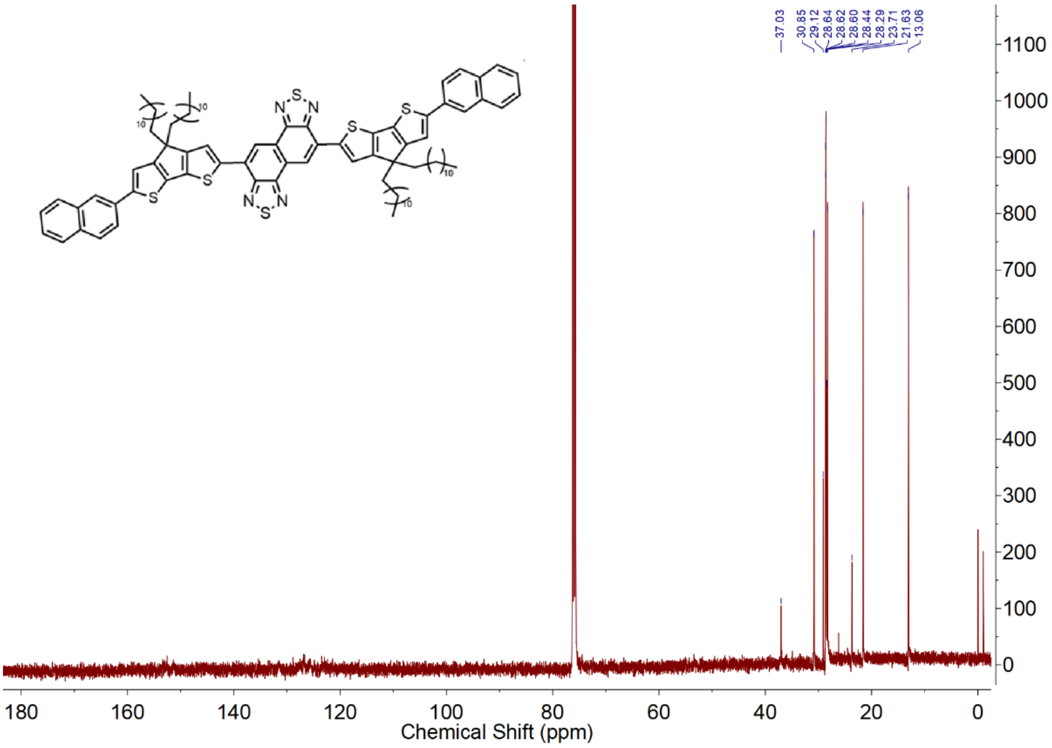

Supplementary Figure 81. ^{13}C NMR of 2N-NTC in Chloroform-*d*.

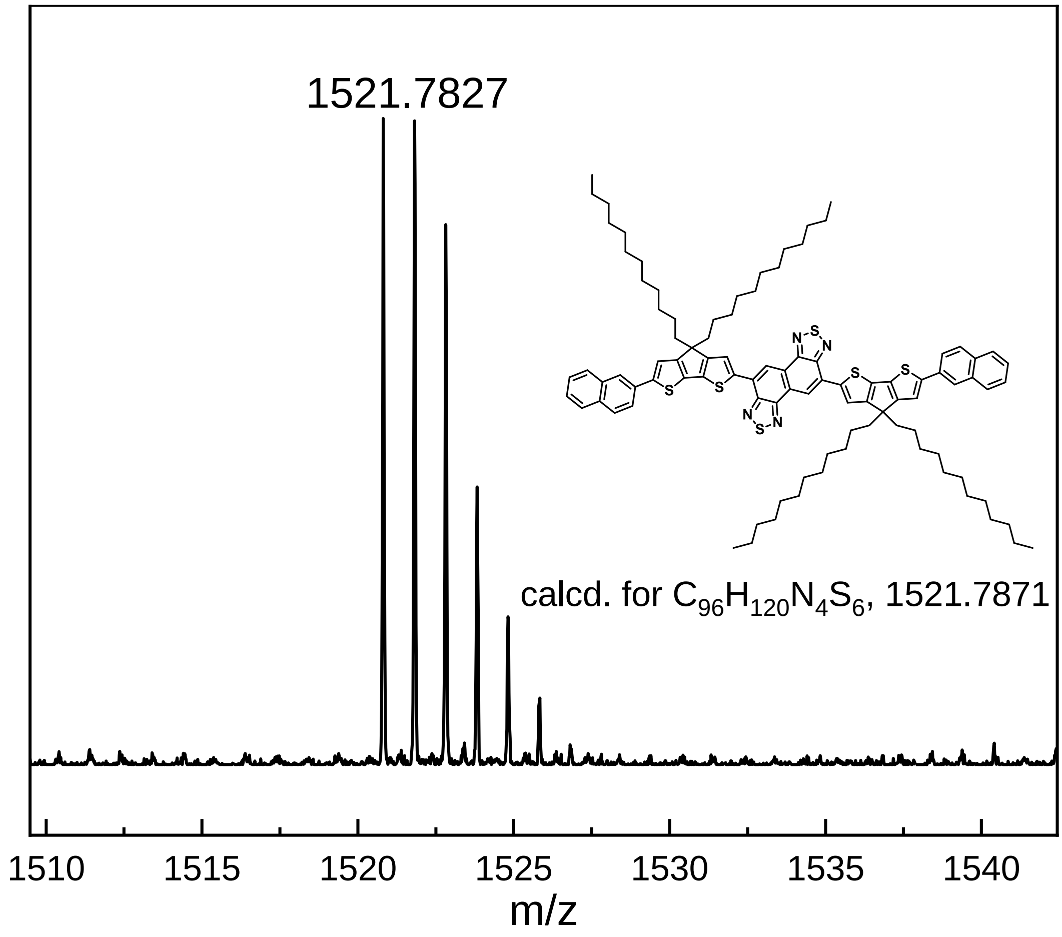

Supplementary Figure 82. MALDI-TOF-MS of 2N-NTC. Calcd for $C_{96}H_{120}N_4S_6$: *m/z*: 1521.7871. Found: 1521.7827.

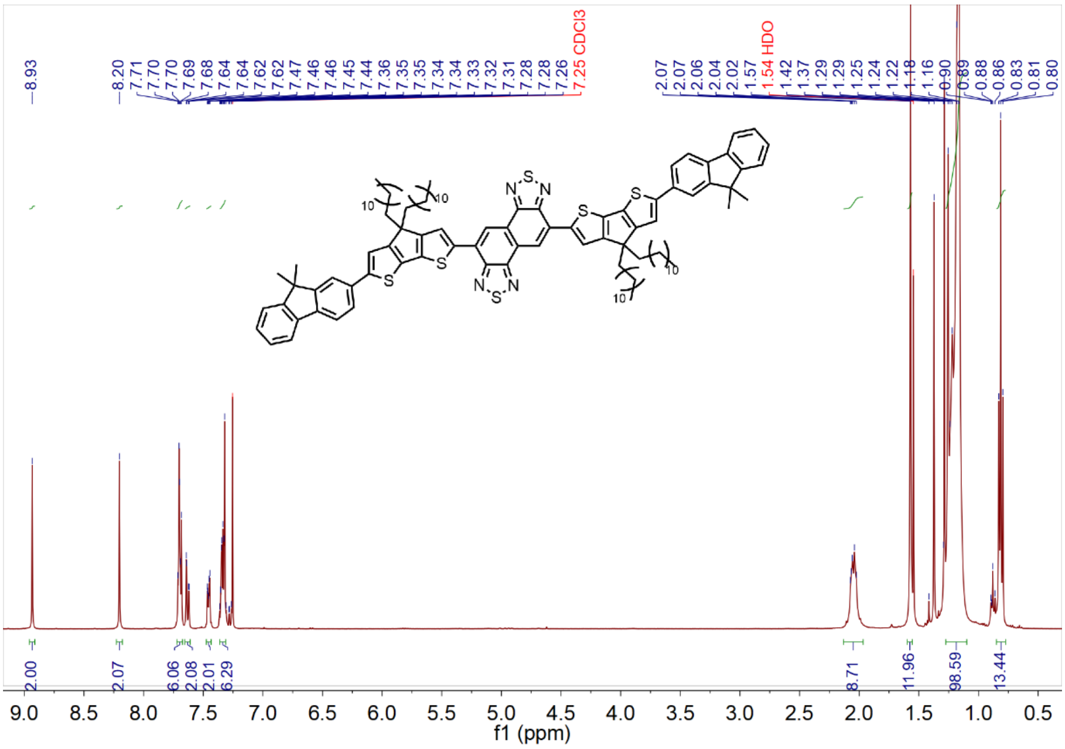

Supplementary Figure 83. ^{1}H NMR of Flu-NTC in Chloroform-*d*.

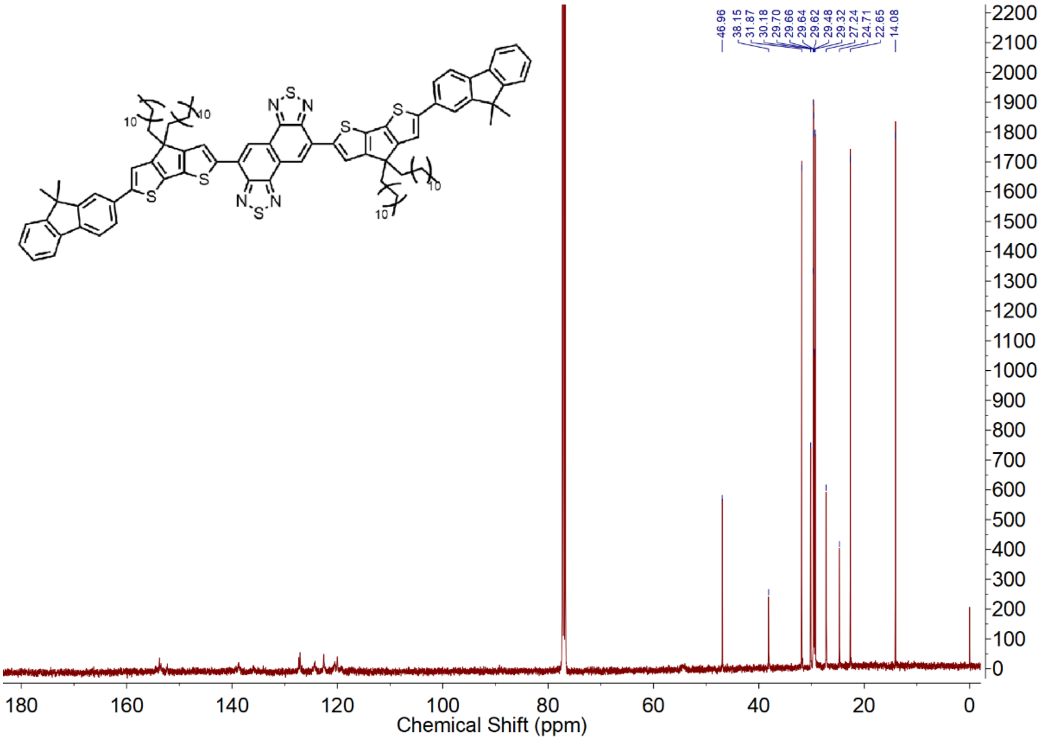

Supplementary Figure 84. ^{13}C NMR of Flu-NTC in Chloroform-*d* (saturated solution).

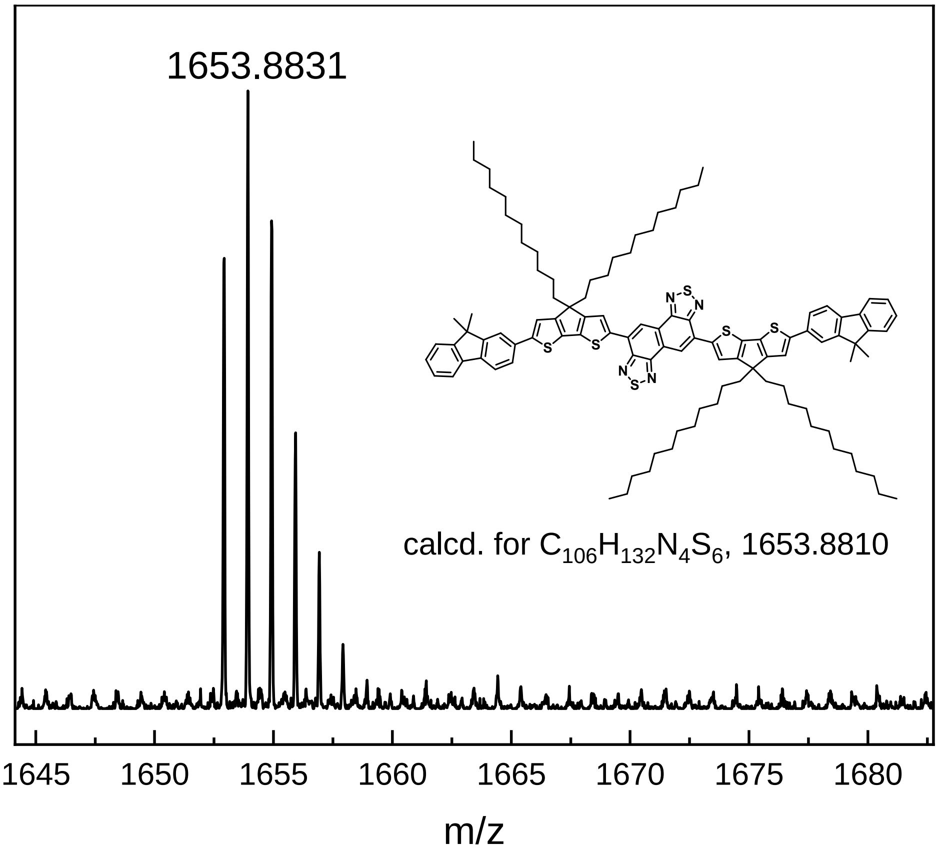

Supplementary Figure 85. MALDI-TOF-MS of Flu-NTC. Calcd for $C_{106}H_{132}N_4S_6$: *m/z*: 1653.8810. Found: 1653.8831.

2.12. Optimized coordinates

Optimized coordinates for Ph-TDPP singlet state at RB3LYP/6-31G(d,p) level of theory and basis set.

C	-0.14107600	-1.79427900	0.00022200
C	-0.57520400	-0.41657700	0.00102700
C	0.57520400	0.41657700	0.00102800
C	1.72304100	-0.37957900	0.00093000
C	-1.72304100	0.37958000	0.00092800
C	0.14107600	1.79427900	0.00022100
N	-1.29108100	1.70483500	0.00076600
N	1.29108000	-1.70483400	0.00076800
C	-2.08242100	2.92198700	0.00050300
H	-2.71040700	2.99451600	0.89334100
H	-1.36722600	3.74611500	0.00018500
C	2.08242100	-2.92198700	0.00051300
H	1.36722600	-3.74611500	0.00019800
H	2.71051400	-2.99382200	-0.89230500
O	0.73642900	2.87021100	-0.00127100
O	-0.73643000	-2.87021000	-0.00127000
C	-3.08752600	-0.07072900	0.00161900
C	-3.48153000	-1.40387700	-0.00075900
C	-4.87924300	-1.57733800	-0.00182700
H	-2.75904000	-2.21399800	-0.01087900
C	-5.59716400	-0.39601200	-0.00196100
H	-5.35422300	-2.55072300	-0.03298100
C	3.08752600	0.07072900	0.00162200
C	3.48153000	1.40387700	-0.00075300
C	4.87924300	1.57733800	-0.00182200
H	2.75904000	2.21399900	-0.01087200
C	5.59716500	0.39601200	-0.00195900
H	5.35422400	2.55072300	-0.03297500
S	4.50875600	-0.97124200	0.01156600
S	-4.50875600	0.97124200	0.01156600
C	-7.05114900	-0.21344000	-0.00357300
C	-7.64897700	0.95476700	-0.51183400
C	-7.88970500	-1.22246400	0.50812000
C	-9.03297800	1.10736400	-0.50861700
H	-7.02527400	1.73826700	-0.93208500
C	-9.27310400	-1.07011300	0.50081300
H	-7.44984100	-2.11884400	0.93313500
C	-9.85248000	0.09539300	-0.00568100
H	-9.47256200	2.01601100	-0.90915100
H	-9.90077400	-1.85996100	0.90281100
H	-10.93166400	0.21423100	-0.00628000
C	7.05114900	0.21344000	-0.00357300
C	7.64897600	-0.95476400	-0.51184200
C	7.88970600	1.22246000	0.50812600
C	9.03297800	-1.10736200	-0.50862600
H	7.02527300	-1.73826200	-0.93209600
C	9.27310500	1.07011000	0.50081700
H	7.44984200	2.11883700	0.93314600
C	9.85248000	-0.09539400	-0.00568500
H	9.47256200	-2.01600600	-0.90916700
H	9.90077500	1.85995500	0.90281900
H	10.93166400	-0.21423100	-0.00628600
H	2.71040500	-2.99451100	0.89335300
H	-2.71051200	2.99381800	-0.89231700

Optimized coordinates for 1N-TDPP singlet state at RB3LYP/6-31G(d,p) level of theory and basis set.

C	-0.28480600	-1.63249900	-0.70244500
C	-0.60532100	-0.32234300	-0.18560300
C	0.60531800	0.32236800	0.18555800
C	1.68118100	-0.52859200	-0.07567500
C	-1.68118300	0.52861600	0.07563200
C	0.28480300	1.63252500	0.70239700
N	-1.14560700	1.70036800	0.60733300
N	1.14560500	-1.70034300	-0.60738000
C	-1.83466600	2.90012900	1.04704000
H	-2.52186900	2.69089000	1.87214700
H	-1.05808400	3.58310700	1.39511100
C	1.83466400	-2.90010500	-1.04708500
H	1.05808200	-3.58307800	-1.39516800
H	2.52187600	-2.69086300	-1.87218400
O	0.96259500	2.55766100	1.14586300
O	-0.96259700	-2.55763500	-1.14591300
C	-3.07466400	0.25888900	-0.15464600
C	-3.57317000	-0.92096100	-0.69412300
C	-4.97833000	-0.93761300	-0.81245300
H	-2.91975800	-1.73365400	-0.99589200
C	-5.59622400	0.21887600	-0.37639500
H	-5.52929100	-1.76512900	-1.24283100
C	3.07466100	-0.25886800	0.15461200
C	3.57316800	0.92098700	0.69408000
C	4.97832800	0.93763100	0.81242600
H	2.91975800	1.73368800	0.99582900
C	5.59621900	-0.21886800	0.37639400
H	5.52928800	1.76515000	1.24279900
S	4.40469700	-1.35478900	-0.20994300
S	-4.40470200	1.35479700	0.20994000
H	2.38445200	-3.37206900	-0.22746600
H	-2.38446400	3.37208700	0.22742400
C	-7.02053600	0.58985000	-0.39825100
C	-8.04547100	-0.31088500	0.06574600
C	-7.38733500	1.83584800	-0.89007200
C	-7.76835300	-1.56823000	0.66873800
C	-9.41892500	0.09211200	-0.05155800
C	-8.73843400	2.22947600	-0.97944000
H	-6.61515400	2.50887100	-1.24941900
C	-8.78462500	-2.39695000	1.09078000
H	-6.73776700	-1.86806700	0.81459000
C	-10.44207600	-0.79209700	0.38503100
C	-9.73536100	1.36995800	-0.58280400
H	-8.98301700	3.20769200	-1.38187200
C	-10.13683000	-2.01385600	0.93738900
H	-8.54438000	-3.34973100	1.55318000
H	-11.47699100	-0.47728400	0.28027400
H	-10.77995800	1.65778000	-0.66470600
H	-10.92893200	-2.67861400	1.26873900
C	7.02052800	-0.58985300	0.39827900
C	8.04547700	0.31086400	-0.06572200
C	7.38731000	-1.83584300	0.89013300
C	7.76837700	1.56819700	-0.66874800
C	9.41892600	-0.09213900	0.05161400
C	8.73840500	-2.22947700	0.97953300
H	6.61511800	-2.50885200	1.24948300
C	8.78466100	2.39690100	-1.09079200
H	6.73779400	1.86803800	-0.81462300
C	10.44209000	0.79205400	-0.38497900
C	9.73534400	-1.36997600	0.58289500
H	8.98297400	-3.20768700	1.38199100
C	10.13686100	2.01380200	-0.93737000
H	8.54443000	3.34967200	-1.55321900
H	11.47700100	0.47723600	-0.28019700
H	10.77993900	-1.65780200	0.66482200
H	10.92897300	2.67854700	-1.26872200

Optimized coordinates for 2N-TDPP singlet state at RB3LYP/6-31G(d,p) level of theory and basis set.

C	-0.22437700	1.76857200	0.24712400
C	-0.59369800	0.38362100	0.06815500
C	0.59369800	-0.38361800	-0.06817200
C	1.70305000	0.46143300	0.01695100
C	-1.70305000	-0.46143000	-0.01696500
C	0.22437600	-1.76856900	-0.24714000
N	-1.20975700	-1.75147200	-0.20583400
N	1.20975700	1.75147500	0.20582300
C	-1.94322000	-2.99525200	-0.35631300
H	-2.59132000	-2.97678900	-1.23746700
H	-1.19067900	-3.77503900	-0.48402200
C	1.94322000	2.99525600	0.35629600
H	1.19067800	3.77504200	0.48400800
H	2.59132400	2.97679500	1.23744600
O	0.86906100	-2.80361000	-0.40620000
O	-0.86906100	2.80361300	0.40618400
C	-3.08613000	-0.08361200	0.07082600
C	-3.54179700	1.21601900	0.26394300
C	-4.94463400	1.31817500	0.31861300
H	-2.85750900	2.05192600	0.37090200
C	-5.60775800	0.11365900	0.16905400
H	-5.46349900	2.25286700	0.49515100
C	3.08612900	0.08361400	-0.07084000
C	3.54179700	-1.21601600	-0.26396500
C	4.94463400	-1.31817200	-0.31863000
H	2.85750900	-2.05192100	-0.37093300
C	5.60775900	-0.11365800	-0.16905700
H	5.46349900	-2.25286200	-0.49517300
S	4.45693100	1.18392000	0.05129100
S	-4.45693100	-1.18391900	-0.05128900
H	2.54267500	3.21924000	-0.53100100
H	-2.54268000	-3.21923700	0.53098000
C	-11.61547500	1.45508700	-0.50171300
C	-10.25960000	1.68958200	-0.51094900
C	-9.34110700	0.65978200	-0.16638900
C	-9.85264500	-0.63161600	0.18611100
C	-11.25603000	-0.84045300	0.18617200
C	-12.11934700	0.17873200	-0.14936700
H	-7.56576000	1.84321900	-0.47343200
H	-12.30652500	2.25018000	-0.76537700
H	-9.87146900	2.66807300	-0.78113100
C	-7.94077900	0.86971800	-0.17173500
C	-8.92638900	-1.65560200	0.52206600
H	-11.63939400	-1.82102400	0.45584700
H	-13.19160800	0.00731400	-0.14627500
C	-7.57435000	-1.42117100	0.51656000
C	-7.04962600	-0.13952000	0.16789900
H	-9.30630100	-2.63607900	0.79648900
H	-6.89090900	-2.21457800	0.80345300
C	7.57435300	1.42117700	-0.51653000
C	8.92639200	1.65560800	-0.52202600
C	9.85264700	0.63161500	-0.18608500
C	9.34110600	-0.65978800	0.16639200
C	7.94077800	-0.86972400	0.17172800
C	7.04962700	0.13952100	-0.16789300
H	11.63939700	1.82102700	-0.45579300
H	6.89091400	2.21459000	-0.80341100
H	9.30630700	2.63608900	-0.79643100
C	11.25603200	0.84045200	-0.18613600
C	10.25959700	-1.68959400	0.51093900
H	7.56575700	-1.84322900	0.47340900
C	11.61547200	-1.45510000	0.50171200
C	12.11934600	-0.17873900	0.14939000
H	9.87146600	-2.66809000	0.78110300
H	12.30652100	-2.25019700	0.76536600
H	13.19160800	-0.00732100	0.14630500

Optimized coordinates for An-TDPP singlet state at RB3LYP/6-31G(d,p) level of theory and basis set.

C	-0.13195800	0.00036900	1.79506800
C	-0.57361100	0.00008500	0.41992500
C	0.57361000	-0.00008300	-0.41992800
C	1.72387700	0.00008400	0.36987700
C	-1.72387800	-0.00008400	-0.36988000
C	0.13195700	-0.00037100	-1.79507100
N	-1.30050000	-0.00035800	-1.69722400
N	1.30049900	0.00035600	1.69722100
C	-2.09929100	-0.00061500	-2.90982700
H	-2.72800800	0.89195100	-2.97835300
H	-1.38913500	-0.00078700	-3.73827300
C	2.09928900	0.00060000	2.90982500
H	1.38913300	0.00077200	3.73827100
H	2.72800100	-0.89197200	2.97834500
O	0.72082300	-0.00059200	-2.87427600
O	-0.72082400	0.00058700	2.87427300
C	-3.08986800	0.00000400	0.08699100
C	-3.47587000	0.00027200	1.42030600
C	-4.87892600	0.00030700	1.60013500
H	-2.75017800	0.00043500	2.22767200
C	-5.59592500	0.00006800	0.42520900
H	-5.35985200	0.00050100	2.57153800
C	3.08986800	-0.00000500	-0.08699400
C	3.47587000	-0.00027400	-1.42030900
C	4.87892700	-0.00030600	-1.60013700
H	2.75017900	-0.00043800	-2.22767500
C	5.59592600	-0.00006300	-0.42521000
H	5.35985400	-0.00049800	-2.57154000
S	4.51630500	0.00020600	0.94837200
S	-4.51630600	-0.00020400	-0.94837400
C	-7.10708000	2.49784700	0.23721200
C	-7.76320200	1.22941300	0.14100200
C	-7.81174100	3.66925500	0.14311200
C	-7.06723600	0.00003500	0.23274400
C	-9.19455300	1.22150200	-0.05480800
C	-9.22218100	3.65777800	-0.05358200
H	-7.29157300	4.61940000	0.22077300
C	-7.76318400	-1.22937200	0.14125300
C	-9.86788700	-0.00002500	-0.14918300
C	-9.89038700	2.46726700	-0.14819200
H	-9.76210000	4.59709200	-0.12580200
C	-7.10704500	-2.49777600	0.23774200
C	-9.19453400	-1.22152200	-0.05456300
H	-10.94510300	-0.00004800	-0.29898100
H	-10.96685200	2.44563600	-0.29583400
C	-7.81168900	-3.66921300	0.14387900
H	-6.03457900	-2.51946200	0.39524100
C	-9.89034900	-2.46731600	-0.14769700
C	-9.22212700	-3.65779800	-0.05283600
H	-7.29150900	-4.61933500	0.22175000
H	-10.96681300	-2.44573200	-0.29535200
H	-9.76203100	-4.59713400	-0.12486700
C	7.10703600	2.49778000	-0.23763600
C	7.76318000	1.22937500	-0.14119800
C	7.81167500	3.66921700	-0.14372100
C	7.06723600	-0.00003100	-0.23274300
C	9.19452900	1.22152200	0.05462000
C	9.22211200	3.65779900	0.05299900
H	7.29149100	4.61934000	-0.22155300
C	7.76320800	-1.22941000	-0.14105500
C	9.86788700	0.00002400	0.14918600
C	9.89033900	2.46731500	0.14781000
H	9.76201200	4.59713400	0.12507200
C	7.10709000	-2.49784300	-0.23731600
C	9.19455800	-1.22150100	0.05475600
H	10.94510300	0.00004500	0.29898400
H	10.96680300	2.44572900	0.29546600
C	7.81175700	-3.66925200	-0.14326600

H	6.03462000	-2.51957400	-0.39478900
C	9.89039800	-2.46726800	0.14808500
C	9.22219700	-3.65777800	0.05342600
H	7.29159200	-4.61939600	-0.22096600
H	10.96686300	-2.44563900	0.29572700
H	9.76211900	-4.59709200	0.12560500
H	6.03457000	2.51946900	-0.39513800
H	-6.03461100	2.51958000	0.39468800
H	2.72800800	0.89319400	2.97798000
H	-2.72800200	-0.89321400	-2.97797800

Optimized coordinates for Py-TDPP singlet state at RB3LYP/6-31G(d,p) level of theory and basis set.

C	0.08431800	0.68272300	1.11888600
C	0.52433300	-0.47739500	0.37937200
C	-0.62271900	-1.17955300	-0.07841000
C	-1.77353500	-0.50775900	0.33959100
C	1.67528000	-1.14522400	-0.04492800
C	-0.18263700	-2.33854400	-0.81989900
N	1.24913600	-2.26103900	-0.76340400
N	-1.34754900	0.60845400	1.05745300
C	2.04614600	-3.28627100	-1.41251500
H	2.67407800	-3.82254300	-0.69500000
H	1.33490200	-3.98492600	-1.85602700
C	-2.14425700	1.62809500	1.71589100
H	-1.43283300	2.31436700	2.17804700
H	-2.76143200	2.18149900	1.00206600
O	-0.77327600	-3.24580400	-1.40298000
O	0.67496200	1.58697800	1.70671100
C	3.03865500	-0.76405100	0.20388200
C	3.42697900	0.35054800	0.93825800
C	4.82471900	0.50640800	1.03064200
H	2.70136500	1.01615400	1.39537300
C	5.54944400	-0.47416800	0.37818200
H	5.29803400	1.29890000	1.59759100
C	-3.13711800	-0.88747600	0.08688300
C	-3.52520900	-2.01243300	-0.63052700
C	-4.92299900	-2.15097200	-0.74415700
H	-2.79987700	-2.69660700	-1.05971300
C	-5.64846300	-1.14310500	-0.13712800
H	-5.40104300	-2.96250300	-1.28048000
S	-4.56168100	0.00115000	0.62126300
S	4.46441500	-1.61820200	-0.37983300
C	7.00286500	-0.67594700	0.30411300
C	7.90543000	0.39047400	0.02828900
C	7.51925900	-1.96544400	0.52901900
C	9.31415800	0.13148800	0.04835100
C	7.47203700	1.71935900	-0.31016100
C	8.88208600	-2.21811200	0.53033400
H	6.82809800	-2.77407500	0.74491100
C	10.24429300	1.18845100	-0.19805400
C	9.80498300	-1.18462800	0.30850100
C	8.36072700	2.72301700	-0.55094700
H	6.41022900	1.91465900	-0.39400200
H	9.24572900	-3.22299400	0.72594300
C	9.77619000	2.50500200	-0.48764600
C	11.65045800	0.93410000	-0.16082900
C	11.22178300	-1.41203100	0.33556400
H	8.00161700	3.71516900	-0.81114800
C	10.70681300	3.53078600	-0.72112900
C	12.54352200	1.99135400	-0.39821400
C	12.10463600	-0.39979500	0.11570400
H	11.57678200	-2.41872900	0.53875600
C	12.07497000	3.27450000	-0.67366400
H	10.34552200	4.53136200	-0.94240100
H	13.61201300	1.79629600	-0.36647000
H	13.17480600	-0.58653600	0.14183900
H	12.78140100	4.07895600	-0.85546600
C	-7.11257100	-1.07525800	0.00009000

C	-7.88469400	0.09588800	-0.23804600
C	-7.76925700	-2.25760100	0.38864700
C	-9.30161900	0.04941600	-0.02930600
C	-7.31934000	1.32705100	-0.72138100
C	-9.14231600	-2.30646300	0.57325100
H	-7.17160700	-3.14291900	0.57941900
C	-10.10014000	1.21593200	-0.24089800
C	-9.93307900	-1.16278800	0.38601200
C	-8.08351700	2.43540800	-0.92733100
H	-6.26024800	1.36468800	-0.94335600
H	-9.61336300	-3.23369500	0.88748000
C	-9.49568100	2.43095900	-0.68204300
C	-11.51140900	1.17387000	-0.01682800
C	-11.35226900	-1.17707900	0.60016900
H	-7.62605300	3.34869600	-1.29834800
C	-10.29719800	3.56737000	-0.87916900
C	-12.27226400	2.33553600	-0.22462000
C	-12.10699400	-0.06042800	0.41183500
H	-11.81409800	-2.10726900	0.91986400
C	-11.66998200	3.51810700	-0.64967100
H	-9.83209300	4.48968700	-1.21631700
H	-13.34436400	2.30167900	-0.05086400
H	-13.18027700	-0.08568600	0.57988700
H	-12.27559700	4.40586700	-0.80572900
H	-2.78412100	1.19960300	2.49286500
H	2.67517700	-2.86747200	-2.20358200

Optimized coordinates for TDPP singlet state at RB3LYP/6-31G(d,p) level of theory and basis set.

C	0.09942100	-1.79714000	0.00004100
C	0.56596900	-0.43032700	0.00009100
C	-0.56596900	0.43032700	0.00009100
C	-1.72999300	-0.33818500	0.00003700
C	1.72999300	0.33818600	0.00004000
C	-0.09942100	1.79714000	0.00004400
N	1.33132200	1.67292300	0.00001600
N	-1.33132200	-1.67292300	0.00001100
C	2.15188000	2.87062500	0.00002300
H	2.78142000	2.92755600	-0.89287100
H	1.45693700	3.71187200	0.00004800
C	-2.15188100	-2.87062500	0.00001600
H	-1.45693700	-3.71187200	0.00004000
H	-2.78145200	-2.92752300	0.89288900
O	-0.66798900	2.88698600	0.00003400
O	0.66798900	-2.88698600	0.00003000
C	3.08728500	-0.14476400	0.00000800
C	3.44935900	-1.48492300	0.00003100
C	4.85027000	-1.68912800	-0.00001500
H	2.70827300	-2.27805100	0.00007600
C	5.56345500	-0.51826500	-0.00007100
H	5.31377400	-2.66857400	-0.00000600
C	-3.08728500	0.14476400	0.00000500
C	-3.44935900	1.48492300	0.00002900
C	-4.85027100	1.68912700	-0.00001800
H	-2.70827400	2.27805100	0.00007600
C	-5.56345500	0.51826400	-0.00007700
H	-5.31377500	2.66857300	-0.00000800
S	-4.53205400	-0.86708900	-0.00007800
S	4.53205400	0.86708900	-0.00007100
H	-2.78141900	-2.92755400	-0.89287900
H	2.78145000	2.92752300	0.89289700
H	6.63644700	-0.38449500	-0.00011200
H	-6.63644700	0.38449300	-0.00011900

Optimized coordinates for Th-TDPP singlet state at RB3LYP/6-31G(d,p) level of theory and basis set.

C	-0.19921400	-1.78871400	-0.01375400
C	-0.58797100	-0.39742700	-0.01322400
C	0.58797000	0.39742500	-0.01322200

C	1.71020500	-0.43596300	-0.01320900
C	-1.71020500	0.43596100	-0.01320900
C	0.19921400	1.78871100	-0.01375000
N	-1.23457400	1.74657100	-0.01339900
N	1.23457400	-1.74657400	-0.01340300
C	-1.98550300	2.98903500	-0.01318700
H	-2.61028100	3.08209900	0.88008200
H	-1.24366200	3.78929000	-0.01321100
C	1.98550200	-2.98903700	-0.01319300
H	1.24366100	-3.78929200	-0.01321900
H	2.61063300	-3.08214100	-0.90617000
O	0.83030300	2.84416100	-0.01461900
O	-0.83030300	-2.84416300	-0.01462600
C	-3.08632800	0.02987100	-0.01198300
C	-3.52380800	-1.29046500	-0.01355200
C	-4.92402600	-1.42111200	-0.01832000
H	-2.82719600	-2.12308100	-0.01704700
C	-5.60415100	-0.21489800	-0.01980700
H	-5.43073000	-2.37938200	-0.03320000
C	3.08632800	-0.02987200	-0.01198100
C	3.52380700	1.29046400	-0.01354700
C	4.92402500	1.42111200	-0.01831400
H	2.82719400	2.12307900	-0.01704000
C	5.60415100	0.21489800	-0.01980300
H	5.43072800	2.37938300	-0.03319100
S	4.47362900	-1.12047600	-0.00540700
S	-4.47362800	1.12047600	-0.00540700
H	2.61028000	-3.08210400	0.88007600
H	-2.61063300	3.08214100	-0.90616400
C	-7.02901600	0.02365700	-0.02773000
C	-7.71220500	1.20879600	-0.21613300
S	-8.16472700	-1.29291500	0.22836400
C	-9.12517200	1.06554700	-0.16113100
H	-7.21219300	2.15263400	-0.40212800
C	-9.52009600	-0.22469700	0.06761900
H	-9.81932600	1.88743000	-0.29144100
H	-10.52428000	-0.61548200	0.15289600
C	7.02901700	-0.02365500	-0.02772600
C	7.71220600	-1.20879400	-0.21613100
S	8.16472600	1.29291700	0.22837100
C	9.12517300	-1.06554300	-0.16112800
H	7.21219500	-2.15263200	-0.40212800
C	9.52009600	0.22470000	0.06762400
H	9.81932700	-1.88742600	-0.29143900
H	10.52428000	0.61548600	0.15290200

Optimized coordinates for Flu-TDPP singlet state at RB3LYP/6-31G(d,p) level of theory and basis set.

C	0.04394500	-1.79913600	-0.23023300
C	-0.52927900	-0.47345800	-0.23119700
C	0.52928000	0.47344900	-0.23119500
C	1.75345900	-0.20046000	-0.23100100
C	-1.75345800	0.20045100	-0.23099800
C	-0.04394300	1.79912700	-0.23022200
N	-1.45916300	1.56324700	-0.23081300
N	1.45916400	-1.56325600	-0.23082400
C	-2.37104700	2.69267100	-0.23103100
H	-3.00322800	2.70017200	0.66180800
H	-1.74409100	3.58579800	-0.23130000
C	2.37104700	-2.69267900	-0.23105400
H	1.74409200	-3.58580600	-0.23132900
H	3.00345400	-2.69986600	-1.12373200
O	0.43776800	2.93078100	-0.22852200
O	-0.43776700	-2.93078900	-0.22854000
C	-3.06372600	-0.38695100	-0.23162100
C	-3.31938600	-1.75388300	-0.22898300
C	-4.69083100	-2.07053700	-0.22641200
H	-2.51735300	-2.48538300	-0.21885000

C	-5.52894600	-0.96953700	-0.22617800
H	-5.06172200	-3.08801400	-0.19363800
C	3.06372600	0.38694200	-0.23162300
C	3.31938500	1.75387600	-0.22897800
C	4.69083000	2.07053100	-0.22640700
H	2.51735100	2.48537400	-0.21883900
C	5.52894500	0.96953100	-0.22618000
H	5.06172000	3.08800700	-0.19362800
S	4.58535500	-0.50307500	-0.24154700
S	-4.58535300	0.50306900	-0.24153800
H	3.00323100	-2.70018900	0.66178300
H	-3.00345500	2.69986500	-1.12370700
C	-7.72099700	-2.04267400	-0.70769400
C	-6.99146100	-0.93658300	-0.22151800
C	-7.70218800	0.18270200	0.26393700
C	-9.08749500	0.18118500	0.25826400
C	-9.80104100	-0.93490600	-0.22353100
C	-9.11213900	-2.05028500	-0.70666700
H	-7.18459200	-2.89480100	-1.11178300
H	-7.15568000	1.03363900	0.66189300
H	-9.64785500	-2.91389500	-1.09020800
C	-10.04421100	1.27360300	0.73701500
C	-11.39872300	0.62341000	0.45440100
C	-12.67304700	1.13271200	0.67601200
C	-13.78671100	0.35473300	0.33836600
C	-13.62336000	-0.91954000	-0.21518100
C	-12.34683800	-1.43751300	-0.44058800
C	-11.23558400	-0.66088000	-0.10386800
H	-12.81166800	2.12143100	1.10568800
H	-14.78649900	0.74369300	0.50768000
H	-14.49758800	-1.51068800	-0.47170400
H	-12.22487500	-2.42766000	-0.87074700
C	-9.86335400	2.57538400	-0.07551100
H	-8.86608300	2.99772900	0.08639100
H	-9.98742600	2.39207000	-1.14636800
H	-10.59916100	3.32628800	0.23049500
C	-9.86143700	1.56186700	2.24404000
H	-9.98458300	0.65152700	2.83717700
H	-8.86407800	1.96783900	2.44291700
H	-10.59702400	2.29640600	2.58796500
C	13.62335800	0.91954400	-0.21519900
C	13.78671200	-0.35472200	0.33836300
C	12.67304900	-1.13269800	0.67602000
C	11.39872300	-0.62340000	0.45440700
C	11.23558200	0.66088300	-0.10387800
C	12.34683500	1.43751300	-0.44060900
H	14.49758500	1.51068900	-0.47173100
H	14.78650000	-0.74367900	0.50767900
H	12.81167100	-2.12141200	1.10570900
H	12.22487000	2.42765500	-0.87078000
C	10.04421300	-1.27359000	0.73703300
C	9.08749500	-0.18118000	0.25826900
C	7.70218800	-0.18269800	0.26394400
C	6.99145900	0.93658000	-0.22152200
C	7.72099200	2.04266700	-0.70771200
C	9.11213500	2.05027900	-0.70668800
C	9.80103900	0.93490700	-0.22354000
H	7.15568200	-1.03363000	0.66191200
H	7.18458500	2.89478800	-1.11181000
H	9.64784900	2.91388500	-1.09024000
C	9.86144300	-1.56183100	2.24406300
H	9.98459000	-0.65148300	2.83718600
H	8.86408500	-1.96780200	2.44294900
H	10.59703200	-2.29636500	2.58799700
C	9.86335500	-2.57538300	-0.07547300
H	8.86608500	-2.99772700	0.08643800
H	9.98742500	-2.39208600	-1.14633400
H	10.59916300	-3.32628200	0.23054200

Optimized coordinates for TPAOMe-TDPP singlet state at RB3LYP/6-31G(d,p) level of theory and basis set.

C	-0.08874000	-1.66147800	0.76171400
C	-0.56410100	-0.41991300	0.19928700
C	0.56035200	0.36403500	-0.17399900
C	1.73325600	-0.33365700	0.12924300
C	-1.73699200	0.27739000	-0.10453800
C	0.08503600	1.60628700	-0.73479900
N	-1.34331700	1.49262300	-0.66490300
N	1.33963200	-1.54837100	0.69056500
C	-2.17033700	2.58210700	-1.14983600
H	-2.79092200	3.00275600	-0.35304900
H	-1.47974900	3.34811000	-1.50648300
C	2.16683300	-2.63671100	1.17782500
H	1.47645100	-3.40153100	1.53738400
H	2.78640700	-3.06014900	0.38167000
O	0.64734300	2.59984100	-1.19448900
O	-0.65106500	-2.65345100	1.22460200
C	-3.08553700	-0.16238200	0.11095800
C	-3.44193800	-1.38136700	0.67782600
C	-4.83216700	-1.57053600	0.78300900
H	-2.69530100	-2.09653300	1.00893700
C	-5.59002800	-0.51452000	0.30570800
H	-5.27386800	-2.45441500	1.22755000
C	3.08174200	0.10339100	-0.09205000
C	3.43773200	1.32466600	-0.65459900
C	4.82789400	1.51569000	-0.75704600
H	2.69090100	2.04874800	-0.96530200
C	5.58553800	0.45866600	-0.28148100
H	5.27141500	2.42540700	-1.14402100
S	4.53617300	-0.81638200	0.29797200
S	-4.53983900	0.74681400	-0.30288300
C	-7.04309400	-0.38199300	0.26120600
C	-7.68106300	0.86754200	0.14146000
C	-7.87243400	-1.51915800	0.33952100
C	-9.06306400	0.98051300	0.09675700
H	-7.08409000	1.77342800	0.08737000
C	-9.25457400	-1.41564200	0.31230900
H	-7.42486400	-2.50482700	0.41648700
C	-9.88355000	-0.16067900	0.18617900
H	-9.51759500	1.95934800	-0.00429900
H	-9.86033100	-2.31181400	0.38318800
C	7.03803200	0.32748100	-0.23674300
C	7.68681300	-0.55748900	0.64573500
C	7.85578000	1.09822100	-1.08794500
C	9.06924800	-0.66495500	0.68582200
H	7.09867100	-1.14982300	1.34099100
C	9.23771700	0.99559900	-1.05843900
H	7.39662000	1.76420600	-1.81164800
C	9.87819300	0.11094800	-0.16724200
H	9.53413900	-1.34288500	1.39241400
H	9.83432700	1.59015700	-1.74087500
N	-11.28610100	-0.05238000	0.15154400
N	11.28029400	0.00416900	-0.13498100
C	11.91009600	-1.22228900	0.23307900
C	12.94670100	-1.23774400	1.17099600
C	11.52008000	-2.43697100	-0.35779800
C	13.59129600	-2.42797800	1.51428000
H	13.25917800	-0.30724000	1.63347800
C	12.14050500	-3.62655400	-0.00576300
H	10.72333900	-2.44033600	-1.09438300
C	13.18554300	-3.63316500	0.93068600
H	14.39389500	-2.40122700	2.24144600
H	11.84339100	-4.56715100	-0.45752300
C	12.10494400	1.12654200	-0.44618700
C	11.87185200	2.37633900	0.13670200
C	13.18390400	0.99089000	-1.33622500
C	12.67744500	3.47547800	-0.16809000
H	11.04750800	2.49460900	0.83241100
C	14.00234100	2.07246000	-1.62704800

H	13.37701900	0.02674100	-1.79491700
C	13.75362100	3.32704000	-1.05028100
H	12.46448300	4.42964600	0.29872400
H	14.83721900	1.97379300	-2.31286000
C	-12.08539200	-1.09705800	-0.40179000
C	-13.21021900	-1.57531600	0.29161700
C	-11.78126300	-1.65453200	-1.64793400
C	-14.00382800	-2.57570100	-0.25033200
H	-13.45875200	-1.15124300	1.25892400
C	-12.56195400	-2.67755600	-2.19005900
H	-10.92060900	-1.28994100	-2.19921100
C	-13.68407800	-3.14093200	-1.49400600
H	-14.87385200	-2.94847300	0.27995700
H	-12.29348700	-3.08901200	-3.15562500
C	-11.93920600	1.11624200	0.64545700
C	-12.93055700	1.75169100	-0.10813500
C	-11.61837300	1.63867900	1.91072000
C	-13.59788500	2.87700000	0.38022900
H	-13.18959100	1.35818700	-1.08559300
C	-12.26093800	2.76919300	2.39337700
H	-10.85770200	1.15134700	2.51178100
C	-13.26052300	3.39698000	1.63459900
H	-14.36415700	3.34054600	-0.22939200
H	-12.01698500	3.17756900	3.36847100
O	14.61079100	4.32734100	-1.41092100
O	13.73613400	-4.85421900	1.19740500
O	-13.83860300	4.49449500	2.20619300
O	-14.52236500	-4.12615300	-1.93237400
C	14.40489000	5.61737400	-0.85776300
H	15.18548900	6.25347600	-1.27733100
H	13.42278500	6.02405700	-1.13146700
H	14.49618300	5.60927000	0.23607500
C	14.79617300	-4.91960100	2.13784400
H	14.47900700	-4.57691100	3.13123500
H	15.08291500	-5.97048200	2.19639800
H	15.66206400	-4.32723900	1.81494200
C	-14.85557800	5.16870500	1.48267400
H	-15.17445700	5.99941000	2.11375300
H	-14.48155400	5.56365100	0.52925900
H	-15.71509700	4.51483700	1.28612400
C	-14.24480900	-4.73338700	-3.18408300
H	-15.02220700	-5.48380600	-3.33418800
H	-14.28293400	-4.00612500	-4.00538200
H	-13.26357600	-5.22533600	-3.18759500
H	-2.81079900	2.26473100	-1.97817700
H	2.80828100	-2.31686300	2.00438800

Optimized coordinates for TPAOMe-TTDPP singlet state at RB3LYP/6-31G(d,p) level of theory and basis set.

C	-0.43666900	-1.62359500	0.64762100
C	-0.63142500	-0.25777100	0.22091400
C	0.63790300	0.29998000	-0.07599400
C	1.63462800	-0.65993400	0.14294000
C	-1.62903900	0.69457900	-0.01760600
C	0.44018400	1.66006400	-0.51708500
N	-0.97809200	1.85887000	-0.43808700
N	0.98294200	-1.82142000	0.56542800
C	-1.51894600	3.19326400	-0.69948600
H	-0.77357262	3.79253078	-1.17927537
H	-2.37554886	3.11260038	-1.33558735
C	1.52298300	-3.14268200	0.88030100
H	2.40983525	-3.03445365	1.46909613
H	0.79610495	-3.70334472	1.43003331
O	1.21172300	2.53817000	-0.90734300
O	-1.20408300	-2.50658200	1.02976400
C	-3.04471800	0.48249600	0.09160100
C	-3.63513700	-0.66854900	0.60680800
C	-5.03876300	-0.66102500	0.55044900
H	-3.04151300	-1.49072500	0.99297600

C	-5.57704300	0.48471700	-0.01553400
H	-5.65139700	-1.47869100	0.91279000
C	3.04911100	-0.46206300	-0.00088100
C	3.63860200	0.70222000	-0.48708800
C	5.04273000	0.66685600	-0.49822300
H	3.04367400	1.54831800	-0.81578700
C	5.58355600	-0.51586500	-0.01724900
H	5.65424900	1.48620400	-0.85860800
S	4.30480300	-1.61219400	0.46257400
S	-4.29632500	1.58449500	-0.48295100
C	-10.91094700	0.49388400	-0.20823300
C	-11.89484700	1.49916900	-0.28550600
C	-11.35984300	-0.82705500	-0.01864100
C	-13.24705900	1.20358400	-0.19641900
H	-11.59475100	2.53463500	-0.41146000
C	-12.70904300	-1.13085100	0.09116900
H	-10.63845300	-1.63716100	0.03943000
C	-13.68616300	-0.12096400	-0.00175700
H	-13.97570500	2.00282000	-0.26996200
H	-13.01589000	-2.15893900	0.24561800
C	10.91793000	-0.56735200	0.07486500
C	11.87985500	-1.29752500	0.80022900
C	11.38617100	0.49418100	-0.72280800
C	13.23046300	-0.99002100	0.73217700
H	11.55827900	-2.09829300	1.45892100
C	12.73524700	0.80776800	-0.80041600
H	10.68310500	1.06529800	-1.32261600
C	13.69016800	0.07140200	-0.07261300
H	13.94060800	-1.56110600	1.31933900
H	13.06118100	1.62020500	-1.43977400
N	-15.05693100	-0.42478000	0.10048100
N	15.05973300	0.38639400	-0.14571300
C	16.05019200	-0.62690800	0.02080400
C	17.13638200	-0.42810200	0.87834300
C	15.96935200	-1.83633300	-0.69161400
C	18.12748500	-1.40085700	1.02496900
H	17.21257400	0.50137900	1.43297900
C	16.93808500	-2.81664700	-0.53454800
H	15.13759900	-2.00192100	-1.36848000
C	18.02935900	-2.60670700	0.32216300
H	18.95730000	-1.21008100	1.69482000
H	16.88065600	-3.75216600	-1.08102200
C	15.48894300	1.72960300	-0.36490100
C	14.96265600	2.78654700	0.38480500
C	16.46732100	2.01432500	-1.33262400
C	15.38105900	4.10136200	0.16994400
H	14.21204300	2.58169600	1.14120900
C	16.90461500	3.31490100	-1.53653800
H	16.88506400	1.20404800	-1.92103700
C	16.36142900	4.37221000	-0.79128400
H	14.94773100	4.89551900	0.76595300
H	17.66015600	3.54069800	-2.28170900
C	-15.56016100	-1.68448900	-0.34244000
C	-16.42721100	-2.43068200	0.47378300
C	-15.21925400	-2.19611100	-1.59870700
C	-16.93731400	-3.64584900	0.04078200
H	-16.70091500	-2.04572500	1.45060500
C	-15.71025800	-3.42926900	-2.03261400
H	-14.55675300	-1.62800200	-2.24361700
C	-16.57916200	-4.16003100	-1.21446000
H	-17.60766600	-4.22591900	0.66628900
H	-15.42073000	-3.79668500	-3.00980400
C	-15.97715300	0.52724100	0.63194600
C	-17.17533600	0.80772500	-0.03163500
C	-15.71191800	1.18466400	1.84615300
C	-18.09758600	1.71384800	0.49609400
H	-17.39397600	0.30612500	-0.96863300
C	-16.61263800	2.10284300	2.36555000
H	-14.79070900	0.97092700	2.37821100

C	-17.81677200	2.37314400	1.69778200
H	-19.01801700	1.90249100	-0.04327300
H	-16.41300500	2.61383900	3.30157800
O	16.85197500	5.61443600	-1.07725400
O	18.93213600	-3.62914800	0.39567600
O	-18.63843600	3.28327300	2.29977900
O	-17.12699100	-5.36814100	-1.54020900
C	16.33586100	6.72073000	-0.35454000
H	16.85270800	7.60054100	-0.74030200
H	15.25599400	6.83955100	-0.51133100
H	16.53298900	6.63028300	0.72150100
C	20.05541900	-3.47004100	1.24723700
H	19.75514000	-3.33922600	2.29490800
H	20.63729300	-4.38802300	1.15362700
H	20.67660800	-2.61721200	0.94436200
C	-19.86997600	3.59365700	1.66806500
H	-20.36135100	4.32696600	2.30904000
H	-19.71685300	4.03052500	0.67275900
H	-20.51254800	2.70858500	1.57478000
C	-16.79680800	-5.93564600	-2.79769800
H	-17.32941600	-6.88630300	-2.84873700
H	-17.12068400	-5.29637800	-3.62919000
H	-15.71877900	-6.12126800	-2.88854500
C	6.96689900	-0.89041500	0.11829300
C	7.51132700	-2.10801500	0.48298400
S	8.25102900	0.27109200	-0.18584500
C	8.92463200	-2.11858800	0.50422100
H	6.90501000	-2.97833200	0.70907800
C	9.50146500	-0.91196000	0.15786200
H	9.50866800	-3.00188000	0.73398000
C	-6.95870400	0.82585700	-0.23200400
C	-7.49796200	1.94824000	-0.83294400
S	-8.24787700	-0.24818500	0.29244900
C	-8.91038700	1.94578600	-0.88469400
H	-6.88827900	2.74745000	-1.23996800
C	-9.49302200	0.82485800	-0.32547200
H	-9.48713000	2.73630800	-1.34992000
H	-1.80318226	3.65035317	0.22527302
H	1.75833606	-3.65745363	-0.02772934

Optimized coordinates for NTT singlet state at RB3LYP/6-31G(d,p) level of theory and basis set.

C	-2.18316300	-1.36681900	0.00000600
C	-0.76586200	-1.68984100	0.00000700
C	0.24035500	-0.65926100	0.00000200
C	-0.24035500	0.65926100	-0.00000300
C	-1.63107000	0.96601500	-0.00000400
C	-2.63239300	0.01362100	0.00000100
C	1.63107000	-0.96601500	0.00000300
C	0.76586200	1.68984100	-0.00000700
C	2.18316300	1.36681900	-0.00000600
C	2.63239300	-0.01362100	-0.00000100
H	1.88831300	-2.02001200	0.00000900
H	-1.88831300	2.02001200	-0.00001000
N	0.50994400	2.99671300	-0.00001300
N	2.96125100	2.45054100	-0.00001100
S	1.96606600	3.76322900	-0.00001600
N	-2.96125100	-2.45054100	0.00001100
N	-0.50994400	-2.99671300	0.00001200
S	-1.96606600	-3.76322900	0.00001600
C	4.04641200	-0.37266400	-0.00000200
C	5.15954200	0.44144100	0.00001700
S	4.54562000	-2.06338000	-0.00002800
C	6.40421500	-0.25988800	0.00001200
H	5.08044700	1.51998100	0.00003400
C	6.21894000	-1.61752000	-0.00001200
C	-4.04641200	0.37266400	0.00000200
C	-5.15954200	-0.44144100	-0.00002400

S	-4.54562000	2.06338000	0.00004000
C	-6.40421500	0.25988800	-0.00001700
H	-5.08044700	-1.51998100	-0.00004800
C	-6.21894000	1.61752000	0.00001700
C	7.74624500	0.42105800	0.00003100
H	7.86667000	1.06074100	-0.88157500
H	7.86666100	1.06070900	0.88166000
H	8.56189200	-0.30661900	0.00002200
C	-7.74624500	-0.42105800	-0.00004200
H	-7.86665900	-1.06070400	-0.88167600
H	-7.86667200	-1.06074600	0.88155900
H	-8.56189200	0.30661900	-0.00003100
H	6.97672400	-2.38951700	-0.00002100
H	-6.97672400	2.38951700	0.00003000

Optimized coordinates for 2N-NTT singlet state at RB3LYP/6-31G(d,p) level of theory and basis set.

C	-1.56947500	2.04101000	-0.08888800
C	-0.13022000	1.85131100	-0.01385100
C	0.45431500	0.53500200	0.02123000
C	-0.45431500	-0.53500500	-0.02124100
C	-1.86207500	-0.33976200	-0.09588300
C	-2.46937900	0.90238300	-0.12978400
C	1.86207500	0.33975900	0.09587200
C	0.13022000	-1.85131500	0.01383900
C	1.56947500	-2.04101300	0.08887500
C	2.46937900	-0.90238700	0.12977200
H	2.46960000	1.23796900	0.13095200
H	-2.46960000	-1.23797200	-0.13096500
N	-0.56285300	-2.98786100	-0.01871600
N	1.92245500	-3.32715700	0.11060500
S	0.53474600	-4.21274100	0.04177500
N	-1.92245500	3.32715300	-0.11061800
N	0.56285300	2.98785700	0.01870300
S	-0.53474700	4.21273800	-0.04178800
C	3.91600400	-1.05660000	0.19803200
C	4.67258300	-2.20078600	0.34653400
S	4.97672200	0.33985500	0.09440300
C	6.07939700	-1.98837900	0.38834900
H	4.22014000	-3.17877700	0.43838200
C	6.41415900	-0.64988600	0.26548500
C	-3.91600400	1.05659700	-0.19804300
C	-4.67258200	2.20078300	-0.34655500
S	-4.97672300	-0.33985500	-0.09439900
C	-6.07939600	1.98837600	-0.38836900
H	-4.22013800	3.17877300	-0.43841200
C	-6.41415900	0.64988500	-0.26548900
C	7.04943800	-3.12284900	0.59599500
H	6.60376400	-3.89181100	1.23417700
H	7.31815500	-3.60743400	-0.35095300
H	7.97534800	-2.78259300	1.06570300
C	-7.04943600	3.12284500	-0.59602500
H	-7.31815000	3.60744100	0.35091700
H	-6.60376300	3.89179900	-1.23421700
H	-7.97534600	2.78258400	-1.06572900
C	8.81822700	-0.57988100	-0.38306100
C	10.09687700	0.03336200	-0.38642000
C	7.73389600	-0.00432200	0.26396200
C	11.21195800	-0.55191900	-1.04640600
C	10.27231400	1.28682000	0.28414300
C	7.92453400	1.24892800	0.92391800
C	12.44077900	0.06790000	-1.03748800
H	11.07757400	-1.50083400	-1.55906200
C	11.55245700	1.89943100	0.27620100
C	9.14889500	1.86909900	0.93089100
H	7.08624600	1.70164000	1.44417800
C	12.61382100	1.30471800	-0.36895900
H	13.28474300	-0.39040200	-1.54450900
H	11.68114400	2.84933100	0.78851600

H	9.27416300	2.81855800	1.44476200
H	13.58866900	1.78319000	-0.36907400
C	-8.81822600	0.57989000	0.38306200
C	-10.09687600	-0.03335300	0.38643100
C	-7.73389700	0.00432200	-0.26395500
C	-11.21195600	0.55193700	1.04641100
C	-10.27231500	-1.28682000	-0.28411500
C	-7.92453600	-1.24893600	-0.92389500
C	-12.44077700	-0.06788200	1.03750400
H	-11.07757200	1.50085900	1.55905400
C	-11.55245700	-1.89943100	-0.27616200
C	-9.14889700	-1.86910800	-0.93085700
H	-7.08624900	-1.70165500	-1.44415000
C	-12.61382000	-1.30471000	0.36899300
H	-13.28474100	0.39042600	1.54452100
H	-11.68114500	-2.84933900	-0.78846300
H	-9.27416600	-2.81857400	-1.44471500
H	-13.58866800	-1.78318200	0.36911600
H	-8.69103000	1.50841600	0.93000600
H	8.69103100	-1.50839900	-0.93001800

Optimized coordinates for Flu-NTT singlet state at RB3LYP/6-31G(d,p) level of theory and basis set.

C	-1.49843100	-2.08600200	-0.20080500
C	-0.06837700	-1.85284500	-0.08266400
C	0.47126100	-0.52045200	0.01379300
C	-0.47125800	0.52048700	-0.01383200
C	-1.86947600	0.28282600	-0.13123800
C	-2.43445500	-0.97633200	-0.22403100
C	1.86948000	-0.28279100	0.13119800
C	0.06838100	1.85288000	0.08262100
C	1.49843600	2.08603600	0.20075900
C	2.43446000	0.97636600	0.22398800
H	2.50554100	-1.16149100	0.15135400
H	-2.50553800	1.16152500	-0.15139900
N	-0.66092200	2.96694300	0.07269400
N	1.80814200	3.38122400	0.27669600
S	0.39386200	4.22363600	0.20412700
N	-1.80813800	-3.38118900	-0.27674700
N	0.66092700	-2.96690800	-0.07274000
S	-0.39385800	-4.22360100	-0.20417900
C	3.87288800	1.17457200	0.33295900
C	4.58698700	2.33430100	0.55446400
S	4.98326800	-0.17911500	0.18610400
C	5.99874600	2.16582700	0.62106400
H	4.09975900	3.29140000	0.68209400
C	6.38177200	0.84635500	0.44419800
C	-3.87288300	-1.17453800	-0.33300200
C	-4.58697600	-2.33425700	-0.55457800
S	-4.98326800	0.17913300	-0.18605600
C	-5.99873600	-2.16578500	-0.62116500
H	-4.09974400	-3.29134600	-0.68227000
C	-6.38176800	-0.84632700	-0.44421200
C	6.92472400	3.32012300	0.90819600
H	7.18613600	3.86966700	-0.00480700
H	6.44390400	4.03367200	1.58425900
H	7.85649300	2.98713100	1.37118200
C	-6.92470800	-3.32006800	-0.90837200
H	-6.44387100	-4.03358900	-1.58445300
H	-7.18614600	-3.86965200	0.00460100
H	-7.85646400	-2.98705400	-1.37136800
C	13.74987500	-2.52625400	0.43987000
C	14.40084900	-1.50910900	-0.26589200
C	13.69653700	-0.37883700	-0.69721500
C	12.33900000	-0.27807200	-0.41522700
C	11.68423700	-1.30388000	0.29620800
C	12.38669000	-2.43187100	0.72627800
H	14.31009100	-3.39706600	0.76771900
H	15.46176200	-1.59718300	-0.48109400

H	14.21206900	0.40560100	-1.24533100
H	11.88543400	-3.22461100	1.27460400
C	11.36557400	0.84417700	-0.77643200
C	10.06646900	0.31574600	-0.16673900
C	8.80826200	0.89916800	-0.17515000
C	7.72222400	0.24452300	0.44543000
C	7.94609300	-1.00964800	1.05164900
C	9.20625600	-1.60135000	1.05454200
C	10.27298800	-0.93543800	0.44710900
H	8.64219200	1.84423900	-0.68192600
H	7.11867100	-1.51218700	1.54294600
H	9.34992100	-2.56587900	1.53317300
C	-7.94609300	1.00972200	-1.05151500
C	-7.72222300	-0.24450100	-0.44540400
C	-8.80826300	-0.89920400	0.17511100
C	-10.06647300	-0.31578700	0.16674000
C	-10.27299200	0.93545100	-0.44699800
C	-9.20625800	1.60142000	-1.05436400
H	-7.11867000	1.51230700	-1.54276200
H	-8.64219600	-1.84431900	0.68180700
H	-9.34992200	2.56599100	-1.53291100
C	-11.36558100	-0.84427800	0.77637400
C	-12.33900800	0.27800000	0.41526300
C	-13.69654800	0.37873600	0.69724900
C	-14.40086000	1.50904400	0.26602400
C	-13.74988400	2.52625500	-0.43964100
C	-12.38669600	2.43190300	-0.72604700
C	-11.68424300	1.30387500	-0.29607400
H	-14.21208100	-0.40575400	1.24529000
H	-15.46177500	1.59709600	0.48122600
H	-14.31010000	3.39709600	-0.76741500
H	-11.88543800	3.22469300	-1.27429700
C	-11.24270400	-1.01163700	2.30752500
H	-10.49609000	-1.77335200	2.55562800
H	-10.94477800	-0.07337100	2.78350400
H	-12.19866600	-1.32594600	2.73930600
C	-11.78574000	-2.18384700	0.13119400
H	-11.87806100	-2.08648800	-0.95412000
H	-11.04751200	-2.96428900	0.34388200
H	-12.75025700	-2.51797400	0.52758800
C	11.78574300	2.18380400	-0.13137900
H	11.04751600	2.96422900	-0.34413300
H	11.87807300	2.08654400	0.95394300
H	12.75025800	2.51789000	-0.52781300
C	11.24268300	1.01139500	-2.30759700
H	10.94475000	0.07308600	-2.78348700
H	10.49607100	1.77309000	-2.55576400
H	12.19864300	1.32566100	-2.73941600

Optimized coordinates for NTC singlet state at RB3LYP/6-31G(d,p) level of theory and basis set.

C	-1.92938800	-1.70804600	0.00000000
C	-0.47771300	-1.79327500	0.00000000
C	0.34678800	-0.61135400	-0.00000200
C	-0.34678800	0.61135300	-0.00000400
C	-1.76575700	0.68605000	-0.00000600
C	-2.60138800	-0.41931700	-0.00000200
C	1.76575700	-0.68605100	-0.00000200
C	0.47771300	1.79327400	-0.00000600
C	1.92938800	1.70804600	-0.00000500
C	2.60138700	0.41931600	-0.00000200
H	2.19083700	-1.68430300	-0.00000300
H	-2.19083700	1.68430200	-0.00001200
N	0.01075800	3.04000300	-0.00000900
N	2.51809700	2.90470700	-0.00000600
S	1.32030800	4.03716900	-0.00001000
N	-2.51809700	-2.90470700	0.00000100
N	-0.01075900	-3.04000400	0.00000100
S	-1.32030800	-4.03717000	0.00000300

C	7.74101100	1.38384000	-0.00004100
C	8.57317600	0.10164600	-0.00001000
C	7.75501300	-1.01674800	0.00002300
C	6.38017500	-0.59346500	0.00001800
C	6.33119200	0.79218900	-0.00002000
C	4.04832300	0.30149700	0.00000200
C	5.01647600	1.29875800	-0.00002900
H	4.74661700	2.34566900	-0.00005700
C	10.15722500	-1.58800800	0.00002900
S	4.80982100	-1.30848100	0.00004800
H	11.09651700	-2.12300400	0.00004100
C	8.00039200	2.22773500	-1.26768100
H	7.34204800	3.10229800	-1.28768000
H	9.03570000	2.58407500	-1.28750100
H	7.82105000	1.64224300	-2.17327000
C	8.00040100	2.22780200	1.26755200
H	7.34205700	3.10236600	1.28751100
H	7.82106600	1.64235700	2.17317400
H	9.03570900	2.58414300	1.28734500
S	8.66074100	-2.49226900	0.00005700
C	9.95309700	-0.22972900	-0.00000500
H	10.76735600	0.48614300	-0.00002700
C	-7.74101100	-1.38384000	-0.00005000
C	-8.57317600	-0.10164500	-0.00001200
C	-7.75501300	1.01674900	0.00002700
C	-6.38017500	0.59346500	0.00001900
C	-6.33119300	-0.79218900	-0.00002700
C	-4.04832300	-0.30149800	0.00000000
C	-5.01647600	-1.29875800	-0.00003900
H	-4.74661800	-2.34567000	-0.00007300
C	-10.15722400	1.58800900	0.00003600
S	-4.80982000	1.30848000	0.00005200
H	-11.09651600	2.12300600	0.00005000
C	-8.00039400	-2.22772700	-1.26769400
H	-7.34205000	-3.10229100	-1.28769900
H	-9.03570100	-2.58406700	-1.28751500
H	-7.82105200	-1.64223000	-2.17328100
C	-8.00040100	-2.22780800	1.26753800
H	-7.34205600	-3.10237200	1.28749200
H	-7.82106600	-1.64236800	2.17316300
H	-9.03570900	-2.58415000	1.28733000
S	-8.66074000	2.49227000	0.00007000
C	-9.95309700	0.22973000	-0.00000500
H	-10.76735600	-0.48614100	-0.00003000

Optimized coordinates for 2N-NTC singlet state at RB3LYP/6-31G(d,p) level of theory and basis set.

C	-1.53925900	-2.06386500	-0.11152700
C	-0.10095400	-1.84961700	-0.11768900
C	0.46454900	-0.52624600	-0.04031400
C	-0.46454800	0.52624700	0.04031300
C	-1.86809600	0.30859300	0.04579800
C	-2.46036600	-0.94266000	-0.02648500
C	1.86809600	-0.30859100	-0.04579900
C	0.10095500	1.84961800	0.11768700
C	1.53925900	2.06386600	0.11152500
C	2.46036600	0.94266100	0.02648200
H	2.48832200	-1.19639400	-0.11168400
H	-2.48832200	1.19639500	0.11168200
N	-0.61092000	2.97152200	0.19982000
N	1.87085200	3.35314300	0.18997300
S	0.46690200	4.21393500	0.26492800
N	-1.87085200	-3.35314200	-0.18997500
N	0.61092100	-2.97152100	-0.19982000
S	-0.46690100	-4.21393400	-0.26493000
C	7.29150300	2.93950600	0.08663300
C	8.36770900	1.85745800	-0.00023400
C	7.79426900	0.59614400	-0.07064100
C	6.36561000	0.72740100	-0.04190700

C	6.03311700	2.07229300	0.04874900
C	3.89986300	1.12350700	0.01785200
C	4.64397700	2.29676300	0.08256400
H	4.16518200	3.26362800	0.15154600
C	10.28807600	0.53320800	-0.10015000
S	4.97431200	-0.29316200	-0.08785700
C	7.35443500	3.89877700	-1.12256700
H	6.53102100	4.61923100	-1.08369900
H	8.29467100	4.46015400	-1.11959900
H	7.28535400	3.34895400	-2.06489900
C	7.39166300	3.73442700	1.40736400
H	6.56901400	4.45251700	1.48596800
H	7.34882000	3.06719100	2.27222200
H	8.33265700	4.29290800	1.44990300
S	8.98022900	-0.65877100	-0.16956900
C	9.77880400	1.81936600	-0.01884100
H	10.41834500	2.69135700	0.05467400
C	-7.29150300	-2.93950500	-0.08665500
C	-8.36770900	-1.85745800	0.00022100
C	-7.79426900	-0.59614500	0.07064000
C	-6.36561000	-0.72740100	0.04190600
C	-6.03311700	-2.07229200	-0.04876200
C	-3.89986300	-1.12350600	-0.01785500
C	-4.64397600	-2.29676200	-0.08257800
H	-4.16518100	-3.26362600	-0.15156900
C	-10.28807600	-0.53320900	0.10014800
S	-4.97431300	0.29316200	0.08786800
C	-7.39166200	-3.73441400	-1.40739400
H	-6.56901200	-4.45250300	-1.48600500
H	-8.33265500	-4.29289400	-1.44994000
H	-7.34881700	-3.06717000	-2.27224600
C	-7.35443600	-3.89878700	1.12253500
H	-6.53102200	-4.61924100	1.08366200
H	-7.28535600	-3.34897400	2.06487300
H	-8.29467200	-4.46016500	1.11956100
S	-8.98023000	0.65876900	0.16958000
C	-9.77880300	-1.81936700	0.01882600
H	-10.41834500	-2.69135700	-0.05469600
C	-15.18490700	3.22346200	-0.58098900
C	-13.85699100	2.86323500	-0.61856300
C	-13.44467700	1.56141000	-0.22212900
C	-14.43910500	0.62711100	0.21435100
C	-15.79894500	1.02964500	0.24255700
C	-16.16602900	2.29918700	-0.14609800
H	-11.35012400	1.87148300	-0.61907200
H	-15.48597300	4.22145600	-0.88559200
H	-13.10330100	3.57180500	-0.95180600
C	-12.08664500	1.15942900	-0.25597300
C	-14.01586300	-0.67208800	0.60693200
H	-16.54892400	0.31725400	0.57647800
H	-17.21022700	2.59623100	-0.12113800
C	-12.69223800	-1.03021300	0.57371000
C	-11.68843900	-0.11237500	0.13256100
H	-14.76189300	-1.38371600	0.95109600
H	-12.39404300	-2.01761100	0.91021800
C	12.69223700	1.03020500	-0.57372100
C	14.01586200	0.67207900	-0.60694000
C	14.43910400	-0.62711400	-0.21434400
C	13.44467700	-1.56140800	0.22214800
C	12.08664500	-1.15942700	0.25599000
C	11.68843900	0.11237300	-0.13256000
H	16.54892300	-0.31726300	-0.57647800
H	12.39404200	2.01760000	-0.91024100
H	14.76189200	1.38370300	-0.95111400
C	15.79894400	-1.02964900	-0.24254800
C	13.85699100	-2.86322800	0.61859800
H	11.35012400	-1.87147600	0.61909800
C	15.18490700	-3.22345600	0.58102700
C	16.16602800	-2.29918600	0.14612200

H	13.10330200	-3.57179400	0.95185100
H	15.48597400	-4.22144600	0.88564200
H	17.21022700	-2.59623100	0.12116500

Optimized coordinates for Flu-NTC singlet state at RB3LYP/6-31G(d,p) level of theory and basis set.

C	-1.41735300	-2.15234600	0.19173800
C	0.00644300	-1.85609800	0.19145600
C	0.49443600	-0.49997500	0.19106200
C	-0.49443700	0.49996400	0.19102000
C	-1.88313000	0.20230600	0.19122200
C	-2.40216400	-1.08323400	0.19134200
C	1.88312900	-0.20231600	0.19128200
C	-0.00644300	1.85608700	0.19134400
C	1.41735200	2.15233600	0.19164300
C	2.40216200	1.08322400	0.19133300
H	2.55388500	-1.05513300	0.19197300
H	-2.55388700	1.05512300	0.19183700
N	-0.78221300	2.93790500	0.19140600
N	1.67374700	3.46097800	0.19193700
S	0.22202700	4.24225800	0.19190000
N	-1.67374800	-3.46098800	0.19210500
N	0.78221200	-2.93791500	0.19160200
S	-0.22202800	-4.24226800	0.19215000
C	7.11057600	3.35544200	0.19987600
C	8.24795600	2.33406300	0.19444700
C	7.74801700	1.03980000	0.18429500
C	6.31409500	1.09003000	0.18545900
C	5.90409100	2.41652100	0.19525800
C	3.82873200	1.34616600	0.19100200
C	4.50417900	2.56206800	0.19826500
H	3.97030800	3.50218500	0.20478400
C	10.24282400	1.12065700	0.16659000
S	4.98382300	-0.00997000	0.17979400
C	7.14114700	4.24428400	-1.06310700
H	6.27816000	4.91779400	-1.07899600
H	8.04843100	4.85726400	-1.08030300
H	7.11955200	3.63708900	-1.97188900
C	7.14187900	4.23077700	1.47228300
H	6.27832500	4.90331100	1.49637300
H	7.12186700	3.61394500	2.37460900
H	8.04862500	4.84438900	1.49476700
S	9.00551500	-0.14757200	0.17159600
C	9.65878700	2.37735400	0.18394600
H	10.24711700	3.28735000	0.15804500
C	-7.11057900	-3.35545100	0.19974200
C	-8.24795800	-2.33407200	0.19436600
C	-7.74801900	-1.03980800	0.18429000
C	-6.31409700	-1.09004000	0.18545900
C	-5.90409300	-2.41653100	0.19518400
C	-3.82873400	-1.34617600	0.19100000
C	-4.50418100	-2.56207900	0.19819100
H	-3.97031100	-3.50219700	0.20466200
C	-10.24282600	-1.12066300	0.16656300
S	-4.98382500	0.00996100	0.17987000
C	-7.14114400	-4.24422300	-1.06329100
H	-6.27815700	-4.91773100	-1.07921500
H	-8.04842800	-4.85720100	-1.08052600
H	-7.11954500	-3.63697600	-1.97203800
C	-7.14188800	-4.23085800	1.47209900
H	-6.27833500	-4.90339500	1.49615500
H	-7.12188000	-3.61407800	2.37446000
H	-8.04863500	-4.84447200	1.49454400
S	-9.00551600	0.14756500	0.17165300
C	-9.65878900	-2.37736200	0.18385200
H	-10.24712200	-3.28735500	0.15789300
C	-12.61504900	-1.68734600	0.66613300
C	-11.66455600	-0.77796400	0.15341300
C	-12.12153800	0.45216700	-0.36817900

C	-13.47556000	0.74629400	-0.37089800
C	-14.41180500	-0.17746800	0.13522000
C	-13.97622000	-1.39961700	0.65336300
H	-12.27280300	-2.62084600	1.10086300
H	-11.40636400	1.15540800	-0.78682800
H	-14.68388100	-2.11809900	1.05740500
C	-14.17611500	2.00413600	-0.88595500
C	-15.63888500	1.66685300	-0.59592500
C	-16.77440200	2.43035300	-0.84055800
C	-18.02951900	1.91861700	-0.49022300
C	-18.14300500	0.65499300	0.09868200
C	-17.00697200	-0.11754100	0.34723600
C	-15.75461900	0.39328500	-0.00202000
H	-16.69742200	3.41350500	-1.29773400
H	-18.92265800	2.50762600	-0.67720200
H	-19.12377200	0.27169100	0.36488200
H	-17.10044100	-1.09847800	0.80489900
C	18.14300500	-0.65498900	0.09868700
C	18.02952300	-1.91859300	-0.49026300
C	16.77440900	-2.43031800	-0.84062200
C	15.63889000	-1.66682700	-0.59596900
C	15.75462000	-0.39328000	-0.00201900
C	17.00697100	0.11753400	0.34726300
H	19.12377100	-0.27169600	0.36490600
H	18.92266400	-2.50759500	-0.67725700
H	16.69743200	-3.41345300	-1.29783300
H	17.10043600	1.09845600	0.80496000
C	14.17612200	-2.00410100	-0.88601900
C	13.47556300	-0.74627700	-0.37092200
C	12.12154100	-0.45215100	-0.36820000
C	11.66455500	0.77796000	0.15343400
C	12.61504400	1.68732500	0.66619300
C	13.97621600	1.39959700	0.65342000
C	14.41180400	0.17746700	0.13523500
H	11.40637000	-1.15537800	-0.78687800
H	12.27279600	2.62080800	1.10095600
H	14.68387400	2.11806400	1.05749200
C	13.72169600	-3.25932000	-0.10799600
H	13.88426100	-3.13710300	0.96642900
H	12.65664300	-3.45282000	-0.27322100
H	14.27854700	-4.14175100	-0.44006900
C	13.93390100	-2.20435300	-2.39886700
H	12.87241400	-2.38145800	-2.60125400
H	14.24844800	-1.32510300	-2.96786200
H	14.49461300	-3.06940200	-2.76811100
C	-13.93388500	2.20444300	-2.39879400
H	-12.87239700	2.38155400	-2.60116900
H	-14.24842900	1.32521200	-2.96782200
H	-14.49459400	3.06950400	-2.76801100
C	-13.72169300	3.25932800	-0.10788400
H	-13.88426500	3.13707300	0.96653600
H	-12.65663900	3.45283300	-0.27309700
H	-14.27854100	4.14177100	-0.43993000

Optimized coordinates for TPAOMe-BTT singlet state at RB3LYP/6-31G(d,p) level of theory and basis set.

C	2.93727700	-2.64806800	0.09640400
C	3.84934300	-3.68033900	0.17769700
C	5.21496100	-3.27961000	0.16019300
H	3.53772300	-4.71228100	0.26550300
C	5.36386400	-1.90586900	0.05951300
C	-2.93727700	-2.64806800	0.09640100
C	-3.84934400	-3.68034100	0.17768400
C	-5.21496200	-3.27961000	0.16018000
H	-3.53772300	-4.71228300	0.26548000
C	-5.36386400	-1.90586900	0.05951400
S	-3.79742800	-1.11870200	-0.02439300
S	3.79742800	-1.11870300	-0.02440700

C	6.58000100	-1.08762200	0.01585000
C	6.64672800	0.16219100	0.66203700
C	7.72523000	-1.50483700	-0.68990800
C	7.79229600	0.94498600	0.62207500
H	5.79247400	0.51063700	1.23507500
C	8.87413000	-0.72612100	-0.74048900
H	7.70080100	-2.43591100	-1.24548500
C	8.93381300	0.51566400	-0.08107600
H	7.81414800	1.89229900	1.14900200
H	9.73057000	-1.06963700	-1.30989900
C	-6.58000200	-1.08762200	0.01585300
C	-6.64673500	0.16218100	0.66205900
C	-7.72522400	-1.50482700	-0.68992100
C	-7.79230200	0.94497600	0.62209900
H	-5.79248400	0.51061900	1.23510900
C	-8.87412400	-0.72611000	-0.74050000
H	-7.70078900	-2.43589300	-1.24551200
C	-8.93381400	0.51566400	-0.08106800
H	-7.81415900	1.89228100	1.14904000
H	-9.73055900	-1.06961800	-1.30992200
N	10.09932200	1.30760400	-0.12868200
N	-10.09932200	1.30760400	-0.12867200
C	-10.01247800	2.73043600	-0.09140500
C	-10.83415300	3.47405600	0.76135100
C	-9.11551400	3.41888200	-0.92735400
C	-10.77811000	4.86930900	0.78280000
H	-11.53199400	2.95686000	1.41164800
C	-9.03780000	4.80344400	-0.89532900
H	-8.47647800	2.85744800	-1.60091600
C	-9.87190700	5.54284800	-0.04322600
H	-11.43378800	5.41208700	1.45305300
H	-8.34652000	5.33935500	-1.53727600
C	-11.38832400	0.70146800	-0.19364400
C	-11.73759000	-0.34569100	0.66558400
C	-12.34425800	1.15740000	-1.11777100
C	-12.99864800	-0.94216900	0.60112000
H	-11.01346700	-0.70420000	1.38984900
C	-13.60690200	0.58537500	-1.17247700
H	-12.08823900	1.96903600	-1.79076300
C	-13.94477100	-0.47411600	-0.31741100
H	-13.23194200	-1.75410000	1.27948600
H	-14.34853500	0.93433300	-1.88339800
C	11.38832500	0.70146600	-0.19363200
C	12.34426800	1.15738400	-1.11775500
C	11.73758200	-0.34568000	0.66561600
C	13.60691300	0.58535900	-1.17244100
H	12.08825600	1.96901000	-1.79076300
C	12.99864100	-0.94215900	0.60117300
H	11.01345200	-0.70417900	1.38987900
C	13.94477300	-0.47412000	-0.31735600
H	14.34855200	0.93430600	-1.88335900
H	13.23192800	-1.75408000	1.27955300
C	10.01247800	2.73043500	-0.09143700
C	10.83414500	3.47406800	0.76131600
C	9.11552300	3.41887000	-0.92740600
C	10.77810200	4.86932200	0.78274300
H	11.53197800	2.95688300	1.41162800
C	9.03780900	4.80343200	-0.89540300
H	8.47649400	2.85742500	-1.60096600
C	9.87190700	5.54284900	-0.04330300
H	11.43377300	5.41211000	1.45299500
H	8.34653600	5.33933400	-1.53736500
O	-15.20883900	-0.97375000	-0.45987800
O	-9.72448700	6.90027900	-0.09804800
O	9.72448900	6.90027800	-0.09814700
O	15.20884300	-0.97375600	-0.45980300
C	-15.60243300	-2.04588500	0.38082600
H	-16.62849800	-2.28837200	0.10053400
H	-14.97002100	-2.93095200	0.23315100

H	-15.57649600	-1.76163600	1.44084300
C	-10.54180000	7.69597800	0.74458700
H	-10.36530500	7.47732800	1.80576400
H	-10.26590400	8.73184600	0.54203900
H	-11.60830600	7.55764300	0.52421300
C	10.54179300	7.69599100	0.74448400
H	10.26589900	8.73185600	0.54191700
H	10.36528700	7.47735700	1.80566300
H	11.60830200	7.55765100	0.52412300
C	15.60242800	-2.04587800	0.38092100
H	16.62849600	-2.28836900	0.10064200
H	15.57648000	-1.76161400	1.44093300
H	14.97001700	-2.93094800	0.23325300
C	1.48319000	-2.68617100	0.10324400
C	0.70579300	-1.53652000	0.12696900
C	0.73109000	-3.91855500	0.08307900
C	-0.70579300	-1.53652000	0.12696800
C	-0.73108900	-3.91855500	0.08307800
C	-1.48319000	-2.68617100	0.10324300
N	1.25372600	-5.14801400	0.06030200
N	-1.25372500	-5.14801500	0.06030100
S	0.00000100	-6.20852800	0.04091800
C	-6.33781300	-4.27655200	0.29373500
H	-7.22997400	-3.82465800	0.73407400
H	-6.62692000	-4.70002700	-0.67639500
H	-6.02992200	-5.11289800	0.92881700
C	6.33781200	-4.27655000	0.29376000
H	6.62692500	-4.70003100	-0.67636600
H	7.22997100	-3.82465200	0.73410000
H	6.02991800	-5.11289200	0.92884500
H	-1.19866900	-0.56958800	0.15473900
H	1.19866800	-0.56958800	0.15474100

Optimized coordinates for TPAOMe-NTT singlet state at RB3LYP/6-31G(d,p) level of theory and basis set.

C	1.20967100	-1.59548400	1.62156800
C	-0.17932600	-1.32759800	1.28440600
C	-0.53762700	-0.33793300	0.30029700
C	0.53763200	0.33797100	-0.30022900
C	1.89363800	0.07013900	0.03682400
C	2.28742100	-0.87040800	0.97229900
C	-1.89363200	-0.07010400	-0.03675900
C	0.17933200	1.32763600	-1.28433800
C	-1.20966500	1.59552100	-1.62150200
C	-2.28741500	0.87044100	-0.97223600
H	-2.64265000	-0.65751600	0.48379400
H	2.64265600	0.65754900	-0.48373200
N	1.05194800	2.07204300	-1.96063600
N	-1.34421100	2.54323200	-2.55035500
S	0.17238700	3.04501500	-2.95562100
N	1.34421700	-2.54319500	2.55042300
N	-1.05194200	-2.07200300	1.96070700
S	-0.17238000	-3.04497400	2.95569300
C	-3.68772800	1.11871400	-1.28039600
C	-4.24678400	1.92302600	-2.25257600
S	-4.96653000	0.33697200	-0.36169000
C	-5.66913500	1.91872800	-2.29097200
H	-3.63946000	2.49561300	-2.94038700
C	-6.22258700	1.09526200	-1.32349300
C	3.68773500	-1.11868900	1.28045200
C	4.24679200	-1.92302400	2.25261100
S	4.96653600	-0.33693500	0.36175300
C	5.66914400	-1.91873600	2.29099700
H	3.63946900	-2.49562200	2.94041400
C	6.22259500	-1.09525400	1.32353200
C	-6.44073200	2.69970100	-3.32383400
H	-6.63269100	3.73006500	-2.99882100
H	-5.87348900	2.75916500	-4.25767500
H	-7.40705900	2.23814100	-3.54102800

C	6.44074600	-2.69973600	3.32383600
H	5.87351800	-2.75920400	4.25768600
H	6.63268200	-3.73009800	2.99880400
H	7.40708400	-2.23819500	3.54101900
C	-9.94980200	1.52816500	-0.79777700
C	-8.61806000	1.80380900	-1.07968300
C	-7.62887300	0.80295100	-1.02517700
C	-8.04526900	-0.48902400	-0.64967800
C	-9.37073400	-0.77021300	-0.34839900
C	-10.35532700	0.23335800	-0.42328700
H	-8.33741700	2.82034800	-1.33277000
H	-7.31398900	-1.29024300	-0.59869700
H	-9.65304700	-1.77556100	-0.05704200
C	8.04528800	0.48903300	0.64975100
C	7.62888100	-0.80295100	1.02520800
C	8.61805500	-1.80382400	1.07966200
C	9.94979500	-1.52818900	0.79774000
C	10.35533200	-0.23337500	0.42328800
C	9.37075200	0.77021300	0.34845800
H	7.31401900	1.29026400	0.59881300
H	8.33740200	-2.82036900	1.33271800
H	9.65307400	1.77556800	0.05713300
H	10.68560700	-2.32226700	0.85678300
H	-10.68562400	2.32222900	-0.85686200
N	-11.70503800	-0.04913600	-0.13103000
N	11.70504000	0.04911000	0.13101100
C	-12.54678000	0.94371800	0.45224600
C	-13.82955700	1.18508300	-0.06879500
C	-12.12568700	1.68634700	1.56027100
C	-14.66123600	2.13483100	0.50637700
H	-14.17015300	0.61629000	-0.92772300
C	-12.94793000	2.66032600	2.13073600
H	-11.14084300	1.50661700	1.97893600
C	-14.22631600	2.88607300	1.60841600
H	-15.65281100	2.32431100	0.10880700
H	-12.58620800	3.21943000	2.98534100
C	-12.25016600	-1.34147400	-0.38916500
C	-13.01609200	-1.99968100	0.57790100
C	-12.04999100	-1.97225800	-1.62979700
C	-13.57995400	-3.25168300	0.32320400
H	-13.18015600	-1.52493800	1.53968600
C	-12.58752500	-3.22583500	-1.88215300
H	-11.46503900	-1.47149000	-2.39424100
C	-13.36199300	-3.87613500	-0.90958700
H	-14.17167900	-3.72916100	1.09497800
H	-12.43467800	-3.71767700	-2.83708400
C	12.54675700	-0.94372900	-0.45232300
C	13.82955400	-1.18511000	0.06866200
C	12.12562300	-1.68632800	-1.56035300
C	14.66121100	-2.13484200	-0.50656700
H	14.17018400	-0.61634000	0.92759200
C	12.94784300	-2.66029100	-2.13087600
H	11.14076300	-1.50658600	-1.97897500
C	14.22624900	-2.88605300	-1.60861000
H	15.65280100	-2.32433300	-0.10904100
H	12.58608900	-3.21937100	-2.98548200
C	12.25018700	1.34143500	0.38917500
C	13.01607100	1.99967800	-0.57789800
C	12.05007200	1.97216800	1.62984400
C	13.57995200	3.25166700	-0.32317400
H	13.18008800	1.52497500	-1.53971100
C	12.58762500	3.22573100	1.88222800
H	11.46515200	1.47137100	2.39429200
C	13.36205100	3.87606700	0.90965300
H	14.17164400	3.72917500	-1.09495500
H	12.43482500	3.71753400	2.83718700
O	-15.11674500	3.80277600	2.09211400
O	-13.85468400	-5.10055000	-1.26321500
O	13.85476700	5.10046400	1.26331000

O	15.11666000	-3.80274300	-2.09236700
C	-14.72813300	4.58862000	3.20707900
H	-15.57192500	5.24591600	3.42181300
H	-14.52146000	3.96836000	4.08887600
H	-13.84337200	5.19919000	2.98481300
C	-14.64118100	-5.80384200	-0.31543900
H	-14.92683600	-6.74063300	-0.79602000
H	-14.07412800	-6.02597900	0.59784500
H	-15.54759700	-5.24615600	-0.04617700
C	14.64122100	5.80379400	0.31552600
H	14.07412400	6.02597100	-0.59772200
H	14.92690200	6.74056400	0.79613300
H	15.54762200	5.24611600	0.04619600
C	14.72800600	-4.58856000	-3.20733700
H	14.52130000	-3.96827900	-4.08911200
H	15.57178900	-5.24585100	-3.42211800
H	13.84325300	-5.19913500	-2.98505200

Optimized coordinates for TPAOMe-BBTT singlet state at UB3LYP/6-31G(d,p) level of theory and basis set.

C	-2.81558900	0.92740700	0.00082300
C	-3.29072100	2.23849300	0.05361700
C	-4.69441800	2.37482800	0.03882900
H	-2.61767800	3.08120400	0.11963900
C	-5.34312600	1.14257900	-0.02300000
C	2.81558900	-0.92740600	-0.00082000
C	3.29072100	-2.23849300	-0.05361500
C	4.69441700	-2.37482800	-0.03882700
H	2.61767700	-3.08120500	-0.11963600
C	5.34312600	-1.14257900	0.02300200
S	4.19087200	0.17732600	0.07392900
S	-4.19087200	-0.17732600	-0.07392700
C	-6.77312300	0.83861300	-0.03784400
C	-7.28086700	-0.32369700	0.58017400
C	-7.70642600	1.67336300	-0.68686700
C	-8.63354800	-0.62847400	0.56646900
H	-6.60137400	-0.98219700	1.11276000
C	-9.06099100	1.37254200	-0.71261200
H	-7.35954000	2.54947600	-1.22257000
C	-9.55667000	0.21544800	-0.08170600
H	-8.98787500	-1.51919700	1.07284200
H	-9.74527800	2.02667800	-1.24124000
C	6.77312300	-0.83861300	0.03784600
C	7.28086700	0.32369700	-0.58017300
C	7.70642600	-1.67336300	0.68686800
C	8.63354800	0.62847400	-0.56646900
H	6.60137400	0.98219700	-1.11275900
C	9.06099200	-1.37254300	0.71261200
H	7.35954100	-2.54947600	1.22257100
C	9.55667000	-0.21544800	0.08170600
H	8.98787400	1.51919700	-1.07284200
H	9.74527900	-2.02667800	1.24124000
N	-10.93088600	-0.09057300	-0.10284500
N	10.93088600	0.09057200	0.10284400
C	11.37504900	1.44588900	0.08352700
C	12.39104300	1.85119900	-0.78740300
C	10.81839400	2.39802800	0.95571300
C	12.85403200	3.16861000	-0.79264600
H	12.83065000	1.12691500	-1.46526500
C	11.25758600	3.71360300	0.94097200
H	10.03494700	2.09759200	1.64359500
C	12.28302600	4.11101500	0.06956600
H	13.64588600	3.44553000	-1.47820600
H	10.83072300	4.45284700	1.61059500
C	11.90826100	-0.94790900	0.12703300
C	11.83517100	-2.02775600	-0.75908000
C	12.97887600	-0.89697500	1.03617900
C	12.79034200	-3.04610000	-0.73545500
H	11.01895400	-2.07694000	-1.47239800

C	13.94392700	-1.89331100	1.05099300
H	13.04882000	-0.06513300	1.72926800
C	13.85611200	-2.98082900	0.16906500
H	12.69933600	-3.86932000	-1.43387800
H	14.77276700	-1.85880700	1.75026300
C	-11.90826100	0.94790800	-0.12703500
C	-12.97887500	0.89697500	-1.03618100
C	-11.83517100	2.02775500	0.75907900
C	-13.94392600	1.89331100	-1.05099500
H	-13.04881800	0.06513300	-1.72927000
C	-12.79034200	3.04610000	0.73545400
H	-11.01895500	2.07694000	1.47239800
C	-13.85611200	2.98082900	-0.16906700
H	-14.77276500	1.85880700	-1.75026600
H	-12.69933700	3.86931900	1.43387700
C	-11.37504800	-1.44589000	-0.08352800
C	-12.39104300	-1.85119900	0.78740100
C	-10.81839300	-2.39802800	-0.95571400
C	-12.85403200	-3.16861000	0.79264400
H	-12.83065100	-1.12691500	1.46526300
C	-11.25758500	-3.71360300	-0.94097400
H	-10.03494600	-2.09759200	-1.64359500
C	-12.28302500	-4.11101500	-0.06956800
H	-13.64588600	-3.44553100	1.47820300
H	-10.83072200	-4.45284700	-1.61059700
O	14.85042400	-3.91204300	0.27268100
O	12.64916100	5.42504900	0.14305900
O	-12.64916100	-5.42504900	-0.14306200
O	-14.85042400	3.91204300	-0.27268400
C	14.81086600	-5.03260700	-0.59599500
H	15.68072400	-5.64074700	-0.34379900
H	13.90034200	-5.62793200	-0.44929500
H	14.87505700	-4.73196800	-1.64978800
C	13.67827300	5.88205000	-0.71939500
H	13.40371000	5.76615800	-1.77586000
H	13.80900300	6.94218200	-0.49778400
H	14.62449500	5.35645000	-0.53645700
C	-13.67827300	-5.88205100	0.71939200
H	-13.80900200	-6.94218300	0.49778100
H	-13.40371000	-5.76615800	1.77585700
H	-14.62449500	-5.35645000	0.53645400
C	-14.81086700	5.03260600	0.59599300
H	-15.68072400	5.64074600	0.34379600
H	-14.87505800	4.73196700	1.64978500
H	-13.90034300	5.62793200	0.44929300
C	-1.44916300	0.48387300	-0.00016300
C	-0.32365100	1.37216700	0.03288700
C	-1.06926500	-0.89540400	-0.03458900
C	1.06926400	0.89540400	0.03459100
C	0.32365000	-1.37216600	-0.03288500
C	1.44916300	-0.48387300	0.00016600
N	-1.95927900	-1.89606400	-0.07207400
N	0.40547200	-2.71110500	-0.06692800
N	-0.40547300	2.71110500	0.06693100
N	1.95927800	1.89606500	0.07207600
S	-1.12228400	-3.30329200	-0.10008000
S	1.12228300	3.30329300	0.10008200
C	5.36350300	-3.72216600	-0.14090900
H	6.31298600	-3.66312600	-0.67959700
H	5.57244000	-4.15363000	0.84605500
H	4.71537100	-4.42791800	-0.66849200
C	-5.36350300	3.72216500	0.14091100
H	-5.57244100	4.15363000	-0.84605200
H	-6.31298600	3.66312600	0.67959900
H	-4.71537100	4.42791800	0.66849500

Supplementary Reference

1. Dimroth, K.; Umbach, W.; Blöcher, K. H., Bis-phenoxy-Radicals of the Polyphenyl Series. *Angew. Chem. Int. Ed.* **1963,** *2* (10), 620-621.
2. Zeng, Z.; Sung, Y. M.; Bao, N.; Tan, D.; Lee, R.; Zafra, J. L.; Lee, B. S.; Ishida, M.; Ding, J.; Lopez Navarrete, J. T.; Li, Y.; Zeng, W.; Kim, D.; Huang, K. W.; Webster, R. D.; Casado, J.; Wu, J., Stable tetrabenzo-Chichibabin's hydrocarbons: tunable ground state and unusual transition between their closed-shell and open-shell resonance forms. *J. Am. Chem. Soc.* **2012,** *134* (35), 14513-14525.
3. Casado, J.; Miller, L. L.; Mann, K. R.; Pappenfus, T. M.; Higuchi, H.; Ortí, E.; Milián, B.; Pou-Amérigo, R.; Hernández, V.; López Navarrete, J. T., Quinonoid oligothiophenes as electron-donor and electron-acceptor materials. A spectroelectrochemical and theoretical study. *J. Am. Chem. Soc.* **2002,** *124* (41), 12380-12388.
4. Takahashi, T.; Matsuoka, K.-i.;Takimiya, K.; Otsubo, T.; Aso, Y., Extensive quinoidal oligothiophenes with dicyanomethylene groups at terminal positions as highly amphoteric redox molecules. *J. Am. Chem. Soc.* **2005,** *127* (25), 8928-8929.
5. Qiao, Y.; Guo, Y.; Yu, C.; Zhang, F.; Xu, W.; Liu, Y.; Zhu, D. Diketopyrrolopyrrole-containing quinoidal small molecules for high-performance, air-stable, and solution-processable *n*-channel organic field-effect transistors. *J. Am. Chem. Soc.* **2012,** *134* (9), 4084-4087.
6. Xia, D.; Keerthi, A.; An, C.; Baumgarten, M., Synthesis of a quinoidal dithieno[2,3-d;2′,3′-d]benzo[2,1-*b*;3,4-*b*′]-dithiophene based open-shell singlet biradicaloid. *Org. Chem. Front.* **2017,** *4* (1), 18-21.
7. Zhang, C.; Medina Rivero, S.; Liu, W.; Casanova, D.; Zhu, X.; Casado, J., Stable cross-conjugated tetrathiophene diradical. *Angew. Chem. Int. Ed.* **2019,** *58* (33), 11291-11295.
8. Yang, K.; Zhang, X.; Harbuzaru, A.; Wang, L.; Wang, Y.; Koh, C.; Guo, H.; Shi, Y.; Chen, J.; Sun, H.; Feng, K.; Ruiz Delgado, M. C.; Woo, H. Y.; Ortiz, R. P.; Guo, X., Stable organic diradicals based on fused quinoidal oligothiophene imides with high electrical conductivity. *J. Am. Chem. Soc.* **2020**. 142 (9), 4329–4340
9. Chase, D. T.; Rose, B. D.; McClintock, S. P.; Zakharov, L. N.; Haley, M. M., Indeno[1,2-b]fluorenes: fully conjugated antiaromatic analogues of acenes. *Angew. Chem. Int. Ed.* **2011,** *50* (5), 1127-1130.
10. Shimizu, A.; Kishi, R.; Nakano, M.; Shiomi, D.; Sato, K.; Takui, T.; Hisaki, I.; Miyata, M.; Tobe, Y., Indeno[2,1-*b*]fluorene: a 20-pi-electron hydrocarbon with very low-energy light absorption. *Angew. Chem. Int. Ed.* **2013,** *52* (23), 6076-6079.
11. Barker, J. E.; Dressler, J. J.; Cardenas Valdivia, A.; Kishi, R.; Strand, E. T.; Zakharov, L. N.; MacMillan, S. N.; Gomez-Garcia, C. J.; Nakano, M.; Casado, J.; Haley, M. M., Molecule isomerism modulates the diradical properties of sSupplementary Table inglet diradicaloids. *J. Am. Chem. Soc.* **2020,** *142* (3), 1548-1555.
12. Hiroto, S.; Furukawa, K.; Shinokubo, H.; Osuka, A., Synthesis and biradicaloid character of doubly linked corrole dimers. *J. Am. Chem. Soc.* **2006,** *128* (38), 12380-12381.
13. Konishi, A.; Hirao, Y.; Nakano, M.; Shimizu, A.; Botek, E.; Champagne, B.; Shiomi, D.; Sato, K.; Takui, T.; Matsumoto, K.; Kurata, H.; Kubo, T., Synthesis and characterization of teranthene: a singlet biradical polycyclic aromatic hydrocarbon having kekulé structures. *J. Am. Chem. Soc.* **2010,** *132* (32), 11021-11023.
14. Sun, Z.; Huang, K.-W.; Wu, J., Soluble and stable zethrenebis(dicarboximide) and its quinone. *Org. Lett.* **2010,** *12* (20), 4690-4693.

15. Rana, A.; Hong, Y.; Gopalakrishna, T. Y.; Phan, H.; Herng, T. S.; Yadav, P.; Ding, J.; Kim, D.; Wu, J., Stable expanded porphycene-based diradicaloid and tetraradicaloid. *Angew. Chem. Int. Ed.* **2018,** *57* (38), 12534-12537.

16. Benincori, T.; Appoloni, G.; Mussini, P. R.; Arnaboldi, S.; Cirilli, R.; Quartapelle Procopio, E.; Panigati, M.; Abbate, S.; Mazzeo, G.; Longhi, G., Searching for models exhibiting high circularly polarized luminescence: electroactive inherently chiral oligothiophenes. *Chem. Eur. J.* **2018,** *24* (43), 11082-11093.

17. Du, T.; Gao, R.; Deng, Y.; Wang, C.; Zhou, Q.; Geng, Y., Indandione-terminated quinoids: facile synthesis by alkoxide-mediated rearrangement reaction and semiconducting properties. *Angew. Chem. Int. Ed.* **2020,** *59* (1), 221-225.

18. Wang, M.; Hu, X.; Liu, P.; Li, W.; Gong, X.; Huang, F.; Cao, Y., Donor-acceptor conjugated polymer based on naphtho[1,2-c:5,6-c]bis[1,2,5]thiadiazole for high-performance polymer solar cells. *J. Am. Chem. Soc.* **2011,** *133* (25), 9638-9641.

19. Li, Y.; Li, L.; Wu, Y.; Li, Y., A review on the origin of synthetic metal radical: singlet open-shell radical ground state? *J. Phys. Chem. C* **2017,** *121* (15), 8579-8588.

20. Canola S, Casado J, Negri F. The double exciton state of conjugated chromophores with strong diradical character: insights from TDDFT calculations. *Phys. Chem. Chem. Phys*. **2018**, *20* (37), 24227-24238.

21. Noodleman L. Valence bond description of antiferromagnetic coupling in transition metal dimers. *J. Chem. Phys.* 1981, 74(10), 5737-5743.

22. Rudebusch GE, Zafra JL, Jorner K, Fukuda K, Marshall JL, Arrechea-Marcos I, Espejo GL, Ortiz RP, Gómez-García CJ, Zakharov LN, Nakano M, Ottosson H, Casado, J and Haley, MM. Diindeno-fusion of an anthracene as a design strategy for stable organic biradicals. *Nat. Chem.* **2016**, *8* (8), 753-759.

23. Dressler JJ, Teraoka M, Espejo GL, Kishi R, Takamuku S, Gómez-García CJ, Zakharov LN, Nakano M, Casado J, Haley MM. Thiophene and its sulfur inhibit indenoindenodibenzothiophene diradicals from low-energy lying thermal triplets. *Nat. Chem.* **2018**, *10*(11), 1134-1140.

24. Stuyver T, Chen B, Zeng T, Geerlings P, De Proft F, Hoffmann R. Do diradicals behave like radicals? *Chem. Rev.* **2019**, *119* (21), 11291-11351.

25. Sabuj MA, Huda MM, Rai N. Donor-acceptor conjugated macrocycles with polyradical character and global aromaticity. *Iscience* **2020**, *23*(11), 101675.

26. Sabuj MA, Huda MM, Sarap CS, Rai N. Benzobisthiadiazole-based high-spin donor–acceptor conjugated polymers with localized spin distribution. *Mater. Adv.* **2021**, *2*, 2943 - 2955.

27. Grimme S, Hansen A. A practicable real‐space measure and visualization of static electron‐correlation effects. *Angew. Chem. Int. Ed.* **2015**, *54*(42), 12308-12313.

28. Pérez-Guardiola A, Sandoval-Salinas ME, Casanova D, San-Fabián E, Pérez-Jiménez AJ, Sancho-Garcia JC. The role of topology in organic molecules: origin and comparison of the radical character in linear and cyclic oligoacenes and related oligomers. *Phys. Chem. Chem. Phys*. **2018**, *20*(10), 7112-7124.
